# Harnessing artificial intelligence for genomic variant prediction: advances, challenges, and future directions

**DOI:** 10.1093/gigascience/giag004

**Published:** 2026-01-10

**Authors:** Indah Pakpahan, Mentari Sihombing, Haohan Liu, Mengyao Wang, Zheng Su, Mingyan Fang

**Affiliations:** School of Bioengineering, Dalian University of Technology, No. 2 Linggong Road, Ganjingzi District, Dalian, Liaoning Province 116024, China; BGI Research, No. 59 Keji 3rd Road, East Lake High-Tech Development Zone, Wuhan, Hubei Province 430074, China; Department of Bioprocess Engineering, Faculty of Biotechnology, Institut Teknologi Del, Jalan Sisingamangaraja, Sitoluama District, Laguboti, Toba Samosir Regency, North Sumatra 22381, Indonesia; School of Bioengineering, Dalian University of Technology, No. 2 Linggong Road, Ganjingzi District, Dalian, Liaoning Province 116024, China; BGI Research, No. 59 Keji 3rd Road, East Lake High-Tech Development Zone, Wuhan, Hubei Province 430074, China; Department of Software Engineering, Faculty of Vocational Studies, Institut Teknologi Del, Jalan Sisingamangaraja, Sitoluama District, Laguboti, Toba Samosir Regency, North Sumatra 22381, Indonesia; BGI Research, No. 59 Keji 3rd Road, East Lake High-Tech Development Zone, Wuhan, Hubei Province 430074, China; BGI Research, No. 59 Keji 3rd Road, East Lake High-Tech Development Zone, Wuhan, Hubei Province 430074, China; School of Biotechnology and Biomolecular Sciences, Faculty of Science, The University of New South Wales, High Street, Kensington, Sydney, NSW 2052, Australia; State Key Laboratory of Genome and Multi-omics Technologies, BGI Research, No. 11 Beishan Industrial Zone, Yantian District, Shenzhen 518083, China

**Keywords:** variant impact predictors (VIPs), artificial intelligence (AI), variant databases, multi-omics integration, variants of uncertain significance (VUS)

## Abstract

Accurate genetic variant interpretation is crucial for disease research and the development of targeted therapies. Artificial intelligence is transforming this field by integrating computational methodologies across structural biology, evolutionary analysis, and multimodal genomic data. This review examines the evolution from traditional rule-based systems and statistical models to contemporary machine learning, deep learning, and protein language models, while addressing critical challenges in variant classification. Key obstacles include data heterogeneity, interpretability, and the persistence of variants of uncertain significance, emphasizing the critical need for explainable artificial intelligence frameworks and more inclusive genomic databases to improve predictive accuracy across diverse populations. Based on the assessment of current variant impact predictors, we propose strategies for enhanced predictor selection, effective multi-omics data integration, and optimized computational workflows. These recommendations aim to enhance variant interpretation accuracy in both research settings and clinical practice, ultimately contributing to advances in personalized medicine.

## Background

High-throughput sequencing and the Human Genome Project have enabled comprehensive catalogs of human genetic variation, yet distinguishing pathogenic from benign variants remains a central bottleneck in research and clinical genetics [1–3]. Over the last decade, variant impact predictors (VIPs) have progressed from rule-based heuristics to statistical models, machine learning (ML), deep learning (DL), and, most recently, transformer-based large language models that integrate evolutionary, structural, and multi-omics signals [4, 5].

Despite rapid methodological advances, the field faces a paradox: while computational methods have improved significantly, their clinical utility remains limited. ML and DL approaches have demonstrably improved pathogenicity prediction for coding variants, and sequence-based models have advanced interpretation of splicing and regulatory variants. However, several critical challenges persist. First, a substantial proportion of variants remain classified as variants of uncertain significance (VUS), reducing their diagnostic impact. Second, reference datasets such as Genome Aggregation Database (gnomAD) [6] and ClinVar [7] exhibit ancestry imbalances that affect generalizability across populations. Third, DL and transformer-based models often operate as “black boxes,” complicating their alignment with American College of Medical Genetics and Genomics (ACMG)/Association for Molecular Pathology (AMP) interpretive guidelines. Finally, experimental validation lags behind computational predictions, creating a gap between algorithmic output and clinical translation.

This review addresses these challenges through a comprehensive, method-focused synthesis. We map the technological evolution of VIPs across computational paradigms, characterize the supporting database ecosystem, and outline practical workflows for data preprocessing, model development, and evaluation. Rather than proposing new algorithms, we assess current artificial intelligence (AI) capabilities: identifying where approaches are robust, where caution is warranted, and what translational gaps must be addressed for clinical implementation. We provide actionable recommendations for model selection, ancestry-aware algorithm design, multi-omics integration, and validation frameworks to improve the reliability, transparency, and equity of variant interpretation in diverse populations.

Methodologically, this review adopts a narrative, method-focused search of PubMed, Google Scholar, and Scopus combining terms such as variant pathogenicity, *in silico* prediction, deep learning, transformer/foundation model, splicing prediction, non-coding variants, functional assays, and database/tool names. This primary search was supplemented by manual cross-referencing of seminal reviews and benchmarking repositories to ensure a comprehensive coverage of diverse computational frameworks. We prioritized peer-reviewed method papers with clear training/validation descriptions, widely used tools across coding and non-coding tasks, comparative evaluations, and resources on functional screening, fairness, and explainable AI (XAI).

## Curated database infrastructure supporting variant interpretation

The accurate interpretation of genetic variants relies on a stratified data ecosystem integrating genomic, clinical, and functional evidence, in which each layer offers a distinct analytical perspective while remaining interconnected to enable comprehensive pathogenicity assessment.

Population-scale genomic initiatives provide baseline frequency profiling, establishing context for variant rarity assessment. Resources including the Single Nucleotide Polymorphism Database (dbSNP) [8], the 1000 Genomes Project (1KGP) [9], gnomAD [6], and UK Biobank [10] provide allele frequency distributions and mutational constraint profiles across diverse ancestries, enabling distinction between rare pathogenic variants and common benign polymorphisms, especially in underrepresented populations [11]. Functional Annotation of Variants—Online Resource (FAVOR) [12] complements these resources by integrating functional annotations to assess variants lacking clinical evidence.

Pathogenicity evidence emerges from curated clinical and disease-specific repositories. ClinVar [7], Human Gene Mutation Database (HGMD) [13], Human Variants Database (HuVarBase) [14], and ClinGen [15] aggregate experimentally or clinically validated genotype–phenotype associations, forming an empirical foundation for supervised learning approaches in variant classification.

Standardized disease ontologies help transform clinical observations into computational frameworks. Online Mendelian Inheritance in Man (OMIM) [16], Orphanet [17], and Human Phenotype Ontology (HPO) [18] connect genetic alterations to specific disease mechanisms and biological pathways. Additionally, Gene Ontology (GO) [19] complements phenotype-focused resources by clarifying functional impacts at molecular and cellular levels.

Domain-specific repositories provide further refinement of variant interpretation in specialized contexts. Oncology–focused databases, such as Catalogue of Somatic Mutations in Cancer (COSMIC) [20], Database of Curated Mutations (DoCM) [21], and ONGene [22], aggregate tumor-specific mutations. Gene-centric functional assays (e.g., a curated *BRCA1* functional dataset [23]) provide confidence labels for many clinically relevant genes.

Beyond sequence and phenotype resources, protein architecture databases including Universal Protein Resource (UniProt) [24] and Protein Data Bank (PDB) [25] provide the three-dimensional framework essential for understanding variant consequences at the molecular level, particularly useful for structure–function relationship modeling.

Finally, specialized benchmarking and validation resources, including the Benchmark Database for Variations (VariBench) [26], VariSNP [27], and VarCards2 [28], support model evaluation and comparison of emerging AI predictors by providing standardized test sets and performance metrics.

Across this data ecosystem (Table 1) spanning evolutionary, biochemical, structural, and regulatory domains lies the foundation for advanced AI architectures that can effectively model the complex relationships underlying variant pathogenicity [29]. Access to these resources is governed by institutional guidelines and data use agreements ensuring appropriate use of de-identified data.

**Table 1:** Genomic database overview.

No.	Name	Description	Data types	Cross-link	Entries (as of September 2025)	Last updates	Website	Reference
1	1KGP	Large-scale project to create a comprehensive resource on human genetic variation	Population genomic data	Uses refID from dbSNP; variants included in gnomAD	>88 million variants (84.7 million SNPs, 3.6 million indels, 60,000 SVs)	2024–11-18	https://www.internationalgenome.org/	[9]
2	*BRCA1* Dataset	Focused dataset on SNVs in the *BRCA1* gene	Gene-specific genomic data	Variants cross-validated with ClinVar and HGMD	3,893 SNVs	2018–08-20	https://sge.gs.washington.edu/BRCA1/	[23]
3	ClinGen	Clinical genomics resource defining the clinical relevance of genes and variants	Clinical genomics resource	Provides gene-disease evidence to ClinVar; uses HPO and OMIM	3,256 genes, 11,062 variants	2025–09-18	https://clinicalgenome.org/	[15]
4	ClinVar	Public archive of reports of the relationships among human variations and phenotypes.	Clinical genomic data	Integrates rsID from dbSNP; links to ClinGen, OMIM, HPO	5,640,148 records (3,759,476 unique variants)	2025–08-24	https://www.ncbi.nlm.nih.gov/clinvar/	[7]
5	COSMIC	Catalogue of Somatic Mutations in Cancer	Somatic genomic data	Overlaps with DoCM and ClinVar	25,014,261 variants	2025–05-21	https://cancer.sanger.ac.uk/cosmic	[20]
6	dbSNP	Database of Single Nucleotide Polymorphisms and other variants	Genomic variant registry	Referenced by ClinVar and gnomAD via rsID	1,206,053,617 unique rs	2025–01-15	https://www.ncbi.nlm.nih.gov/snp/	[8]
7	DoCM	Manually curated database of clinically relevant mutations.	Clinically curated genomic data	Overlaps with COSMIC and ClinVar	3,818 variants	2024–10-15	http://www.docm.info/	[21]
8	FAVOR	Aggregated variant and indel functional annotations from multiple databases.	Functional annotation data	Indexed by genomic coordinates and rsID; integrates multi-source functional annotations	8,892,915,237 variants (8,812,917,339 SNVs and 79,997,898 indels)	2025–02-05	https://favor.genohub.org/	[12]
9	gnomAD	Aggregated and harmonized human exome and genome sequencing data	Population genomic data	Uses rsID from dbSNP, referenced in ClinVar	730,947 exomes and 76,215 whole genomes	2024–04-19	https://gnomad.broadinstitute.org/	[6]
10	GO	Structured vocabulary for gene product functions and processes	Ontology	Used in UniProt	39,906 terms, 9.41 million annotations, 1.60 million gene products, 5,497 species	2025–07-22	http://www.geneontology.org/	[19]
11	HGMD	Comprehensive collection of germline mutations in human genes	Clinically curated genomic data	Often cross-validated with ClinVar and OMIM	549,178 mutations	2025–07-07	http://www.hgmd.cf.ac.uk/ac/index.php	[13]
12	HPO	Ontology for describing human phenotypic abnormalities	Phenotype ontology	Used by OMIM and ClinGen; mapped to GO	18,000 terms and >156,000 annotations	2024–04-19	https://hpo.jax.org/	[18]
13	HuVarBase	Annotated human variation database	Curated genomic variant data	Merged from COSMIC, ClinVar, 1000 Genomes	774,863 variants from 18,318 proteins (702,048 disease causing and 72,815 neutral variants)	2018–06-01	https://www.iitm.ac.in/bioinfo/huvarbase	[14]
14	OMIM	Comprehensive catalog of human genes and genetic phenotypes	Gene–phenotype data	Includes HPO annotations; links to ClinVar and UniProt	27,938 entries (26,394 autosomal, 1,407 X linked, 64 Y linked, 73 mitochondrial)	2025–09-17	https://www.omim.org/	[16]
15	ONGene	Curated database of human oncogenes	Cancer gene annotation data	–	803 oncogenes (698 protein-coding genes + 105 non-coding)	2016–12-26	https://ongene.bioinfo-minzhao.org/	[22]
16	Orphanet	Database for rare diseases and orphan drugs	Rare disease ontology	Shares HPO and OMIM terms	9,785 clinical entities, 6,528 rare disorders, 8,296 disease gene relationships, 115,611 phenotypic annotations, 16,418 epidemiological data, 689 orphan drugs 8,648, expert centers	2025–06-24	https://www.orpha.net/	[17]
17	PDB	Repository for 3D structural data of biological macromolecules	Protein structural data	Linked from UniProt entries	242,296 structures, 1,068,577 computed structure models	2025–09-17	https://www.rcsb.org/	[25]
18	UK Biobank	Large-scale biomedical database with genetic and health information	Population-scale genotype–phenotype data	Overlaps with dbSNP	90 million variants	2024–08-13	https://www.ukbiobank.ac.uk/	[10]
19	UniProt/UniProtKB	Comprehensive protein sequence and annotation resource	Protein sequence and functional annotation data	Cross-links to PDB, GO, dbSNP, ClinGen, Orphanet,	253,206,171 entries (UniProtKB/Swiss-Prot: 572,970 entries and UniProtKB/TrEMBL: 252,633,201 entries)	2025–06-17	https://www.uniprot.org/	[24]
20	VarCards2	Updated version of VarCards, human variant annotation and interpretation	Variant annotation and interpretation data	Some extracted from gnomAD, ClinVar, COSMIC, dbSNP	368,820,266 indels, 2,773,555 CNVs	2023–10-21	http://www.genemed.tech/varcards2/	[28]
21	VariBench	Benchmark database for variation datasets in bioinformatics	Benchmark variant datasets	Some extracted from dbSNP, OMIM	>90 million variants	2023–05-12	https://structure.bmc.lu.se/VariBench/	[26]
22	VariSNP	A benchmark database suite comprising variation datasets that can be used for developing and testing the performance of variant effect prediction	Benchmark variant data	Selected from dbSNP	30,571,777 variants	2017–02-16	https://structure.bmc.lu.se/VariSNP/	[27]

## The technological trajectory of variant pathogenicity assessment

**Figure 1:**
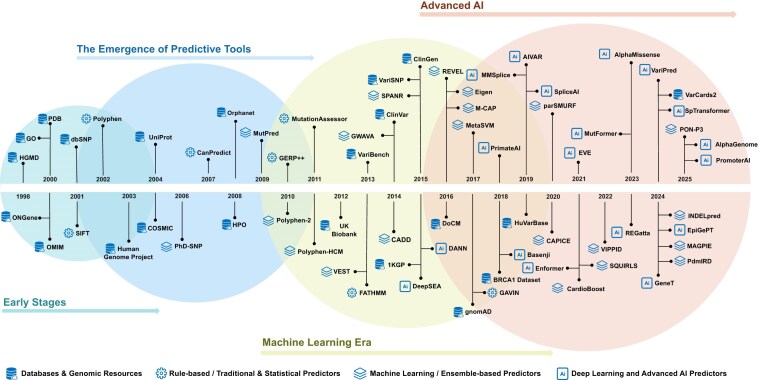
Evolution of *in silico* tools for predicting genetic variant pathogenicity. This figure illustrates the temporal progression of computational approaches for genetic variant pathogenicity prediction. The evolutionary trajectory is categorized into four phases: (1) early stages, characterized by rudimentary rule-based algorithms; (2) the emergence of predictive tools, marking the transition to more sophisticated statistical frameworks; (3) machine learning era, defined by the integration of supervised and unsupervised learning methodologies; and (4) advanced AI, representing contemporary approaches that leverage deep learning architectures and multi-modal data integration.

The development of VIPs has evolved through five distinct yet overlapping paradigms, each addressing limitations of previous approaches while expanding analytical capabilities (Fig. 1). This progression mirrors broader technological trends within computational biology, and has evolved from initial rule-based heuristics toward advanced transformer architectures. Each step has facilitated progressively refined insights into genomic variation (Supplementary Table 1).

### Phase 1: Rule-based biological heuristics

Early predictive tools emerged from foundational biological insights. Pioneering rule-based predictors such as Sorting Intolerant From Tolerant (SIFT) [30] and Polymorphism Phenotyping (PolyPhen) [31] relied on empirical knowledge and evolutionary principles to evaluate variant pathogenicity. These tools primarily relied on sequence conservation and amino acid physicochemical properties to distinguish between benign and deleterious variants. Gene-Aware Variant INterpretation (GAVIN) [32] applied predefined classification logic to refine pathogenicity predictions within gene-specific contexts. Their strength lay in their high interpretability, as the underlying biological rules were explicit and transparent.

However, this rule-based approach inherently limited their scalability and ability to capture the complex, nonlinear patterns emerging from rapidly expanding genomic datasets. Though computationally efficient, these methods typically operated within limited genomic contexts and largely overlooked non-coding or regulatory regions such as promoters or splice sites [33]. Despite these constraints, early-phase predictors remain valuable for preliminary assessments and continue to be incorporated into comprehensive prediction frameworks [34], particularly where interpretability is prioritized over prediction complexity.

### Phase 2: Statistical modeling and probabilistic frameworks

As genomic databases expanded in both size and diversity, statistical methods emerged to enhance the prediction accuracy through probabilistic modeling. Tools such as MutationAssessor [35] utilized evolutionary conservation patterns within protein families, while Functional Analysis Through Hidden Markov Models (FATHMM) [36] integrated evolutionary conservation scores into sequence-based probabilistic models to estimate variant pathogenicity. Eigen [37], an unsupervised spectral method, prioritized variants by analyzing annotation correlations and constructing a weighted score across both coding and non-coding genomic regions. In parallel, Genomic Evolutionary Rate Profiling++ (GERP++) [38] quantifies evolutionary constraint using a maximum likelihood model to calculate rejected substitutions, and is widely used as an annotation feature in downstream predictive frameworks.

While these tools have advanced the ability to contextualize genomic variation, predictive methods still face substantial challenges when analyzing rare or novel variants due to insufficient representation in existing reference datasets. Additionally, their heavy reliance on high-quality reference annotations limited their effectiveness in classifying clinically important yet poorly characterized VUS [39], prompting further developments toward data-driven ML methods.

### Phase 3: Traditional machine learning and ensemble approaches

ML algorithms transformed variant prediction by capturing complex, nonlinear patterns in multidimensional genomic datasets, enabling integration of diverse biological features [40]. Approaches in this era can be broadly categorized into:

*Classical machine learning classifiers:* Naive Bayes classifiers were applied in tools like PolyPhen-2 [41], which integrates sequence and structure-based features to predict the effects of amino acid substitutions. This algorithm was also effectively deployed in disease-specific contexts, such as Polymorphism Phenotyping for Hypertrophic Cardiomyopathy (PolyPhen-HCM) [42], CanPredict [43], and Splicing-based Analysis of Variants (SPANR) [44]. Support vector machines (SVMs) and random forests (RFs) underpin tools such as Combined Annotation Dependent Depletion (CADD) [33], Predictor of human Deleterious Single Nucleotide Polymorphisms (PhD-SNP) [45], Variant Effect Scoring Tool (VEST) [46], Meta-analytic Support Vector Machine (MetaSVM) [47], MutPred [48], incorporating diverse features such as evolutionary conservation, protein structure, and gene-level annotations within unified predictive frameworks. SQUIRLS [49] focuses on splice-altering variants, while genome-wide annotation of variants (GWAVA) [50] extends this approach to non-coding regions, prioritizing functional regulatory variants across diverse biological contexts.

The RF algorithm was similarly adapted for specialized tasks, powering tools like the Variant Impact Predictor for Primary Immunodeficiency Diseases (VIPPID) [51] for immunodeficiencies and Prediction of Deleterious Missense Mutation for Inherited Retinal Diseases (PdmIRD) [52] for retinal diseases. For instance, SVMs identify optimal hyperplanes to separate classes, while RFs build multiple decision trees and aggregate their results, offering robustness and handling high-dimensional data effectively.

*Gradient boosting approaches:* More recent implementations employ gradient boosting machines (GBMs) to enhance classification performance. Multimodal Annotation Generated Pathogenic Impact Evaluator (MAGPIE) [5], Consequence-Agnostic Pathogenicity Interpretation of Clinical Exome variations (CAPICE) [53], INDELpred [54], Mendelian Clinically Applicable Pathogenicity (M-CAP) [55], and PON-P3 [56] exemplify this approach by integrating multi-source annotations via gradient-boosted decision trees. The strength of GBMs for leveraging complex feature sets also made them ideal for building specialized predictors like CardioBoost [57] for cardiac genetics. GBMs operate on the principle of iterative improvement: they sequentially build models, with each new model attempting to correct the errors of its predecessor.

While these models demonstrate superior accuracy, they require high-quality, well-curated training datasets; when data are limited, sparse, or imbalanced, challenges such as overfitting and limited generalizability can compromise their clinical utility [58].

*Ensemble prediction systems:* To address the inherent limitations of individual algorithms, ensemble methodologies aggregate outputs from multiple predictors, thereby enhancing robustness and reliability [59]. Tools such as the Rare Exome Variant Ensemble Learner (REVEL) [60] and Parallel SMote Undersampled Random Forest (parSMURF) [61] exemplify this strategy by combining complementary models to mitigate individual weaknesses while amplifying collective strengths. Although ensemble methods introduce additional computational costs and diminish transparency, these frameworks have become integral to clinical genetics pipelines where reliability is paramount. Despite improved accuracy, ML-based methods remain sensitive to training data and often lack interpretability, limiting their use as standalone clinical tools.

### Phase 4: Deep learning approaches

DL has further advanced variant interpretation by exploiting neural networks to model nonlinear relationships in large, multidimensional genomic datasets [62]. DL models can learn hierarchical feature representations directly from raw data, reducing the reliance on manual feature engineering. Deleterious annotation of genetic variants using neural networks (DANN) [63], employs a deep neural network trained on the same functional annotations as CADD to score both coding and non-coding variants. PrimateAI [64] leverages evolutionary signatures across primates to enhance prediction accuracy. This capacity for learning from raw sequence data has improved the prediction of non-coding and regulatory variants. Models like DeepSEA [65] and Basenji [66] demonstrated that chromatin accessibility and gene expression could be predicted directly from DNA sequence, capturing both proximal and distal effects. PromoterAI [67] focused on predicting the impact of promoter variants, further refining DL-based modeling of regulatory sequences.

In splicing prediction, DL brought a significant leap in accuracy. SpliceAI [68] utilized convolutional neural networks to capture long-range dependencies in pre-mRNA sequences, often outperforming previous models, though at the cost of interpretability. MMSplice [69] attempted to address this interpretability gap through more modular or explainable designs. While these approaches demonstrate substantial sensitivity, challenges persist regarding their “black box” nature and intensive computational requirements [70].

### Phase 5: Transformer architectures

The most recent technological leap has been catalyzed by adapting transformer architectures and language modeling principles to genomic and protein sequences. Originally developed for natural language processing, transformer models have become powerful tools for predicting functional impacts of genetic variants [71]. The core innovation lies in the self-attention mechanism, which allows the model to weigh the importance of different parts of the input sequence when processing each element [72]. This enables the capture of long-range dependencies and global context within biological sequences, a key aspect for understanding how mutations in one part of a protein can affect distant functional regions [73]. The application of transformers has evolved into two powerful, complementary paradigms:

*Protein language models (pLMs):* Pre-trained on vast corpora of evolutionary sequences, pLMs learn fundamental principles of protein structure and function. Models like ESM-1b [74] excel at capturing subtle sequence constraints, distinguishing isoform-specific pathogenic variants with high accuracy (area under the receiver operating characteristic curve [ROC-AUC]: 0.905 on ClinVar, 0.897 on HGMD/gnomAD). This approach is exemplified by tools like Variant impact Predictor (VariPred) [75], which leverages these learned representations to predict variant effects, often outperforming traditional structure-dependent methods. The strength of pLMs is further demonstrated by their versatility in tasks such as masked residue prediction (82% accuracy) [76] and sequence conservation analysis (Matthews Correlation Coefficient (MCC) = 0.596) [77].
*Regulatory genome transformers:* These models are designed to interpret the non-coding genome by learning the regulatory code directly from DNA sequence. Enformer [78] set a new standard by using a transformer architecture to achieve leading performance in predicting chromatin accessibility and gene expression profiles from sequence context, capturing effects of distal enhancers. This paradigm is extended by tools like AlphaGenome [79], which integrates >100 regulatory features in a multitask framework. EpiGePT [80] focuses on precise prediction of context-specific epigenomic signals, achieving high performance (Pearson *r* = 0.710; auROC = 0.949).

The potential of this era is revealed in models that fuse these approaches or leverage their insights for specific clinical tasks. AlphaMissense [71] by DeepMind represents a seminal work, fusing structural insights from AlphaFold [81] with the pattern recognition of language models to generate a massive, highly accurate map of missense variant pathogenicity. Similarly, MutFormer [82] integrates self-attention with convolutional layers for missense analysis, and Genetic Transformer (GeneT) [83] achieves high recall rates (99% in synthetic data, 97.85% in clinical cohorts) for identifying causative variants. This fusion also enables more specialized applications, such as SpTransformer [84], which incorporates tissue specificity into splicing prediction.

Together, these findings suggest that transformer architectures can achieve improved performance across diverse variant interpretation tasks, including coding, non-coding, and splicing variants. In benchmark comparisons, these models often outperform earlier DL approaches, though performance gains vary considerably with task definition, training data, and evaluation frameworks. These advances reflect a transition from feature-engineered methods to models that learn context-aware representations directly from biological sequences, potentially improving predictive capacity while introducing challenges in scalability and interpretability. Representative algorithms and VIPs across these technological phases are summarized in Table 2, with detailed tool characteristics in Supplementary Table 1.

**Table 2:** Computational algorithms used for variant impact predictors.

No.	Algorithm	Description	VIPs	References
1	Naive Bayes	A probabilistic graphical model that represents a set of variables and their conditional dependencies	PolyPhen-2, PolyPhen-HCM, CanPredict, SPANR	[41–44]
2	Support vector machines	A supervised learning model that analyzes data for classification and regression analysis	CADD, PhD-SNP, MetaSVM	[33, 45, 47]
3	Random forest	An ensemble learning method that operates by constructing multiple decision trees	VEST, MutPred, SQUIRLS, GWAVA, VIPPID, PdmIRD, REVEL, parSMURF	[46, 48–52, 60, 61]
4	Gradient boosting machines	An ML technique for regression and classification problems that builds a model in a stage-wise fashion.	MAGPIE, CAPICE, INDELpred, M-CAP, PON-P3, CardioBoost	[5, 53–57]
5	Neural networks	A set of algorithms modeled after the human brain, designed to recognize patterns	DANN, PrimateAI, DeepSEA, Basenji, PromoterAI, SpliceAI, MMSplice, AIVAR	[63–69, 85]
6	Transformer	A DL model that uses self-attention mechanisms to process sequential data, capturing long-range dependencies and relationships in the data.	AlphaMissense, VariPred, Enformer, AlphaGenome, EpiGePT, MutFormer, GeneT, SpTransformer	[71, 75, 78–80, 82–84]

To provide illustrative quantitative context, Table 3 presents performance metrics for representative VIPs spanning major methodological phases, as reported in original publications. These values derive from heterogeneous evaluation settings and are not directly comparable.

**Table 3:** Representative variant impact predictors and their reported performance.

No.	Methodological paradigm	VIPs	Variant type(s)	Reported performance (as published)	References
1	Rule-based	SIFT	Missense (amino acid-altering) variants	ROC-AUC: 0.80–0.82	[30]
		PolyPhen	Missense (amino acid-altering) variants	ROC-AUC: ≈0.83	[31]
2	Statistical	MutationAssessor	Missense (amino acid-altering) variants	ROC-AUC: ≈0.86	[35]
		FATHMM	Coding and non-coding variants	Accuracy: ≈0.86	[36]
3	Machine learning	CADD	Coding and non-coding variants	ROC-AUC: 0.90–0.93	[33]
		REVEL	Missense (amino acid-altering) variants	ROC-AUC: 0.90–0.91	[60]
4	Deep learning	SpliceAI	Splicing-altering variants	PR-AUC: ≈0.90	[68]
		DeepSEA	Non-coding regulatory variants	ROC-AUC: ≈0.96	[65]
5	Transformer/pLM	AlphaMissense	Missense (amino acid-altering) variants	ROC-AUC: ≈0.94	[71]
		MutFormer	Missense (amino acid-altering) variants	ROC-AUC: 0.92–0.97	[82]

## Integrative tools for variant annotation

Comprehensive annotation pipelines consolidate outputs from diverse databases and predictive algorithms to streamline variant interpretation. Ensembl Variant Effect Predictor (VEP) [86] annotates variants with gene-level and regulatory features, supporting flexible plug-in integration of tools such as SIFT, CADD, and SpliceAI. ANNOVAR [87] enables gene-based, region-based, and filter-based annotation in unified workflows, incorporating population frequency data (e.g., gnomAD) and multiple pathogenicity scores.

Web-based platforms further enhance usability and evidence integration. VarSome [88] and MobiDetails [89] aggregate clinical annotations, *in silico* predictions, and allele frequencies via interactive interfaces apply ACMG-based classification framework. InterVar [90] emphasizes rule-based implementation of ACMG guidelines, offering reproducible, guideline-concordant classification support. Exomiser [91] combines variant pathogenicity scores with phenotype data (HPO terms) to prioritize candidate variants, especially in rare disease diagnosis.

These integrative tools reduce the burden of manual curation by consolidating diverse resources into unified workflows, enabling efficient filtering and evidence synthesis for both research and clinical applications. However, while these pipelines effectively consolidate evidence from multiple sources, the complexity of variant interpretation requires a systematic approach to optimize their implementation. The selection of appropriate predictors and the integration of their outputs into coherent clinical decisions necessitates a structured workflow framework.

## Variant interpretation workflow and evaluation considerations

**Figure 2:**
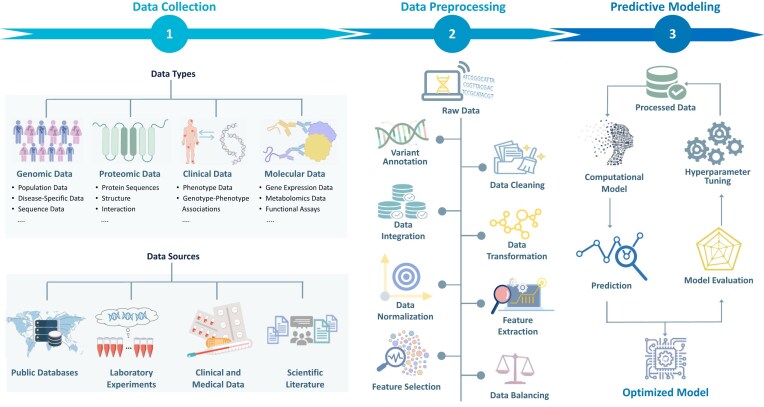
Integrated pipeline for variant pathogenicity prediction. This figure delineates a systematic framework for developing variant pathogenicity prediction models. The pipeline harnesses multi-modal data inputs (genomic, proteomic, molecular, and clinical) acquired from public repositories, experimental assays, clinical documentation, and literature curation. Critical preprocessing steps include variant annotation, normalization, cross-platform integration, and feature engineering to enhance signal integrity. The analytical workflow culminates in a machine learning implementation with rigorous hyperparameter optimization and comprehensive performance assessment to ensure robust predictive capacity across diverse genetic contexts.

To address these challenges systematically, the variant interpretation process can be conceptualized as a structured pipeline that links raw genomic data to actionable clinical predictions (Fig. 2). This systematic approach provides a comprehensive framework for improved variant classification accuracy and supports the development of more equitable genomic medicine applications.

The framework encompasses three interconnected phases that collectively transform raw genomic data into clinically actionable insights. The initial phase centers on comprehensive data acquisition, integrating population-level variant frequencies, disease-specific variants repositories, high-throughput molecular characterization profiles, and curated clinical annotations. This foundation transitions into an advanced preprocessing phase by systematically integrating heterogeneous data sources, including evolutionary conservation, protein structural, and regulatory element characterizations. Subsequent steps include normalization, feature extraction, and dimensionality reduction to optimize the computational feature space for downstream analytical applications [92].

The analytical phase leverages sophisticated predictive modeling approaches, employing rigorous hyperparameter optimization strategies to achieve optimal discriminative performance [93]. This computational framework generates probabilistic assessments that require systematic validation through robust benchmarking protocols. Evaluation encompasses multiple complementary metrics: sensitivity and specificity for detection of pathogenic versus benign variants, precision metrics that characterize predictive accuracy, and ROC-AUC, which provides threshold-independent assessment of discriminative performance [94]. Comparative analyses against benchmark datasets, such as ClinVar or consortium-established validation cohorts, enable cross-method evaluations while ensuring generalizability through cross-validation [95].

The optimization of these computational models requires adherence to stringent development protocols that emphasize data integrity through integration of high-quality variant annotations from diverse repositories such as gnomAD and ClinVar. Advanced feature engineering requires integration of multi-omics data layers spanning genetic, proteomic, regulatory, and clinical domains [96]. Robust model development paradigms demand thorough cross-validation, systematic hyperparameter tuning, and validation against independent external datasets to ensure reproducibility and clinical applicability [97].

## Challenges limiting clinical translation

Despite these methodological advances across both general and specialized AI approaches, several interconnected challenges persist in limiting the clinical deployment of VIPs. These challenges span data limitations, dataset biases, polygenic and complex traits, and model interpretability, each of which would benefit from targeted solutions to bridge the gap between computational prediction and clinical application.

### Data limitations and functional validation bottlenecks

VUS continue to represent a large fraction of clinical findings, particularly for rare and novel or non-coding variants [98]. AI-based models address this challenge by leveraging evolutionary conservation, regulatory annotations, and protein structural features to infer potential pathogenicity and prioritize variants lacking database evidence or functional validation, although significant VUS challenges remain. While computational methods such as matrix factorization [99] and active learning [100] provide incremental gains, they often remain dependent on sparse or biased training data.

Recent years have seen growing reliance on high-throughput functional assays such as CRISPR-Cas9 screens and multiplexed assays of variant effects (MAVEs), which can directly measure variant function at scale [101]. These approaches are beginning to be incorporated into clinical frameworks, e.g., MAVE-derived scores have been incorporated into ClinGen rules for *BRCA1, TP53*, and *PTEN* [102]. Yet, widespread clinical use remains limited by assay cost, turnaround time, and availability outside specialized centers.

### Dataset biases and generalizability issues

Beyond general data limitations, demographic imbalances in genomic databases primarily skewed toward individuals of European ancestry constrain model performance across diverse populations [103]. Such sampling biases may reduce the reliability of variant interpretation in underrepresented groups [104]. Training datasets for AI models are typically de-identified and accessed through controlled mechanisms governed by data use agreements and ethical guidelines. While targeted sequencing and federated learning are emerging, coverage remains incomplete for many populations, limiting equitable variant interpretation [105].

### Modeling polygenic and complex traits

Current single-variant VIPs like AlphaMissense are optimized for high-penetrance Mendelian variants, which differs substantially from diverges from the genetic architecture of complex, polygenic diseases. While these models excel at identifying variants disrupting protein stability or evolutionary conservation, they struggle to capture the cumulative contribution of numerous small-effect variants underlying conditions such as hypertension, hyperlipidemia, and type 2 diabetes [106].

Furthermore, current predictors operate largely in isolation, neglecting the “omnigenic” reality in which disease susceptibility is influenced by epistatic interactions and regulatory networks rather than single-locus disruptions. Consequently, binary classification of variants as “pathogenic” or “benign” lacks the granularity required for complex trait prediction, where risk is continuous and context-dependent [107].

### Model interpretability versus predictive power

Even when data issues are mitigated, the interpretability of advanced AI models remains an important barrier to clinical adoption. ML models like evolutionary model of variant effect (EVE) [108] have demonstrated high performance in variant prediction but are often too complex to explain and their opacity poses challenges for clinical trust, regulatory approval, and usability [109, 110]. Recent efforts in XAI strategies [111] and interpretable classifiers like Artificial Intelligent Variant Classifier (AIVAR) [85] seek to bridge this gap by providing transparent and biologically meaningful rationales for prediction. XAI frameworks help distinguish robust predictions from algorithmic artifacts, but AI outputs are best interpreted as probabilistic prioritization requiring validation with clinical and experimental evidence.

### Clinical role in AI-based variant interpretation

AI-based VIPs are designed to support clinical decision-making within established diagnostic workflows rather than operate as standalone classifiers. Empirical evaluations show VIPs function most effectively when augmenting expert review, with clinicians retaining final classification decisions, particularly for VUS [112, 113].

In practice, clinicians integrate AI predictions with phenotypic information, family history, segregation validation, and functional evidence to guide variant classification. This workflow underscores that successful clinical adoption is strongly influenced by how AI outputs are presented: interpretable scores, confidence estimates, and evidence aligned with ACMG/AMP guidelines are essential. Ultimately, clinical utility depends as much on usability, explainability, and seamless workflow integration as on algorithmic performance.

## Toward more interpretable and clinically actionable predictions

Emerging trends ranging from multi–omics fusion to foundation models have the potential to advance variant interpretation along five strategic axes. These axes collectively span molecular, computational, functional, and population-level dimensions of clinical genomics.

### Phenotype-conditioned variant interpretation

Phenotype-conditioned approaches link genetic alterations directly to clinical manifestations, addressing allelic heterogeneity. Multi-task architectures such as the Variant-to-Phenotype framework [114] integrate protein interactomes, structural descriptors, and evolutionary constraints to predict pathogenicity conditioned on HPO categories. By capturing genotype–phenotype associations, these approaches accelerate rare disease diagnosis and identify therapeutic targets, enhancing variant scoring into a clinically useful tool that can inform patient-specific pathology.

### Multi-omics evidence integration

Integrating genomic, transcriptomic, proteomic, and epigenetic data [115] improves statistical power for associations with low-frequency variants [116]. Effective integration typically involves selectively, prioritizing omics layers most informative for specific variant types [117, 118]. Transcriptomic and splicing data are critical for interpreting non-coding and splice-site variants, particularly for resolving VUS [119, 120], whereas proteomic and structural information is more informative for missense variants [121]. Tissue-specific expression needs to be considered, as variant effects can be context-dependent.

AI-based approaches integrate multi-omics through feature-level aggregation, model-level fusion, or post hoc synthesis according to ACMG/AMP guidelines. Despite their potential, these approaches remain constrained by data heterogeneity, tissue specificity, and incomplete population coverage. Selective prioritization of high-value omics layers and shared databases offer a promising strategy for scalable clinical adoption.

### Foundation models and few–shot adaptation

Transformer-based language models enable fine-tuning with minimal labeled data, addressing challenges posed by rare variants. Pre-trained on large, unlabeled datasets, these models can be efficiently adapted to novel variant types using parameter-efficient fine-tuning [122] and prompt engineering [123], potentially making high-performance variant prediction accessible for understudied conditions. Recent benchmarking of DNA foundation models demonstrates their capacity for zero-shot variant effect prediction, with large, multi-species architectures demonstrating strong discriminative power in capturing both local and extended contextual effects [124].

**Figure 3:**
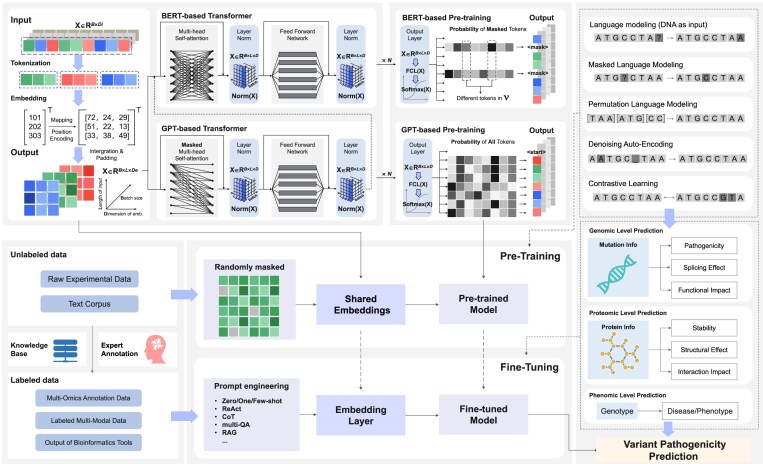
Transformer-based framework for variant pathogenicity prediction. This figure illustrates a transformer-based pipeline for predicting genetic variant pathogenicity. Input sequences (*X*∈*R*^*B*×*L*×*D*^, where *X* represents the input tensor, *R* denotes real numbers, *B* = batch size, *L* = sequence length, and *D* = embedding dimension) are processed through three key stages: (1) tokenization and embedding, (2) contextual encoding via multi-head self-attention, and (3) classification. The model undergoes pre-training on multi-omics datasets followed by pathogenicity-specific fine-tuning. Prompt engineering facilitates performance with limited labeled data. Other key components include LayerNorm to mitigate vanishing or exploding gradients, fully connected layers for effective feature extraction, and Softmax activation for generating robust probability distributions. Together, the overall architecture and training strategies enable the model to leverage latent biological mechanisms for accurate variant classification.

These transformer-based approaches can be conceptually unified through a shared pipeline that transforms biological sequences into clinical predictions (Fig. 3). Biological sequences undergo tokenization and embedding generation, converting raw genomic data into computational representations. Contextual encoding via multi-head self-attention processes these embeddings, where unsupervised pre-training objectives, including masked language modeling, permutation language modeling, and contrastive learning [125], enable the models to capture meaningful sequence patterns without labeled data. Task-specific adaptation occurs through specialized prediction heads (task-specific output layers) coupled with either frozen or fine-tuned backbones. Prompt-engineering strategies enable efficient knowledge transfer when limited labeled datasets are limited, and predictions can be further interpreted or calibrated using functional validation and systems-level analyses to support clinical interpretation.

### Scalable functional validation and systems-level context

Scaling functional validation beyond specialized centers is increasingly feasible through collaborative resources. Resources such as the Atlas of Variant Effects [126] and MaveDB [127] provide standardized repositories of multiplexed assay data directly incorporated into ACMG/AMP classification frameworks. At the same time, integration of functional readouts with systems biology analyses, including pathway and interaction networks, may help contextualize variant effects at multiple biological levels [128]. Functional evidence is being more systematically integrated into precision medicine workflows, rather than an ad hoc supplement, and pairing these insights with diverse population data supports robust cumulative-risk interpretation while accounting for population-specific variation.

### Equity-aware modeling across variant and polygenic risk

Systematically diversifying genomic databases addresses biases from population underrepresentation [103]. Collaborative initiatives like the Human Heredity and Health in Africa consortium [129] provide models for ethical, scientifically robust data collection among underserved populations. Ethical AI practices, such as fairness assessments, transparent model documentation, and inclusive stakeholder engagement, help mitigate algorithmic biases.

### Algorithmic strategies for polygenic risk score transferability

Equity challenges become particularly pronounced when extending variant-level prediction frameworks to polygenic risk modeling. While generating diverse population-scale data is essential for addressing these challenges, several computational strategies address ancestry bias using existing resources. Transfer learning adjusts models for population-specific linkage disequilibrium and variants shift using existing summary statistics [130, 131], while domain adaptation constructs ancestry-invariant representations through adversarial learning [132, 133]. Data augmentation via generative models supplements scarce data with synthetic genomes [134, 135].

For polygenic risk prediction, ensemble methods integrate multiple polygenic risk score construction strategies: supervised approaches like CT-SLEB [136] and multi-ancestry polygenic risk scores based on ensemble of penalized regression models [137] combine clumping-thresholding, empirical Bayes, and penalized regression using target population genome-wide association study data, while unsupervised frameworks such as Unsupervised Ensembles [138] aggregate pre-trained models based on prediction concordance without requiring phenotype data from target populations, which enables robust performance even in underrepresented populations by circumventing sparse phenotype availability.

### Transitioning to polygenic modeling

For complex diseases, single-variant predictions may have limited utility. Emerging frameworks are moving toward systems-level polygenic modeling that integrates cumulative genetic architecture. Ensemble methods like polygenic risk predictions integrating common and rare variants [139] combine polygenic background with rare variant burden, enhancing predictive accuracy across ancestries. Graph neural networks like PRS-Net [140] model genes and pathways as networks, capturing epistatic dependencies [141] and potentially enhancing both interpretability and clinical actionability. Benchmark studies have shown that combining rare and common variant modeling improves cross-ancestry predictive performance, providing stronger clinical evidence for polygenic risk application. Realizing these advances will require continued efforts in both algorithmic innovation and data diversification to promote equitable benefits across populations.

## Implications and limitations

Recent advances in AI-based variant prediction have shown substantial progress. DL approaches, particularly transformer architectures and pLMs, have improved sequence context capture and biological signal integration across diverse variant interpretation tasks.

However, important limitations constrain clinical translation of these findings. Reported performance gains are context-dependent, derived from specific benchmarks and curated datasets, and do not establish universal superiority of any single method. Differences in data composition, labeling practices, and evaluation protocols limit direct cross-method comparisons. Despite improved predictive accuracy, many models require further validation or interpretability improvements for standalone clinical use, and challenges including ancestry bias and limited experimental validation persist.

These limitations underscore that AI-based VIPs function primarily as decision-support tools that complement expert judgment within established clinical workflows rather than as definitive classifiers. Continued progress requires standardized benchmarking across diverse populations, transparent reporting of model scope and limitations, development of interpretable architectures, and systematic experimental validation of computational predictions.

## Conclusions

In summary, the integration of diverse datasets, transparent predictive algorithms, and consistent validation practices will be critical for advancing AI-driven variant interpretation into routine clinical use. The rapid advancement of computational tools has significantly enhanced our ability to predict genetic variant pathogenicity, offering scalability and accuracy. However, several challenges remain, particularly around accurately classifying VUS, and ensuring fairness and transparency of predictive models. Continued improvement will require effectively integrating multi-omics information, expanding international cooperation to diversify genomic datasets, and systematically linking computational predictions to robust experimental validations. With these concerted efforts, next-generation computational tools can help realize the promise of personalized medicine, guiding clinicians and researchers toward deeper mechanistic insights and improved patient care.

## Supplementary Material

giag004_Supplemental_File

giag004_Authors_Response_To_Reviewer_Comments_original_submission

giag004_GIGA-D-25-00463_original_submission

giag004_GIGA-D-25-00463_Revision_1

giag004_Reviewer_1_Report_original_submission Ammar Husami -- 11/12/2025

giag004_Reviewer_1_Report_Revision_1 Ammar Husami -- 1/5/2026

giag004_Reviewer_2_Report_original_submission Weiyang Li -- 12/8/2025

giag004_Reviewer_2_Report_Revision_1 Weiyang Li -- 1/6/2026

giag004_Reviewer_3_Report_original_submission Jinming Han -- 12/17/2025

giag004_Reviewer_3_Report_Revision_1 Jinming Han -- 1/5/2026

## Data Availability

Not applicable.

## Additional file

**Supplementary Table S1:** Existing *in silico* predictor for genetic variant pathogenicity.

## Abbreviations

1KGP: 1000 Genomes Project; ACMG: American College of Medical Genetics and Genomics; AI: artificial intelligence; AIVAR: Artificial Intelligent Variant Classifier; AMP: Association for Molecular Pathology; AUC: area under the curve; CADD: Combined Annotation Dependent Depletion; CAPICE: Consequence-Agnostic Pathogenicity Interpretation of Clinical Exome variations; COSMIC: Catalogue of Somatic Mutations in Cancer; DANN: deleterious annotation of genetic variants using neural networks; dbSNP: Single Nucleotide Polymorphism Database; DL: deep learning; DoCM: Database of Curated Mutations; EVE: evolutionary model of variant effect; FATHMM: Functional Analysis Through Hidden Markov Models; GAVIN: Gene-Aware Variant INterpretation; GBM: gradient boosting machine; GeneT: Genetic Transformer; GERP++: Genomic Evolutionary Rate Profiling++; gnomAD: Genome Aggregation Database; GO: Gene Ontology; GWAVA: genome-wide annotation of variants; HGMD: Human Gene Mutation Database; HPO: Human Phenotype Ontology; HuVarBase: Human Variants Database; IRDs: Inherited Retinal Diseases; MAGPIE: Multimodal Annotation Generated Pathogenic Impact Evaluator; MAVEs: multiplexed assays of variant effect; MCC: Matthews Correlation Coefficient; M-CAP: Mendelian Clinically Applicable Pathogenicity; MetaSVM: Meta-analytic Support Vector Machine; ML: machine learning; OMIM: Online Mendelian Inheritance in Man; parSMURF: Parallel SMote Undersampled Random Forest; PDB: Protein Data Bank; PdmIRD: Prediction of Deleterious Missense Mutation for IRDs; PhD-SNP: Predictor of human Deleterious Single Nucleotide Polymorphisms; pLMs: protein language models; PolyPhen: Polymorphism Phenotyping; PolyPhen-HCM: Polymorphism Phenotyping for Hypertrophic Cardiomyopathy; REVEL: Rare Exome Variant Ensemble Learner; RF: Random Forest; ROC: receiver operating characteristics; SIFT: Sorting Intolerant From Tolerant; SPANR: Splicing-based Analysis of Variants; SVM: support vector machine; VariBench: Benchmark Database for Variations; VariPred: Variant impact Predictor; VEP: Variant Effect Predictor; VEST: Variant Effect Scoring Tool; VIPPID: Variant Impact Predictor for Primary Immunodeficiency Diseases; VIPs: variant impact predictors; VUS: variants of uncertain significance; XAI: explainable artificial intelligence.

## References

[1] Aworunse OS, Adeniji O, Oyesola OL, et al. Genomic interventions in medicine. Bioinf Biol Insights. 2018;12:1177932218816100. 10.1177/1177932218816100. PMC6287307 30546257

[2] Rego SM, Snyder MP. High throughput sequencing and assessing disease risk. Cold Spring Harb Perspect Med. 2019;9:a026849. 10.1101/cshperspect.a026849. 29959131 PMC6314070

[3] Spielmann M, Kircher M. Computational and experimental methods for classifying variants of unknown clinical significance. Cold Spring Harb Mol Case Stud. 2022;8:a006196. 10.1101/mcs.a006196. 35483875 PMC9059783

[4] Boulaimen Y, Fossi G, Outemzabet L, et al. Integrating large language models for genetic variant classification. arXiv 2024; 10.48550/arXiv.2411.05055.

[5] Liu Y, Zhang T, You N et al. MAGPIE: accurate pathogenic prediction for multiple variant types using machine learning approach. Genome Med. 2024;16:3. 10.1186/s13073-023-01274-4. 38185709 PMC10773112

[6] Karczewski KJ, Weisburd B, Thomas B et al. The ExAC browser: displaying reference data information from over 60 000 exomes. Nucleic Acids Res. 2017;45:D840–45. 10.1093/nar/gkw971. 27899611 PMC5210650

[7] Landrum MJ, Lee JM, Riley GR, et al. ClinVar: public archive of relationships among sequence variation and human phenotype. Nucleic Acids Res. 2014;42:D980–85. 10.1093/nar/gkt1113. 24234437 PMC3965032

[8] Sherry ST, Ward M-H, Kholodov M et al. dbSNP: the NCBI database of genetic variation. Nucleic Acids Res. 2001;29:308–11. 10.1093/nar/29.1.308. 11125122 PMC29783

[9] 1000 Genomes Project Consortium; Auton A, Brooks LD, Durbin RM, et al. A global reference for human genetic variation. Nature. 2015;526:68–74. 10.1038/nature15393. 26432245 PMC4750478

[10] Allen N, Sudlow C, Downey P, et al. UK Biobank: current status and what it means for epidemiology. Health Policy Technol. 2012;1:123–26. 10.1016/j.hlpt.2012.07.003.

[11] Karczewski KJ, Francioli LC, Tiao G, et al. The mutational constraint spectrum quantified from variation in 141,456 humans. Nature. 2020;581:434–43. 10.1038/s41586-020-2308-7. 32461654 PMC7334197

[12] Zhou H, Arapoglou T, Li X, et al. FAVOR: functional annotation of variants online resource and annotator for variation across the human genome. Nucleic Acids Res. 2023;51:D1300–11. 10.1093/nar/gkac966. 36350676 PMC9825437

[13] Cooper DN, Ball EV, Krawczak M. The human gene mutation database. Nucleic Acids Res. 1998;26:285–87. 10.1093/nar/26.1.285. 9399854 PMC147254

[14] Ganesan K, Kulandaisamy A, Binny Priya S, et al. HuVarBase: a human variant database with comprehensive information at gene and protein levels. PLoS One. 2019;14:e0210475. 10.1371/journal.pone.0210475. 30703169 PMC6354970

[15] Rehm HL, Berg JS, Brooks LD et al. ClinGen—The Clinical Genome Resource. N Engl J Med. 2015;372:2235–42. 10.1056/NEJMsr1406261. 26014595 PMC4474187

[16] Hamosh A, Scott AF, Amberger J, et al. Online Mendelian Inheritance in Man (OMIM). Hum Mutat. 2000;15:57–61. 10.1002/(sici)1098-1004(200001)15:1<57::Aid-humu12>3.0.Co;2-g. 10612823

[17] Weinreich SS, Mangon R, Sikkens JJ et al. [Orphanet: a European database for rare diseases]. Ned Tijdschr Geneeskd. 2008;152:518–19. Orphanet: een Europese database over zeldzame ziekten. 18389888

[18] Robinson PN, Köhler S, Bauer S, et al. The Human Phenotype Ontology: a tool for annotating and analyzing human hereditary disease. Am J Hum Genet. 2008;83:610–15. 10.1016/j.ajhg.2008.09.017. 18950739 PMC2668030

[19] Ashburner M, Ball CA, Blake JA, et al. Gene ontology: tool for the unification of biology. The Gene Ontology Consortium. Nat Genet. 2000;25:25–29. 10.1038/75556. 10802651 PMC3037419

[20] Bamford S, Dawson E, Forbes S, et al. The COSMIC (Catalogue of Somatic Mutations in Cancer) database and website. Br J Cancer. 2004;91:355–58. 10.1038/sj.bjc.6601894. 15188009 PMC2409828

[21] Ainscough BJ, Griffith M, Coffman AC et al. DoCM: a database of curated mutations in cancer. Nat Methods. 2016;13:806–807. 10.1038/nmeth.4000. 27684579 PMC5317181

[22] Liu Y, Sun J, Zhao M. ONGene: a literature-based database for human oncogenes. J Genet Genomics. 2017;44:119–21. 10.1016/j.jgg.2016.12.004. 28162959

[23] Findlay GM, Daza RM, Martin B et al. Accurate classification of BRCA1 variants with saturation genome editing. Nature. 2018;562:217–22. 10.1038/s41586-018-0461-z. 30209399 PMC6181777

[24] Leinonen R, Diez FG, Binns D et al. UniProt archive. Bioinformatics. 2004;20:3236–37. 10.1093/bioinformatics/bth191. 15044231

[25] Berman HM, Westbrook J, Feng Z, et al. The Protein Data Bank. Nucleic Acids Res. 2000;28:235–42. 10.1093/nar/28.1.235. 10592235 PMC102472

[26] Sasidharan Nair P, Vihinen M. VariBench: a benchmark database for variations. Hum Mutat. 2013;34:42–49. 10.1002/humu.22204. 22903802

[27] Schaafsma GC, Vihinen M. VariSNP, a benchmark database for variations from dbSNP. Hum Mutat. 2015;36:161–66. 10.1002/humu.22727. 25385275

[28] Wang Z, Zhao G, Zhu Z, et al. VarCards2: an integrated genetic and clinical database for ACMG-AMP variant-interpretation guidelines in the human whole genome. Nucleic Acids Res. 2024;52:D1478–89. 10.1093/nar/gkad1061. 37956311 PMC10767961

[29] Yazar M, Ozbek P. In silico tools and approaches for the prediction of functional and structural effects of single-nucleotide polymorphisms on proteins: an expert review. OMICS. 2021;25:23–37. 10.1089/omi.2020.0141. 33058752

[30] Ng PC, Henikoff S. Predicting deleterious amino acid substitutions. Genome Res. 2001;11:863–74. 10.1101/gr.176601. 11337480 PMC311071

[31] Ramensky V, Bork P, Sunyaev S. Human non-synonymous SNPs: server and survey. Nucleic Acids Res. 2002;30:3894–900. 10.1093/nar/gkf493. 12202775 PMC137415

[32] van der Velde KJ, de Boer EN, van Diemen CC, et al. GAVIN: Gene-Aware Variant INterpretation for medical sequencing. Genome Biol. 2017;18:6. 10.1186/s13059-016-1141-7. 28093075 PMC5240400

[33] Kircher M, Witten DM, Jain P, et al. A general framework for estimating the relative pathogenicity of human genetic variants. Nat Genet. 2014;46:310–15. 10.1038/ng.2892. 24487276 PMC3992975

[34] Ertürk RA, Baysan M. Utilizing tree-based algorithms for genetic variant interpretation. In: 2024 9th International Conference on Computer Science and Engineering (UBMK). 2024:689–94. 10.1109/UBMK63289.2024.10773498.

[35] Reva B, Antipin Y, Sander C. Predicting the functional impact of protein mutations: application to cancer genomics. Nucleic Acids Res. 2011;39:e118. 10.1093/nar/gkr407. 21727090 PMC3177186

[36] Shihab HA, Gough J, Cooper DN et al. Predicting the functional, molecular, and phenotypic consequences of amino acid substitutions using hidden Markov models. Hum Mutat. 2013;34:57–65. 10.1002/humu.22225. 23033316 PMC3558800

[37] Ionita-Laza I, McCallum K, Xu B et al. A spectral approach integrating functional genomic annotations for coding and noncoding variants. Nat Genet. 2016;48:214–20. 10.1038/ng.3477. 26727659 PMC4731313

[38] Davydov EV, Goode DL, Sirota M et al. Identifying a high fraction of the human genome to be under selective constraint using GERP++. PLoS Comput Biol. 2010;6:e1001025. 10.1371/journal.pcbi.1001025. 21152010 PMC2996323

[39] Burke W, Parens E, Chung WK et al. The challenge of genetic variants of uncertain clinical significance: a narrative review. Ann Intern Med. 2022;175:994–1000. 10.7326/M21-4109. 35436152 PMC10555957

[40] MacEachern SJ, Forkert ND. Machine learning for precision medicine. Genome. 2021;64:416–25. 10.1139/gen-2020-0131. 33091314

[41] Adzhubei IA, Schmidt S, Peshkin L, et al. A method and server for predicting damaging missense mutations. Nat Methods. 2010;7:248–49. 10.1038/nmeth0410-248. 20354512 PMC2855889

[42] Jordan DM, Kiezun A, Baxter SM et al. Development and validation of a computational method for assessment of missense variants in hypertrophic cardiomyopathy. Am J Hum Genet. 2011;88:183–92. 10.1016/j.ajhg.2011.01.011. 21310275 PMC3035712

[43] Kaminker JS, Zhang Y, Watanabe C et al. CanPredict: a computational tool for predicting cancer-associated missense mutations. Nucleic Acids Res. 2007;35:W595–98. 10.1093/nar/gkm405. 17537827 PMC1933186

[44] Xiong HY, Alipanahi B, Lee LJ, et al. The human splicing code reveals new insights into the genetic determinants of disease. Science. 2015;347:1254806. 10.1126/science.1254806. 25525159 PMC4362528

[45] Capriotti E, Calabrese R, Casadio R. Predicting the insurgence of human genetic diseases associated to single point protein mutations with support vector machines and evolutionary information. Bioinformatics. 2006;22:2729–34. 10.1093/bioinformatics/btl423. 16895930

[46] Carter H, Douville C, Stenson PD et al. Identifying Mendelian disease genes with the variant effect scoring tool. BMC Genomics. 2013;14:S3. 10.1186/1471-2164-14-s3-s3. PMC3665549 23819870

[47] Dong C, Wei P, Jian X, et al. Comparison and integration of deleteriousness prediction methods for nonsynonymous SNVs in whole exome sequencing studies. Hum Mol Genet. 2014;24:2125–37. 10.1093/hmg/ddu733. 25552646 PMC4375422

[48] Li B, Wei P, Jian X et al. Automated inference of molecular mechanisms of disease from amino acid substitutions. Bioinformatics. 2009;25:2744–50. 10.1093/bioinformatics/btp528. 19734154 PMC3140805

[49] Danis D, Jacobsen JOB, Carmody LC, et al. Interpretable prioritization of splice variants in diagnostic next-generation sequencing. Am Hum Genet. 2021;108:1564–77. 10.1016/j.ajhg.2021.06.014. PMC8456162 34289339

[50] Ritchie GR, Dunham I, Zeggini E, et al. Functional annotation of noncoding sequence variants. Nat Methods. 2014;11:294–96. 10.1038/nmeth.2832. 24487584 PMC5015703

[51] Fang M, Su Z, Abolhassani H et al. VIPPID: a gene-specific single nucleotide variant pathogenicity prediction tool for primary immunodeficiency diseases. Brief Bioinform. 2022;23:bbac176. 10.1093/bib/bbac176. 35598327 PMC9487673

[52] Zeng B, Liu DC, Huang JG et al. PdmIRD: missense variants pathogenicity prediction for inherited retinal diseases in a disease-specific manner. Hum Genet. 2024;143:331–42. 10.1007/s00439-024-02645-6. 38478153

[53] Li S, van der Velde KJ, de Ridder D et al. CAPICE: a computational method for consequence-agnostic pathogenicity interpretation of clinical exome variations. Genome Med. 2020;12:75. 10.1186/s13073-020-00775-w. 32831124 PMC7446154

[54] Wei Y, Zhang T, Wang B et al. INDELpred: improving the prediction and interpretation of indel pathogenicity within the clinical genome. HGG Adv. 2024;5:100325. 10.1016/j.xhgg.2024.100325. 38993112 PMC11321314

[55] Jagadeesh KA, Wenger AM, Berger MJ et al. M-CAP eliminates a majority of variants of uncertain significance in clinical exomes at high sensitivity. Nat Genet. 2016;48:1581–86. 10.1038/ng.3703. 27776117

[56] Kabir M, Ahmed S, Zhang H et al. PON-P3: accurate prediction of pathogenicity of amino acid substitutions. Int J Mol Sci. 2025;26:2004. 10.3390/ijms26052004. 40076632 PMC11899954

[57] Zhang X, Walsh R, Whiffin N, et al. Disease-specific variant pathogenicity prediction significantly improves variant interpretation in inherited cardiac conditions. Genet Med. 2021;23:69–79. 10.1038/s41436-020-00972-3. 33046849 PMC7790749

[58] Gunning AC, Fryer V, Fasham J, et al. Assessing performance of pathogenicity predictors using clinically relevant variant datasets. J Med Genet. 2021;58:547–55. 10.1136/jmedgenet-2020-107003. 32843488 PMC8327323

[59] Whalen S, Pandey G. A comparative analysis of ensemble classifiers: case studies in genomics. In: 2013 IEEE 13th International Conference on Data Mining. 2013:807–16., 10.1109/ICDM.2013.21.

[60] Ioannidis NM, Rothstein JH, Pejaver V, et al. REVEL: an ensemble method for predicting the pathogenicity of rare missense variants. Am J Hum Genet. 2016;99:877–85. 10.1016/j.ajhg.2016.08.016. 27666373 PMC5065685

[61] Petrini A, Mesiti M, Schubach M et al. parSMURF, a high-performance computing tool for the genome-wide detection of pathogenic variants. Gigascience. 2020;9:giaa052. 10.1093/gigascience/giaa052. 32444882 PMC7244787

[62] Zou J, Huss M, Abid A, et al. A primer on deep learning in genomics. Nat Genet. 2019;51:12–18. 10.1038/s41588-018-0295-5. 30478442 PMC11180539

[63] Quang D, Chen Y, Xie X. DANN: a deep learning approach for annotating the pathogenicity of genetic variants. Bioinformatics. 2015;31:761–63. 10.1093/bioinformatics/btu703. 25338716 PMC4341060

[64] Sundaram L, Gao H, Padigepati SR, et al. Predicting the clinical impact of human mutation with deep neural networks. Nat Genet. 2018;50:1161–70. 10.1038/s41588-018-0167-z. 30038395 PMC6237276

[65] Zhou J, Troyanskaya OG. Predicting effects of noncoding variants with deep learning-based sequence model. Nat Methods. 2015;12:931–34. 10.1038/nmeth.3547. 26301843 PMC4768299

[66] Kelley DR, Reshef YA, Bileschi M et al. Sequential regulatory activity prediction across chromosomes with convolutional neural networks. Genome Res. 2018;28:739–50. 10.1101/gr.227819.117. 29588361 PMC5932613

[67] Jaganathan K, Ersaro N, Novakovsky G et al. Predicting expression-altering promoter mutations with deep learning. Science. 2025;389:eads7373. 10.1126/science.ads7373. 40440429

[68] Jaganathan K, Kyriazopoulou Panagiotopoulou S, McRae JF, et al. Predicting splicing from primary sequence with deep learning. Cell. 2019;176:535–548.e24. 10.1016/j.cell.2018.12.015. 30661751

[69] Cheng J, Panagiotopoulou SK, McRae JF, et al. MMSplice: modular modeling improves the predictions of genetic variant effects on splicing. Genome Biol. 2019;20:48. 10.1186/s13059-019-1653-z. 30823901 PMC6396468

[70] LeCun Y, Bengio Y, Hinton G. Deep learning. Nature. 2015;521:436–44. 10.1038/nature14539. 26017442

[71] Cheng J, Novati G, Pan J et al. Accurate proteome-wide missense variant effect prediction with AlphaMissense. Science. 2023;381:eadg7492. 10.1126/science.adg7492. 37733863

[72] Vaswani A, Shazeer N, Parmar N, et al. Attention is all you need. Presented at the Proceedings of the 31st International Conference on Neural Information Processing Systems, Long Beach, California, USA, 2017.

[73] Rives A, Meier J, Sercu T et al. Biological structure and function emerge from scaling unsupervised learning to 250 million protein sequences. Proc Natl Acad Sci USA. 2021;118:e2016239118. 10.1073/pnas.2016239118. 33876751 PMC8053943

[74] Brandes N, Goldman G, Wang CH et al. Genome-wide prediction of disease variant effects with a deep protein language model. Nat Genet. 2023;55:1512–1522. 10.1038/s41588-023-01465-0. 37563329 PMC10484790

[75] Lin W, Wells J, Wang Z et al. Enhancing missense variant pathogenicity prediction with protein language models using VariPred. Sci Rep. 2024;14:8136. 10.1038/s41598-024-51489-7. 38584172 PMC10999449

[76] Kulikova AV, Diaz DJ, Chen T et al. Two sequence- and two structure-based ML models have learned different aspects of protein biochemistry. Sci Rep. 2023;13:13280. 10.1038/s41598-023-40247-w. 37587128 PMC10432456

[77] Marquet C, Heinzinger M, Olenyi T, et al. Embeddings from protein language models predict conservation and variant effects. Hum Genet. 2022;141:1629–47. 10.1007/s00439-021-02411-y. 34967936 PMC8716573

[78] Avsec Ž, Agarwal V, Visentin D, et al. Effective gene expression prediction from sequence by integrating long-range interactions. Nat Methods. 2021;18:1196–203. 10.1038/s41592-021-01252-x. 34608324 PMC8490152

[79] Avsec Ž, Latysheva N, Cheng J, et al. AlphaGenome: advancing regulatory variant effect prediction with a unified DNA sequence model. bioRxiv. 2025; 10.1101/2025.06.25.661532.

[80] Gao Z, Liu Q, Zeng W et al. EpiGePT: a pretrained transformer-based language model for context-specific human epigenomics. Genome Biol. 2024;25:310. 10.1186/s13059-024-03449-7. 39696471 PMC11657395

[81] Jumper J, Evans R, Pritzel A, et al. Highly accurate protein structure prediction with AlphaFold. Nature. 2021;596:583–89. 10.1038/s41586-021-03819-2. 34265844 PMC8371605

[82] Jiang TT, Fang L, Wang K. Deciphering “the language of nature”: a transformer-based language model for deleterious mutations in proteins. Innovation. 2023;4:100487. 10.1016/j.xinn.2023.100487. 37636282 PMC10448337

[83] Liang L, Chen Y, Wang T et al. Genetic transformer: an innovative large language model driven approach for rapid and accurate identification of causative variants in rare Genetic diseases. medRxiv. 2024; 10.1101/2024.07.18.24310666.

[84] You N, van Dijk C, Timpanaro I, et al. SpliceTransformer predicts tissue-specific splicing linked to human diseases. Nat Commun. 2024;15:9129. 10.1038/s41467-024-53088-6. 39443442 PMC11500173

[85] Luo J, Zhou T, You X, et al. Assessing concordance among human, in silico predictions and functional assays on genetic variant classification. Bioinformatics. 2019;35:5163–70. 10.1093/bioinformatics/btz442. 31141141

[86] McLaren W, Zhou T, You X, et al. The Ensembl variant effect predictor. Genome Biol. 2016;17:122. 10.1186/s13059-016-0974-4. 27268795 PMC4893825

[87] Wang K, Li M, Hakonarson H. ANNOVAR: functional annotation of genetic variants from high-throughput sequencing data. Nucleic Acids Res. 2010;38:e164. 10.1093/nar/gkq603. 20601685 PMC2938201

[88] Kopanos C, Tsiolkas V, Kouris A et al. VarSome: the human genomic variant search engine. Bioinformatics. 2019;35:1978–80. 10.1093/bioinformatics/bty897. 30376034 PMC6546127

[89] Baux D, Goethem CV, Ardouin O et al. MobiDetails: online DNA variants interpretation. Eur J Hum Genet. 2021;29:356–60. 10.1038/s41431-020-00755-z. 33161418 PMC7868358

[90] Li Q, Wang K. InterVar: clinical interpretation of genetic variants by the 2015 ACMG-AMP Guidelines. Am Hum Genet. 2017;100:267–80. 10.1016/j.ajhg.2017.01.004. PMC5294755 28132688

[91] Robinson PN, Köhler S, Oellrich A et al. Improved exome prioritization of disease genes through cross-species phenotype comparison. Genome Res. 2014;24:340–48. 10.1101/gr.160325.113. 24162188 PMC3912424

[92] Maharana K, Mondal S, Nemade B. A review: data pre-processing and data augmentation techniques. Glob Transit Proc. 2022;3:91–99. 10.1016/j.gltp.2022.04.020.

[93] Pfob A, Lu S-C, Sidey-Gibbons C. Machine learning in medicine: a practical introduction to techniques for data pre-processing, hyperparameter tuning, and model comparison. BMC Med Res Method. 2022;22:282. 10.1186/s12874-022-01758-8. PMC9624048 36319956

[94] Marzban C . The ROC curve and the area under it as performance measures. Weather Forecast. 2004;19:1106–14. 10.1175/825.1.

[95] Krusche P, Trigg L, Boutros PC et al. Best practices for benchmarking germline small-variant calls in human genomes. Nat Biotechnol. 2019;37:555–60. 10.1038/s41587-019-0054-x. 30858580 PMC6699627

[96] Zitnik M, Nguyen F, Wang B et al. Machine learning for integrating data in biology and medicine: principles, practice, and opportunities. Inf Fusion. 2019;50:71–91. 10.1016/j.inffus.2018.09.012. 30467459 PMC6242341

[97] Charilaou P, Battat R. Machine learning models and over-fitting considerations. World J Gastroenterol. 2022;28(5):605–607. 10.3748/wjg.v28.i5.605. 35316964 PMC8905023

[98] Hoffman-Andrews L . The known unknown: the challenges of genetic variants of uncertain significance in clinical practice. J Law Biosci. 2017;4:648–57. 10.1093/jlb/lsx038. 29868193 PMC5965500

[99] Schnabel T, Swaminathan A, Singh A et al. Recommendations as treatments: debiasing learning and evaluation. In: International Conference on Machine Learning, 2016.

[100] Karimi R, Freudenthaler C, Nanopoulos A et al. Towards optimal active learning for matrix factorization in Recommender systems. In: 2011 IEEE 23rd International Conference on Tools with Artificial Intelligence, 2011:1069–76., 10.1109/ICTAI.2011.182.

[101] Gasperini M, Starita L, Shendure J. The power of multiplexed functional analysis of genetic variants. Nat Protoc. 2016;11:1782–87. 10.1038/nprot.2016.135. 27583640 PMC6690347

[102] McEwen AE, Tejura M, Fayer S et al. Multiplexed assays of variant effect for clinical variant interpretation. Nat Rev Genet. 2025;27:137–154. 10.1038/s41576-025-00870-x. 40691352

[103] Landry LG, Ali N, Williams DR et al. Lack of diversity In genomic databases is a barrier to translating precision medicine research into practice. Health Aff (Millwood). 2018;37:780–85. 10.1377/hlthaff.2017.1595. 29733732

[104] Martin AR, Kanai M, Kamatani Y et al. Clinical use of current polygenic risk scores may exacerbate health disparities. Nat Genet. 2019;51:584–91. 10.1038/s41588-019-0379-x. 30926966 PMC6563838

[105] Hindorff LA, Bonham VL, Ohno-Machado L. Enhancing diversity to reduce health information disparities and build an evidence base for genomic medicine. Per Med. 2018;15:403–12. 10.2217/pme-2018-0037. 30209973 PMC6287493

[106] Curtis D . Assessment of ability of AlphaMissense to identify variants affecting susceptibility to common disease. Eur J Hum Genet. 2024;32:1419–27. 10.1038/s41431-024-01675-y. 39097650 PMC11576984

[107] Boyle EA, Li YI, Pritchard JK. An expanded view of complex traits: from polygenic to omnigenic. Cell. 2017;169:1177–86. 10.1016/j.cell.2017.05.038. 28622505 PMC5536862

[108] Frazer J, Notin P, Dias M et al. Disease variant prediction with deep generative models of evolutionary data. Nature. 2021;599:91–95. 10.1038/s41586-021-04043-8. 34707284

[109] Raz A, Heinrichs B, Avnoon N et al. Prediction and explainability in AI: striking a new balance?. Big Data Soc. 2024;11:20539517241235871. 10.1177/20539517241235871.

[110] Zheng Z, Wang Y, Huang Y et al. Attention heads of large language models. Patterns (N Y). 2025;6:101176. 10.1016/j.patter.2025.101176. 40041856 PMC11873009

[111] Mallela IR, Aravind S, Tharan O, et al. Explainable AI for compliance and regulatory models. Int J Res Publ Semin. 2020; 11(4):319–339. 10.36676/jrps.v11.i4.1584.

[112] Doig KD, Perera R, Kankanige Y, et al. Using artificial intelligence (AI) to model clinical variant reporting for next generation sequencing (NGS) oncology assays. BioData Min. 2025;18:74. 10.1186/s13040-025-00489-y. 41163022 PMC12570631

[113] Costa M, García S A, León A et al. The promises and pitfalls of automated variant interpretation: a comprehensive review. Briefings Bioinf. 2025;26:bbaf545. 10.1093/bib/bbaf545. PMC12513165 41071614

[114] Stein D, Kars ME, Milisavljevic B, et al. Expanding the utility of variant effect predictions with phenotype-specific models. Nat Commun. 2025;16:11113. 10.1038/s41467-025-66607-w. 41315332 PMC12705684

[115] Subramanian I, Verma S, Kumar S et al. Multi-omics data integration, interpretation, and its application. Bioinform Biol Insights. 2020;14:1177932219899051. 10.1177/1177932219899051. 32076369 PMC7003173

[116] Yang T, Wei P, Pan W. Integrative analysis of multi-omics data for discovering low-frequency variants associated with low-density lipoprotein cholesterol levels. Bioinformatics. 2020;36:5223–28. 10.1093/bioinformatics/btaa898. PMC7850048 33070182

[117] Hsu CY, Askar S, Alshkarchy SS, et al. AI-driven multi-omics integration in precision oncology: bridging the data deluge to clinical decisions. Clin Exp Med. 2025;26:29. 10.1007/s10238-025-01965-9. 41266662 PMC12634751

[118] Hernandez-Lemus E, Ochoa S. Methods for multi-omic data integration in cancer research. Front Genet. 2024;15:1425456. 10.3389/fgene.2024.1425456. 39364009 PMC11446849

[119] Chui MM-C, Kwong AK-Y, Leung HYC, et al. An outlier approach: advancing diagnosis of neurological diseases through integrating proteomics into multi-omics guided exome reanalysis. NPJ Genom Med. 2025;10(1):36. 10.1038/s41525-025-00493-5. 40319040 PMC12049463

[120] Smirnov D, Konstantinovskiy N, Prokisch H. Integrative omics approaches to advance rare disease diagnostics. J Inherit Metab Dis. 2023;46:824–38. 10.1002/jimd.12663. 37553850

[121] Gerasimavicius L, Teichmann SA, Marsh JA. Leveraging protein structural information to improve variant effect prediction. Curr Opin Struct Biol. 2025;92:103023. 10.1016/j.sbi.2025.103023. 39987793 PMC7618235

[122] Ding N, Qin Y, Yang G, et al. Parameter-efficient fine-tuning of large-scale pre-trained language models. Nat Mach Intell. 2023;5:220–235. 10.1038/s42256-023-00626-4

[123] Wang L, Chen X, Deng XW, et al. Prompt engineering in consistency and reliability with the evidence-based guideline for LLMs. NPJ Digital Med. 2024;7:41. 10.1038/s41746-024-01029-4 PMC10879172 38378899

[124] Feng H, Wu L, Zhao B et al. Benchmarking DNA foundation models for genomic and genetic tasks. Nat Commun. 2025;16:10780. 10.1038/s41467-025-65823-8. 41315262 PMC12663285

[125] Sajun AR, Zualkernan I, Sankalpa D. A historical survey of advances in transformer architectures. Appl Sci. 2024;14:4316. 10.3390/app14104316.

[126] Fowler DM, Adams DJ, Gloyn AL et al. An atlas of variant effects to understand the genome at nucleotide resolution. Genome Biol. 2023;24:147. 10.1186/s13059-023-02986-x. 37394429 PMC10316620

[127] Rubin AF, Stone J, Bianchi AH, et al. MaveDB 2024: a curated community database with over seven million variant effects from multiplexed functional assays. Genome Biol. 2025;26:13. 10.1186/s13059-025-03476-y. 39838450 PMC11753097

[128] Buphamalai P, Kokotovic T, Nagy V et al. Network analysis reveals rare disease signatures across multiple levels of biological organization. Nat Commun. 2021;12:6306. 10.1038/s41467-021-26674-1. 34753928 PMC8578255

[129] Dandara C, Huzair F, Borda-Rodriguez A et al. H3Africa and the African life sciences ecosystem: building sustainable innovation. OMICS. 2014;18:733–39. 10.1089/omi.2014.0145. 25454511 PMC4253142

[130] Zhao Z, Fritsche LG, Smith JA, et al. The construction of cross-population polygenic risk scores using transfer learning. Am J Hum Genet. 2022;109:1998–2008. 10.1016/j.ajhg.2022.09.010. 36240765 PMC9674947

[131] Tian P, Chan TH, Wang YF, et al. Multiethnic polygenic risk prediction in diverse populations through transfer learning. Front Genet. 2022;13:906965. 10.3389/fgene.2022.906965. 36061179 PMC9438789

[132] Tzeng E, Hoffman J, Saenko K, et al. Adversarial discriminative domain adaptation. In: Proceedings of the IEEE Conference on Computer Vision and Pattern Recognition, 2017:7167–76.

[133] Cara MC, Montserrat DM, Ioannidis AG. PopGenAdapt: semi-supervised domain adaptation for genotype-to-phenotype prediction in underrepresented populations. Pac Symp Biocomput. 2024;29:327–40. PMC10906137 38160290

[134] Yelmen B, Decelle A, Ongaro L, et al. Creating artificial human genomes using generative neural networks. PLoS Genet. 2021;17:e1009303. 10.1371/journal.pgen.1009303. 33539374 PMC7861435

[135] Das S, Shi X. Offspring GAN augments biased human genomic data. Presented at the Proceedings of the 13th ACM International Conference on Bioinformatics, Computational Biology and Health Informatics, Northbrook, Illinois, 2022. 10.1145/3535508.3545537.

[136] Zhang H, Zhan J, Jin J, et al. A new method for multiancestry polygenic prediction improves performance across diverse populations. Nat Genet. 2023;55:1757–68. 10.1038/s41588-023-01501-z. 37749244 PMC10923245

[137] Zhang J, Zhan J, Jin J et al. An ensemble penalized regression method for multi-ancestry polygenic risk prediction. Nat Commun. 2024;15:3238. 10.1038/s41467-024-47357-7. 38622117 PMC11271575

[138] Gao C, Tubbs JD, Han Y et al. Unsupervised ensemble learning for efficient integration of pre-trained polygenic risk scores. medRxiv. 2025; 10.1101/2025.01.06.25320058.

[139] Williams J, Chen T, Hua X et al. Integrating common and rare variants improves polygenic risk prediction across diverse populations. medRxiv. 2024; 10.1101/2024.11.05.24316779. PMC13323733 42031785

[140] Li H, Zeng J, Snyder MP, et al. PRS-Net: interpretable polygenic risk scores via geometric learning. In: Research in Computational Molecular Biology. Cham: Springer Nature Switzerland; 2024:377–80.

[141] Li H, Zeng J, Snyder MP et al. Modeling gene interactions in polygenic prediction via geometric deep learning. Genome Res. 2025;35:178–87. 10.1101/gr.279694.124. 39562137 PMC11789630

