# Supplementary material for: Harnessing artificial intelligence for genomic variant prediction: advances, challenges, and future directions
Source: Gigascience. 2026 Jan 10;15:giag004. doi: 10.1093/gigascience/giag004 (PMC12888390; doi:10.1093/gigascience/giag004)
Supplement: giag004_Supplemental_File [file giag004_supplemental_file.docx]

**Supplementary Table 1.** Existing in silico Predictor for Genetic Variant Pathogenicity

| **VIPs** | **Type** | **Input Features** | **Training Dataset** | **Validating Datasets** | **Description** | **Input/Output** | **Type of Tool** | **Variant Type** | **Application** | **Website** | **Programming Language** | **Algorithm/**  **Model** | **Classification** | **Threshold** | **Reported**  **Performance** | **Reference** |
| --- | --- | --- | --- | --- | --- | --- | --- | --- | --- | --- | --- | --- | --- | --- | --- | --- |
| AIVAR | Multiple Features | Functional, structural and regional annotations, allele frequency (AF), predictive score from existing tools | ClinVar, SGE_BRCA1 | Lincoln_BRCA1/2, ClinVar, TopGeneDB (internal) | Classifies genetic variants into pathogenicity categories. Aims to reclassify variants of uncertain significance (VUS) into clinically informative categories | Input: annotated variant file from ANNOVAR;  Output: AIVAR score and classification | Source code | Coding variants | General-purpose (not disease-specific) | <https://github.com/TopGene/AIvar> | Perl, Python | Neural Network (NN) | Benign/Likely Benign (B/LB), Pathogenic/Likely Pathogenic (P/LP) | <0.5 → B/LB;  >0.5 → P/LP | ROC-AUC: 0.85-0.87 | [1] |
| AlphaGenome | Multiple Features | Raw 1 Mb DNA sequence windows (hg38 + mouse mm10) | ENCODE (phase-IV), Genotype-Tissue Expression (GTEx) v8, Roadmap Epigenomics, 4D-Nucleome, FANTOM5, CAGE-peak, PRO-cap, ChIA-PET, Hi-C, RNA-seq, ATAC-seq, DNase-seq, ChIP-seq, human & mouse loci across >400 biosamples | 18 external benchmarks: ENCODE3 cCRE-MPRA, STARR-seq enhancer assays, GTEx expression Quantitative Trait Loci (eQTL) & splicing Quantitative Trait Loci (sQTL), Roadmap Epigenome matched expression, ClinVar pathogenic variants, DeepSEA, Basenji2, SpliceAI comparative sets, TAL1 enhancer saturation mutagenesis, 1000 Genomes, gnomAD common variant controls | A unified deep learning (DL) model that predicts thousands of functional genomic tracks from a 1 Mb DNA sequence with up to single base-pair resolution. It is designed to overcome the limitations of previous models regarding input sequence length and prediction resolution | Input: A VCF or BED file (hg38) with genomic coordinates, or a raw 1 Mb DNA sequence string;  Output: AlphaGenome score, classification, tissue-specific functional predictions (RNA expression, splicing, chromatin accessibility, Transcription factor (TF) binding, 3-D contacts) | Source code + Web API | Non-coding variants (SNVs, indels, structural variations) | General-purpose (not disease-specific), rare disease diagnostics, GWAS interpretation, and sequence design | <http://deepmind.google.com/science/alphagenome> | Python | Ensemble of deep transformers (DNA-BERT + Enformer-style architecture) fine-tuned with multi-task heads | Quantitative predictions across multiple genomic modalities | - | ROC-AUC: ≈ 0.80 | [2] |
| Align-GVGD | Homology | Multiple Sequence Alignment (MSA) | - | FamHx-LR | Combines biophysical characteristics of amino acids and evolutionary conservation to predict the impact of missense changes | Input: protein sequences;  Output: C-score representing the range from least likely (C0) to most likely (C65) to interfere with function | Web-based tool | Missense mutations | BRCA1, BRCA2, and any disease where missense mutations are relevant | <http://agvgd.hci.utah.edu/agvgd_input.php> | R | Grantham Variation/Grantham Deviation (GV/GD) rule-based model | Benign, Pathogenic | >C35 → Likely Pathogenic | - | [3] |
| AlphaMissense | Protein Structure and Sequence-based | Protein sequences, embeddings from protein language models (PLM) (e.g., ESM-1b), AlphaFold structure-derived features | gnomAD, AlphaFold | ClinVar, DDD, MAVE, ACMG gene set, Cancer hotspot variants | An adaptation of AlphaFold fine-tuned on human and primate variant population frequency databases to predict missense variant pathogenicity | Input: -;  Output: AlphaMissense score and classification | Precomputed scores | Missense mutations | General-purpose (not disease-specific) | <https://console.cloud.google.com/storage/browser/dm_alphamissense;tab=objects?prefix=&forceOnObjectsSortingFiltering=false> | Python | Protein-specific large language models (Protein LLMs) + AlphaFold structure | Likely Benign, Ambiguous, Likely Pathogenic | >0.56 →  Likely Pathogenic | ROC-AUC: ≈ 0.94 | [4] |
| Basenji | Multiple Features | 131-kb DNA sequence regions | ENCODE (DNase & histone ChIP), Roadmap Epigenomics (DNase & histone ChIP), FANTOM5 CAGE | ENCODE3 cCRE-MPRA, GTEx eQTL / sQTL | A deep convolutional neural network (CNN) that predicts cell-type-specific epigenetic and transcriptional profiles from large (131-kb) DNA sequences. It is designed to model distal regulatory interactions and predict quantitative genomic profiles to interpret the influence of genomic variants | Input: 131-kb DNA sequence (FASTA region) + optional JSON cell-type metadata;  Output: predicted signal tracks (HDF5/BigWig) and SNP-Expression Difference (SED) scores | Source code | Non-coding variants (SNPs) | General-purpose (not disease-specific), with specific utility for analyzing eQTLs and fine-mapping disease-associated loci from GWAS | <https://www.github.com/calico/basenji> | Python | Deep dilated CNN with multitask Poisson regression. | Regression (Poisson λ) for signal, signed SED for variant impact | - | PR-AUC: 0.58 | [5] |
| CADD | Multiple Features | 63 annotations, including conservation (GERP++, phastCons, phyloP), regulatory (DNase, TFBS), protein-level (SIFT, PolyPhen), VEP output, etc | Simulated de novo variants | ClinVar, ESP, HbVar, IARC TP53, ASD/ID Studies, Patwardhan Enhancer/Promoter Assays, GWAS Catalog, Personal Genomes (11 Men) | Framework that integrates diverse annotations to estimate deleteriousness of SNVs and indels genome-wide. Outputs a continuous score (raw and scaled C-scores), predictive of functional impact and pathogenicity across variant types | Input: genetic variants;  Output: raw C-score and PHRED-scaled score | Web-based tool | SNVs, small indels (<50 bp), coding, noncoding | General-purpose (not disease-specific) | <https://cadd.gs.washington.edu/> | Python | Support Vector Machine (SVM) | Non-Pathogenic (Benign), Pathogenic | - | ROC-AUC: 0.91-0.93 | [6] |
| CanPredict | Multiple Features | SIFT score, Pfam-based LogR.E-value, Gene Ontology Similarity Score (GOSS) | COSMIC, dbSNP | Cross-validation experiment, analysis of recurrently identified mutations, and data from large-scale cancer mutation screens | Uses SIFT, Pfam-based LogR.E-value metric, and GOSS analyzed by a random forest (RF) classifier to predict cancer-associated changes | Input: missense variant;  Output: probability of cancer association | Web-based tool | Missense mutations | Cancer | <http://genetics.bwh.harvard.edu/hcm/> | - | RF | Likely Non-Cancer, Not Determined, Likely Cancer | - | - | [7] |
| CAPICE | Multiple Features | Genomic annotations including conservation scores, regulatory features, AF, functional predictions | ClinVar, VKGL, van der Velde et al. datasets | ClinVar, VKGL, ExAC | Predicts the pathogenicity of rare coding variants using a machine learning (ML) model trained on large-scale exome data | Input: variants;  Output: CAPICE score and classification | Web-based tool & Stand-alone software | SNV, InDels | General-purpose (not disease-specific) | <https://vip.molgeniscloud.org> | Python | Gradient Boosting | Likely Benign, Likely Pathogenic | <0.5 → Likely Benign;  ≥0.5 → Likely Pathogenic | ROC-AUC: ≈ 0.97 | [8] |
| Cardioboost | Ensemble | 76 functional features, including grantham score, BLOSUM62, PAM250, SIFT, Polyphen2, LRT_score, MutationTaster, MutationAssessor, FATHMM, PROVEAN, VEST3, CADD, DANN, FATHMM-MKL, MetaSV, MetaLR, Eigen, M-CAP, REVEL, GERP++, PhyloP, integrated fitcons, PhastCons, SiPhy, paraZscore, paraZscore_exist, misbadness, misbadness_exist, MPC | Clinical Myopathies (CM) Dataset, Inherited Arrhythmia Dataset | - | Predict the pathogenicity of rare (gnomAD AF ≤0.1%) missense variant in genes associated with cardiomyopathies and arrhythmias that outperforms existing genome-wide prediction tools | Input: gene information;  Output: Cardioboost score and classification | Web based tool | Missense mutations | Familial Cardiomyopathies (HCM, DCM), Inherited Arrhythmia Syndromes (IAS): Long QT syndrome and Brugada syndrome | <https://www.cardiodb.org/cardioboost/> | R | Adaptive Boosting (Adaboost) classifiers | Benign, VUS, Disease-causing | ≤0.1 → Benign;  0.1< score <0.9 → VUS;  ≥0.9 → Disease-causing | ROC-AUC: ≈ 0.91 | [9] |
| ClinPred | Multiple Features | Several popular pathogenicity predictors, population AF | ClinVar | ClinVarTest, MouseVariSNP, DoCM, Oncogene, Tumor Suppressor Gene (TSG), Gain-of-Function, Loss-of-Function (LoF), Clinical Case Data, and BRCA1 Functional Dataset | Identifying disease-relevant nonsynonymous SNPs by analyzing multiple genomic features | Input: -;  Output: ClinPred score | Precomputed scores | Non-synonymous SNPs | Mendelian disease | <https://sites.google.com/site/clinpred/> | - | Combines Random Forest (cforest) and Gradient Boosted Decision Tree (XGBoost) | Benign, Pathogenic | <0.5 → Benign;  ≥0.5 → Pathogenic | ROC-AUC: ≈ 0.98 | [10] |
| Condel | Ensemble | 5 predictors, including Logre, MAPP, MutationAssessor, Polyphen2, SIFT | HumVar, HumDiv, COSMIC, p53 | - | Integrates the scores from different variant effect prediction tools to provide a consensus score on the deleteriousness of nsSNVs | Input: list of mutations (genomic or protein format);  Output: Condel consensus score and classification | Web-based tool | Non-synonymous SNVs | General-purpose (not disease-specific) | <http://bbglab.irbbarcelona.org/fannsdb/> | Perl | Weighted average of scores from multiple prediction tools | Tolerated, Deleterious | 0.0 → Neutral;  1.0 → Deleterious | Accuracy: 0.76–0.89 | [11] |
| DANN | Multiple Features | 949 features, including predictors, conservation scores, function annotation | CADD’s training set | ClinVar, ESP | Uses DL to predict the deleteriousness of genetic variants based on a wide range of genomic features | Input: genetic variants;  Output: DANN score | Source code | SNV (coding and noncoding) | General-purpose (not disease-specific) | <https://github.com/greenelab/deep-review/issues/5> | - | Deep Neural Network (DNN) | Tolerated, Deleterious | <0.5 → Tolerated;  >0.5 → Deleterious | ROC-AUC: 0.72-0.94 | [12] |
| DeepSEA | Multiple Features | One-hot encoded DNA sequence (1,000 bp); model is trained to learn chromatin features | ENCODE & Roadmap Epigenomics | Chromosome 7 (validation); Chromosomes 8–9 (testing) | Predicts functional effects of noncoding variants by learning regulatory code from DNA sequence. Trained with multitask DL to predict chromatin features | Input: 1,000 bp DNA sequence with ref/alt allele;  Output: probabilities of chromatin feature binding; delta score for variant effect | Web-based tool | Noncoding SNPs | General-purpose (not disease-specific) | <http://deepsea.princeton.edu/> | - | CNN | - | - | ROC-AUC: ≈ 0.96 | [13] |
| DeMaSk | Homology | MSA | Deep Mutational Scanning  (DMS) data for 18 proteins | DMS datasets for proteins that are not used for training | Predict the impact of missense mutations on a protein's function by leveraging data from DMS studies | Input: protein sequence;  Output: fitness impact scores for all amino acid substitutions with accompanying entropy, variant frequency, and DMS-fit matrix features | Web-based tool & Stand-alone software | Missense mutations | Not disease-specific; applicable to studies of protein function | <https://demask.princeton.edu/> | Python, R | Linear model | - | - | - | [14] |
| DEOGEN/ DEOGEN2 | Multiple Features | PROVEAN score, Conservation Index, mutant/wildtype log-odd ratio, Early Folding predictions, PFAM log-odd score, Interaction patches annotation, RVIS, GDI, Recessiveness index, Gene essentiality, Pathway log-odd score | Humsavar11, PROVEAN/ Humsavar16 | Humsavar11, p53, F8, BRCA1, Independent blind set | DEOGEN and DEOGEN2 predict the pathogenicity of nsSNVs using ML models trained on a variety of genomic and evolutionary features | Input: sequence,  Uniprot ID;  Output: DEOGEN/ DEOGEN2 score and classification | Web-based tool | Missense SNVs and in-frame INDELs | Various diseases | DEOGEN:  <http://ibsquare.be/deogen>  DEOGEN2:  <http://deogen2.mutaframe.com/> | - | RF | Benign, Deleterious | <0.45 → Benign;  >0.45 → Deleterious | ROC-AUC: ≈ 0.94/ BAC: 0.87–0.89 | [15, 16] |
| DvPred | Multiple Features | Conservation scores, prediction scores, population frequencies, gene intolerance metrics, variants information | China Deafness Genetics Consortium (CDGC), Deafness Variation Database (DVD), ClinVar, HGMD | Same as training dataset (random subset) | A tool developed to classify the pathogenicity of nonsynonymous SNVs associated with hearing loss | Input: -;  Output:  DvPred score | Source code | SNVs | Hearing Loss | <https://github.com/WCH-IRD/DVPred> | Python | Gradient Boosting | Neutral, Deleterious | <0.5 → Neutral;  >0.5 → Deleterious | ROC-AUC: ≈ 0.98 | [17] |
| EFIN | Homology | Sequence conservation features | UniProt, HumDiv | Swiss-Prot, HumDiv | Uses a block-wise structure to evaluate sequence conservation and predict the functional impact of Amino Acid Substitution (AAS) | Input: protein sequence;  Output: EFIN score and classification | Web-based tool | Non-synonymous SNPs | General-purpose (not disease-specific) | <http://paed.hku.hk/efin/> | - | RF | Neutral, Damaging | Swiss-Prot:  >0.6 → Neutral;  <0.6 → Damaging  HumDiv:  >0.28 → Neutral;  <0.28 → Damaging | ROC-AUC: ≈ 0.91 | [18] |
| Eigen | Multiple Features | Protein function scores, evolutionary conservation scores, AF in four populations | dbSNFP, UCSC genome browser, 1KGP | ClinVar, BRCA1/2, MLL2, CFTR, De novo mutations (ASD, SCZ, EPI, ID, Control), GWAS SNPs, COSMIC | Integrates various genomic annotations to predict the functional importance of genetic variants using a ML model | Input: genetic variants;  Output: Eigen score | Precomputed scores | Coding (non-synonymous), Synonymous, Noncoding | General-purpose (not disease-specific) | <https://www.columbia.edu/~ii2135/eigen.html> | Perl | Unsupervised Statistical Learning | Not categorical; produces continuous Eigen score | - | ROC-AUC: ≈ 0.86 | [19] |
| Enformer | Multiple Features | 196,608 bp of one-hot encoded DNA sequence | ENCODE (DNase, histone ChIP, CAGE, RNA-seq), Roadmap Epigenomics (DNase, histone ChIP), mouse ENCODE RNA-seq | ENCODE3 cCRE-MPRA, GTEx eQTL, GTEx sQTL, CRISPR-FlowFISH, held-out chromosomes 1 and 8–22 | A DL architecture that uses transformer modules to significantly improve the accuracy of gene expression prediction from DNA sequence, with the ability to integrate information from long-range interactions up to 100 kb | Input: FASTA region 196 kb + JSON metadata;  Output: HDF5 (per-gene log TPM, per-bin regulatory tracks) & variant Δ-scores | Source code | Non-coding genetic variants | General-purpose (not disease-specific), fine-mapping of GWAS disease loci, prioritizing rare variants in genetic diseases, and interpretation of cis-regulatory evolution | <https://github.com/google-deepmind/deepmind-research/tree/master/enformer> | Python | Transformer | Regression (log TPM) + signed effect (Δ-logit) for variants | - | Pearson r: ≈ 0.85 | [20] |
| ENTPRISE | Multiple Features | Entropy derived from MSA, wild-type amino acid type, mutant amino acid type, domain composition of 20 amino acid types, contacting composition | ENTPRISE-TR (derived from PredictSNP’s OVERFIT set), 1KGP | COSMIC, TCGA, COBR, VariSNP, 1KGP, ENTPRISE-TE, ENTPRISE-balance, 1k-Genome set | Predicting human disease-associated amino acid substitutions from sequence entropy and predicted protein structures | Input: NCBI protein sequence ID, position of the amino acid substitution, reference amino acid type, substitution residue type;  Output: ENTPRISE score and cutoff information for disease-associated classification as well as sequence entropy | Web-based tool | Non-synonymous SNPs | General-purpose (not disease-specific) | <http://cssb.biology.gatech.edu/entprise/> | - | Regression model | Neutral, Disease-associated | ≤0.45 → Neutral;  >0.45 → Disease-associated | ROC-AUC: ≈ 0.90 | [21] |
| Envision | Multiple Features | Evolutionary conservation, physicochemical properties, structural context, sequence context, functional annotations, AAS characteristics | DMS data | DMS, ClinVar, Non-DMS functional assay data | Quantitative missense variant effect prediction using large-scale mutagenesis data | Input: protein sequence missense variant;  Output: Envision score | Web-based tool | Missense mutations | General-purpose (not disease-specific) | <https://envision.gs.washington.edu/shiny/envision_new/> | Python | Gradient Boosting | Most damaging, Most wild-type-like | Continuous scores, with higher scores indicating a greater likelihood of pathogenicity | ROC-AUC: ≈ 0.72 | [22] |
| EpiGePT | Multiple Features | DNA sequence, TF expression profile, TF motif scores, chromatin contact data | ENCODE DNase-seq, RNA-seq, ChIP-seq (H3K27ac, H3K4me1, etc.), HiChIP (HiChIPdb) | CRISPRi screens (Gasperini et al., Fulco et al.), ClinVar, eQTL (GTEx), COVID-19 GWAS | Transformer-based large language models (LLM) for predicting epigenomic signals, chromatin interactions, variant effect across cell types with TF context awareness and 3D genome modeling | Input: genomic regions transcription factor expression profile;  Output: predicted chromatin states | Web-based tool | Noncoding SNVs | Various diseases | <http://health.tsinghua.edu.cn/epigept/> | Python | Transformer (multi-task genomic language model) | Non-Causal, Causal | Not explicitly defined (depends on downstream classifier using LOS features) | ROC-AUC: ≈ 0.76 | [23] |
| EVE | Homology | MSA | UniRef | ClinVar | Captures the distribution of natural protein sequence variation to assess the impact of amino acid substitutions on fitness/pathogenicity | Input: gene symbol or UniProt ID;  Output: EVE score and classification | Web-based tool | Missense mutations | Various diseases associated | <https://evemodel.org/> | Python | Bayesian Variational Autoencoder (VAE) & Gaussian Mixture Model (GMM) | Benign, Uncertain, Pathogenic | >75% → high confidence | ROC-AUC: ≈ 0.91 | [24] |
| Evolutionary Action | Homology | MSA | - | E. coli RecA mutations, bacteriophage T4 lysozyme mutations, E. coli lac repressor mutations, TP53, GAA mutations, HIV-1 protease mutations, 1KGP Phase 1, TCGA | EA quantifies the effect of protein-coding variants on phenotype based on evolutionary conservation | Input: variant data;  Output: EA score | Web-based tool | Protein-coding variations, including missense mutations | Various diseases | <http://mammoth.bcm.tmc.edu/EvolutionaryAction> | - | Evolutionary Action equation based on perturbation theory and evolutionary conservation | Neutral, Deleterious; EA score ranges from 0 (neutral) to 100 (maximum impact), indicating the deleterious impact of mutations | - | ROC-AUC: 0.86–0.89 | [25] |
| FATHMM | Homology | MSA | - | VariBench, SwissVar, BRCA1, MSH2, MLH1, TP53 | Uses Hidden Markov Models (HMM) to predict the functional impact of missense mutations | Input: missense mutations;  Output: FATHMM score and classification | Web-based tool | Missense mutations | Various diseases | <http://fathmm.biocompute.org.uk/> | Python | HMM | T: Tolerated,  D: Deleterious | Weighted: –1.5  Unweighted: –3.0  >threshold → Tolerated;  <threshold → Deleterious | Accuracy: ≈ 0.86 | [26] |
| fitCons | Sequence-based | DNase-seq data, RNA-seq data, ChIP-seq data | - | eQTL enhancers | Integrates functional annotation with patterns of genetic variation to score genomic regions by their inferred level of selective constraint | Input: -;  Output: fitCons score and quantized covariates | Precomputed scores | Not variant-specific; scores genomic regions | General-purpose (not disease-specific) | <http://compgen.cshl.edu/fitCons/> | - | Inference of Natural Selection from Interspersed Genomically coHerent elemenTs (INSIGHT) model | fitCons scores reflect selective constraint rather than pathogenicity | - | - | [27] |
| GAVIN | Multiple Features | Minor allele frequency (MAF), impact prediction, CADD score | ExAC, SnpEff, CADD, VariBench tolerance DS7 (PhenCode database, IDbases, 18 individual LSDBs) | Clinical Genomics Database (CGD), VariBench tolerance DS7, MutationTaster2 benchmark set, ClinVar, UMCG | A multi-feature-based tool that integrates various prediction tool’s outputs and applies gene-specific thresholds for variant classification | Input VCF file, thresholds for gene classification;  Output: GAVIN classification | Web-based tool | Missense, frameshift, splice site, and other coding variants | Various diseases | <http://molgenis.org/gavin> | Java | Rule-based model (Gene-specific calibrations based on ExAC, SnpEff, and CADD score) | Benign, VUS, Pathogenic | CADD score thresholds:  <15 → Benign;  >15 → Pathogenic | Sensitivity: 0.91 | [28] |
| GENESIS | Multiple Features | Consensus amino acid, mutant amino acid, amino acid position, domain, evolutionary conservation, rate of evolution, signal-to-noise ratio, position-specific scoring matrix (PSSM) score | Model Creation Datasets (RYR2, KCNQ1, KCNH2, SCN5A) | ES VUS, ClinVar VUS, Patient VUS | A Python framework for gene-specific ML models to predict pathogenicity of VUS in genes associated with CPVT and LQTS | - | Source code | Missense mutations | Channelopathies (CPVT and LQTS) | <https://github.com/rachellea/medgenetics> | Python | Logistic Regression Model | Benign, Pathogenic | - | ROC-AUC: ≈ 0.87 | [29] |
| GeneT | Multiple Features | Genotype, phenotype | HGMD, OMIM, GPCards, 1KGP | Synthetic dataset from data used in training | Fine-tuned LLM for filtering and prioritization of causative variants in rare genetic diseases | Input: clinical phenotype;  Output: prioritized variant list | Web-based tool | SNVs, InDels | Rare diseases | <http://igenet.genomics.cn/> | - | Fine-tuned LLM (Qwen1.5, Mistral, Meta-LLaMA) | - | - | ROC-AUC: ≈ 0.9 | [30] |
| GERP++ | Homology | MSA, phylogenetic tree, neutral rate, scaling factor, RS scores, p-values for constrained elements | UCSC Genome Browser data | UCSC Genome Browser data | Predicts evolutionary constraint using rejected substitution scores (RS). It estimates site-specific evolutionary rates based on multiple alignments and phylogenetic trees | Input: MSA, phylogenetic tree;  Output: constrained element predictions | Stand-alone software | It does not classify variants (identifies regions under selective constraint, which could correlate with functional variants) | - | <http://mendel.stanford.edu/SidowLab/downloads/gerp/index.html> | - | Statistical Model | Focuses more on identifying constrained elements | - | - | [31] |
| GWAVA | Multiple Features | Open chromatin, TF binding, histone modifications, RNA Pol II binding, CpG islands, genome segmentation, conservation, human variation, genic context, sequence context | HGMD, 1KGP, Transcription Start Site (TSS)-distance matched SNVs | 10-fold cross-validation on training datasets | Predicts functional impact of noncoding variants using ML over diverse genomic and epigenomic features | Input: SNVs;  Output: GWAVA scores | Web-based tool & Stand-alone software | Noncoding SNVs | Various diseases | <https://www.sanger.ac.uk/tool/gwav>a | Python | RF | Non-functional, Functional | ≤0.5 → Non-functional;  >0.5 → Functional | ROC-AUC: 0.75–0.84 | [32] |
| INDELpred | Multiple Features | AF, indel length, gene-based scores, function-based scores | ClinVar | ClinVar, VKGL, HGMD, Clinical WGS data | Machine-learning-based predictive model for discerning pathogenic from benign indels | Input: annotated indels variants;  Output: INDELpred score and classification | Source code | InDels | General-purpose (not disease-specific) | <https://github.com/yilin-wei98/INDELpred> | Python | Gradient Boosting | Benign, Pathogenic | - | ROC-AUC: 0.97–0.99 | [33] |
| InMeRF | Ensemble | 34 rank scores | HGMD, dbSNP | VariBench, PredictSNP, SwissVar | Predicts pathogenicity of missense variants using individual modeling for each amino acid substitution | Input: missense;  Output: InMeRF score and classification | Web-based tool | Missense mutations | General-purpose (not disease-specific) | <https://www.med.nagoya-u.ac.jp/neurogenetics/InMeRF/> | Python | RF | Normal, Pathogenic | <0.5 → Normal;  ≥0.5 → Pathogenic | ROC-AUC: 0.79–0.96 | [34] |
| MAGPIE | Multiple Features | Conservation scores, protein structure, population frequency, regulatory annotations, functional scores, SIFT, PolyPhen-2, splicing features, transcript-level annotations | ClinVar, gnomAD | ClinVar, gnomAD | Predicts pathogenicity across multiple variant types, leveraging diverse biological features | Input: VCF file;  Output: MAGPIE score and classification | Web-based tool | Missense, synonymous, stop gain/loss, frameshift, inframe indel, splice region | General-purpose (not disease-specific | <http://tools.shenlab-genomics.org/tools/MAGPIE> | Python | Gradient Boosting | Benign, Pathogenic | <0.5 → Benign;  >0.5 → Pathogenic | ROC-AUC: 0.99 | [35] |
| MAPP | Homology | Physicochemical properties, MSA | - | LacI, T4 lysozyme, HIV protease, HIV RT, pyruvate kinase, G6PD, HBB, IARC p53 | Analyzes the impact of missense mutations on protein function by considering evolutionary conservation and physicochemical differences | Input: missense mutations;  Output: MAPP score | Stand-alone software | Missense mutations | General-purpose (not disease-specific) | <http://mendel.stanford.edu/SidowLab/downloads/MAPP/index.html> | - | Statistical model using physicochemical constraint violation analysis | Positive, Deleterious variants | No fixed threshold; ranking-based interpretation (Continuous score; higher means more likely to be deleterious) | Accuracy: 0.64–0.80 | [36] |
| M-CAP | Multiple Features | 9 predictors, 7 conservation scores, 298 alignment-based features | HGMD, ExAC | - | Predicts the pathogenicity of missense variants using a ML model trained on a variety of genomic features | Input: missense variants;  Output: M-CAP score and classification | Web-based tool | Missense mutations | Mendelian disorders | <http://bejerano.stanford.edu/mcap/> | - | Gradient Boosting | Benign, Pathogenic | <0.025 → Benign;  >0.025 → Pathogenic | ROC-AUC: ≈ 0.90 | [37] |
| MetaLR/ MetaSVM | Ensemble | 15 predictors, 3 conservation scores | UniProt | VariBench, CHARGE database | MetaLR and MetaSVM integrate scores from various prediction tools using logistic regression (MetaLR) and support vector machine (MetaSVM) models to predict variant pathogenicity | Input: nsSNVs;  Output: MetaLR and MetaSVM score | Precomputed scores through ANNOVAR and dbSNFP | Non-synonymous SNVs | Various diseases associated with genetic mutations | - | - | MetaLR; MetaSVM | Benign, Damaging | <0.5 → Benign;  >0.5 → Damaging | ROC-AUC: 0.92–0.94/  ROC-AUC: 0.91–0.93, | [38] |
| Meta-SNP | Ensemble | 4 predictors: PANTHER, PhD-SNP, SIFT, SNAP | SwissVar | SwissVar | Meta-SNP is a meta-predictor that combines predictions from SIFT, PolyPhen-2, MutationAssessor, and FATHMM to assess the pathogenicity of nsSNPs | Input: protein sequences;  Output: Meta-SNP score | Web-based tool | Non-synonymous SNPs | Various diseases associated with genetic mutations | <https://snps.biofold.org/meta-snp/> | - | RF | Polymorphic non-synonymous, Disease-related | <0.5 → Polymorphic non-synonymous;  >0.5 → Disease related | ROC-AUC: ≈ 0.87 | [39] |
| MISTIC | Multiple Features | Multi-ethnic MAF, conservation measures, functional measures, pathogenicity predictors | ClinVar, gnomAD | ClinVar, DoCM,  SweGen set, The WesternAsia | Predicts the deleteriousness of missense variants by integrating evolutionary, structural, and biochemical features | Input: missense variant;  Output: MISTIC score | Web-based tool | Missense mutations | Rare Mendelian disorders | <http://lbgi.fr/mistic> | Python | RF and Logistic Regression | Benign, Deleterious | <0.5 → Benign;  >0.5 → Deleterious | ROC-AUC: ≈ 0.95 | [40] |
| MMSplice | Multiple Features | Sequence-based features: exon, intron, splice site sequences; includes modular scores for acceptor site, donor site, exon, intron, junction regions | GENCODE, MPRA, Vex-seq, MaPSy, ClinVar | GENCODE, MPRA, Vex-seq, MaPSy, ClinVar | Modular DL framework that predicts the effect of genetic variants on splicing by modeling different splicing modules (acceptor, donor, exon, intron) | Input: genetic variant (VCF, GTF, fasta file);  Output: delta_logit_psi (splicing effect score), classification, and splicing efficiency per variant and exon | Source code & Stand-alone software | SNVs, indels | General-purpose (not disease-specific) | <https://github.com/gagneurlab/MMSplice>  <https://kipoi.org/models/MMSplice> | Python, Perl | CNN | Benign, Pathogenic | <-2 → Pathogenic;  -2≤ delta_logit_psi ≤2 → Benign;  >2 → Pathogenic | ROC-AUC: ≈ 0.94 | [41] |
| MPC | Multiple Features | Missense depletion (γ), missense badness, PolyPhen-2, BLOSUM, Grantham scores | ClinVar, ExAC | 5620 neurodevelopmental disorder cases and 2078 controls | Evaluates the deleteriousness of missense variants by incorporating regional missense constraint and specific AAS impact | Input: missense variant;  Output: MPC score | Precomputed scores | Missense mutations | Neurodevelopmental disorders | <https://grr.iossifovlab.com/hg19/scores/MPC/> | - | Logistic Regression | MPC score ranges from 0 to 5, with larger numbers indicating increased deleteriousness | - | Specificity: ≈ 0.96 | [42] |
| MutationAssessor | Homology | MSA, entropy change, evolutionary conservation | - | HUMSAVAR, COSMIC UniProt, IARC p53, COSMIC | MutationAssessor predicts the functional impact of protein mutations based on evolutionary conservation and the structure-function relationship | Input: amino acid substitutions;  Output: functional impact scores (FI) and classification | Web-based tool | Non-synonymous SNPs | Cancer and other diseases with genetic underpinnings | <http://mutationassessor.org/> | - | Entropy of MSA | L: Low,  M: Medium,  H: high, N: Neutral | 0.8< x ≤1.9 → Low;  1.9< x ≤ 3.5 → Medium; x>3.5→ High | ROC-AUC: ≈ 0.86 | [43] |
| MutationTaster/  MutationTaster2 | Multiple Features | Evolutionary conservation, splice site changes, protein features, changes to regulatory elements, amino acid substitutions, known disease mutations | HGMD, ClinVar, 1KGP, HapMap | Curated ClinVar, HGMD datasets | MutationTaster and MutationTaster2 predict the pathogenicity of genetic variants based on sequence annotations, evolutionary conservation, and in silico predictions of variant effects | Input: genetic variants;  Output: MutationTaster/  MutationTaster2 classification, disease-causing potential, conservation, splice site changes, etc | Web-based tool | Missense, nonsense, synonymous, intronic, UTR, indels | General-purpose (not disease-specific) | [http://www.mutationtaster.org](http://www.mutationtaster.org/) | - | Naive Bayes classifier | Benign, Deleterious | - | Accuracy: ≈ 0.92 | [44, 45] |
| MutFormer | Multiple Features | Protein sequences (both reference and variant sequences), 27 features from DBNSFPv3 | HGMD, gnomAD | Meta_SVM_LR_set_1, Meta_SVM_LR_set_2, Meta_SVM_LR_set_3, Varibench_PPARG, Varibench_TP53 | Transformer-based DL model, uses convolutions to enhance sequence representation and attention mechanisms to predict the deleteriousness of variants in proteins | Input: protein sequence;  Output: MutFormer score and classification | Precomputed scores | Missense mutations | General-purpose (not disease-specific) | <http://www.openbioinformatics.org/mutformer/hg19_MutFormer.zip> | Python | Transformer architecture and BERT | Benign, Deleterious | - | ROC-AUC: 0.925–0.970 | [46] |
| MutPred/MutPred2 | Multiple Features | Structural properties, functional properties | CANCER, KINASE, HGMD, Swissprot disease-causing (SPd), SwissProt polymorphic (SPp), HGMD, SwissVar, dbSNP and inter-species pairwise alignment | CANCER, KINASE, HGMD, gnomAD | MutPred and MutPred2 predict the pathogenicity of missense mutations, provide insights into potential molecular mechanisms by analyzing various structural and functional properties of proteins | Input: protein sequences, amino acid substitution;  Output: MutPred/ MutPred2 score and classification | Web-based tool | Missense mutations | General-purpose (not disease-specific) | <http://mutpred2.mutdb.org> | - | RF | Neutral polymorphisms, Disease-associated mutations / Neutral, Pathogenic | <0.5 → Neutral;  >0.5 → Disease-associated mutations/Pathogenic | ROC-AUC: ≈ 0.82/ ROC-AUC: ≈ 0.87 | [47, 48] |
| MutScore | Ensemble | 5 Predictors: SIFT, SIFT4G, LRT, PROVEAN, GERP++ RS, 9 conservation scores | ClinVar | ClinVar, HGMD, DoCM | Integrates positional information and amino acid change likelihood with existing unsupervised features to predict the pathogenicity of missense variants. It aims to improve classification of VUSs and CIs | Input: gene, isoform, type of DNA or protein change;  Output: MutScore score and conservation profile across selected gene region | Web-based tool | Missense variants, including PLP, BLB, VUS, and CIs | Hereditary Conditions, Cancer | <https://mutscore-wgt7hvakhq-ew.a.run.app/> | R, Perl | RF | Pathogenic, Likely Pathogenic, VUS, Likely Benign, Benign | <0.140 → Likely Benign;  >0.730 → Likely Pathogenic | ROC-AUC: 0.91 - 0.96 | [49] |
| MVP | Multiple Features | Features computed per base pair, features computed per local context, features computed per gene, deleteriousness scores | HGMD, UniProt, ClinVar, DiscovEHR, human-derived variants | VariBench, DiscovEHR Cancer hotspot, DiscovEHR, De novo datasets (cases: ASD, CHD and controls: Simons Simplex Collection unaffected siblings) | Predicts the pathogenicity of missense variants using a deep residual network model, trained separately on genes based on their LoF intolerance | Input: missense variant data;  Output: MVP score | Precomputed scores | Missense mutations | Genetic Diseases, cancer, Congenital  Heart Disease (CHD), Autism  Spectrum Disorder (ASD) | <https://www.dropbox.com/s/d9we7gx42b7yatg/MVP_score_hg19.txt.bz2?dl=0> | Python | Deep Residual Neural Network (ResNet) | Pathogenic, Benign | Constrained genes:  >0.7 → Pathogenic;  Non-constrained genes:  >0.75 → Pathogenic | ROC-AUC: 0.85 – 0.96 | [50] |
| NBDriver | Multiple Features | Sequence, structural, conservation, functional annotation, predictive scores | Brown et al. dataset | Martelotto et al. dataset, CMC, The Catalog of Validated Oncogenic Mutations, COSMIC | Uses neighborhood sequences and descriptive genomic features to distinguish between driver and passenger mutations in cancer genomes | Input: -;  Output: NBDriver classification | Source code | Missense mutations | Cancer | <https://github.com/RamanLab/NBDriver> | Phyton, R | Three classifiers: RF classifier, the extra trees (ET) classifier (extreme RF), and the generative KDE classifier | Passenger, Driver | - | Accuracy: ≈ 0.89 | [51] |
| PANTHER | Homology | MSA | - | HGMD, dbSNP, SNPs sampled from healthy individuals | The tool uses HMMs for classifying protein families and predicting the functional likelihood of amino acid substitutions | Input: protein sequence, amino acid substitutions;  Output: PANTHER score and classification | Web-based tool | Nonsynonymous coding SNP | General-purpose (not disease-specific) | <http://pantherdb.org/> | - | HMMs, statistical models, PSEP (position-specific evolutionary preservation) | Probably benign, Possibly damaging,  Probably damaging | time<200my → Probably benign;  time>200my, → Possibly damaging;  time> 50my → Probably damaging | - | [52] |
| parSMURF | Multiple Features | Genomic conservation features, sequence features, population features, chromatin effect predictions, synthetic features, regulatory and transcriptional annotation | Mendelian dataset, GWAS dataset, synthetic datasets | - | ML framework combining SMOTE, under-sampling, and RF to detect rare pathogenic SNVs in highly imbalanced datasets | Input: -;  Output: parSMURF score and classification | Source code | SNVs | General-purpose (not disease-specific) | <https://github.com/AnacletoLAB/parSMURF> | C++, Python | RF | Negative, Positive | - | ROC-AUC: ≈ 0.99 | [53] |
| PdmIRD | Multiple Features | Population frequencies in multiple large-scale cohorts, multiple existing pathogenicity prediction tools, evolutionary conservations in multi-species alignment, gene mutation intolerances | RetNet, ClinVar, HGMD, UniProt, VariSNP | HGMD, VariSNP, Uniprot | A model specifically designed to predict the pathogenicity of missense mutations in IRDs, showing superior performance compared to pan-disease tools and eye disease-specific tools | Input: variant data;  Output: PdmIRD classification and graph plot (for one mutation) | Web-based tool | Missense Mutations | Inherited Retinal Diseases (IRDs) | <https://zbshiny.shinyapps.io/IRDmis/> | R, Perl | Conditional RF | Benign, Pathogenic | <0.5 → Benign;  ≥0.5 → Pathogenic | ROC-AUC: ≈ 0.91 – 0.98 | [54] |
| PhD-SNP | Sequence-based | Protein Sequence | Swiss-Prot, HumVar, HumVarProf | HumVar, HumVarProf,  NewHumVar | Predicts whether a given nsSNP is disease-related or neutral based on protein sequence or sequence profile information | Input: SNP details;  Output: reliability index (RI) score and classification | Web-based tool | Non-synonymous SNPs | General-purpose (not disease-specific) | <http://snps.biofold.org/phd-snp/phd-snp.html> | - | SVM | N: Neutral,  D: Disease | <0.5 → Neutral;  >0.5 → Disease | ROC-AUC: ≈ 0.79 | [55] |
| PolyPhen-HCM | Homology | PolyPhen-2 Prediction, MrBayes Substitution Rate Score, Coiled-Coil Score, protein structure comparison score | 74 curated variants from literatures and manually classified by Laboratory for Molecular Medicine standard variant-assessment pipeline | The same dataset used for training | A specialized predictor that integrates phylogenetic and structural information with a probabilistic classifier, designed for clinical use in HCM variant assessment | Input: -;  Output: PolyPhen-HCM classification | Source code | Missense mutations | Hypertrophic Cardiomyopathy (HCM) | <https://github.com/RalphEST/my-polyphen-hcm> | Python | Naïve Bayes | Benign, Pathogenic, No Call | - | Accuracy: 0.92 | [56] |
| PolyPhen/  PolyPhen-2 | Homology/Multiple Features | Sequence conservation, structural features, amino acid properties, protein domain annotations, profile alignment scores | UniProt & Swiss-Prot/ HumDiv, HumVar datasets) | HumDiv, HumVar | PolyPhen and PolyPhen-2 predicts the impact of amino acid substitutions on protein structure and function | Input: missense mutations;  Output: PolyPhen score and classification | Web-based tool | Missense mutations | General-purpose (not disease-specific) | <http://genetics.bwh.harvard.edu/pph2/> | - | Rule Based/ Naïve Bayes | Benign,  Possibly damaging,  Probably damaging | 0.0–0.15 → Benign;  -0.15–0.85 → Possibly damaging;  >0.85 → Probably damaging | ROC-AUC: ≈ 0.83 | [57, 58] |
| PON-P3 | Multiple Features | Protein and gene features, variation features, structural features | ClinVar, LOVD | ClinVar, LOVD | A ML tool designed to assess the pathogenicity of amino acid substitutions in human MANE-specific proteins | Input: protein, transcript and genomic variation;  Output: PON-P3 score and classification | Web-based tool | Missense mutations | General-purpose (not disease-specific) | <https://structure.bmc.lu.se/PON-P3/> | - | LightGBM | Pathogenic, Neutral, VUS | <0.5 → Neutral;  ≥0.5 → Pathogenic | Accuracy: ≈ 0.94 | [59] |
| PredictSNP/  PredictSNP2 | Ensemble | 8 predictors / 6 predictors | UniProt, PON-P/ ClinVar, GWAS catalog, COSMIC, VariSNP | PMD, experimental studies/Mendelian disease and cancer driver variant | Integrates predictions from multiple tools to provide a consensus prediction of the effects of mutations on protein function | Input: protein sequence and mutations;  Output: PredictSNP/  PredictSNP2 score and classification | Web-based tool | Non-synonymous SNPs | Various genetic disorders and diseases linked to missense mutations | PredictSNP:  <http://loschmidt.chemi.muni.cz/predictsnp>;  PredictSNP2:  <https://loschmidt.chemi.muni.cz/predictsnp2/> | - | RF | Neutral, Deleterious | - | ROC-AUC: 0.80 - 0.83/ ROC-AUC: ≈ 0.83 | [60, 61] |
| PrimateAI | Multiple Features | 51‑AA sequence flanking variant, orthologous MSA from six non‑human primates, predicted secondary‑structure, solvent accessibility sub‑networks | ExAC/gnomAD + chimpanzee, bonobo, gorilla, orangutan, rhesus, marmoset | Independent ClinVar/HGMD, de novo DDD & SSC sets, BRCA1 functional assay data | Uses a DL model to predict the pathogenicity of coding variants based on evolutionary data from primates | Input: variants data;  Output: PrimateAI score | Precomputed scores | Missense mutations | General-purpose (not disease-specific) | <https://basespace.illumina.com/s/cPgCSmecvhb4> | Python | Neural Network | 0 (Less pathogenic) to 1 (More pathogenic) | ≥0.803 → Pathogenic | ROC-AUC: ≈ 0.73 | [62] |
| PromoterAI | Multiple Features | 4,096-bp DNA sequence centered on the TSS (one-hot 4 × 4,096) + optional cell-type embedding | GTEx v8, ENCODE CAGE, Roadmap Epigenomics RNA-seq, FANTOM5 CAGE | Genomics England (GEL) cohort, UK Biobank (UKBB), GTEx, ClinVar, gnomAD, Massively Parallel Reporter Assay (MPRA) datasets | Predicts whether a promoter mutation increases, decreases, or leaves unchanged gene expression; models core-promoter motifs, TF binding, and local chromatin context | Input: Genetic variant (in promoter region);  Output: PromoterAI score and classification | Source code & Precomputed scores | Non-coding promoter variants within +/- 500 bp of the TSS | Rare diseases | <https://github.com/Illumina/PromoterAI> | Python | Ensemble of two CNN (MetaFormer architecture) | Under-expression, Overexpression, Null (No effect) | >0.5 or <-0.5 → strong effects  In rare disease analysis  < -0.05 → enrichment | ROC-AUC: ≈ 0.76 | [63] |
| PROVEAN | Homology | Sequences alignment,  sequence-based predictions of structure features | - | LacI, TP53, ABCA1 UniProtKB/Swiss-Prot | Developed to predict whether a protein sequence variation affects protein function | Input: a protein sequence and amino acid variants;  Output: PROVEAN score, classification, and number of homologous sequences used | Web-based tool | Single or multiple amino acid substitutions, and in-frame insertions and deletions | General-purpose (not disease-specific) | <http://provean.jcvi.org./index.php> | - | SVM | Neutral, Deleterious | >-2.5 → Neutral;  ≤-2.5→ Deleterious | ROC-AUC: ≈ 0.85 | [64] |
| REGatta | Multiple Features | Regional location of variant within gene, pathogenic variant clustering in ClinVar; clinical outcome data | UKBB | UKBB | Assigns regional risk levels based on enrichment of pathogenic variants and clinical outcomes | Input: -;  Output: REGatta classification | Source code | Missense mutations | General-purpose (not disease-specific | <https://github.com/cassalab/regatta> | Python | Cox Regression Model | HRR, LRR | ≥1.15 → HRR;  ≤0.85 → LRR | - | [65] |
| REVEL | Ensemble | Pathogenicity predictions from 18 individual scores, 8 conservation scores, 10 functional scores | HGMD, ESP, ARIC, 1KGP | ClinVar and SwissVar | An ensemble method that integrates multiple existing tools to predict the pathogenicity of rare missense variants | Input: missense variant;  Output: REVEL score | Precomputed scores | Missense mutations | General-purpose (not disease-specific) | <https://sites.google.com/site/revelgenomics/> | - | RF | Benign, Pathogenic | >0.75 → Pathogenic | ROC-AUC: 0.90 - 0.91 | [66] |
| SIFT/SIFT4G | Homology | MSA | - | lacI, lysozyme, HIV protease, HumDiv, HumVar | Predicts the effects of non-synonymous mutations on protein function based on sequence conservation. SIFT4G is an optimized version for genome-scale analysis | Input: amino acid substitutions;  Output: SIFT score and classification | Web-based tool | Non-synonymous SNPs | General-purpose (not disease-specific) | SIFT: <https://sift.bii.a-star.edu.sg/>;  SIFT4G: <https://sift.bii.a-star.edu.sg/sift4g/> | Perl (SIFT); C++ (SIFT4G) | Sequence homology-based prediction | Tolerated, Deleterious | >0.05 → Tolerated;  <0.05 → Deleterious | ROC-AUC: 0.80 – 0.82/  Accuracy: 0.8 – 0.76 | [67, 68] |
| SNAP/SNAP2 | Multiple Features | Conservation, structure prediction | PMD, SwissProt, OMIM, HumVar | lacI, lysozyme, HIV protease, Melanocortin-4 | SNAP and SNAP2 predict the effects of nsSNPs on protein function using NNs and a variety of sequence and structural features | Input: AAS variants; Output: SNAP/SNAP2 score and classification | Web-based tool | Non-synonymous SNPs | General-purpose (not disease-specific) | SNAP: not available;  SNAP2: <http://rostlab.org/services/snap2/> | - | NN | Neutral;  Non-neutral/  Neutral, Effect | SNAP:  <0 → Neutral;  ≥0 → Non-neutral,  SNAP2:  < -0.05 → Neutral;  >-0.05 → Effect | ROC-AUC: ≈ 0.905 | [69, 70] |
| SNPs&GO | Multiple Features | Sequence alignment, prediction data provided by the PANTHER classification system, functional-based log-odds score calculated considering the GO classification | Swiss-Prot | Gene Ontology (GO) | Prediction of single point protein mutations likely to be involved in the insurgence of diseases in humans | Input: Uniprot accession number, mutation position, wild-type residue, substituting residue;  Output: SNPs&GO classification, reliability index | Web-based tool | Non-synonymous SNPs | General-purpose (not disease-specific) | <http://snps-and-go.biocomp.unibo.it/snps-and-go/> | - | SVM | Neutral, Disease | ≤0.5 → Neutral;  >0.5 → Disease | Accuracy: ≈ 0.82 | [71] |
| SPANR | Multiple Features | Sequence features extracted from exon/intron boundaries, including exon/intron lengths, splice site signals, splicing factor motifs, 1- to 3-mer frequencies, retrovirus repeats, nucleosome positioning, RNA secondary structures, and others | 16 normal tissues from human BodyMap, RNA-seq data (GSE30611) | RNA-seq, RT-PCR, RBP binding, MBNL knockdown, dbSNP135, ANNOVAR, HGMD | A tool designed to predict the impact of genetic variants (SNVs) on RNA splicing, with the goal of identifying variants that influence exon inclusion (Ψ) | Input: DNA sequence (exonic/intronic regions);  Output: predicted percent spliced-in (Ψ) values for each exon | Web-based tool | Intronic/exonic SNVs, disease mutations | Disease-related mutations affecting splicing (e.g., spinal muscular atrophy (SMA), hereditary nonpolyposis colorectal cancer (NPCC), ASD) | <http://tools.genes.toronto.edu/> | Python | Bayesian Model | - | - | ROC-AUC: ≈ 0.95 | [72] |
| SpliceAI | Sequence-based | 10,000 bp surrounding genomic sequence | GENCODE, GTEx | RNA-seq (Simons Simplex autism cohort), ExAC/gnomAD (population depletion), DDD + ASD de novo variant burden | DL model that predicts splice donor and acceptor probability changes at nucleotide resolution, capturing both nearby and distal sequence features | Input: variants data;  Output: SpliceAI score | Source code & Stand-alone software | SNVs, rare noncoding variants | Mendelian disease, neurodevelopmental disorders, cancer | <https://github.com/Illumina/SpliceAI> | Python | ResNet | Benign splice effect, Pathogenic | ≥0.5 → high confidence gain/loss  ≥0.1 → permissive | PR-AUC: ≈ 0.90 | [73] |
| SpTransformer | Multiple Features | DNA sequence surrounding variant, splice junction signals, RNA-seq based features | GTEx, RNA-seq data, ClinVar | GTEx, RNA-seq data, ClinVar | Predicts tissue-specific splicing alterations from pre-mRNA sequences | Input: variant position with flanking sequence;  Output: ΔSplice score | Source code & Stand-alone software | SNVs affecting splicing regions | General-purpose (not disease-specific) | <https://github.com/ShenLab-Genomics/SpliceTransformer> | Python | Transformer | Non Splice-Altering, Splice-Altering | ΔSplice ≤0.27 → Non Splice-Altering;  ΔSplice >0.27 → Splice-Altering | Accuracy: ≈85%, | [74] |
| SQUIRLS | Multiple Features | 15 interpretable features, including information content scores, sequence context around splice sites | Medical literature, ClinVar | Medical literature, ClinVar | Uses interpretable biological features and ML to prioritize non-canonical splice variants based on sequence context and information content | Input: variant sequences;  Output: SQUIRLS score | Source code, Stand-alone software & Precomputed scores | Non-canonical splice variants | General-purpose (not disease-specific) | <https://github.com/TheJacksonLaboratory/Squirls> | Python | RF + Logistic Regression | Neutral, Pathogenic | - | ROC-AUC: ≈ 0.97 | [75] |
| VariPred | Multiple Features | Wild-type and mutant sequences, target mutated position, wildtype amino acid, mutant amino acid | ClinVar, gnomAD | ClinVar, SwissVarFilteredMix, VaribenchSelectPure | Combines residue embedding and log-likelihood ratio (LLR) from ESM-1b with a twin-network to predict pathogenicity of missense variants | Input: protein sequence;  Output: VariPred score | Source code | Missense mutations | General-purpose (not disease-specific) | <https://github.com/wlin16/VariPred> | Python | Twin-network + ESM-1b PLM + LLR | Benign, Pathogenic | <0.2 → Benign;  >0.2 → Pathogenic | ROC-AUC: ≈ 0.928 | [76] |
| VEST | Multiple Features | 86 features of SNVBox | HGMD, ESP | Swissprot, 1KGP, Complete Genomics diversity panel | Predicts the impact of genetic variants on protein function using a RF classifier trained on a large dataset of known variants | Input: CRAVAT & VCF format;  Output: VEST score | Stand-alone software | Missense mutations | General-purpose (not disease-specific) | <http://wiki.chasmsoftware.org/> | - | RF | Neutral, Functional (disease-related) | ≤0.5 → Neutral;  >0.5 → Functional (disease-related) | ROC-AUC: 0.91 – 0.92 | [77] |
| VIPPID | Multiple Features | 85 features, including AA, exonic, protein structural, conservation, 20 pre-existing prediction tools | RAPID, HGMD ClinVar, gnomAD | Independent mutation set from 26 verified pathogenic or likely pathogenic variants from a large PID cohort (n = 1318 patients)  - 39 in-house verified PID pathogenic variants | Predict the pathogenicity of SNVs in genes associated with Primary Immunodeficiency Disorders (PIDs). It includes both general and gene-specific sub-models to improve accuracy, especially for genes with many known variants | Input: variants data  Output: VIPPID score and classification | Web-based tool | Missense mutations | Primary Immunodeficiency Diseases (PIDs) | <https://mylab.shinyapps.io/VIPPID/> | R | Conditional Inference Forest | Likely Benign, Benign, Likely Pathogenic, Pathogenic | - | ROC-AUC: 0.89 – 0.91 | [78] |
| VIPUR | Multiple Features | 5 sequence-based features from PSIBLAST, 17 from Rosetta ddg monomer, 83 from Rosetta FastRelax, 1 from PROBE | HumDIv, UniProt, PDB, Swiss Model, ModBase | - | Uses the Rosetta suite to predict the structural and functional impact of protein variants, integrating various data sources for robust classification | Input: native variant, protein variant;  Output: VIPUR score, classification and 3D models of the variant protein | Web-based tool | Protein | Mutations associated with inflammation, diabetes, and ASD | <https://osf.io/bd2h4> | Python, with Rosetta components | Sparse Logistic Regression | Neutral,  Deleterious | >0.5 → Deleterious;  >0.7 → Strongly Deleterious | ROC-AUC: ≈ 0.83 | [79] |

1. Type:

This column indicates the primary methodological category used by each prediction tool.

- "Homology" refers to methods based on sequence conservation, such as multiple sequence alignments or evolutionary models.
- "Sequence-based" refers to approaches that primarily rely on the raw nucleotide or amino acid sequence
- "Multiple Features" includes tools that integrate a broad range of genomic annotations like conservation scores, AF, and functional predictions.
- "Ensemble" refers to models that combine outputs from multiple predictive tools or annotations.
- "Protein structure-based" describes methods that utilize 3D structural information, either experimentally determined or predicted.

1. Output:

Describes the result returned by each tool. “Classification” refers to the categorical label (e.g., Benign, Pathogenic) assigned based on internal thresholds, while “score” indicates a numerical value representing the predicted likelihood of pathogenicity. When both are present, the classification is derived from the score using a tool-specific decision threshold.

1. Reported Performance: Refers to the predictive accuracy reported in the original publication for each tool. These metrics are study-specific and may not be directly comparable across tools due to differences in validation datasets and the lack of a universal benchmarking standard.

References

[1] J. Luo *et al.*, "Assessing concordance among human, in silico predictions and functional assays on genetic variant classification," *Bioinformatics,* vol. 35, no. 24, pp. 5163-5170, 2019, doi: 10.1093/bioinformatics/btz442.

[2] Ž. Avsec *et al.*, "AlphaGenome: advancing regulatory variant effect prediction with a unified DNA sequence model," *bioRxiv,* p. 2025.06.25.661532, 2025, doi: 10.1101/2025.06.25.661532.

[3] S. V. Tavtigian, "Comprehensive statistical study of 452 BRCA1 missense substitutions with classification of eight recurrent substitutions as neutral," *Journal of Medical Genetics,* vol. 43, no. 4, pp. 295-305, 2005, doi: 10.1136/jmg.2005.033878.

[4] J. Cheng *et al.*, "Accurate proteome-wide missense variant effect prediction with AlphaMissense," *Science,* vol. 381, no. 6664, p. eadg7492, Sep 22 2023, doi: 10.1126/science.adg7492.

[5] D. R. Kelley, Y. A. Reshef, M. Bileschi, D. Belanger, C. Y. McLean, and J. Snoek, "Sequential regulatory activity prediction across chromosomes with convolutional neural networks," (in eng), *Genome Res,* vol. 28, no. 5, pp. 739-750, May 2018, doi: 10.1101/gr.227819.117.

[6] M. Kircher, D. M. Witten, P. Jain, B. J. O'Roak, G. M. Cooper, and J. Shendure, "A general framework for estimating the relative pathogenicity of human genetic variants," *Nat Genet,* vol. 46, no. 3, pp. 310-5, Mar 2014, doi: 10.1038/ng.2892.

[7] J. S. Kaminker, Y. Zhang, C. Watanabe, and Z. Zhang, "CanPredict: a computational tool for predicting cancer-associated missense mutations," *Nucleic Acids Research,* vol. 35, no. Web Server, pp. W595-W598, 2007, doi: 10.1093/nar/gkm405.

[8] S. Li *et al.*, "CAPICE: a computational method for Consequence-Agnostic Pathogenicity Interpretation of Clinical Exome variations," *Genome Medicine,* vol. 12, no. 1, 2020, doi: 10.1186/s13073-020-00775-w.

[9] X. Zhang *et al.*, "Disease-specific variant pathogenicity prediction significantly improves variant interpretation in inherited cardiac conditions," *Genetics in Medicine,* vol. 23, no. 1, pp. 69-79, 2021/01/01 2021, doi: 10.1038/s41436-020-00972-3.

[10] N. Alirezaie, K. D. Kernohan, T. Hartley, J. Majewski, and T. D. Hocking, "ClinPred: Prediction Tool to Identify Disease-Relevant Nonsynonymous Single-Nucleotide Variants," *The American Journal of Human Genetics,* vol. 103, no. 4, pp. 474-483, 2018, doi: 10.1016/j.ajhg.2018.08.005.

[11] A. González-Pérez and N. López-Bigas, "Improving the Assessment of the Outcome of Nonsynonymous SNVs with a Consensus Deleteriousness Score, Condel," *The American Journal of Human Genetics,* vol. 88, no. 4, pp. 440-449, 2011, doi: 10.1016/j.ajhg.2011.03.004.

[12] D. Quang, Y. Chen, and X. Xie, "DANN: a deep learning approach for annotating the pathogenicity of genetic variants," *Bioinformatics,* vol. 31, no. 5, pp. 761-763, 2015, doi: 10.1093/bioinformatics/btu703.

[13] J. Zhou and O. G. Troyanskaya, "Predicting effects of noncoding variants with deep learning-based sequence model," *Nat Methods,* vol. 12, no. 10, pp. 931-4, Oct 2015, doi: 10.1038/nmeth.3547.

[14] D. Munro, M. Singh, and J. Xu, "DeMaSk: a deep mutational scanning substitution matrix and its use for variant impact prediction," *Bioinformatics,* vol. 36, no. 22-23, pp. 5322-5329, 2020, doi: 10.1093/bioinformatics/btaa1030.

[15] D. Raimondi, A. M. Gazzo, M. Rooman, T. Lenaerts, and W. F. Vranken, "Multilevel biological characterization of exomic variants at the protein level significantly improves the identification of their deleterious effects," *Bioinformatics,* vol. 32, no. 12, pp. 1797-1804, 2016, doi: 10.1093/bioinformatics/btw094.

[16] D. Raimondi *et al.*, "DEOGEN2: prediction and interactive visualization of single amino acid variant deleteriousness in human proteins," *Nucleic Acids Research,* vol. 45, no. W1, pp. W201-W206, 2017, doi: 10.1093/nar/gkx390.

[17] F. Bu *et al.*, "DVPred: a disease-specific prediction tool for variant pathogenicity classification for hearing loss," (in eng), *Hum Genet,* vol. 141, no. 3-4, pp. 401-411, Apr 2022, doi: 10.1007/s00439-022-02440-1.

[18] S. Zeng, J. Yang, B. H.-Y. Chung, Y. L. Lau, and W. Yang, "EFIN: predicting the functional impact of nonsynonymous single nucleotide polymorphisms in human genome."

[19] I. Ionita-Laza, K. McCallum, B. Xu, and J. D. Buxbaum, "A spectral approach integrating functional genomic annotations for coding and noncoding variants," *Nature Genetics,* vol. 48, no. 2, pp. 214-220, 2016, doi: 10.1038/ng.3477.

[20] Ž. Avsec *et al.*, "Effective gene expression prediction from sequence by integrating long-range interactions," *Nature Methods,* vol. 18, no. 10, pp. 1196-1203, 2021/10/01 2021, doi: 10.1038/s41592-021-01252-x.

[21] A. G. de Brevern, H. Zhou, M. Gao, and J. Skolnick, "ENTPRISE: An Algorithm for Predicting Human Disease-Associated Amino Acid Substitutions from Sequence Entropy and Predicted Protein Structures," *Plos One,* vol. 11, no. 3, 2016, doi: 10.1371/journal.pone.0150965.

[22] V. E. Gray, R. J. Hause, J. Luebeck, J. Shendure, and D. M. Fowler, "Quantitative Missense Variant Effect Prediction Using Large-Scale Mutagenesis Data," *Cell Systems,* vol. 6, no. 1, pp. 116-124.e3, 2018, doi: 10.1016/j.cels.2017.11.003.

[23] Z. Gao, Q. Liu, W. Zeng, R. Jiang, and W. H. Wong, "EpiGePT: a Pretrained Transformer model for epigenomics," *bioRxiv,* p. 2023.07.15.549134, 2024, doi: 10.1101/2023.07.15.549134.

[24] J. Frazer *et al.*, "Disease variant prediction with deep generative models of evolutionary data," *Nature,* vol. 599, no. 7883, pp. 91-95, Nov 2021, doi: 10.1038/s41586-021-04043-8.

[25] P. Katsonis and O. Lichtarge, "A formal perturbation equation between genotype and phenotype determines the Evolutionary Action of protein-coding variations on fitness," *Genome Research,* vol. 24, no. 12, pp. 2050-2058, 2014, doi: 10.1101/gr.176214.114.

[26] H. A. Shihab *et al.*, "Predicting the Functional, Molecular, and Phenotypic Consequences of Amino Acid Substitutions using Hidden Markov Models," *Human Mutation,* vol. 34, no. 1, pp. 57-65, 2012, doi: 10.1002/humu.22225.

[27] B. Gulko, M. J. Hubisz, I. Gronau, and A. Siepel, "A method for calculating probabilities of fitness consequences for point mutations across the human genome," *Nature Genetics,* vol. 47, no. 3, pp. 276-283, 2015, doi: 10.1038/ng.3196.

[28] K. J. van der Velde *et al.*, "GAVIN: Gene-Aware Variant INterpretation for medical sequencing," *Genome Biology,* vol. 18, no. 1, p. 6, 2017/01/16 2017, doi: 10.1186/s13059-016-1141-7.

[29] R. L. Draelos *et al.*, "GENESIS: Gene-Specific Machine Learning Models for Variants of Uncertain Significance Found in Catecholaminergic Polymorphic Ventricular Tachycardia and Long QT Syndrome-Associated Genes," *Circulation: Arrhythmia and Electrophysiology,* vol. 15, no. 4, 2022, doi: 10.1161/circep.121.010326.

[30] L. Liang *et al.*, "Genetic Transformer: An Innovative Large Language Model Driven Approach for Rapid and Accurate Identification of Causative Variants in Rare Genetic Diseases," *medRxiv,* p. 2024.07.18.24310666, 2024, doi: 10.1101/2024.07.18.24310666.

[31] E. V. Davydov, D. L. Goode, M. Sirota, G. M. Cooper, A. Sidow, and S. Batzoglou, "Identifying a high fraction of the human genome to be under selective constraint using GERP++," (in eng), *PLoS Comput Biol,* vol. 6, no. 12, p. e1001025, Dec 2 2010, doi: 10.1371/journal.pcbi.1001025.

[32] G. R. Ritchie, I. Dunham, E. Zeggini, and P. Flicek, "Functional annotation of noncoding sequence variants," (in eng), *Nat Methods,* vol. 11, no. 3, pp. 294-6, Mar 2014, doi: 10.1038/nmeth.2832.

[33] Y. Wei *et al.*, "INDELpred: Improving the prediction and interpretation of indel pathogenicity within the clinical genome," *HGG Adv,* vol. 5, no. 4, p. 100325, Oct 10 2024, doi: 10.1016/j.xhgg.2024.100325.

[34] J.-i. Takeda *et al.*, "InMeRF: prediction of pathogenicity of missense variants by individual modeling for each amino acid substitution," *NAR Genomics and Bioinformatics,* vol. 2, no. 2, 2020, doi: 10.1093/nargab/lqaa038.

[35] Y. Liu, T. Zhang, N. You, S. Wu, and N. Shen, "MAGPIE: accurate pathogenic prediction for multiple variant types using machine learning approach," *Genome Medicine,* vol. 16, no. 1, p. 3, 2024/01/08 2024, doi: 10.1186/s13073-023-01274-4.

[36] E. A. Stone and A. Sidow, "Physicochemical constraint violation by missense substitutions mediates impairment of protein function and disease severity," *Genome Research,* vol. 15, no. 7, pp. 978-986, 2005, doi: 10.1101/gr.3804205.

[37] K. A. Jagadeesh *et al.*, "M-CAP eliminates a majority of variants of uncertain significance in clinical exomes at high sensitivity," *Nature Genetics,* vol. 48, no. 12, pp. 1581-1586, 2016, doi: 10.1038/ng.3703.

[38] C. Dong *et al.*, "Comparison and integration of deleteriousness prediction methods for nonsynonymous SNVs in whole exome sequencing studies," *Human Molecular Genetics,* vol. 24, no. 8, pp. 2125-2137, 2014, doi: 10.1093/hmg/ddu733.

[39] E. Capriotti, R. B. Altman, and Y. Bromberg, "Collective judgment predicts disease-associated single nucleotide variants," (in eng), *BMC Genomics,* vol. 14 Suppl 3, no. Suppl 3, p. S2, 2013, doi: 10.1186/1471-2164-14-s3-s2.

[40] M. A. Andrade-Navarro *et al.*, "MISTIC: A prediction tool to reveal disease-relevant deleterious missense variants," *Plos One,* vol. 15, no. 7, 2020, doi: 10.1371/journal.pone.0236962.

[41] J. Cheng *et al.*, "MMSplice: modular modeling improves the predictions of genetic variant effects on splicing," *Genome Biology,* vol. 20, no. 1, p. 48, 2019/03/01 2019, doi: 10.1186/s13059-019-1653-z.

[42] K. E. Samocha *et al.*, "Regional missense constraint improves variant deleteriousness prediction," *bioRxiv,* p. 148353, 2017, doi: 10.1101/148353.

[43] B. Reva, Y. Antipin, and C. Sander, "Predicting the functional impact of protein mutations: application to cancer genomics," *Nucleic Acids Research,* vol. 39, no. 17, pp. e118-e118, 2011, doi: 10.1093/nar/gkr407.

[44] J. M. Schwarz, D. N. Cooper, M. Schuelke, and D. Seelow, "MutationTaster2: mutation prediction for the deep-sequencing age," *Nature Methods,* vol. 11, no. 4, pp. 361-362, 2014, doi: 10.1038/nmeth.2890.

[45] J. M. Schwarz, C. Rödelsperger, M. Schuelke, and D. Seelow, "MutationTaster evaluates disease-causing potential of sequence alterations," *Nature Methods,* vol. 7, no. 8, pp. 575-576, 2010, doi: 10.1038/nmeth0810-575.

[46] T. T. Jiang, L. Fang, and K. Wang, "Deciphering “the language of nature”: A transformer-based language model for deleterious mutations in proteins," *The Innovation,* vol. 4, no. 5, p. 100487, 2023/09/11/ 2023, doi: <https://doi.org/10.1016/j.xinn.2023.100487>.

[47] V. Pejaver *et al.*, "Inferring the molecular and phenotypic impact of amino acid variants with MutPred2," *Nature Communications,* vol. 11, no. 1, 2020, doi: 10.1038/s41467-020-19669-x.

[48] B. Li *et al.*, "Automated inference of molecular mechanisms of disease from amino acid substitutions," *Bioinformatics,* vol. 25, no. 21, pp. 2744-2750, 2009, doi: 10.1093/bioinformatics/btp528.

[49] Q. Mathieu *et al.*, "Analysis of missense variants in the human genome reveals widespread gene-specific clustering and improves prediction of pathogenicity," *The American Journal of Human Genetics,* vol. 109, no. 3, pp. 457-470, 2022, doi: <https://doi.org/10.1016/j.ajhg.2022.01.006>.

[50] H. Qi *et al.*, "MVP predicts the pathogenicity of missense variants by deep learning," *Nature Communications,* vol. 12, no. 1, 2021, doi: 10.1038/s41467-020-20847-0.

[51] S. Banerjee, K. Raman, and B. Ravindran, "Sequence Neighborhoods Enable Reliable Prediction of Pathogenic Mutations in Cancer Genomes," *Cancers,* vol. 13, no. 10, 2021, doi: 10.3390/cancers13102366.

[52] P. D. Thomas *et al.*, "PANTHER: A Library of Protein Families and Subfamilies Indexed by Function," *Genome Research,* vol. 13, no. 9, pp. 2129-2141, 2003, doi: 10.1101/gr.772403.

[53] A. Petrini *et al.*, "parSMURF, a high-performance computing tool for the genome-wide detection of pathogenic variants," (in eng), *Gigascience,* vol. 9, no. 5, May 1 2020, doi: 10.1093/gigascience/giaa052.

[54] B. Zeng, D. C. Liu, J. G. Huang, X. B. Xia, and B. Qin, "PdmIRD: missense variants pathogenicity prediction for inherited retinal diseases in a disease-specific manner," *Hum Genet,* vol. 143, no. 3, pp. 331-342, Mar 2024, doi: 10.1007/s00439-024-02645-6.

[55] E. Capriotti, R. Calabrese, and R. Casadio, "Predicting the insurgence of human genetic diseases associated to single point protein mutations with support vector machines and evolutionary information," *Bioinformatics,* vol. 22, no. 22, pp. 2729-2734, 2006, doi: 10.1093/bioinformatics/btl423.

[56] D. M. Jordan *et al.*, "Development and Validation of a Computational Method for Assessment of Missense Variants in Hypertrophic Cardiomyopathy," *The American Journal of Human Genetics,* vol. 88, no. 2, pp. 183-192, 2011, doi: 10.1016/j.ajhg.2011.01.011.

[57] V. Ramensky, P. Bork, and S. Sunyaev, "Human non-synonymous SNPs: server and survey," (in eng), *Nucleic Acids Res,* vol. 30, no. 17, pp. 3894-900, Sep 1 2002, doi: 10.1093/nar/gkf493.

[58] I. A. Adzhubei *et al.*, "A method and server for predicting damaging missense mutations," *Nature Methods,* vol. 7, no. 4, pp. 248-249, 2010, doi: 10.1038/nmeth0410-248.

[59] M. Kabir, S. Ahmed, H. Zhang, I. Rodriguez-Rodriguez, S. M. Najibi, and M. Vihinen, "PON-P3: Accurate Prediction of Pathogenicity of Amino Acid Substitutions," *Int J Mol Sci,* vol. 26, no. 5, Feb 25 2025, doi: 10.3390/ijms26052004.

[60] P. P. Gardner *et al.*, "PredictSNP: Robust and Accurate Consensus Classifier for Prediction of Disease-Related Mutations," *PLoS Computational Biology,* vol. 10, no. 1, 2014, doi: 10.1371/journal.pcbi.1003440.

[61] P. P. Gardner *et al.*, "PredictSNP2: A Unified Platform for Accurately Evaluating SNP Effects by Exploiting the Different Characteristics of Variants in Distinct Genomic Regions," *PLOS Computational Biology,* vol. 12, no. 5, 2016, doi: 10.1371/journal.pcbi.1004962.

[62] L. Sundaram *et al.*, "Predicting the clinical impact of human mutation with deep neural networks," *Nature Genetics,* vol. 50, no. 8, pp. 1161-1170, 2018, doi: 10.1038/s41588-018-0167-z.

[63] K. Jaganathan *et al.*, "Predicting expression-altering promoter mutations with deep learning," *Science,* vol. 0, no. 0, p. eads7373, doi: 10.1126/science.ads7373.

[64] A. G. de Brevern, Y. Choi, G. E. Sims, S. Murphy, J. R. Miller, and A. P. Chan, "Predicting the Functional Effect of Amino Acid Substitutions and Indels," *PLoS ONE,* vol. 7, no. 10, 2012, doi: 10.1371/journal.pone.0046688.

[65] J. D. Fife and C. A. Cassa, "Estimating clinical risk in gene regions from population sequencing cohort data," *Am J Hum Genet,* vol. 110, no. 6, pp. 940-949, Jun 1 2023, doi: 10.1016/j.ajhg.2023.05.003.

[66] N. M. Ioannidis *et al.*, "REVEL: An Ensemble Method for Predicting the Pathogenicity of Rare Missense Variants," *The American Journal of Human Genetics,* vol. 99, no. 4, pp. 877-885, 2016, doi: 10.1016/j.ajhg.2016.08.016.

[67] P. C. Ng and S. Henikoff, "Predicting deleterious amino acid substitutions," (in eng), *Genome Res,* vol. 11, no. 5, pp. 863-74, May 2001, doi: 10.1101/gr.176601.

[68] R. Vaser, S. Adusumalli, S. Leng, M. Sikic, and P. Ng, "SIFT missense predictions for genomes," *Nature protocols,* vol. 11, pp. 1-9, 12/03 2015, doi: 10.1038/nprot.2015.123.

[69] M. Hecht, Y. Bromberg, and B. Rost, "Better prediction of functional effects for sequence variants," *BMC Genomics,* vol. 16, no. S8, 2015, doi: 10.1186/1471-2164-16-s8-s1.

[70] Y. Bromberg and B. Rost, "SNAP: predict effect of non-synonymous polymorphisms on function," *Nucleic Acids Research,* vol. 35, no. 11, pp. 3823-3835, 2007, doi: 10.1093/nar/gkm238.

[71] R. Calabrese, E. Capriotti, P. Fariselli, P. L. Martelli, and R. Casadio, "Functional annotations improve the predictive score of human disease-related mutations in proteins," *Human Mutation,* vol. 30, no. 8, pp. 1237-1244, 2009, doi: 10.1002/humu.21047.

[72] H. Y. Xiong *et al.*, "RNA splicing. The human splicing code reveals new insights into the genetic determinants of disease," (in eng), *Science,* vol. 347, no. 6218, p. 1254806, Jan 9 2015, doi: 10.1126/science.1254806.

[73] K. Jaganathan *et al.*, "Predicting Splicing from Primary Sequence with Deep Learning," *Cell,* vol. 176, no. 3, pp. 535-548.e24, 2019, doi: 10.1016/j.cell.2018.12.015.

[74] N. You *et al.*, "SpliceTransformer predicts tissue-specific splicing linked to human diseases," (in eng), *Nat Commun,* vol. 15, no. 1, p. 9129, Oct 23 2024, doi: 10.1038/s41467-024-53088-6.

[75] D. Danis *et al.*, "Interpretable prioritization of splice variants in diagnostic next-generation sequencing," *The American Journal of Human Genetics,* vol. 108, no. 9, pp. 1564-1577, 2021, doi: 10.1016/j.ajhg.2021.06.014.

[76] W. Lin, J. Wells, Z. Wang, C. Orengo, and A. C. R. Martin, "Enhancing missense variant pathogenicity prediction with protein language models using VariPred," *Sci Rep,* vol. 14, no. 1, p. 8136, Apr 7 2024, doi: 10.1038/s41598-024-51489-7.

[77] H. Carter, C. Douville, P. D. Stenson, D. N. Cooper, and R. Karchin, "Identifying Mendelian disease genes with the Variant Effect Scoring Tool," *BMC Genomics,* vol. 14, no. S3, 2013, doi: 10.1186/1471-2164-14-s3-s3.

[78] M. Fang, Z. Su, H. Abolhassani, Y. Itan, X. Jin, and L. Hammarström, "VIPPID: a gene-specific single nucleotide variant pathogenicity prediction tool for primary immunodeficiency diseases," *Briefings in Bioinformatics,* vol. 23, no. 5, 2022, doi: 10.1093/bib/bbac176.

[79] E. H. Baugh *et al.*, "Robust classification of protein variation using structural modelling and large-scale data integration," *Nucleic Acids Research,* vol. 44, no. 6, pp. 2501-2513, 2016, doi: 10.1093/nar/gkw120.
