# Supplementary material for: Harnessing artificial intelligence for genomic variant prediction: advances, challenges, and future directions
Source: Gigascience. 2026 Jan 10;15:giag004. doi: 10.1093/gigascience/giag004 (PMC12888390; doi:10.1093/gigascience/giag004)
Supplement: giag004_GIGA-D-25-00463_original_submission [file giag004_giga-d-25-00463_original_submission.pdf]

GigaScience

Harnessing Artificial Intelligence for Genomic Variant Prediction: Advances, Challenges, and Future Directions

--Manuscript Draft--

**Manuscript Number:**	GIGA-D-25-00463
**Full Title:**	Harnessing Artificial Intelligence for Genomic Variant Prediction: Advances, Challenges, and Future Directions
**Article Type:**	Review
**Funding Information:**	
**Abstract:**	Accurate genetic variant interpretation is crucial for disease research and the development of targeted therapies. Artificial intelligence (AI) is transforming this field by integrating computational methodologies across structural biology, evolutionary analysis, and multimodal genomic data. This review examines the evolution from traditional rule-based systems and statistical models to contemporary machine learning, deep learning, and protein language models, while addressing critical challenges in variant classification. Key obstacles include data heterogeneity, interpretability, and Variants of Uncertain Significance (VUS) persist, emphasizing the critical need for explainable AI frameworks and more inclusive genomic databases to improve predictive accuracy across diverse populations. Based on the assessment of current tools, we propose strategies for enhanced predictor selection, effective multiomics data integration, and optimized computational workflows. These recommendations aim to enhance variant interpretation accuracy in both research settings and clinical practice, ultimately contributing to advances in personalized medicine.
**Corresponding Author:**	Mingyan Fang<br>BGI-Shenzhen: BGI Group<br>Shenzhen, China CHINA
**Corresponding Author Secondary Information:**	
**Corresponding Author's Institution:**	BGI-Shenzhen: BGI Group
**Corresponding Author's Secondary Institution:**	
**First Author:**	Indah Pakpahan
**First Author Secondary Information:**	
**Order of Authors:**	Indah Pakpahan
	Mentari Sihombing
	Haohan Liu
	Mengyao Wang
	Zheng Su
	Mingyan Fang
**Order of Authors Secondary Information:**	
**Additional Information:**	
**Question**	**Response**
Are you submitting this manuscript to a special series or article collection?	No
**Experimental design and statistics**	Yes

Powered by Editorial Manager® and ProduXion Manager® from Aries Systems Corporation

Full details of the experimental design and statistical methods used should be given in the Methods section, as detailed in our Minimum Standards Reporting Checklist. Information essential to interpreting the data presented should be made available in the figure legends.<br><br>Have you included all the information requested in your manuscript?	
**Resources**<br><br>A description of all resources used, including antibodies, cell lines, animals and software tools, with enough information to allow them to be uniquely identified, should be included in the Methods section. Authors are strongly encouraged to cite Research Resource Identifiers (RRIDs) for antibodies, model organisms and tools, where possible.<br><br>Have you included the information requested as detailed in our Minimum Standards Reporting Checklist?	Yes
**Availability of data and materials**<br><br>All datasets and code on which the conclusions of the paper rely must be either included in your submission or deposited in publicly available repositories (where available and ethically appropriate), referencing such data using a unique identifier in the references and in the "Availability of Data and Materials" section of your manuscript.<br><br>Have you have met the above requirement as detailed in our Minimum Standards Reporting Checklist?	No
If not, please give reasons for any omissions below.	This is a review article that synthesizes and discusses findings from previously published studies rather than generating or analyzing new datasets or code. Therefore,

as follow-up to "**Availability of data and materials**<br><br>All datasets and code on which the conclusions of the paper rely must be either included in your submission or deposited in publicly available repositories (where available and ethically appropriate), referencing such data using a unique identifier in the references and in the "Availability of Data and Materials" section of your manuscript.<br><br>Have you have met the above requirement as detailed in our Minimum Standards Reporting Checklist?<br><br>"	no new data or code were produced in this work. All referenced datasets and tools are publicly available in the cited original publications.
GigaScience has policies and guidelines in place for the use of generative AI-writing tools such as ChatGPT. If you have used such writing tools to assist with writing the manuscript this must be declared and cited in the text. Authors should not list AI-writing tools and other AI-assisted technologies as an author or co-author and should acknowledge that they are fully responsible for text generated or refined by AI-writing tools.<p><br><br>A summary of use (particularly in the introduction or among methods) needs to be included at the end of the paper, and the outputs should also be included as a supplementary file hosted in GigaDB or other open repositories. Please read our guidelines for more information. <p><br><br>By submitting to GigaScience, you are aware of the journal's AI-writing tools policy, and if you have declared use of such tools below, you have acknowledged this where appropriate in your manuscript and have made a summary of use and outputs available. <p><br><b>AI-assisted writing tools have been used in the preparation of this manuscript?	No

Manuscript

Click here to access/download;Manuscript;main_manuscript-20251105.docx

Harnessing Artificial Intelligence for Genomic Variant Prediction: Advances, Challenges, and Future Directions

Indah Pakpahan [1,2,3#], Mentari Sihombing [1,2,4#], Haohan Liu [2],
Mengyao Wang [2], Zheng Su [5], Mingyan Fang [6]

[1] *Dalian University of Technology, Dalian 116000, China*

[2] *BGI Research, Wuhan 430074, China*

[3] *Department of Bioprocess Engineering, Faculty of Biotechnology, Institut Teknologi Del, Laguboti, North Sumatera, Indonesia*

[4] *Department of Software Engineering, Faculty of Vocational Studies, Institut Teknologi Del, Laguboti, North Sumatera, Indonesia*

[5] *School of Biotechnology and Biomolecular Sciences, Faculty of Science, The University of New South Wales, Sydney, NSW 2052, Australia.*

[6] *State Key Laboratory of Genome and Multi-omics Technologies, BGI Research, Shenzhen 518083, China*

# These authors contributed equally to this work.

***Correspondence*:**

Mingyan Fang, fangmingyan@genomics.cn

Zheng Su, suzheng@whu.edu.cn

Abstract

Accurate genetic variant interpretation is crucial for disease research and the development of targeted therapies. Artificial intelligence (AI) is transforming this field by integrating computational methodologies across structural biology, evolutionary analysis, and multimodal genomic data. This review examines the evolution from traditional rule-based systems and statistical models to contemporary machine learning, deep learning, and protein language models, while addressing critical challenges in variant classification. Key obstacles include data heterogeneity, interpretability, and Variants of Uncertain Significance (VUS) persist, emphasizing the critical need for explainable AI frameworks and more inclusive genomic databases to improve predictive accuracy across diverse populations. Based on the assessment of current tools, we propose strategies for enhanced predictor selection, effective multiomics data integration, and optimized computational workflows. These recommendations aim to enhance variant interpretation accuracy in both research settings and clinical practice, ultimately contributing to advances in personalized medicine.

Keywords: Variant pathogenicity predictors, Artificial intelligence (AI), Variant databases, Multiomics integration, Variants of Uncertain Significance (VUS).

Background

High-throughput sequencing and the Human Genome Project have enabled comprehensive catalogs of human genetic variation, yet distinguishing pathogenic from benign variants remains a central bottleneck in research and clinical genetics [1-3]. Over the last decade, prediction tools have progressed from rule-based heuristics to statistical models, machine learning (ML), deep learning (DL), and, most recently, transformer-based large language models (LLMs) that integrate evolutionary, structural, and multi-omics signals [4, 5]. Despite these advances, persistent challenges include the high prevalence of variants of uncertain significance (VUS), limited performance for non-coding/regulatory variants, ancestry bias in reference datasets, and the limited interpretability of state-of-the-art models.

This review synthesizes the technological evolution of variant pathogenicity predictors; maps the supporting database ecosystem; outlines a practical workflow for data preprocessing, model development, and evaluation; and highlights translational gaps that must be addressed to enable routine clinical use. We provide actionable recommendations for predictor selection, multi-omics integration, and validation strategies to improve reliability and equity in variant interpretation. We performed a narrative, method-focused search of PubMed and Google Scholar combining terms such as variant pathogenicity, in silico prediction, deep learning, transformer/foundation model, splicing prediction, non-coding variants, functional assays, and database/tool names. We prioritized peer-reviewed method papers with clear training/validation descriptions, widely used tools across coding and non-coding tasks, comparative evaluations, and resources on functional screening, fairness, explainable AI (XAI) and screened reference lists of seminal studies to identify additional sources.

Despite rapid methodological advances, several issues continue to limit the clinical utility of variant prediction. A substantial proportion of variants remain classified as VUS, reducing their immediate diagnostic impact. Reference datasets such as gnomAD and ClinVar exhibit

ancestry imbalances that affect generalizability, while deep learning and transformer-based models often operate as “black boxes,” complicating their alignment with ACMG/AMP interpretive guidelines. Moreover, experimental validation lags behind computational predictions, creating a gap between algorithmic output and clinical translation. These challenges define the central focus of this review, which highlights both the progress achieved and the barriers that must be addressed for equitable, explainable, and clinically actionable variant interpretation.

Curated Database Infrastructure Supporting Variant Interpretation

The accurate interpretation of genetic variants relies on a stratified data ecosystem integrating genomic, clinical, and functional evidence, in which each layer offers a distinct analytical perspective while remaining interconnected to enable comprehensive pathogenicity assessment. Population-scale genomic initiatives provide baseline frequency profiling, establishing the fundamental context for variant rarity assessment. Resources including the Single Nucleotide Polymorphism Database (dbSNP) [6], the 1000 Genomes Project (1KGP) [7], Genome Aggregation Database (gnomAD) [8], and UK Biobank [9] provide allele frequency distributions and mutational constraint profiles across diverse ancestries, enabling the critical distinction between rare pathogenic variants from common benign polymorphisms, especially in underrepresented populations [10].

Pathogenicity evidence emerges from curated clinical and disease-specific repositories. ClinVar [11], The Human Gene Mutation Database (HGMD) [12], Human Variants Database (HuVarBase) [13], and ClinGen [14] aggregate experimentally or clinically validated genotype-phenotype associations, forming the empirical foundation for supervised learning approaches in variant classification.

Standardized disease ontologies transform clinical observations into computational frameworks. Online Mendelian Inheritance in Man (OMIM) [15], Orphanet [16], and Human Phenotype Ontology (HPO) [17] connect genetic alterations to specific disease mechanisms and biological pathways. Additionally, Gene Ontology (GO) [18] complements phenotype-focused resources by clarifying functional impacts at molecular and cellular levels.

Domain-specific repositories provide further refinement of variant interpretation in specialized contexts. Oncology-focused databases, such as Catalogue of Somatic Mutations in Cancer (COSMIC) [19], Database of Curated Mutations (DoCM) [20], and ONGene [21] aggregate tumor-specific mutations. Gene-centric functional assays (e.g., a curated *BRCA1* functional dataset [22]), provide high-confidence labels for genes with significant clinical relevance.

Beyond sequence and phenotype resources, protein architecture databases including Universal Protein Resource (UniProt) [23] and Protein Data Bank (PDB) [24] provide the three-dimensional framework necessary for understanding variant consequences at the molecular level, particularly crucial for structure-function relationship modeling.

Finally, specialized benchmarking and validation resources, including the Benchmark Database for Variations (VariBench) [25], VariSNP [26], and VarCards2 [27] enable rigorous model evaluation and comparison of emerging AI predictors by providing standardized test sets and performance metrics.

Across this data ecosystem (**Table 1**) spanning evolutionary, biochemical, structural, and regulatory domains lies the foundation for advanced AI architectures that can effectively model the complex relationships underlying variant pathogenicity [28].

The Technological Trajectory of Variant Pathogenicity Assessment

The development of computational variant consequence prediction has evolved through four distinct yet overlapping paradigms, each addressing limitations of previous approaches while

expanding analytical capabilities (**Figure 1**). This progression mirrors broader technological trends within computational biology, evolving from initial rule-based heuristics toward advanced deep learning architectures. Each step has enabled progressively more refined insights into genomic variation (**Additional file 1, Supplementary Table 1**).

Phase 1: Rule-Based Biological Heuristics

Early predictive tools emerged from foundational biological insights. Pioneering rule-based predictors such as Sorting Intolerant From Tolerant (SIFT) [29] and Polymorphism Phenotyping (PolyPhen) [30] relied on empirical knowledge and evolutionary principles to evaluate variant pathogenicity. These tools primarily relied on sequence conservation and amino acid physicochemical properties to distinguish between benign and deleterious variants. Gene-Aware Variant INterpretation (GAVIN) [31] applied predefined classification logic to refine pathogenicity predictions within gene-specific contexts. Their strength lay in their high interpretability, as the underlying biological rules were explicit and transparent. However, this rule-based approach inherently limited their scalability and ability to capture the complex, non-linear patterns emerging from rapidly expanding genomic datasets. Though computationally efficient, these methods typically operated within limited genomic contexts and largely overlooked non-coding or regulatory regions such as promoters or splice sites [32]. Despite these constraints, early-phase predictors remain valuable for preliminary assessments and continue to be incorporated into comprehensive prediction frameworks [33], particularly where interpretability is prioritized over prediction complexity.

Phase 2: Statistical Modeling and Probabilistic Frameworks

As genomic databases expanded in both size and diversity, statistical methods emerged to enhance the prediction accuracy through probabilistic modeling. Tools such as MutationAssessor [34] utilized evolutionary conservation patterns within protein families, while Functional Analysis Through Hidden Markov Models (FATHMM) [35] integrated

evolutionary conservation scores into sequence-based probabilistic models to estimate variant pathogenicity. Eigen [36], an unsupervised spectral method, prioritizes variants by analyzing annotation correlations and constructing a weighted score across both coding and non-coding genomic regions. In parallel, Genomic Evolutionary Rate Profiling++ (GERP++) [37] quantifies evolutionary constraint using a maximum likelihood model to calculate rejected substitutions, and is widely used as an annotation feature in downstream predictive frameworks. While these tools have advanced the ability to contextualize genomic variation, predictive methods still face substantial challenges when analyzing rare or novel variants due to insufficient representation in existing reference datasets. Additionally, their heavy reliance on high-quality reference annotations limited their effectiveness in classifying clinically important yet poorly characterized VUS [38], prompting further developments toward data-driven machine learning methods.

Phase 3: Machine Learning (ML) and Ensemble Approaches

ML algorithms transformed variant prediction by capturing complex, non-linear patterns in multidimensional genomic datasets, enabling integration of diverse biological features [39]. Approaches in this era can be broadly categorized into:

Classical Machine Learning Classifiers: Naive Bayes classifiers were applied in tools like PolyPhen-2 [40], which integrates sequence and structure-based features to predict the effects of amino acid substitutions. This algorithm was also effectively deployed in disease-specific contexts, such as Polymorphism Phenotyping for Hypertrophic Cardiomyopathy (PolyPhen-HCM) [41], CanPredict [42], and the splicing predictor SPANR [43]. Support Vector Machines (SVMs) and Random Forests underpin tools such as Combined Annotation Dependent Depletion (CADD) [32], Predictor of human Deleterious Single Nucleotide Polymorphisms (PhD-SNP) [44], Variant Effect Scoring Tool (VEST) [45], Meta-analytic Support Vector Machine (MetaSVM) [46], and MutPred [47], incorporating diverse features such as

evolutionary conservation, protein structure, and gene-level annotations within unified predictive frameworks. The Random Forest algorithm was similarly adapted for specialized tasks, powering tools like the Variant Impact Predictor for PIDs (VIPPID) [48] for immunodeficiencies and Prediction of Deleterious Missense Mutation for IRDs (PdmIRD) [49] for retinal diseases. For instance, SVMs identify optimal hyperplanes to separate classes, while Random Forests build multiple decision trees and aggregate their results, offering robustness and handling high-dimensional data effectively.

Gradient Boosting Approaches: More recent implementations employ gradient boosting machines (GBMs) to enhance classification performance. Multimodal Annotation Generated Pathogenic Impact Evaluator (MAGPIE) [5], Consequence-Agnostic Pathogenicity Interpretation of Clinical Exome variations (CAPICE) [50], INDELpred [51], Mendelian Clinically Applicable Pathogenicity (M-CAP) [52], and PON-P3 [53] exemplifies this approach by integrating multi-source annotations via gradient-boosted decision trees. The strength of GBMs for leveraging complex feature sets also made them ideal for building specialized predictors like CardioBoost [54] for cardiac genetics. GBMs operate on the principle of iterative improvement: they sequentially build models, with each new model attempting to correct the errors of its predecessor. While these models demonstrate superior accuracy, they require high-quality, well-curated training datasets; when data are limited, sparse or imbalanced, challenges such as overfitting and limited generalizability can compromise their clinical utility [55].

Ensemble Prediction Systems: To address the inherent limitations of individual algorithms, ensemble methodologies aggregate outputs from multiple predictors, thereby enhancing robustness and reliability [56]. Tools such as the Rare Exome Variant Ensemble Learner (REVEL) [57] and Parallel SMote Undersampled Random Forest (parSMURF) [58] exemplify this strategy by combining complementary models to mitigate individual weaknesses while

amplifying collective strengths. Although ensemble methods introduce additional computational costs and diminish transparency, these frameworks have become integral to clinical genetics pipelines where reliability is paramount. Despite improved accuracy, ML-based methods remain sensitive to training data and often lack interpretability, limiting their use as standalone clinical tools.

Phase 4: Deep Learning (DL) Approaches

DL has further advanced variant interpretation by exploiting neural networks to model highly nonlinear relationships in large, multidimensional genomic datasets [59]. DL models can learn hierarchical feature representations directly from raw data, reducing the reliance on manual feature engineering. Notable examples include DANN [60] uses a deep neural network trained on the same functional annotations as CADD to score both coding and non-coding variants and PrimateAI [61], which leverages evolutionary signatures across primates to enhance prediction accuracy. This capacity for learning from raw sequence data revolutionized the prediction of non-coding and regulatory variants. Models like DeepSEA [62] and Basenji [63] demonstrated that chromatin accessibility and gene expression could be predicted directly from DNA sequence, capturing both proximal and distal effects. In splicing prediction, DL brought a significant leap in accuracy. SpliceAI [64] utilized convolutional neural networks to capture long-range dependencies in pre-mRNA sequences, vastly outperforming previous models, though at the cost of interpretability. MMSplice [65] and SQUIRLS [66] attempted to address this interpretability gap through more modular or explainable designs. While these approaches demonstrate unprecedented sensitivity, challenges persist regarding their "black box" nature and intensive computational requirements [67].

Phase 5: Transformer Architectures

The most recent technological leap has been catalyzed by adapting transformer architectures and language modeling principles to genomic and protein sequences. Originally developed for

natural language processing, transformer models have become powerful tools for predicting functional impacts of genetic variants [68]. The core innovation lies in the self-attention mechanism, which allows the model to weigh the importance of different parts of the input sequence when processing each element [69]. This enables the capture of long-range dependencies and global context within biological sequences, a crucial aspect for understanding how mutations in one part of a protein can affect distant functional regions [70].

The application of transformers has bifurcated into two powerful, complementary paradigms;

Protein Language Models (pLMs): Pre-trained on vast corpora of evolutionary sequences, pLMs learn fundamental principles of protein structure and function. Models like ESM-1b [71] excel at capturing subtle sequence constraints, distinguishing isoform-specific pathogenic variants with high accuracy (ROC-AUC: 0.905 on ClinVar, 0.897 on HGMD/gnomAD). This approach is exemplified by tools like Variant impact Predictor (VariPred) [72], which leverages these learned representations to predict variant effects, often outperforming traditional structure-dependent methods. The strength of pLMs is further demonstrated by their versatility in tasks such as masked residue prediction (82% accuracy) [73] and sequence conservation analysis (MCC = 0.596) [74].

Regulatory Genome Transformers: These models are designed to interpret the non-coding genome by learning the regulatory code directly from DNA sequence. Enformer [75] set a new standard by using a transformer architecture to achieve state-of-the-art accuracy in predicting chromatin accessibility and gene expression profiles from sequence context, capturing effects of distal enhancers. This paradigm is extended by tools like AlphaGenome [76], which integrates over 100 regulatory features in a multitask framework, and PromoterAI [77], which focuses specifically on predicting the impact of promoter variants.

The true power of this era is revealed in models that fuse these approaches or leverage their insights for specific clinical tasks. AlphaMissense [68] by DeepMind represents a seminal work,

fusing structural insights from AlphaFold [78] with the pattern recognition of language models to generate a massive, highly accurate map of missense variant pathogenicity. Similarly, MutFormer [79] integrates self-attention with convolutional layers for missense analysis, and Genetic Transformer (GeneT) [80] achieves remarkable recall rates (99% in synthetic data, 97.85% in clinical cohorts) for identifying causative variants. This fusion also enables more specialized applications, such as SpTransformer [81], which incorporates tissue specificity into splicing prediction.

Together, these findings demonstrate that transformer architectures consistently outperform previous deep learning approaches across the full spectrum of variant interpretation tasks, from coding to non-coding variants. They mark a shift towards models that learn the fundamental "language" of biology itself, offering unprecedented accuracy at the cost of increased computational complexity and continued challenges in interpretability. Representative algorithms and tools across these technological phases are summarized in **Table 2**.

Table 2. Computational Algorithm used for Variant Prediction

No.	Algorithm	Description	Tools	References
1	Naive Bayes	A probabilistic graphical model that represents a set of variables and their conditional dependencies.	PolyPhen-2, SPANR, PolyPhen-HCM, CanPredict	[40-43]
2	Support Vector Machines	A supervised learning model that analyzes data for classification and regression analysis.	CADD, PhD-SNP, MetaSVM	[32, 44, 46]
3	Random Forest	An ensemble learning method that operates by constructing multiple decision trees.	VEST, MutPred, GWAVA, REVEL, parSMURF, SQUIRLS, VIPPID, PdmIRD	[45, 47-49, 57, 58, 66, 82]
4	Gradient Boosting Machines	A machine learning technique for regression and classification problems that builds a model in a stage-wise fashion.	MAGPIE, CAPICE, INDELpred, M-CAP PON-P3, CardioBoost	[5, 50-54]

5	Neural Networks	A set of algorithms modeled after the human brain, designed to recognize patterns.	DANN, DeepSEA, PrimateAI, SpliceAI, MMSplice, Basenji, PromotorAI, EVE, AIVAR	[60-65, 77, 83, 84]
6	Transformer	A deep learning model that uses self-attention mechanisms to process sequential data, capturing long-range dependencies and relationships in the data.	AlphaMissense, VariPred, SpTransformer, Enformer, AlphaGenome, EpiGePT, MutFormer, GeneT	[68, 72, 75, 76, 79-81, 85]

Integrative Tools for Variant Annotation

Comprehensive annotation pipelines consolidate outputs from diverse databases and predictive algorithms to streamline variant interpretation. Ensembl Variant Effect Predictor (VEP) [86] annotates variants with gene-level and regulatory features, supporting flexible plug-in integration of tools such as SIFT, CADD, and SpliceAI. ANNOVAR [87] enables gene-based, region-based, and filter-based annotation in unified workflows, incorporating population frequency data (*e.g.,* gnomAD) and multiple pathogenicity scores.

Web-based platforms further enhance usability and evidence integration. VarSome [88] and MobiDetails [89] aggregate clinical annotations, *in silico* predictions, and allele frequencies via interactive interfaces, apply ACMG-based classification framework. InterVar [90], emphasizes rule-based implementation of ACMG guidelines, offering reproducible, guideline-concordant classification support. Exomiser [91] combines variant pathogenicity scores with phenotype data (HPO terms) to prioritize candidate variants, especially in rare disease diagnosis.

These integrative tools reduce the burden of manual curation by consolidating diverse resources into unified workflows, enabling efficient filtering and evidence synthesis for both research and clinical applications. However, while these pipelines effectively consolidate evidence from multiple sources, the complexity of variant interpretation requires a systematic

approach to optimize their implementation. The selection of appropriate predictors and the integration of their outputs into coherent clinical decisions necessitates a structured workflow framework.

Variant Interpretation Workflow and Evaluation Considerations

To address these challenges systematically, the variant interpretation process can be conceptualized as a structured pipeline that links raw genomic data to actionable clinical predictions (**Figure 2**). This systematic approach provides a rigorous foundation for improved variant classification accuracy and supports the development of more equitable genomic medicine applications.

This systematic framework encompasses three interconnected phases that collectively transform raw genomic data into clinically actionable insights. The initial phase centers on comprehensive data acquisition, integrating population-level variant frequencies, disease-specific variants repositories, high-throughput molecular characterization profiles, and curated clinical annotations. This foundation transitions into an advanced preprocessing, systematically integrating heterogeneous data sources, including evolutionary conservation, protein structural, and regulatory element characterizations. Subsequent normalization, feature extraction, and dimensionality reduction to optimize the computational feature space for downstream analytical applications [92].

The analytical phase leverages sophisticated predictive modeling approaches, employing rigorous hyperparameter optimization strategies to achieve maximum discriminative performance [93]. This computational framework generates probabilistic assessments that require systematic validation through robust benchmarking protocols. Evaluation encompasses multiple complementary metrics: sensitivity and specificity for detection of pathogenic versus benign variants, precision metrics that characterize predictive accuracy, and the area under the

receiver operating characteristic (ROC) curve (AUC), which provides threshold-independent assessment of discriminative performance [94]. Comparative analyses against benchmark datasets, such as ClinVar or consortium-established validation cohorts, enable cross-method evaluations while ensuring generalizability through cross-validation [95].

The optimization of these computational requires adherence to stringent development protocols that emphasize data integrity through integration of high-quality variant annotations from diverse repositories such as gnomAD and ClinVar. Advanced feature engineering requires integration of multi-omics data layers spanning genetic, proteomic, regulatory, and clinical domains [96]. Robust model development paradigms demand rigorous cross-validation, systematic hyperparameter tuning, and validation against independent external datasets to ensure reproducibility and clinical applicability [97].

Challenges Limiting Clinical Translation

Despite these methodological advances across both general and specialized AI approaches, several interconnected challenges continue to impede the clinical deployment of variant prediction tools. These challenges span data limitations, dataset biases, and model interpretability, each of which requires targeted solutions to bridge the gap between computational prediction and clinical application.

Data Limitations and Functional Validation Bottlenecks

Variants of uncertain significance (VUS) continue to represent a large fraction of clinical findings, particularly for rare or non-coding variants [98]. While computational methods such as matrix factorization [99] and active learning [100] provide incremental gains, they remain dependent on sparse or biased training data. Recent years have seen growing reliance on high-throughput functional assays such as CRISPR-Cas9 screens and Multiplexed Assays of Variant Effect (MAVEs), which can directly measure variant function at scale [101] . These approaches

are increasingly feeding into clinical frameworks, for example MAVE-derived scores have been incorporated into ClinGen rules for BRCA1, TP53, and PTEN [102]. Yet, routine clinical use is limited by assay cost, turnaround time, and availability outside specialized centers.

Dataset Biases and Generalizability Issues

Beyond general data limitations, demographic imbalances in genomic databases primarily skewed toward individuals of European ancestry significantly restrict model performance across diverse populations [103]. These sampling biases lead to reduced prediction accuracy in underrepresented groups [104]. Solutions such as targeted sequencing of diverse populations and federated learning also show promise [105], but equitable performance will also require deliberate strategies to validate variants across ancestries. Emerging data confirm that integrating MAVE results and diverse cohort sequencing can help reduce disparities [106].

Model Interpretability versus Predictive Power

Even when data issues are mitigated, the interpretability of advanced AI models remains a significant barrier to clinical adoption. ML models like Evolutionary Model of Variant Effect (EVE) [84] have demonstrated high performance in variant prediction but are often too complex to explain and their opacity hampers clinical trust, regulatory approval, and usability[107, 108]. Recent efforts in XAI strategies [109] and interpretable classifiers like Artificial Intelligent Variant Classifier (AIVAR) [83] seek to bridge this gap by providing transparent and biologically meaningful rationales for prediction.

Toward More Interpretable and Clinically Actionable Predictions

Emerging trends ranging from multi-omics fusion to foundation models, promise to redefine variant interpretation along four strategic axes.

Multi-Omics Data Integration

Recent advances in multi-omics data integration significantly enhance the ability to predict clinical outcomes and elucidate complex biological processes. Numerous tools and

methodologies have been developed to integrate genomic, transcriptomic, proteomic, and epigenetic data [110], improving statistical power for identifying associations with low-frequency variants [111]. Despite challenges in data storage, processing, and analysis stemming from the scale and heterogeneity of these datasets, ongoing research is developing robust methods that leverage complementary multi-omics layers to enhance predictive modeling and biomarker discovery. Emerging strategies such as selective prioritization of high-value omics layers and shared multi-omics databases, offer a path toward scalable adoption.

Foundation/LLM Models & Few-Shot Adaptation

Transformer-based language models have demonstrated exceptional performance across diverse variant interpretation contexts. Their ability to perform few-shot adaptation enables fine-tuning with minimal labeled data, addressing challenges posed by rare variants and limited data in rare diseases. Pre-trained on large, unlabeled datasets, these models can be efficiently adapted to novel variant types using parameter-efficient fine-tuning (PEFT) [112] and prompt engineering [113], making high-performance variant prediction accessible for understudied conditions. These models effectively capture complex biological sequence relationships and domain-specific constraints, establishing transformer-based approaches as a pivotal advancement in variant prediction. Indeed, recent benchmarking of DNA foundation models confirms their capacity for zero-shot variant effect prediction, highlighting that model performance is critically dependent on the training data, with large, multi-species architectures demonstrating superior discriminative power in capturing both local and extended contextual effects [114].

To understand how these sophisticated models translate biological sequences into clinical predictions, it is instructive to examine their underlying computational architecture (**Figure 3**). The transformation process encompasses three interconnected phases that systematically bridge sequence-level information with pathogenicity assessment. Initially, biological

sequences undergo tokenization and embedding generation, converting raw genomic data into computational representations. Subsequently, contextual encoding via multi-head self-attention mechanisms processes these embeddings, where unsupervised pre-training objectives, including masked language modeling (MLM), permutation language modeling (PLM), and contrastive learning (CL) [115], enable the models to learn meaningful sequence patterns without labeled data. The final phase involves task-specific adaptation occurs through specialized prediction heads coupled with either frozen or fine-tuned neural backbones, where prompt-engineering strategies enable efficient knowledge transfer across domains when faced with limited labeled datasets.

Scalable Functional Validation & Systems Biology

Emerging efforts are focused on scaling functional validation beyond specialized centers. Resources such as the Atlas of Variant Effects [116] and MaveDB [117] are beginning to provide standardized repositories of multiplexed assay data that can be directly incorporated into ACMG/AMP classification frameworks. At the same time, integration of functional readouts with systems biology analyses including pathway and interaction networks, promises to contextualize variant effects at multiple biological levels [118]. Together, these developments point toward functional evidence becoming a routine and standardized component of precision medicine rather than an ad hoc supplement.

Expanding Diversity and Ethical AI to Reduce Bias

A critical future direction involves systematically diversifying genomic databases to address biases arising from population underrepresentation [103]. This goal requires two complementary approaches. First, collaborative initiatives like the Human Heredity and Health in Africa (H3Africa) consortium [119] provide models for ethical, scientifically robust data collection among underserved populations. Second, the integration of ethical AI practices, such as fairness assessments, transparent model documentation, and inclusive stakeholder

engagement plays a pivotal role in mitigating algorithmic biases. These frameworks must prioritize equitable performance across diverse populations and healthcare settings. By combining responsible AI strategies with deliberate efforts to diversify genomic reference databases, the field can address longstanding disparities in genomic medicine, ensuring benefits for patients worldwide.

Conclusions

In summary, the integration of diverse datasets, transparent predictive algorithms, and consistent validation practices is essential to move AI-driven variant interpretation into routine clinical use. The rapid advancement of computational tools has significantly enhanced our ability to predict genetic variant pathogenicity, offering scalability and accuracy. However, several challenges remain, particularly around accurately classifying VUS, and ensuring fairness and transparency of predictive models. Continued improvement will depend on effectively integrating multi-omics information, expanding international cooperation to diversify genomic datasets, and systematically linking computational predictions to robust experimental validations. Through these concerted efforts, next-generation computational tools will fully realize the promise of personalized medicine, guiding clinicians and researchers toward deeper mechanistic insights and improved patient care.

Data Availability

Not applicable.

List of abbreviations

1KGP: 1000 Genomes Project; AI: Artificial Intelligence; AIVAR: Artificial Intelligent Variant Classifier; AUC: Area Under the Curve; CAPICE: Consequence-Agnostic Pathogenicity Interpretation of Clinical Exome Variations; CADD: Combined Annotation Dependent Depletion; CL: Contrastive Learning; COSMIC: Catalogue of Somatic Mutations in Cancer; dbSNP: Single Nucleotide Polymorphism Database; DL: Deep Learning; DoCM: Database of Curated Mutations; EVE: Evolutionary Model of Variant Effect; FATHMM: Functional Analysis Through Hidden Markov Models; FM-HCR: Fluorescent Multiplex Host Cell Reactivation; GBM: Gradient Boosting Machines; GERP++: Genomic Evolutionary Rate Profiling ++; GAVIN: Gene-Aware Variant INterpretation; GO: Gene Ontology; gnomAD: Genome Aggregation Database; H3Africa: Human Heredity and Health in Africa; HGMD: Human Gene Mutation Database; HPO: Human Phenotype Ontology; HuVarBase: Human Variants Database; IRDs: Inherited Retinal Diseases; LLM: Large Language Models; MAGPIE: Multimodal Annotation Generated Pathogenic Impact Evaluator; MAVEs: Multiplexed Assays of Variant Effect; M-CAP: Mendelian Clinically Applicable Pathogenicity; MetaSVM: Meta-analytic Support Vector Machine; ML: Machine Learning; MLM: Masked Language Modeling; OMIM: Online Mendelian Inheritance in Man; parSMURF: Parallel SMote Undersampled Random Forest; PDB: Protein Data Bank; PdmIRD: Prediction of Deleterious Missense Mutation for IRDs; PhD-SNP: Predictor of human Deleterious Single Nucleotide Polymorphisms; PLM: Permutation Language Modeling; PolyPhen: Polymorphism Phenotyping; PolyPhen-HCM: Polymorphism Phenotyping for Hypertrophic Cardiomyopathy; REVEL: Rare Exome Variant Ensemble Learner; ROC: Receiver Operating Characteristic; SIFT: Sorting Intolerant From Tolerant; SVM: Support Vector Machines; VEP: Variant Effect

Predictor; VEST: Variant Effect Scoring Tool; VariBench: Benchmark Database for Variations; VariPred: Variant impact Predictor; VIPPID: Variant Impact Predictor for Primary Immunodeficiency Diseases; VUS: Variants of Uncertain Significance; XAI: Explainable Artificial Intelligence.

Ethics approval and consent to participate

Not applicable.

Consent for publication

Not applicable.

Competing interests

The authors declare that they have no competing interests.

Funding

No funding was received.

Authors' contributions

IP, MS: Conceptualization, Investigation, Methodology, Writing – original draft

HL, MW: Visualization

ZS, MF: Conceptualization, Supervision, Project administration, Writing – review & editing

All authors read and approved the final manuscript.

Acknowledgments

We would like to thank Dr. Merry Meryam Martgrita from the Department of Bioprocess Engineering at Institut Teknologi Del, and Riyanthi Angrainy Sianturi from the Department of Software Engineering at Institut Teknologi Del, for their valuable advice.

References

[1] O. S. Aworunse, O. Adeniji, O. L. Oyesola, I. Isewon, J. Oyelade, and O. O. Obembe, "Genomic Interventions in Medicine," *Bioinformatics and Biology Insights,* vol. 12, 2018, doi: 10.1177/1177932218816100.

[2] S. M. Rego and M. P. Snyder, "High Throughput Sequencing and Assessing Disease Risk," *Cold Spring Harbor Perspectives in Medicine,* vol. 9, no. 1, 2019, doi: 10.1101/cshperspect.a026849.

[3] M. Spielmann and M. Kircher, "Computational and experimental methods for classifying variants of unknown clinical significance," (in eng), *Cold Spring Harb Mol Case Stud,* vol. 8, no. 3, Apr 2022, doi: 10.1101/mcs.a006196.

[4] Y. Boulaimen *et al.*, "Integrating Large Language Models for Genetic Variant Classification," *arXiv preprint arXiv:2411.05055,* 2024.

[5] Y. Liu, T. Zhang, N. You, S. Wu, and N. Shen, "MAGPIE: accurate pathogenic prediction for multiple variant types using machine learning approach," *Genome Medicine,* vol. 16, no. 1, p. 3, 2024/01/08 2024, doi: 10.1186/s13073-023-01274-4.

[6] S. T. Sherry *et al.*, "dbSNP: the NCBI database of genetic variation," (in eng), *Nucleic Acids Res,* vol. 29, no. 1, pp. 308-11, Jan 1 2001, doi: 10.1093/nar/29.1.308.

[7] A. Auton *et al.*, "A global reference for human genetic variation," *Nature,* vol. 526, no. 7571, pp. 68-74, 2015, doi: 10.1038/nature15393.

[8] K. J. Karczewski *et al.*, "The ExAC browser: displaying reference data information from over 60 000 exomes," *Nucleic Acids Research,* vol. 45, no. D1, pp. D840-D845, 2017, doi: 10.1093/nar/gkw971.

[9] N. Allen *et al.*, "UK Biobank: Current status and what it means for epidemiology," *Health Policy and Technology,* vol. 1, no. 3, pp. 123-126, 2012/09/01/ 2012, doi: https://doi.org/10.1016/j.hlpt.2012.07.003.

[10] K. J. Karczewski *et al.*, "The mutational constraint spectrum quantified from variation in 141,456 humans," *Nature,* vol. 581, no. 7809, pp. 434-443, May 2020, doi: 10.1038/s41586-020-2308-7.

[11] M. J. Landrum *et al.*, "ClinVar: public archive of relationships among sequence variation and human phenotype," *Nucleic Acids Res,* vol. 42, no. Database issue, pp. D980-5, Jan 2014, doi: 10.1093/nar/gkt1113.

[12] D. N. Cooper, E. V. Ball, and M. Krawczak, "The human gene mutation database," *Nucleic acids research,* vol. 26, no. 1, pp. 285-7, Jan 1 1998, doi: 10.1093/nar/26.1.285.

[13] K. Ganesan, A. Kulandaisamy, S. Binny Priya, and M. M. Gromiha, "HuVarBase: A human variant database with comprehensive information at gene and protein levels," (in eng), *PLoS One,* vol. 14, no. 1, p. e0210475, 2019, doi: 10.1371/journal.pone.0210475.

[14] H. L. Rehm *et al.*, "ClinGen — The Clinical Genome Resource," *New England Journal of Medicine,* vol. 372, no. 23, pp. 2235-2242, 2015, doi: doi:10.1056/NEJMsr1406261.

[15] A. Hamosh, A. F. Scott, J. Amberger, D. Valle, and V. A. McKusick, "Online Mendelian Inheritance in Man (OMIM)," (in eng), *Hum Mutat,* vol. 15, no. 1, pp. 57-61, 2000, doi: 10.1002/(sici)1098-1004(200001)15:1<57::Aid-humu12>3.0.Co;2-g.

[16] S. S. Weinreich, R. Mangon, J. J. Sikkens, M. E. Teeuw, and M. C. Cornel, "[Orphanet: a European database for rare diseases]," (in dut), *Ned Tijdschr Geneeskd,* vol. 152, no. 9, pp. 518-9, Mar 1 2008. Orphanet: een Europese database over zeldzame ziekten.

[17] P. N. Robinson, S. Köhler, S. Bauer, D. Seelow, D. Horn, and S. Mundlos, "The Human Phenotype Ontology: a tool for annotating and analyzing human hereditary disease," (in eng), *Am J Hum Genet,* vol. 83, no. 5, pp. 610-5, Nov 2008, doi: 10.1016/j.ajhg.2008.09.017.

[18] M. Ashburner *et al.*, "Gene ontology: tool for the unification of biology. The Gene Ontology Consortium," (in eng), *Nat Genet,* vol. 25, no. 1, pp. 25-9, May 2000, doi: 10.1038/75556.

[19] S. Bamford *et al.*, "The COSMIC (Catalogue of Somatic Mutations in Cancer) database and website," (in eng), *Br J Cancer,* vol. 91, no. 2, pp. 355-8, Jul 19 2004, doi: 10.1038/sj.bjc.6601894.

[20] B. J. Ainscough *et al.*, "DoCM: a database of curated mutations in cancer," (in eng), *Nat Methods,* vol. 13, no. 10, pp. 806-7, Sep 29 2016, doi: 10.1038/nmeth.4000.

[21] Y. Liu, J. Sun, and M. Zhao, "ONGene: A literature-based database for human oncogenes," *Journal of Genetics and Genomics,* vol. 44, no. 2, pp. 119-121, 2017, doi: 10.1016/j.jgg.2016.12.004.

[22] G. M. Findlay *et al.*, "Accurate classification of BRCA1 variants with saturation genome editing," *Nature,* vol. 562, no. 7726, pp. 217-222, 2018, doi: 10.1038/s41586-018-0461-z.

[23] R. Leinonen, F. G. Diez, D. Binns, W. Fleischmann, R. Lopez, and R. Apweiler, "UniProt archive," (in eng), *Bioinformatics,* vol. 20, no. 17, pp. 3236-7, Nov 22 2004, doi: 10.1093/bioinformatics/bth191.

[24] H. M. Berman *et al.*, "The Protein Data Bank," (in eng), *Nucleic Acids Res,* vol. 28, no. 1, pp. 235-42, Jan 1 2000, doi: 10.1093/nar/28.1.235.

[25] P. Sasidharan Nair and M. Vihinen, "VariBench: a benchmark database for variations," (in eng), *Hum Mutat,* vol. 34, no. 1, pp. 42-9, Jan 2013, doi: 10.1002/humu.22204.

[26] G. C. Schaafsma and M. Vihinen, "VariSNP, a benchmark database for variations from dbSNP," (in eng), *Hum Mutat,* vol. 36, no. 2, pp. 161-6, Feb 2015, doi: 10.1002/humu.22727.

[27] Z. Wang *et al.*, "VarCards2: an integrated genetic and clinical database for ACMG-AMP variant-interpretation guidelines in the human whole genome," *Nucleic Acids Research,* vol. 52, no. D1, pp. D1478-D1489, 2024, doi: 10.1093/nar/gkad1061.

[28] M. Yazar and P. Ozbek, "In Silico Tools and Approaches for the Prediction of Functional and Structural Effects of Single-Nucleotide Polymorphisms on Proteins: An Expert Review," *OMICS,* vol. 25, no. 1, pp. 23-37, Jan 2021, doi: 10.1089/omi.2020.0141.

[29] P. C. Ng and S. Henikoff, "Predicting deleterious amino acid substitutions," (in eng), *Genome Res,* vol. 11, no. 5, pp. 863-74, May 2001, doi: 10.1101/gr.176601.

[30] V. Ramensky, P. Bork, and S. Sunyaev, "Human non-synonymous SNPs: server and survey," (in eng), *Nucleic Acids Res,* vol. 30, no. 17, pp. 3894-900, Sep 1 2002, doi: 10.1093/nar/gkf493.

[31] K. J. van der Velde *et al.*, "GAVIN: Gene-Aware Variant INterpretation for medical sequencing," *Genome Biology,* vol. 18, no. 1, p. 6, 2017/01/16 2017, doi: 10.1186/s13059-016-1141-7.

[32] M. Kircher, D. M. Witten, P. Jain, B. J. O'Roak, G. M. Cooper, and J. Shendure, "A general framework for estimating the relative pathogenicity of human genetic variants," *Nat Genet,* vol. 46, no. 3, pp. 310-5, Mar 2014, doi: 10.1038/ng.2892.

[33] R. A. Ertürk and M. Baysan, "Utilizing Tree-Based Algorithms for Genetic Variant Interpretation," in *2024 9th International Conference on Computer Science and Engineering (UBMK)*, 26-28 Oct. 2024 2024, pp. 689-694, doi: 10.1109/UBMK63289.2024.10773498.

[34] B. Reva, Y. Antipin, and C. Sander, "Predicting the functional impact of protein mutations: application to cancer genomics," *Nucleic Acids Res,* vol. 39, no. 17, p. e118, Sep 1 2011, doi: 10.1093/nar/gkr407.

[35] H. A. Shihab *et al.*, "Predicting the functional, molecular, and phenotypic consequences of amino acid substitutions using hidden Markov models," *Hum Mutat,* vol. 34, no. 1, pp. 57-65, Jan 2013, doi: 10.1002/humu.22225.

[36] I. Ionita-Laza, K. McCallum, B. Xu, and J. D. Buxbaum, "A spectral approach integrating functional genomic annotations for coding and noncoding variants," *Nature Genetics,* vol. 48, no. 2, pp. 214-220, 2016, doi: 10.1038/ng.3477.

[37] E. V. Davydov, D. L. Goode, M. Sirota, G. M. Cooper, A. Sidow, and S. Batzoglou, "Identifying a high fraction of the human genome to be under selective constraint using GERP++," (in eng), *PLoS Comput Biol,* vol. 6, no. 12, p. e1001025, Dec 2 2010, doi: 10.1371/journal.pcbi.1001025.

[38] W. Burke, E. Parens, W. K. Chung, S. M. Berger, and P. S. Appelbaum, "The Challenge of Genetic Variants of Uncertain Clinical Significance : A Narrative Review," *Ann Intern Med,* vol. 175, no. 7, pp. 994-1000, Jul 2022, doi: 10.7326/M21-4109.

[39] S. J. MacEachern and N. D. Forkert, "Machine learning for precision medicine," *Genome,* vol. 64, no. 4, pp. 416-425, Apr 2021, doi: 10.1139/gen-2020-0131.

[40] I. A. Adzhubei *et al.*, "A method and server for predicting damaging missense mutations," *Nature Methods,* vol. 7, no. 4, pp. 248-249, 2010/04/01 2010, doi: 10.1038/nmeth0410-248.

[41] D. M. Jordan *et al.*, "Development and validation of a computational method for assessment of missense variants in hypertrophic cardiomyopathy," *Am J Hum Genet,* vol. 88, no. 2, pp. 183-92, Feb 11 2011, doi: 10.1016/j.ajhg.2011.01.011.

[42] J. S. Kaminker, Y. Zhang, C. Watanabe, and Z. Zhang, "CanPredict: a computational tool for predicting cancer-associated missense mutations," *Nucleic Acids Res,* vol. 35, no. Web Server issue, pp. W595-8, Jul 2007, doi: 10.1093/nar/gkm405.

[43] H. Y. Xiong *et al.*, "RNA splicing. The human splicing code reveals new insights into the genetic determinants of disease," (in eng), *Science,* vol. 347, no. 6218, p. 1254806, Jan 9 2015, doi: 10.1126/science.1254806.

[44] E. Capriotti, R. Calabrese, and R. Casadio, "Predicting the insurgence of human genetic diseases associated to single point protein mutations with support vector machines and evolutionary information," *Bioinformatics,* vol. 22, no. 22, pp. 2729-2734, 2006, doi: 10.1093/bioinformatics/btl423.

[45] H. Carter, C. Douville, P. D. Stenson, D. N. Cooper, and R. Karchin, "Identifying Mendelian disease genes with the Variant Effect Scoring Tool," *BMC Genomics,* vol. 14, no. S3, 2013, doi: 10.1186/1471-2164-14-s3-s3.

[46] C. Dong *et al.*, "Comparison and integration of deleteriousness prediction methods for nonsynonymous SNVs in whole exome sequencing studies," *Human Molecular Genetics,* vol. 24, no. 8, pp. 2125-2137, 2014, doi: 10.1093/hmg/ddu733.

[47] B. Li *et al.*, "Automated inference of molecular mechanisms of disease from amino acid substitutions," *Bioinformatics,* vol. 25, no. 21, pp. 2744-50, Nov 1 2009, doi: 10.1093/bioinformatics/btp528.

[48] M. Fang, Z. Su, H. Abolhassani, Y. Itan, X. Jin, and L. Hammarstrom, "VIPPID: a gene-specific single nucleotide variant pathogenicity prediction tool for primary immunodeficiency diseases," *Brief Bioinform,* vol. 23, no. 5, Sep 20 2022, doi: 10.1093/bib/bbac176.

[49] B. Zeng, D. C. Liu, J. G. Huang, X. B. Xia, and B. Qin, "PdmIRD: missense variants pathogenicity prediction for inherited retinal diseases in a disease-specific manner," *Hum Genet,* vol. 143, no. 3, pp. 331-342, Mar 2024, doi: 10.1007/s00439-024-02645-6.

[50] S. Li *et al.*, "CAPICE: a computational method for Consequence-Agnostic Pathogenicity Interpretation of Clinical Exome variations," *Genome Medicine,* vol. 12, no. 1, 2020, doi: 10.1186/s13073-020-00775-w.

[51] Y. Wei *et al.*, "INDELpred: Improving the prediction and interpretation of indel pathogenicity within the clinical genome," (in eng), *HGG Adv,* vol. 5, no. 4, p. 100325, Oct 10 2024, doi: 10.1016/j.xhgg.2024.100325.

[52] K. A. Jagadeesh *et al.*, "M-CAP eliminates a majority of variants of uncertain significance in clinical exomes at high sensitivity," *Nature Genetics,* vol. 48, no. 12, pp. 1581-1586, 2016, doi: 10.1038/ng.3703.

[53] M. Kabir, S. Ahmed, H. Zhang, I. Rodríguez-Rodríguez, S. M. Najibi, and M. Vihinen, "PON-P3: Accurate Prediction of Pathogenicity of Amino Acid Substitutions," (in eng), *Int J Mol Sci,* vol. 26, no. 5, Feb 25 2025, doi: 10.3390/ijms26052004.

[54] X. Zhang *et al.*, "Disease-specific variant pathogenicity prediction significantly improves variant interpretation in inherited cardiac conditions," *Genet Med,* vol. 23, no. 1, pp. 69-79, Jan 2021, doi: 10.1038/s41436-020-00972-3.

[55] A. C. Gunning *et al.*, "Assessing performance of pathogenicity predictors using clinically relevant variant datasets," *J Med Genet,* vol. 58, no. 8, pp. 547-555, Aug 2021, doi: 10.1136/jmedgenet-2020-107003.

[56] S. Whalen and G. Pandey, "A Comparative Analysis of Ensemble Classifiers: Case Studies in Genomics," in *2013 IEEE 13th International Conference on Data Mining*, 7-10 Dec. 2013 2013, pp. 807-816, doi: 10.1109/ICDM.2013.21.

[57] N. M. Ioannidis *et al.*, "REVEL: An Ensemble Method for Predicting the Pathogenicity of Rare Missense Variants," *Am J Hum Genet,* vol. 99, no. 4, pp. 877-885, Oct 6 2016, doi: 10.1016/j.ajhg.2016.08.016.

[58] A. Petrini *et al.*, "parSMURF, a high-performance computing tool for the genome-wide detection of pathogenic variants," (in eng), *Gigascience,* vol. 9, no. 5, May 1 2020, doi: 10.1093/gigascience/giaa052.

[59] J. Zou, M. Huss, A. Abid, P. Mohammadi, A. Torkamani, and A. Telenti, "A primer on deep learning in genomics," *Nat Genet,* vol. 51, no. 1, pp. 12-18, Jan 2019, doi: 10.1038/s41588-018-0295-5.

[60] D. Quang, Y. Chen, and X. Xie, "DANN: a deep learning approach for annotating the pathogenicity of genetic variants," *Bioinformatics,* vol. 31, no. 5, pp. 761-3, Mar 1 2015, doi: 10.1093/bioinformatics/btu703.

[61] L. Sundaram *et al.*, "Predicting the clinical impact of human mutation with deep neural networks," *Nature Genetics,* vol. 50, no. 8, pp. 1161-1170, 2018, doi: 10.1038/s41588-018-0167-z.

[62] J. Zhou and O. G. Troyanskaya, "Predicting effects of noncoding variants with deep learning-based sequence model," *Nat Methods,* vol. 12, no. 10, pp. 931-4, Oct 2015, doi: 10.1038/nmeth.3547.

[63] D. R. Kelley, Y. A. Reshef, M. Bileschi, D. Belanger, C. Y. McLean, and J. Snoek, "Sequential regulatory activity prediction across chromosomes with convolutional neural networks," (in eng), *Genome Res,* vol. 28, no. 5, pp. 739-750, May 2018, doi: 10.1101/gr.227819.117.

[64] K. Jaganathan *et al.*, "Predicting Splicing from Primary Sequence with Deep Learning," *Cell,* vol. 176, no. 3, pp. 535-548.e24, 2019, doi: 10.1016/j.cell.2018.12.015.

[65] J. Cheng *et al.*, "MMSplice: modular modeling improves the predictions of genetic variant effects on splicing," *Genome Biology,* vol. 20, no. 1, p. 48, 2019/03/01 2019, doi: 10.1186/s13059-019-1653-z.

[66] D. Danis *et al.*, "Interpretable prioritization of splice variants in diagnostic next-generation sequencing," *The American Journal of Human Genetics,* vol. 108, no. 9, pp. 1564-1577, 2021, doi: 10.1016/j.ajhg.2021.06.014.

[67] Y. LeCun, Y. Bengio, and G. Hinton, "Deep learning," *Nature,* vol. 521, no. 7553, pp. 436-444, 2015/05/01 2015, doi: 10.1038/nature14539.

[68] J. Cheng *et al.*, "Accurate proteome-wide missense variant effect prediction with AlphaMissense," *Science,* vol. 381, no. 6664, p. eadg7492, Sep 22 2023, doi: 10.1126/science.adg7492.

[69] A. Vaswani *et al.*, "Attention Is All You Need," 06/12 2017, doi: 10.48550/arXiv.1706.03762.

[70] A. Rives *et al.*, "Biological structure and function emerge from scaling unsupervised learning to 250 million protein sequences," (in eng), *Proc Natl Acad Sci U S A,* vol. 118, no. 15, Apr 13 2021, doi: 10.1073/pnas.2016239118.

[71] N. Brandes, G. Goldman, C. H. Wang, C. J. Ye, and V. Ntranos, "Genome-wide prediction of disease variant effects with a deep protein language model," *Nature Genetics,* vol. 55, no. 9, pp. 1512-1522, 2023/09/01 2023, doi: 10.1038/s41588-023-01465-0.

[72] W. Lin, J. Wells, Z. Wang, C. Orengo, and A. C. R. Martin, "Enhancing missense variant pathogenicity prediction with protein language models using VariPred," *Sci Rep,* vol. 14, no. 1, p. 8136, Apr 7 2024, doi: 10.1038/s41598-024-51489-7.

[73] A. V. Kulikova, D. J. Diaz, T. Chen, T. J. Cole, A. D. Ellington, and C. O. Wilke, "Two sequence- and two structure-based ML models have learned different aspects of protein biochemistry," *bioRxiv,* p. 2023.03.20.533508, 2023, doi: 10.1101/2023.03.20.533508.

[74] C. Marquet *et al.*, "Embeddings from protein language models predict conservation and variant effects," *Human Genetics,* vol. 141, no. 10, pp. 1629-1647, 2022/10/01 2022, doi: 10.1007/s00439-021-02411-y.

[75] Ž. Avsec *et al.*, "Effective gene expression prediction from sequence by integrating long-range interactions," *Nature Methods,* vol. 18, no. 10, pp. 1196-1203, 2021/10/01 2021, doi: 10.1038/s41592-021-01252-x.

[76] Ž. Avsec *et al.*, "AlphaGenome: advancing regulatory variant effect prediction with a unified DNA sequence model," *bioRxiv,* p. 2025.06.25.661532, 2025, doi: 10.1101/2025.06.25.661532.

[77] K. Jaganathan *et al.*, "Predicting expression-altering promoter mutations with deep learning," *Science,* vol. 0, no. 0, p. eads7373, doi: doi:10.1126/science.ads7373.

[78] J. Jumper *et al.*, "Highly accurate protein structure prediction with AlphaFold," *Nature,* vol. 596, no. 7873, pp. 583-589, 2021/08/01 2021, doi: 10.1038/s41586-021-03819-2.

[79] T. T. Jiang, L. Fang, and K. Wang, "Deciphering “the language of nature”: A transformer-based language model for deleterious mutations in proteins," *The Innovation,* vol. 4, no. 5, p. 100487, 2023/09/11/ 2023, doi: https://doi.org/10.1016/j.xinn.2023.100487.

[80] L. Liang *et al.*, "Genetic Transformer: An Innovative Large Language Model Driven Approach for Rapid and Accurate Identification of Causative Variants in Rare Genetic Diseases," *medRxiv,* p. 2024.07.18.24310666, 2024, doi: 10.1101/2024.07.18.24310666.

[81] N. You *et al.*, "SpliceTransformer predicts tissue-specific splicing linked to human diseases," (in eng), *Nat Commun,* vol. 15, no. 1, p. 9129, Oct 23 2024, doi: 10.1038/s41467-024-53088-6.

[82] G. R. Ritchie, I. Dunham, E. Zeggini, and P. Flicek, "Functional annotation of noncoding sequence variants," (in eng), *Nat Methods,* vol. 11, no. 3, pp. 294-6, Mar 2014, doi: 10.1038/nmeth.2832.

[83] J. Luo *et al.*, "Assessing concordance among human, in silico predictions and functional assays on genetic variant classification," *Bioinformatics,* vol. 35, no. 24, pp. 5163-5170, 2019, doi: 10.1093/bioinformatics/btz442.

[84] J. Frazer *et al.*, "Disease variant prediction with deep generative models of evolutionary data," *Nature,* vol. 599, no. 7883, pp. 91-95, Nov 2021, doi: 10.1038/s41586-021-04043-8.

[85] Z. Gao, Q. Liu, W. Zeng, R. Jiang, and W. H. Wong, "EpiGePT: a Pretrained Transformer model for epigenomics," *bioRxiv,* p. 2023.07.15.549134, 2024, doi: 10.1101/2023.07.15.549134.

[86] W. McLaren *et al.*, "The Ensembl Variant Effect Predictor," *Genome Biology,* vol. 17, no. 1, p. 122, 2016/06/06 2016, doi: 10.1186/s13059-016-0974-4.

[87] K. Wang, M. Li, and H. Hakonarson, "ANNOVAR: functional annotation of genetic variants from high-throughput sequencing data," (in eng), *Nucleic Acids Res,* vol. 38, no. 16, p. e164, Sep 2010, doi: 10.1093/nar/gkq603.

[88] C. Kopanos *et al.*, "VarSome: the human genomic variant search engine," (in eng), *Bioinformatics,* vol. 35, no. 11, pp. 1978-1980, Jun 1 2019, doi: 10.1093/bioinformatics/bty897.

[89] D. Baux *et al.*, "MobiDetails: online DNA variants interpretation," *European Journal of Human Genetics,* vol. 29, no. 2, pp. 356-360, 2021/02/01 2021, doi: 10.1038/s41431-020-00755-z.

[90] Q. Li and K. Wang, "InterVar: Clinical Interpretation of Genetic Variants by the 2015 ACMG-AMP Guidelines," *The American Journal of Human Genetics,* vol. 100, no. 2, pp. 267-280, 2017, doi: 10.1016/j.ajhg.2017.01.004.

[91] P. N. Robinson *et al.*, "Improved exome prioritization of disease genes through cross-species phenotype comparison," (in eng), *Genome research,* vol. 24, no. 2, pp. 340-348, 2014/02// 2014, doi: 10.1101/gr.160325.113.

[92] K. Maharana, S. Mondal, and B. Nemade, "A review: Data pre-processing and data augmentation techniques," *Global Transitions Proceedings,* vol. 3, no. 1, pp. 91-99, 2022/06/01/ 2022, doi: https://doi.org/10.1016/j.gltp.2022.04.020.

[93] A. Pfob, S.-C. Lu, and C. Sidey-Gibbons, "Machine learning in medicine: a practical introduction to techniques for data pre-processing, hyperparameter tuning, and model comparison," *BMC Medical Research Methodology,* vol. 22, no. 1, p. 282, 2022/11/01 2022, doi: 10.1186/s12874-022-01758-8.

[94] C. Marzban, "The ROC Curve and the Area under It as Performance Measures," (in English), *Weather and Forecasting,* vol. 19, no. 6, pp. 1106-1114, 01 Dec. 2004 2004, doi: https://doi.org/10.1175/825.1.

[95] P. Krusche *et al.*, "Best practices for benchmarking germline small-variant calls in human genomes," (in eng), *Nat Biotechnol,* vol. 37, no. 5, pp. 555-560, May 2019, doi: 10.1038/s41587-019-0054-x.

[96] M. Zitnik, F. Nguyen, B. Wang, J. Leskovec, A. Goldenberg, and M. M. Hoffman, "Machine Learning for Integrating Data in Biology and Medicine: Principles, Practice, and Opportunities," (in eng), *Inf Fusion,* vol. 50, pp. 71-91, Oct 2019, doi: 10.1016/j.inffus.2018.09.012.

[97] P. Charilaou and R. Battat, "Machine learning models and over-fitting considerations," (in eng), *World J Gastroenterol,* vol. 28, no. 5, pp. 605-607, Feb 7 2022, doi: 10.3748/wjg.v28.i5.605.

[98] L. Hoffman-Andrews, "The known unknown: the challenges of genetic variants of uncertain significance in clinical practice," (in eng), *J Law Biosci,* vol. 4, no. 3, pp. 648-657, Dec 2017, doi: 10.1093/jlb/lsx038.

[99] T. Schnabel, A. Swaminathan, A. Singh, N. Chandak, and T. Joachims, "Recommendations as Treatments: Debiasing Learning and Evaluation," in *International Conference on Machine Learning*, 2016.

[100] R. Karimi, C. Freudenthaler, A. Nanopoulos, and L. Schmidt-Thieme, "Towards Optimal Active Learning for Matrix Factorization in Recommender Systems," in *2011 IEEE 23rd International Conference on Tools with Artificial Intelligence*, 7-9 Nov. 2011 2011, pp. 1069-1076, doi: 10.1109/ICTAI.2011.182.

[101] M. Gasperini, L. Starita, and J. Shendure, "The power of multiplexed functional analysis of genetic variants," (in eng), *Nat Protoc,* vol. 11, no. 10, pp. 1782-7, Oct 2016, doi: 10.1038/nprot.2016.135.

[102] A. E. McEwen, M. Tejura, S. Fayer, L. M. Starita, and D. M. Fowler, "Multiplexed assays of variant effect for clinical variant interpretation," (in eng), *Nat Rev Genet,* Jul 21 2025, doi: 10.1038/s41576-025-00870-x.

[103] L. G. Landry, N. Ali, D. R. Williams, H. L. Rehm, and V. L. Bonham, "Lack Of Diversity In Genomic Databases Is A Barrier To Translating Precision Medicine Research Into Practice," *Health Aff (Millwood),* vol. 37, no. 5, pp. 780-785, May 2018, doi: 10.1377/hlthaff.2017.1595.

[104] A. R. Martin, M. Kanai, Y. Kamatani, Y. Okada, B. M. Neale, and M. J. Daly, "Clinical use of current polygenic risk scores may exacerbate health disparities," *Nat Genet,* vol. 51, no. 4, pp. 584-591, Apr 2019, doi: 10.1038/s41588-019-0379-x.

[105] L. A. Hindorff, V. L. Bonham, and L. Ohno-Machado, "Enhancing diversity to reduce health information disparities and build an evidence base for genomic medicine," *Per Med,* vol. 15, no. 5, pp. 403-412, Sep 2018, doi: 10.2217/pme-2018-0037.

[106] M. Dawood *et al.*, "Using multiplexed functional data to reduce variant classification inequities in underrepresented populations," *Genome Medicine,* vol. 16, no. 1, p. 143, 2024/12/03 2024, doi: 10.1186/s13073-024-01392-7.

[107] A. Raz, B. Heinrichs, N. Avnoon, G. Eyal, and Y. Inbar, "Prediction and explainability in AI: Striking a new balance?," *Big Data & Society,* vol. 11, no. 1, p. 20539517241235871, 2024, doi: 10.1177/20539517241235871.

[108] Z. Zheng *et al.*, "Attention heads of large language models," *Patterns (N Y),* vol. 6, no. 2, p. 101176, Feb 14 2025, doi: 10.1016/j.patter.2025.101176.

[109] I. R. Mallela, S. Aravind, O. Tharan, D. P. Goel, D. S. Pal, and Singh, "Explainable AI for Compliance and Regulatory Models," *International Journal for Research Publication and Seminar,* 2020.

[110] I. Subramanian, S. Verma, S. Kumar, A. Jere, and K. Anamika, "Multi-omics Data Integration, Interpretation, and Its Application," (in eng), *Bioinform Biol Insights,* vol. 14, p. 1177932219899051, 2020, doi: 10.1177/1177932219899051.

[111] T. Yang, P. Wei, and W. Pan, "Integrative analysis of multi-omics data for discovering low-frequency variants associated with low-density lipoprotein cholesterol levels," *Bioinformatics,* vol. 36, no. 21, pp. 5223-5228, 2020, doi: 10.1093/bioinformatics/btaa898.

[112] N. Ding *et al.*, "Parameter-efficient fine-tuning of large-scale pre-trained language models," *Nature Machine Intelligence,* vol. 5, no. 3, pp. 220-235, 2023/03/01 2023, doi: 10.1038/s42256-023-00626-4.

[113] L. Wang *et al.*, "Prompt engineering in consistency and reliability with the evidence-based guideline for LLMs," *npj Digital Medicine,* vol. 7, no. 1, p. 41, 2024/02/20 2024, doi: 10.1038/s41746-024-01029-4.

[114] I. Alfisi, F. Ciapi, M. Baragli, and A. Magi, "Benchmarking DNA Foundation Models for zero-shot variant effect prediction: the role of context, training, and architecture," *bioRxiv,* p. 2025.06.15.659748, 2025, doi: 10.1101/2025.06.15.659748.

[115] A. R. Sajun, I. Zualkernan, and D. Sankalpa, "A historical survey of advances in transformer architectures," *Applied Sciences,* vol. 14, no. 10, p. 4316, 2024.

[116] D. M. Fowler *et al.*, "An Atlas of Variant Effects to understand the genome at nucleotide resolution," *Genome Biology,* vol. 24, no. 1, p. 147, 2023/07/03 2023, doi: 10.1186/s13059-023-02986-x.

[117] A. F. Rubin *et al.*, "MaveDB 2024: a curated community database with over seven million variant effects from multiplexed functional assays," *Genome Biology,* vol. 26, no. 1, p. 13, 2025/01/21 2025, doi: 10.1186/s13059-025-03476-y.

[118] P. Buphamalai, T. Kokotovic, V. Nagy, and J. Menche, "Network analysis reveals rare disease signatures across multiple levels of biological organization," *Nature Communications,* vol. 12, no. 1, p. 6306, 2021/11/09 2021, doi: 10.1038/s41467-021-26674-1.

[119] C. Dandara *et al.*, "H3Africa and the African life sciences ecosystem: building sustainable innovation," (in eng), *Omics,* vol. 18, no. 12, pp. 733-9, Dec 2014, doi: 10.1089/omi.2014.0145.

Figure Legends

Figure 1. Evolution of *in silico* Tools for Predicting Genetic Variant Pathogenicity

This figure illustrates the temporal progression of computational approaches for genetic variant pathogenicity prediction. The evolutionary trajectory is categorized into four phases: (1) Early Stages, characterized by rudimentary rule-based algorithms; (2) The Emergence of Predictive Tools, marking the transition to more sophisticated statistical frameworks; (3) Machine Learning Era, defined by the integration of supervised and unsupervised learning methodologies; and (4) Advanced AI, representing contemporary approaches that leverage deep learning architectures and multi-modal data integration.

Figure 2. Integrated Pipeline for Variant Pathogenicity Prediction

This figure delineates a systematic framework for developing variant pathogenicity prediction models. The pipeline harnesses multi-modal data inputs (genomic, proteomic, molecular, and clinical) acquired from public repositories, experimental assays, clinical documentation, and literature curation. Critical preprocessing steps include variant annotation, normalization, cross-platform integration, and feature engineering to enhance signal integrity. The analytical workflow culminates in a machine learning implementation with rigorous hyperparameter optimization and comprehensive performance assessment to ensure robust predictive capacity across diverse genetic contexts.

Figure 3. Transformer-Based Framework for Variant Pathogenicity Prediction

This figure illustrates a transformer-based pipeline for predicting genetic variant pathogenicity. Input sequences ($X \in R^{B\times L\times D}$, where X represents the input tensor, R denotes real numbers, B = batch size, L = sequence length, D = embedding dimension) are processed through three key stages: (1) tokenization and embedding, (2) contextual encoding via multi-head self-attention, and (3) classification. The model undergoes pre-training on multi-omics datasets followed by pathogenicity-specific fine-tuning. Prompt engineering facilitates performance with limited

labeled data. Other key components include LayerNorm to mitigate vanishing or exploding gradients, Fully Connected Layers for effective feature extraction, and Softmax activation for generating robust probability distributions. Together, the overall architecture and training strategies enable the model to leverage latent biological mechanisms for accurate variant classification.

Table 1. Genomic Database Overview

No.	Name	Description	Data Types	Cross-link	Entries (as of September 2025)	Last Updates	Website	Reference
1	1KGP	Large-scale project to create a comprehensive resource on human genetic variation.	Population data	Uses refID from dbSNP; variants included in gnomAD	>88 million variants (84.7M SNPs, 3.6M indels, 60k SVs)	2024-11-18	https://www.internationalgenome.org/	[7]
2	BRCA1 Dataset	Focused dataset on SNVs in the BRCA1 gene.	Genomic data	Variants cross-validated with ClinVar and HGMD	3.893 SNVs	2018-08-20	https://sge.gs.washington.edu/BRCA1/	[22]
3	ClinGen	Clinical genomics resource defining the clinical relevance of genes and variants.	Genomic data	Provides gene-disease evidence to ClinVar; uses HPO and OMIM	3.256 genes, 11.062 variants	2025-09-18	https://clinicalgenome.org/	[14]
4	ClinVar	Public archive of reports of the relationships among human variations and phenotypes.	Genomic data	Integrates rsID from dbSNP; links to ClinGen, OMIM, HPO	5.640.148 records (3.759.476 unique variants)	2025-08-24	https://www.ncbi.nlm.nih.gov/clinvar/	[11]
5	COSMIC	Catalogue of somatic mutations in cancer.	Genomic data	Overlaps with DoCM and ClinVar	25.014.261 variants	2025-05-21	https://cancer.sanger.ac.uk/cosmic	[19]
6	dbSNP	Database of Single Nucleotide Polymorphisms and other variants.	Genomic data	Referenced by ClinVar and gnomAD via rsID	1.206.053.617 unique rs	2025-01-15	https://www.ncbi.nlm.nih.gov/snp/	[6]
7	DoCM	Manually curated database of clinically relevant mutations.	Genomic data	Overlaps with COSMIC and ClinVar	3.818 variants	2024-10-15	http://www.docm.info/	[20]
8	gnomAD	Aggregated and harmonized human exome and genome sequencing data.	Population data	Uses rsID from dbSNP, referenced in ClinVar	730.947 exomes and 76.215 whole genomes	2024-04-19	https://gnomad.broadinstitute.org/	[8]
9	GO	Structured vocabulary for gene product functions and processes.	Ontology	Used in UniProt	39.906 terms, 9.41 M annotations, 1.60 M gene products, 5.497 species	2025-07-22	http://www.geneontology.org/	[18]
10	HGMD	Comprehensive collection of germline mutations in human genes.	Genomic data	Often cross-validated with ClinVar and OMIM	549.178 mutations	2025-07-07	http://www.hgmd.cf.ac.uk/ac/ index.php	[12]

No.	Name	Description	Data Types	Cross-link	Entries (as of September 2025)	Last Updates	Website	Reference
11	HPO	Ontology for describing human phenotypic abnormalities.	Phenotype & Genotype data	Used by OMIM and ClinGen; mapped to GO	18.000 terms and over 156.000 annotations	2024-04-19	https://hpojaxo rg/	[17]
12	HuVarBa se	Annotated human variation database.	Genomic data	Merged from COSMIC, ClinVar, 1000 Genomes	774.863 variants from 18.318 proteins (702.048 disease causing and 72.815 neutral variants)	2018-06-01	https://www.ii tm.ac.in/bioinf o/huvarbase	[13]
13	OMIM	Comprehensive catalog of human genes and genetic phenotypes.	Phenotype & Genotype data	Includes HPO annotations; links to ClinVar and UniProt	27.938 entries (26.394 Autosomal, 1.407 X Linked, 64 Y Linked, 73 Mitochondrial)	2025-09-17	https://www.o mim.org/	[15]
14	ONGene	Curated database of human oncogenes.	Protein data	-	803 oncogenes (698 protein-coding genes + 105 non-coding)	2016-12-26	https://ongene. bioinfo-minzhao.org/	[21]
15	Orphanet	Database for rare diseases and orphan drugs	Ontology	Shares HPO and OMIM terms	9.785 Clinical entities 6.528 Rare disorders 8.296 Disease gene relationships 115.611 Phenotypic annotations 16.418 Epidemiological data 689 Orphan drugs 8.648 Expert centers	2025-06-24	https://www.or pha.net/	[16]
16	PDB	Repository for 3D structural data of biological macromolecules.	Protein data	Linked from UniProt entries	242.296 structures,1.068.577 computed structure models	2025-09-17	https://www.rc sb.org/	[24]
17	UK Biobank	Large-scale biomedical database with genetic and health information.	Population data	Overlaps with dbSNP	90 million variants	2024-08-13	https://www.u kbiobank.ac.u k/	[9]
18	UniProt / UniProt KB	Comprehensive protein sequence and annotation resource.	Protein data	Cross-links to PDB, GO, dbSNP, ClinGen, Orphanet,	253.635.358 (573.661 Swiss-Prot) 253.206.171 entries (UniProtKB/Swiss-Prot:	2025-06-17	https://www.u niprot.org/	[23]

No.	Name	Description	Data Types	Cross-link	Entries (as of September 2025)	Last Updates	Website	Reference
					572.970 entries and UniProtKB/TrEMBL: 252.633.201 entries)			
19	VarCards 2	Updated version of VarCards, human variant annotation and interpretation.	Genomic data	Some extracted from gnomAD, ClinVar, COSMIC, dbSNP	368.820.266 indels, 2.773.555 CNVs	2023-10-21	http://www.genemed.tech/varcards2/	[27]
20	VariBench	Benchmark database for variation datasets in bioinformatics.	Genomic data	Some extracted from dbSNP, OMIM	>90 million variants	2023-05-12	https://structure.bmc.lu.se/VariBench/	[25]
21	VariSNP	A benchmark database suite comprising variation datasets that can be used for developing and testing the performance of variant effect prediction	Genomic Data	Selected from dbSNP	30.571.777 variants	2017-02-16	https://structure.bmc.lu.se/VariSNP/	[26]

Click here to access/download;Figure;Figures_5 Nov 2025.pdf

Figure 1. Evolution of In Silico Tools for Predicting Genetic Variant Pathogenicity

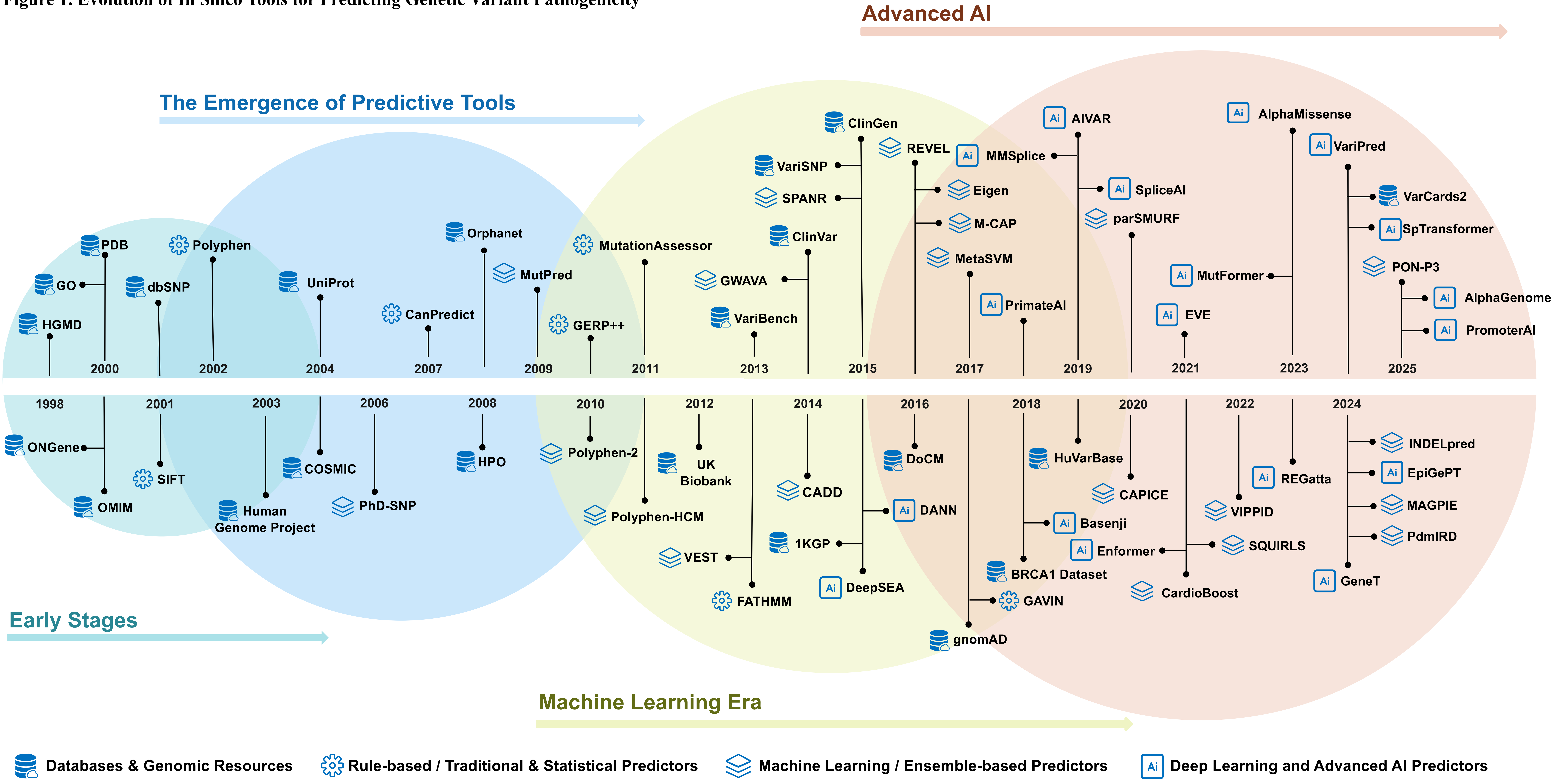

Figure 2. Integrated Pipeline for Variant Pathogenicity Prediction

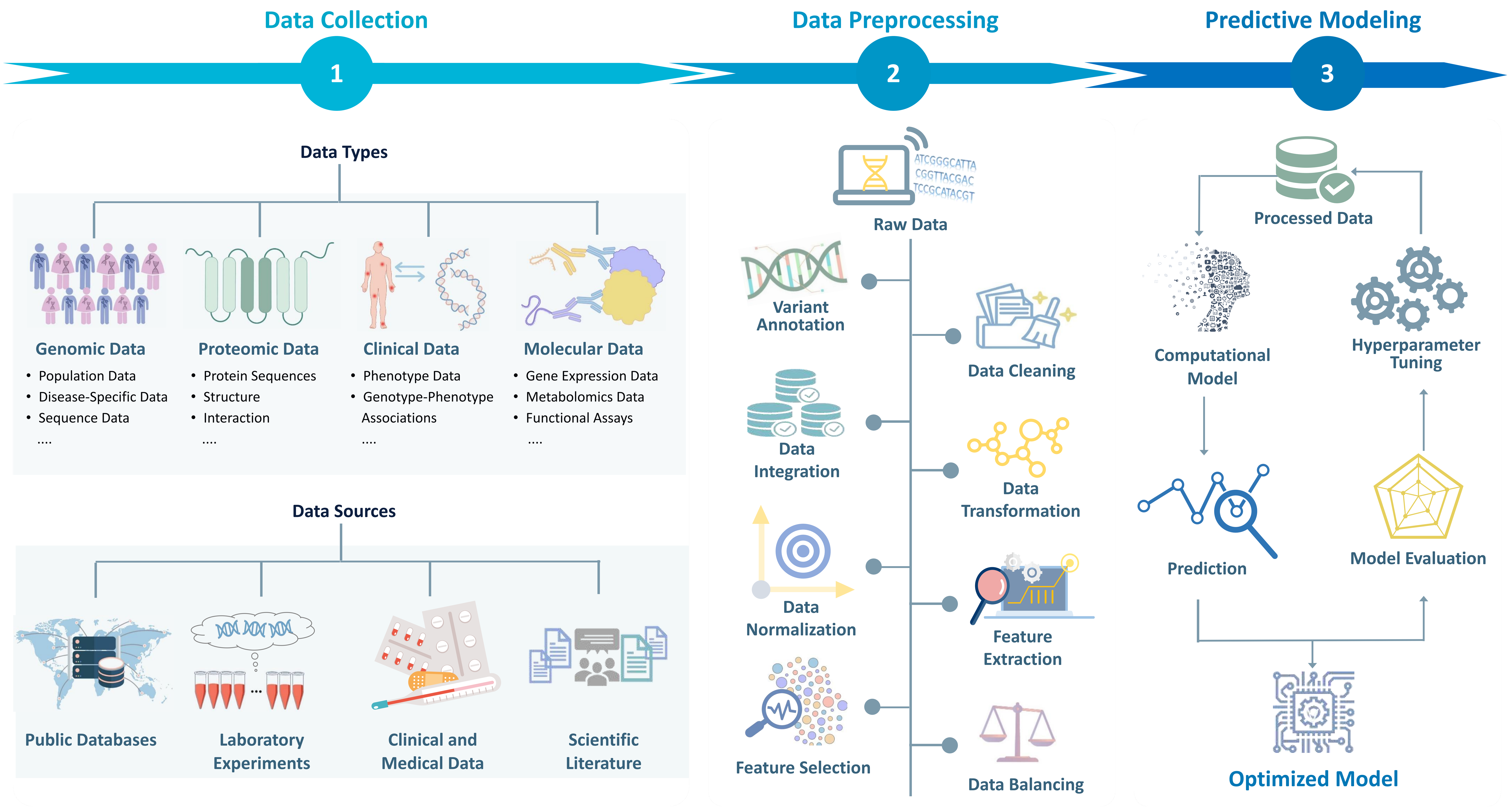

Figure 3. Transformer-Based Framework for Variant Pathogenicity Prediction.

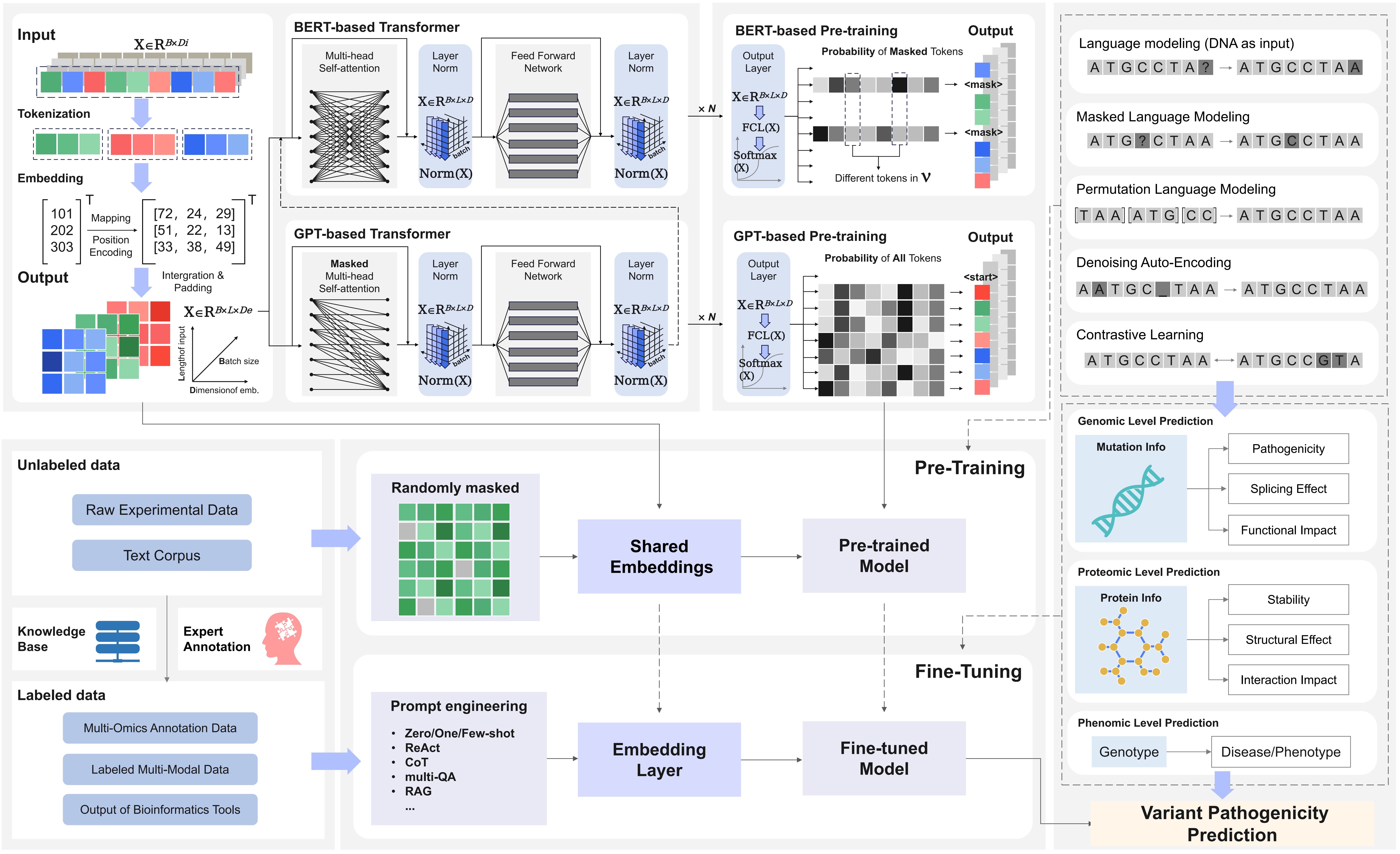

Supplementary Table 1

Click here to access/download
Supplementary Material
Supplementary Table 1_Supplementary Material.docx
