# Supplementary material for: Harnessing artificial intelligence for genomic variant prediction: advances, challenges, and future directions
Source: Gigascience. 2026 Jan 10;15:giag004. doi: 10.1093/gigascience/giag004 (PMC12888390; doi:10.1093/gigascience/giag004)
Supplement: giag004_GIGA-D-25-00463_Revision_1 [file giag004_giga-d-25-00463_revision_1.pdf]

GigaScience

# Harnessing Artificial Intelligence for Genomic Variant Prediction: Advances, Challenges, and Future Directions

--Manuscript Draft--

| | |
|---|---|
| **Manuscript Number:** | GIGA-D-25-00463R1 |
| **Full Title:** | Harnessing Artificial Intelligence for Genomic Variant Prediction: Advances, Challenges, and Future Directions |
| **Article Type:** | Review |
| **Funding Information:** | |
| **Abstract:** | Accurate genetic variant interpretation is crucial for disease research and the development of targeted therapies. Artificial intelligence (AI) is transforming this field by integrating computational methodologies across structural biology, evolutionary analysis, and multimodal genomic data. This review examines the evolution from traditional rule-based systems and statistical models to contemporary machine learning, deep learning, and protein language models, while addressing critical challenges in variant classification. Key obstacles include data heterogeneity, interpretability, and the persistence of Variants of Uncertain Significance (VUS), emphasizing the critical need for explainable AI frameworks and more inclusive genomic databases to improve predictive accuracy across diverse populations. Based on the assessment of current Variant Impact Predictors (VIPs), we propose strategies for enhanced predictor selection, effective multiomics data integration, and optimized computational workflows. These recommendations aim to enhance variant interpretation accuracy in both research settings and clinical practice, ultimately contributing to advances in personalized medicine. |
| **Corresponding Author:** | Mingyan Fang<br>BGI-Shenzhen: BGI Group<br>Shenzhen, China CHINA |
| **Corresponding Author Secondary Information:** | |
| **Corresponding Author's Institution:** | BGI-Shenzhen: BGI Group |
| **Corresponding Author's Secondary Institution:** | |
| **First Author:** | Indah Pakpahan |
| **First Author Secondary Information:** | |
| **Order of Authors:** | Indah Pakpahan |
| | Mentari Sihombing |
| | Haohan Liu |
| | Mengyao Wang |
| | Zheng Su |
| | Mingyan Fang |
| **Order of Authors Secondary Information:** | |
| **Response to Reviewers:** | Dear Dr. Ma,<br>Thank you for the opportunity to revise our manuscript based on the thoughtful feedback provided by the expert reviewer. We have carefully considered all comments and suggestions and have made substantial revisions to address each point raised.<br>A summary of the key revisions is provided below:<br>1.Moderated claims regarding transformer performance to emphasize context-dependent gains, and added a new “Implications and Limitations” section separating evidence-based conclusions from future directions.<br>2.Enhanced quantitative context through a new comparative table (Table 3) |

Powered by Editorial Manager® and ProduXion Manager® from Aries Systems Corporation

summarizing representative models and their reported performance metrics, with explicit caution regarding cross-study comparability.
3.Clarified performance reporting across methodological phases, justifying our emphasis on protein language models while avoiding potentially misleading comparisons.
4.Expanded discussion of ancestry bias, incorporating algorithmic strategies such as transfer learning, domain adaptation, parameter-efficient fine-tuning, fairness-aware optimization, and ancestry-aware embeddings.
5.Addressed limitations for complex traits, clarifying constraints of single-variant predictors and expanding future directions toward polygenic modeling approaches.
6.Refined the Background section to clearly distinguish established knowledge, unresolved challenges, and the novel contribution of this review.
7.Improved search strategy transparency, including verification searches in additional databases.
8.Expanded discussion of the clinician's role, emphasizing AI as decision-support within established diagnostic workflows.
9.Strengthened the multi-omics integration discussion, highlighting selective, context-dependent integration strategies alongside practical implementation.
10.Clarified the interpretation of novel variants, distinguishing variant discovery from AI-based interpretation of rare and unannotated variants.
11.Explicitly addressed erroneous predictions, emphasizing probabilistic outputs, explainable AI approaches, and the need for orthogonal validation.
We believe these revisions have further strengthened the manuscript and addressed all reviewer concerns comprehensively. Thank you for considering this revised version for publication. We look forward to your response.

Yours sincerely
Mingyan Fang on behalf of all authors

Reviewer #1:
This manuscript provides a comprehensive and well-structured review of artificial intelligence methods for genomic variant pathogenicity prediction. It effectively traces the evolution from rule-based systems to transformer architectures and integrates discussion of databases, model interpretability, and clinical translation. The topic is timely and relevant to the GigaScience community. I have few comments:
Author Response: We sincerely thank you for the thoughtful evaluation and positive assessment of our manuscript. We are pleased that you find our work comprehensive, timely, and relevant to the GigaScience community. We have carefully addressed all comments raised and believe the revisions have strengthened the manuscript substantially, with detailed point-by-point responses provided below.
1)The discussion is insightful but should moderate claims such as "transformer architectures consistently outperform prior methods. Consider adding a brief "Implications and Limitations" section to clearly separate evidence-based conclusions from forward-looking speculation.
Author Response: We appreciate this constructive feedback, which has prompted us to examine the nuance of our performance claims more carefully. While the comparative advantages of transformer architectures are well-documented across multiple studies, the magnitude and consistency of these improvements vary substantially depending on variant type, dataset composition, and evaluation framework. We agree that more explicit acknowledgment of context-dependency and benchmarking variability would enhance the manuscript's precision.
We have revised the manuscript to moderate claims regarding transformer performance through two main approaches:
1)Refined performance characterizations throughout the manuscript to explicitly acknowledge context-dependency. We replaced categorical statements about consistent superiority with more nuanced phrasing that recognizes variability across variant types, dataset compositions, and evaluation metrics. These revisions appear primarily in Section 3 “The Technological Trajectory of Variant Pathogenicity Assessment” where transformer architectures are discussed (Page 12, Lines 268-273).
2)Added an “Implications and Limitations” subsection (Pages 22-23, Lines 489-504) that highlights key considerations for interpreting reported benchmark results, including limitations in cross-study comparability, validation, interpretability, and population representation. This section also clarifies that AI-based variant interpretation tools are best viewed as decision-support systems within established clinical workflows, rather

Powered by Editorial Manager® and ProduXion Manager® from Aries Systems Corporation

than standalone clinical classifiers.
We believe this addition improves conceptual clarity and appropriately moderates claims while preserving the manuscript's forward-looking scope. These revisions strengthen the manuscript's scientific rigor while preserving our assessment of genuine advances in transformer-based variant interpretation. We thank you for helping us achieve this improved balance.

2)While no new analyses are conducted, the description of performance metrics is accurate. Including a short comparative table of representative models with reported AUC/sensitivity values (from cited literature) would provide useful quantitative context.
Author Response:
We thank the reviewer for this helpful suggestion and agree that providing concise quantitative context enhances the manuscript's value for readers seeking to understand performance trends across different methodological paradigms. In response, we have added a new comparative table (Page 13, Table 3; Lines 276-279) to the main text.
Table 3 summarizes representative variant pathogenicity predictors spanning major methodological phases, including rule-based, statistical, machine learning, deep learning, and transformer/protein language model–based approaches. For each representative tool, we report the primary variant class addressed and the performance metrics as originally published (e.g., ROC-AUC, PR-AUC, or accuracy, where available). These examples are intended to illustrate the range of performance values reported across methodological generations, with particular emphasis on the increasing use of standardized, large-scale benchmarking practices in later methodological phases, rather than implying strict performance superiority across tools.
To ensure appropriate interpretation, we explicitly state in the table caption and accompanying text that the reported metrics are derived from heterogeneous evaluation settings and are therefore not directly comparable across tools. Differences in datasets, variant class distributions, labeling standards, and benchmarking protocols substantially limit cross-study comparisons. Accordingly, the table is presented to provide historical and illustrative quantitative context, complementing the more detailed methodological descriptions and tool characteristics provided in Supplementary Table 1.
We believe this addition addresses your concern while maintaining appropriate scientific caution about cross-study comparisons.

Reviewer #2:
This review systematically examines the application of artificial intelligence (AI) in genetic variant interpretation, tracing the evolution from traditional methods to modern machine learning, deep learning, and protein language models. It successfully identifies major challenges in the field, such as data heterogeneity, interpretability, and the interpretation of Variants of Uncertain Significance (VUS). The topic is well-positioned and demonstrates significant scientific and clinical relevance, aligning closely with current trends in precision medicine. The article is well-structured, logically coherent, and features accurately chosen keywords, reflecting a comprehensive grasp of the subject area.
Author Response: We sincerely thank you for the thorough evaluation and positive feedback. We are pleased that you find our systematic examination comprehensive and well-aligned with current trends in precision medicine. We appreciate your recognition of the manuscript's structure, logical coherence, and identification of key challenges in the field. We have carefully addressed all your comments, with detailed point-by-point responses provided below.

To further strengthen the manuscript, the following enhancements are recommended:
1)In the section on Protein Language Models (Phase 5), the authors explicitly cite high performance metrics (e.g., ROC-AUC of 0.905 for ESM-1b). However, similar quantitative data is largely absent for the tools discussed in earlier phases (Phases 1-4). Was this disparity in reporting intended to emphasize the specific performance leap of Transformer-based models, or does it reflect a lack of comparable benchmarks for earlier tools? Clarifying this would help justify why pLMs are presented as a significant advancement.
Author Response: We thank you for this insightful observation. The emphasis on specific metrics for pLMs was indeed intentional, designed to highlight the substantial

Powered by Editorial Manager® and ProduXion Manager® from Aries Systems Corporation

performance gains enabled by Transformer architectures. However, we recognize that the relative lack of metrics for earlier phases could suggest an imbalanced presentation.
The disparity reflects both our initial editorial choice and practical constraints in the literature. Earlier phases (1-4) lack the standardized, large-scale benchmarking protocols that have become common in the deep learning era, making direct cross-phase comparisons scientifically problematic. Our original approach was to emphasize methodological evolution while avoiding potentially misleading numeric comparisons across heterogeneous evaluation frameworks.
In response to your feedback and Reviewer 1's suggestion for quantitative context, we have now addressed this in two complementary ways:
1)Added Table 3 in the main text (Page 13, Line 279) providing representative performance metrics across all methodological phases, with explicit caveats about comparability limitations.
2)Enhanced Supplementary Table 1 by adding a “reported performance” column that summarizes the performance values of all discussed tools. The table legend explicitly notes that metrics are study-specific and cross-tool comparisons should be interpreted cautiously.
Together, these additions provide readers with quantitative context for understanding the field's progression while maintaining appropriate scientific caution about heterogeneous benchmarking practices. We believe this approach better justifies the advancement represented by pLMs within a comprehensive historical framework.

2)The manuscript correctly identifies ancestry bias in reference databases as a critical barrier. To address this, the authors primarily suggest data-centric solutions such as targeted sequencing and federated learning. However, generating new population-scale data is time-consuming. Could the authors discuss if there are algorithmic or computational strategies available in recent literature that can improve the generalizability of current models to underrepresented populations without immediately requiring massive new datasets? Discussing how existing models might be adapted or fine-tuned for diverse ancestries would strengthen the practical utility of this review.
Author Response: We thank the reviewer for this excellent suggestion. We agree that while generating diverse population-scale datasets is essential in the long term, near-term algorithmic and computational strategies are needed to mitigate ancestry bias using existing resources.
In response, we have substantially expanded the discussion in the " Algorithmic Strategies for Polygenic Risk Score Transferability" section (Pages 21-22, Lines 459-475) to systematically highlight computational strategies that enhance model generalizability using existing resources. The revised discussion now organizes these approaches into four complementary categories:
1)Transfer Learning and Domain Adaptation: We discuss transfer learning methods that adjust existing models for population-specific linkage disequilibrium and allele frequency differences using available summary statistics [130, 131] [PMID: 36240765; 36061179]. Domain adaptation techniques based on adversarial learning are also noted as a means to construct ancestry-invariant representations, improving cross-population generalizability [132,133] [Corpus ID: 4357800; PMID: 38160290].
2)Synthetic Data Generation: The revised section briefly describes the use of generative models, such as GAN-based approaches, to augment limited real-world data with synthetic genomes, thereby partially alleviating data scarcity in underrepresented populations [134,135] [PMID: 33539374; Corpus ID: 251136196].
3)Ensemble-based PRS methods: We highlight supervised ensemble frameworks, including CT-SLEB and related multi-ancestry penalized regression approaches, which integrate information across multiple PRS construction strategies to improve predictive performance in diverse populations [136,137] [PMID: 37749244; 36993331].
4)Unsupervised and Zero-shot Approaches: we note emerging unsupervised ensemble methods that aggregate pre-trained PRS models based on prediction concordance, enabling adaptation to target populations without requiring labeled phenotype data [138] [PMID: 39830281].
Although several of these strategies were originally developed in the context of polygenic risk scores, the underlying principles of transfer learning, domain adaptation, and ancestry-invariant representation learning are increasingly being adapted to variant-level pathogenicity prediction models as well.
We believe these additions further strengthen the practical utility of our review by providing readers with actionable computational strategies that can be implemented in

the near term while large-scale data generation efforts progress. We appreciate you highlighting this important gap in our original manuscript.

3)The manuscript focuses heavily on predictors for Mendelian diseases. However, the applicability of these tools to complex, polygenic diseases is not explicitly addressed, given that such conditions are driven by cumulative small-effect variants. Could the authors clarify the limitations of current single-variant predictors for complex traits? Additionally, expanding the 'Future Directions' section to discuss how future AI frameworks might model complex polygenic interactions would significantly strengthen the manuscript's forward-looking perspective.
Author Response: We thank the reviewer for this important observation regarding the applicability of variant predictors to complex, polygenic diseases. We agree that distinguishing between Mendelian and polygenic architectures is crucial for a forward-looking review.
In response to your feedback, we have made two substantial additions to the manuscript:
1)Added a new subsection " Modeling Polygenic and Complex Traits" (Page 17, Lines 364-375) which clarifies the limitations of current single-variant predictors, such as AlphaMissense in capturing the "omnigenic" architecture and non-linear epistatic interactions of polygenic diseases [PMID: 28622505].
2)Expanded the "Future Directions" section with a new subsection " Transitioning to Polygenic Modeling" (Page 22, Lines 476-487) that discusses the shift from single-variant scoring to systems-level polygenic modeling. This addition discusses emerging directions that move beyond isolated variant scoring, including ensemble-based approaches that integrate polygenic background with rare variant burden (e.g., polygenic risk predictions integrating common and rare variants [139]) and graph-based neural network models such as PRS-Net [140], which offer a framework for capturing non-linear gene–gene and pathway-level interactions [141].
[DOI: doi.org/10.1101/2024.11.05.24316779; doi.org/10.1007/978-1-0716-3989-4_35; PMID; 39562137].
These additions clarify the current limitations of single-variant predictors for complex traits while providing a comprehensive forward-looking perspective on how AI frameworks are evolving to address polygenic architecture. We believe this further strengthens the manuscript's scope and practical relevance across different genetic architectures. We appreciate your guidance in addressing this important dimension of variant interpretation.

Reviewer #3:
In this review the authors discussed the evolution from traditional rule-based systems and statistical models to contemporary machine learning, deep learning, and protein language models, while addressing critical challenges in variant classification. The topic is interesting. Some concerns and suggestions are listed as below:
Author Response: We sincerely thank you for the positive feedback and for recognizing the significance of tracing the evolution of variant classification methodologies. We appreciate your interest in the topic and have carefully considered all your concerns and suggestions. We believe the revisions have substantially improved the manuscript, and detailed point-by-point responses are provided below.

1)The authors performed a narrative, method-focused search of PubMed and Google Scholar combining terms such as variant pathogenicity, in silico prediction, deep learning, transformer/foundation model, splicing prediction, non-coding variants, functional assays, and database/tool names.  This exclusion of other databases may introduce selection bias, as significant research may have been published in other languages or databases.
Author Response: We thank the reviewer for this important methodological consideration regarding potential selection bias from our search strategy.
Our search strategy, which focused primarily on PubMed, Google Scholar, and Scopus, was designed to capture the dominant methodological frameworks and tools in computational genomics. The field is characterized by a strong convention of publishing major tools and methodological advances in English-language, internationally indexed journals and repositories to ensure broad adoption, reproducibility, and citation. This convention makes a search of these core databases particularly effective for a review tracing influential approach.
Regarding database coverage, we acknowledge the theoretical concern about missing

important work indexed in other databases. To address this, we performed a validation check of our included tools against Scopus indexing. We found that all tools discussed in our review are indexed in Scopus, with the exception of three very recent preprints (AlphaGenome, Genetic Transformer, MPC) that have not yet undergone peer review. This suggests that our PubMed and Google Scholar search strategy, combined with extensive citation tracking and manual examination of tool repositories, effectively captured the major tools and frameworks in the field without systematic gaps.
We note that attempting a comprehensive keyword-based search across all tools proved impractical due to the heterogeneity of tool names, methodological descriptors, and evolving terminology in this rapidly developing field. Instead, our narrative review approach prioritized identifying influential methodological frameworks and widely adopted tools through iterative literature exploration, forward and backward citation tracking, and consultation of major tool repositories and benchmarking studies.
We have updated the Methods section (Page 4, Lines 75-83) to more explicitly describe our search strategy, including the validation check against Scopus indexing, and to clarify that as a narrative review focused on methodological evolution, our goal is to trace influential approaches that have shaped the field rather than to achieve exhaustive cataloging of all related publications.

2)The authors should introduce what is known and unknown in this field (AI for genomic variant prediction). What about new information in current review? The authors mentioned too many general concepts.
Author Response: We thank the reviewer for this important comment. In response, we have substantially revised the Background to clearly distinguish what is currently well established in the field from what remains unresolved, and to articulate the specific contribution of this review.
The revised introductory paragraphs (Pages 3-4, Lines 54-74) now explicitly contrast empirically supported advances with persistent challenges. We acknowledge that ML and DL approaches have demonstrably improved pathogenicity prediction for many coding variants and that sequence-based models have advanced the interpretation of splicing and regulatory variants. However, we also identify critical gaps that limit clinical translation: a substantial proportion of variants remain classified as VUS; reference datasets exhibit ancestry imbalances that compromise generalizability; deep learning models often operate as "black boxes," complicating alignment with ACMG/AMP guidelines; and experimental validation lags behind computational predictions.
Regarding the contribution of this review, we have clarified that rather than introducing new predictive algorithms or repeating general concepts, we provide a structured, method-focused synthesis that: (1) maps the technological evolution of VIPs across computational paradigms, (2) characterizes the supporting database ecosystem and practical workflows for model development and evaluation, (3) critically examines translational barriers to clinical implementation, and (4) provides actionable recommendations for model selection, ancestry-aware algorithm design, multi-omics integration strategies, and validation frameworks.
The analytical purpose is to clarify where current AI approaches are robust, where caution is warranted, and where further validation is most urgently needed. We believe this revised structure directly addresses the reviewer's concern by moving beyond general concepts to provide a clear framework that distinguishes empirical evidence from unresolved questions while emphasizing the practical, translational focus of our synthesis.

3)The V2P (Variant to Phenotype), is designed to accelerate genetic diagnostics and aid in the discovery of new treatments for complex and rare diseases. This point should be discussed.
Author Response: We thank the reviewer for this valuable suggestion to discuss the Variant-to-Phenotype (V2P) framework and its contributions to accelerating genetic diagnostics and therapeutic discovery.
We fully agree that linking molecular variants to specific clinical manifestations represents a critical evolution in the field. This phenotype-conditioned approach addresses the fundamental challenge of allelic heterogeneity and transforms variant interpretation from binary classification into a clinically actionable framework aligned with patient-specific pathology.
In response, we have added a new subsection entitled “Phenotype-Conditioned Variant Interpretation” (Pages 18-19, Lines 402-409). This section introduces phenotype-conditioned approaches that address allelic heterogeneity by conditioning

pathogenicity predictions on clinical phenotype information. We specifically describe the Variant-to-Phenotype (V2P) framework as an example of a multi-task architecture that integrates protein interactomes, structural descriptors, and evolutionary constraints to generate predictions conditioned on Human Phenotype Ontology (HPO) categories. The revised text highlights how modeling genotype phenotype associations can support rare disease diagnosis and facilitate the identification of phenotype-relevant therapeutic targets, thereby enhancing the clinical interpretability of variant scores. We believe this addition strengthens the review by illustrating how emerging AI frameworks move beyond phenotype-agnostic variant scoring toward more clinically contextualized interpretation.

4)The manuscript should be edited. For example, 'Variants of uncertain significance (VUS)' should be 'VUS' in line 316.
Author Response: We thank you for this observation. We have replaced "Variants of uncertain significance (VUS)" with the abbreviated form 'VUS' in the revised manuscript (Page 16, Line 343) and ensured consistent use of abbreviations throughout the document.

5)How AI gets access to big training genetic data? This point should be discussed in details.
Author Response: We thank the reviewer for highlighting this important aspect of AI model development in genomic variant interpretation. In current practice, AI-based variant pathogenicity predictors are trained on large-scale datasets assembled by researchers from multiple complementary data sources.
As described throughout the manuscript, these training resources typically include population-scale variant databases, curated clinical annotation repositories, protein sequence and structure databases, and large collections of genomic sequences. In particular, recent transformer-based and foundation models leverage extensive unlabeled sequence corpora through self-supervised learning, which enables model pretraining at scale without requiring phenotype-linked or patient-identifiable information.
We address these data sources throughout the manuscript:
1)Section 2 comprehensively reviews the database ecosystem (Pages 4-6, Lines 85-124), including data provenance, curation processes, and access mechanisms.
2)Section 5.2 and 5.3 discusses dataset limitations and biases arising from data collection methods (Pages 16-17, Lines 342-363).
We further clarify that most large-scale training resources used by current AI models are publicly accessible or controlled-access, de-identified datasets governed by database-specific usage and ethical guidelines (e.g., data access committees and data use agreements), which limits direct exposure to individual-level clinical or identifiable genetic data (Page 6, Lines 122-124; Page 17, Lines 360-362).
Together, these discussions provide context for how large-scale genetic data are assembled and utilized for training AI-based variant interpretation models, as well as the practical and conceptual limitations associated with these approaches.

6)The role of clinician in AI-driven variant interpretation should be discussed in details. I wonder if clinicians need to know all computational tools (explainable and user-friendly tools?) in this case.
Author Response: We thank the reviewer for raising this important point regarding the role of clinicians in AI-driven variant interpretation. We agree that AI-based tools should support, rather than replace, clinical expertise and that clinicians are not expected to understand the underlying computational or machine learning architectures.
In response, we have added a new section entitled "Clinical Role in AI-based Variant Interpretation" (Page 18, Lines 386-396). This section clarifies that AI-based variant impact predictors function as decision-support tools within established clinical workflows, with clinicians retaining responsibility for final variant classification. The revised text emphasizes that effective clinical use depends on interpretable outputs, confidence estimates, and traceable evidence aligned with ACMG/AMP guidelines, rather than detailed knowledge of individual computational models.
We also describe how clinicians integrate AI-derived predictions with patient phenotype, family history, segregation analysis, and functional evidence to reach informed classification decisions. We believe this addition directly addresses the reviewer's question and clarifies the clinician's role in AI-assisted variant interpretation.

Powered by Editorial Manager® and ProduXion Manager® from Aries Systems Corporation

| | |
|---|---|
| | 7)Regarding effectively integrating multi-omics information, more texts should be added.<br>Author Response: We thank the reviewer for their valuable suggestion to expand the discussion on effective multi-omics integration. In response, we have added a focused paragraph to the manuscript that strengthens the conceptual discussion of how multi-omics information is incorporated into current variant interpretation approaches (Page 19, Lines 410-422).<br>The revised section emphasizes that effective multi-omics integration is typically selective rather than uniform, with omics layers prioritized according to variant class and disease context. We provide illustrative examples highlighting the relevance of transcriptomic and splicing data for non-coding and splice-site variants, and proteomic or structural information for missense variants affecting protein function.<br>In addition, the paragraph outlines common integration strategies used in AI-based frameworks, including feature-level aggregation, model-level fusion, and post hoc evidence synthesis aligned with ACMG/AMP guidelines. We also briefly acknowledge practical challenges such as data heterogeneity, tissue specificity, and incomplete population representation, and note that prioritization of high-value omics layers and shared databases may facilitate scalable clinical application.<br>We believe this addition enhances the clarity of the discussion on multi-omics integration while remaining consistent with the scope and focus of the review.<br><br>8)How AI founds new genetic variants?<br>Author Response: Thank you for raising this question. We note that the technical detection of variants from sequencing data (variant calling) is typically performed by bioinformatics pipelines rather than AI-based predictors. Our review focuses specifically on AI's role in interpreting and prioritizing variants once they have been identified.<br>To clarify AI's role in interpreting novel variants, we have revised the Data Limitations and Functional Validation Bottlenecks section to explicitly describe how AI-based models support the interpretation and prioritization of rare and novel variants that lack prior annotation or functional evidence (Page 16, Lines 343-347). As now discussed, AI approaches leverage patterns learned from evolutionary conservation, regulatory annotations, and protein structural features to infer potential functional impact and contextualize variants absent from existing databases. This is particularly valuable given that VUS continue to represent a large fraction of clinical findings, especially for rare and non-coding variants.<br>We believe the revised text now clarifies how AI contributes to the interpretation of novel variants within the scope of this review.<br><br>9)Some predictions from AI may turn out to be quite wrong. How did you address this issue?<br>Author Response: Thank you for this important concern regarding erroneous predictions from AI models. We agree that addressing the risk of false positives and false negatives is essential for clinical safety and have discussed this issue from multiple perspectives throughout the manuscript:<br>1)Model positioning: We have revised the Model Interpretability versus Predictive Power section (Pages 17-18, Lines 376-385) to explicitly clarify that AI-based predictors are probabilistic prioritization tools rather than definitive classifiers, requiring cross-validation with orthogonal clinical and experimental evidence. As discussed in the revised text, explainable AI (XAI) frameworks help mitigate the risk of 'false confidence' in erroneous outputs by providing biological rationales, allowing clinicians to distinguish between robust predictions and potential algorithmic artifacts.<br>2)Clinical integration: The Clinical Role in AI-based Variant Interpretation section (Page 18, Lines 386-396) emphasizes that clinicians retain responsibility for final classification decisions and must integrate AI predictions with patient phenotype, family history, segregation data, and functional evidence.<br>3)Validation requirements: Throughout the manuscript, we discuss the need for rigorous benchmarking, external validation, and functional validation to identify model limitations and reduce the risk of incorrect predictions affecting clinical decisions.<br>We believe these multi-layered safeguards positioning AI as decision support rather than replacement, requiring explainability, maintaining clinical oversight, and enforcing validation standards, collectively address the concern about erroneous predictions. |
| **Additional Information:** | |

*Powered by Editorial Manager® and ProduXion Manager® from Aries Systems Corporation*

| Question | Response |
|---|---|
| Are you submitting this manuscript to a special series or article collection? | No |
| **Experimental design and statistics**<br><br>Full details of the experimental design and statistical methods used should be given in the Methods section, as detailed in our Minimum Standards Reporting Checklist. Information essential to interpreting the data presented should be made available in the figure legends.<br><br>Have you included all the information requested in your manuscript? | Yes |
| **Resources**<br><br>A description of all resources used, including antibodies, cell lines, animals and software tools, with enough information to allow them to be uniquely identified, should be included in the Methods section. Authors are strongly encouraged to cite Research Resource Identifiers (RRIDs) for antibodies, model organisms and tools, where possible.<br><br>Have you included the information requested as detailed in our Minimum Standards Reporting Checklist? | Yes |
| **Availability of data and materials**<br><br>All datasets and code on which the conclusions of the paper rely must be either included in your submission or deposited in publicly available repositories (where available and ethically appropriate), referencing such data using a unique identifier in the references and in the "Availability of Data and Materials" section of your manuscript. | No |

| | |
|---|---|
| Have you have met the above requirement as detailed in our Minimum Standards Reporting Checklist? | |
| If not, please give reasons for any omissions below.<br><br>as follow-up to "**Availability of data and materials**<br><br>All datasets and code on which the conclusions of the paper rely must be either included in your submission or deposited in publicly available repositories (where available and ethically appropriate), referencing such data using a unique identifier in the references and in the "Availability of Data and Materials" section of your manuscript.<br><br>Have you have met the above requirement as detailed in our Minimum Standards Reporting Checklist?<br><br>" | This is a review article that synthesizes and discusses findings from previously published studies rather than generating or analyzing new datasets or code. Therefore, no new data or code were produced in this work. All referenced datasets and tools are publicly available in the cited original publications. |
| GigaScience has policies and guidelines in place for the use of generative AI-writing tools such as ChatGPT. If you have used such writing tools to assist with writing the manuscript this must be declared and cited in the text. Authors should not list AI-writing tools and other AI-assisted technologies as an author or co-author and should acknowledge that they are fully responsible for text generated or refined by AI-writing tools.<p><br><br>A summary of use (particularly in the introduction or among methods) needs to be included at the end of the paper, and the outputs should also be included as a supplementary file hosted in GigaDB or other open repositories. Please <a href=https://academic.oup.com/gigascience/pages/editorial_policies_and_reporting_standards target="_new" > read our guidelines for more information. </a> <p><br><br>By submitting to GigaScience, you are aware of the journal's AI-writing tools policy, and if you have declared use of such tools below, you have acknowledged this where appropriate in your manuscript | No |

| | |
|---|---|
| and have made a summary of use and outputs available. </b><p> <b>AI-assisted writing tools have been used in the preparation of this manuscript? | |

Manuscript

Click here to access/download;Manuscript;main_manuscript_0107.docx

# Harnessing Artificial Intelligence for Genomic Variant Prediction: Advances, Challenges, and Future Directions

Indah Pakpahan [1,2,3#], Mentari Sihombing [1,2,4#], Haohan Liu [2], Mengyao Wang [2], Zheng Su [5], Mingyan Fang [6]

[1] *Dalian University of Technology, Dalian 116000, China*

[2] *BGI Research, Wuhan 430074, China*

[3] *Department of Bioprocess Engineering, Faculty of Biotechnology, Institut Teknologi Del, Laguboti, North Sumatera, Indonesia*

[4] *Department of Software Engineering, Faculty of Vocational Studies, Institut Teknologi Del, Laguboti, North Sumatera, Indonesia*

[5] *School of Biotechnology and Biomolecular Sciences, Faculty of Science, The University of New South Wales, Sydney, NSW 2052, Australia.*

[6] *State Key Laboratory of Genome and Multi-omics Technologies, BGI Research, Shenzhen 518083, China*

*# These authors contributed equally to this work.*

***Correspondence*:**

Mingyan Fang, fangmingyan@aliyun.com Zheng Su, suzheng@whu.edu.cn

## Abstract

Accurate genetic variant interpretation is crucial for disease research and the development of targeted therapies. Artificial intelligence (AI) is transforming this field by integrating computational methodologies across structural biology, evolutionary analysis, and multimodal genomic data. This review examines the evolution from traditional rule-based systems and statistical models to contemporary machine learning, deep learning, and protein language models, while addressing critical challenges in variant classification. Key obstacles include data heterogeneity, interpretability, and the persistence of Variants of Uncertain Significance (VUS), emphasizing the critical need for explainable AI frameworks and more inclusive genomic databases to improve predictive accuracy across diverse populations. Based on the assessment of current Variant Impact Predictors (VIPs), we propose strategies for enhanced predictor selection, effective multi-omics data integration, and optimized computational workflows. These recommendations aim to enhance variant interpretation accuracy in both research settings and clinical practice, ultimately contributing to advances in personalized medicine.



**Keywords**: Variant Impact Predictors (VIPs), Artificial Intelligence (AI), variant databases, multi-omics integration, Variants of Uncertain Significance (VUS)

## Background

High-throughput sequencing and the Human Genome Project have enabled comprehensive catalogs of human genetic variation, yet distinguishing pathogenic from benign variants remains a central bottleneck in research and clinical genetics [1-3]. Over the last decade, Variant Impact Predictors (VIPs) have progressed from rule-based heuristics to statistical models, machine learning (ML), deep learning (DL), and, most recently, transformer-based large language models that integrate evolutionary, structural, and multi-omics signals [4, 5].

Despite rapid methodological advances, the field faces a paradox: while computational methods have improved significantly, their clinical utility remains limited. ML and DL approaches have demonstrably improved pathogenicity prediction for coding variants, and sequence-based models have advanced interpretation of splicing and regulatory variants. However, several critical challenges persist. First, a substantial proportion of variants remain classified as Variants of Uncertain Significance (VUS), reducing their diagnostic impact. Second, reference datasets such as Genome Aggregation Database (gnomAD) [6] and ClinVar [7] exhibit ancestry imbalances that affect generalizability across populations. Third, DL and transformer-based models often operate as “black boxes,” complicating their alignment with American College of Medical Genetics and Genomics (ACMG)/ Association for Molecular Pathology (AMP) interpretive guidelines. Finally, experimental validation lags behind computational predictions, creating a gap between algorithmic output and clinical translation.

This review addresses these challenges through a comprehensive, method-focused synthesis. We map the technological evolution of VIPs across computational paradigms, characterize the supporting database ecosystem, and outline practical workflows for data preprocessing, model development, and evaluation. Rather than proposing new algorithms, we assess current artificial intelligence (AI) capabilities: identifying where approaches are robust, where caution

is warranted, and what translational gaps must be addressed for clinical implementation. We provide actionable recommendations for model selection, ancestry-aware algorithm design, multi-omics integration, and validation frameworks to improve the reliability, transparency, and equity of variant interpretation in diverse populations.

Methodologically, this review adopts a narrative, method-focused search of PubMed, Google Scholar, and Scopus combining terms such as variant pathogenicity, *in silico* prediction, deep learning, transformer/foundation model, splicing prediction, non-coding variants, functional assays, and database/tool names. This primary search was supplemented by manual cross-referencing of seminal reviews and benchmarking repositories to ensure a comprehensive coverage of diverse computational frameworks. We prioritized peer-reviewed method papers with clear training/validation descriptions, widely used tools across coding and non-coding tasks, comparative evaluations, and resources on functional screening, fairness and explainable AI (XAI).

## Curated Database Infrastructure Supporting Variant Interpretation

The accurate interpretation of genetic variants relies on a stratified data ecosystem integrating genomic, clinical, and functional evidence, in which each layer offers a distinct analytical perspective while remaining interconnected to enable comprehensive pathogenicity assessment.

Population-scale genomic initiatives provide baseline frequency profiling, establishing context for variant rarity assessment. Resources including the Single Nucleotide Polymorphism Database (dbSNP) [8], the 1000 Genomes Project (1KGP) [9], gnomAD [6], and UK Biobank [10] provide allele frequency distributions and mutational constraint profiles across diverse ancestries, enabling distinction between rare pathogenic variants and common benign polymorphisms, especially in underrepresented populations [11]. Functional Annotation of

Variants - Online Resource (FAVOR) [12] complements these resources by integrating functional annotations to assess variants lacking clinical evidence.

Pathogenicity evidence emerges from curated clinical and disease-specific repositories. ClinVar [7], Human Gene Mutation Database (HGMD) [13], Human Variants Database (HuVarBase) [14], and ClinGen [15] aggregate experimentally or clinically validated genotype-phenotype associations, forming an empirical foundation for supervised learning approaches in variant classification.

Standardized disease ontologies help transform clinical observations into computational frameworks. Online Mendelian Inheritance in Man (OMIM) [16], Orphanet [17], and Human Phenotype Ontology (HPO) [18] connect genetic alterations to specific disease mechanisms and biological pathways. Additionally, Gene Ontology (GO) [19] complements phenotype-focused resources by clarifying functional impacts at molecular and cellular levels.

Domain-specific repositories provide further refinement of variant interpretation in specialized contexts. Oncology-focused databases, such as Catalogue of Somatic Mutations in Cancer (COSMIC) [20], Database of Curated Mutations (DoCM) [21], and ONGene [22] aggregate tumor-specific mutations. Gene-centric functional assays (e.g., a curated *BRCA1* functional dataset [23]), provide confidence labels for many clinically relevant genes.

Beyond sequence and phenotype resources, protein architecture databases including Universal Protein Resource (UniProt) [24] and Protein Data Bank (PDB) [25] provide the three-dimensional framework essential for understanding variant consequences at the molecular level, particularly useful for structure-function relationship modeling.

Finally, specialized benchmarking and validation resources, including the Benchmark Database for Variations (VariBench) [26], VariSNP [27], and VarCards2 [28] support model

evaluation and comparison of emerging AI predictors by providing standardized test sets and performance metrics.

Across this data ecosystem (**Table 1**) spanning evolutionary, biochemical, structural, and regulatory domains lies the foundation for advanced AI architectures that can effectively model the complex relationships underlying variant pathogenicity [29]. Access to these resources is governed by institutional guidelines and data use agreements ensuring appropriate use of de-identified data.

## The Technological Trajectory of Variant Pathogenicity Assessment

The development of VIPs has evolved through five distinct yet overlapping paradigms, each addressing limitations of previous approaches while expanding analytical capabilities (**Figure 1**). This progression mirrors broader technological trends within computational biology, and has evolved from initial rule-based heuristics toward advanced transformer architectures. Each step has facilitated progressively refined insights into genomic variation (**Supplementary Table 1**).

### Phase 1: Rule-Based Biological Heuristics

Early predictive tools emerged from foundational biological insights. Pioneering rule-based predictors such as Sorting Intolerant From Tolerant (SIFT) [30] and Polymorphism Phenotyping (PolyPhen) [31] relied on empirical knowledge and evolutionary principles to evaluate variant pathogenicity. These tools primarily relied on sequence conservation and amino acid physicochemical properties to distinguish between benign and deleterious variants. Gene-Aware Variant INterpretation (GAVIN) [32] applied predefined classification logic to refine pathogenicity predictions within gene-specific contexts. Their strength lay in their high interpretability, as the underlying biological rules were explicit and transparent.

However, this rule-based approach inherently limited their scalability and ability to capture the complex, non-linear patterns emerging from rapidly expanding genomic datasets. Though computationally efficient, these methods typically operated within limited genomic contexts and largely overlooked non-coding or regulatory regions such as promoters or splice sites [33]. Despite these constraints, early-phase predictors remain valuable for preliminary assessments and continue to be incorporated into comprehensive prediction frameworks [34], particularly where interpretability is prioritized over prediction complexity.

**Phase 2: Statistical Modeling and Probabilistic Frameworks**

As genomic databases expanded in both size and diversity, statistical methods emerged to enhance the prediction accuracy through probabilistic modeling. Tools such as MutationAssessor [35] utilized evolutionary conservation patterns within protein families, while Functional Analysis Through Hidden Markov Models (FATHMM) [36] integrated evolutionary conservation scores into sequence-based probabilistic models to estimate variant pathogenicity. Eigen [37], an unsupervised spectral method, prioritized variants by analyzing annotation correlations and constructing a weighted score across both coding and non-coding genomic regions. In parallel, Genomic Evolutionary Rate Profiling++ (GERP++) [38] quantifies evolutionary constraint using a maximum likelihood model to calculate rejected substitutions, and is widely used as an annotation feature in downstream predictive frameworks.

While these tools have advanced the ability to contextualize genomic variation, predictive methods still face substantial challenges when analyzing rare or novel variants due to insufficient representation in existing reference datasets. Additionally, their heavy reliance on high-quality reference annotations limited their effectiveness in classifying clinically important yet poorly characterized VUS [39], prompting further developments toward data-driven ML methods.

### Phase 3: Traditional Machine Learning and Ensemble Approaches

ML algorithms transformed variant prediction by capturing complex, non-linear patterns in multidimensional genomic datasets, enabling integration of diverse biological features [40]. Approaches in this era can be broadly categorized into:

**Classical Machine Learning Classifiers:** Naive Bayes classifiers were applied in tools like PolyPhen-2 [41], which integrates sequence and structure-based features to predict the effects of amino acid substitutions. This algorithm was also effectively deployed in disease-specific contexts, such as Polymorphism Phenotyping for Hypertrophic Cardiomyopathy (PolyPhen-HCM) [42], CanPredict [43], and Splicing-based Analysis of Variants (SPANR) [44]. Support Vector Machines (SVMs) and Random Forests (RFs) underpin tools such as Combined Annotation Dependent Depletion (CADD) [33], Predictor of human Deleterious Single Nucleotide Polymorphisms (PhD-SNP) [45], Variant Effect Scoring Tool (VEST) [46], Meta-analytic Support Vector Machine (MetaSVM) [47], MutPred [48], incorporating diverse features such as evolutionary conservation, protein structure, and gene-level annotations within unified predictive frameworks SQUIRLS [49] focuses on splice-altering variants, while genome-wide annotation of variants (GWAVA) [50] extends this approach to non-coding regions, prioritizing functional regulatory variants across diverse biological contexts.

The RF algorithm was similarly adapted for specialized tasks, powering tools like the Variant Impact Predictor for PIDs (VIPPID) [51] for immunodeficiencies and Prediction of Deleterious Missense Mutation for IRDs (PdmIRD) [52] for retinal diseases. For instance, SVMs identify optimal hyperplanes to separate classes, while RF build multiple decision trees and aggregate their results, offering robustness and handling high-dimensional data effectively.

**Gradient Boosting Approaches:** More recent implementations employ gradient boosting machines (GBMs) to enhance classification performance. Multimodal Annotation Generated

Pathogenic Impact Evaluator (MAGPIE) [5], Consequence-Agnostic Pathogenicity Interpretation of Clinical Exome variations (CAPICE) [53], INDELpred [54], Mendelian Clinically Applicable Pathogenicity (M-CAP) [55], and PON-P3 [56] exemplify this approach by integrating multi-source annotations via gradient-boosted decision trees. The strength of GBMs for leveraging complex feature sets also made them ideal for building specialized predictors like CardioBoost [57] for cardiac genetics. GBMs operate on the principle of iterative improvement: they sequentially build models, with each new model attempting to correct the errors of its predecessor.

While these models demonstrate superior accuracy, they require high-quality, well-curated training datasets; when data are limited, sparse or imbalanced, challenges such as overfitting and limited generalizability can compromise their clinical utility [58].

**Ensemble Prediction Systems:** To address the inherent limitations of individual algorithms, ensemble methodologies aggregate outputs from multiple predictors, thereby enhancing robustness and reliability [59]. Tools such as the Rare Exome Variant Ensemble Learner (REVEL) [60] and Parallel SMote Undersampled Random Forest (parSMURF) [61] exemplify this strategy by combining complementary models to mitigate individual weaknesses while amplifying collective strengths. Although ensemble methods introduce additional computational costs and diminish transparency, these frameworks have become integral to clinical genetics pipelines where reliability is paramount. Despite improved accuracy, ML-based methods remain sensitive to training data and often lack interpretability, limiting their use as standalone clinical tools.

**Phase 4: Deep Learning Approaches**

DL has further advanced variant interpretation by exploiting neural networks to model nonlinear relationships in large, multidimensional genomic datasets [62]. DL models can learn

hierarchical feature representations directly from raw data, reducing the reliance on manual feature engineering. Notable examples include deleterious annotation of genetic variants using neural networks (DANN) [63] used a deep neural network trained on the same functional annotations as CADD to score both coding and non-coding variants and PrimateAI [64], which leverages evolutionary signatures across primates to enhance prediction accuracy. This capacity for learning from raw sequence data has improved the prediction of non-coding and regulatory variants. Models like DeepSEA [65] and Basenji [66] demonstrated that chromatin accessibility and gene expression could be predicted directly from DNA sequence, capturing both proximal and distal effects. PromoterAI [67] focused on predicting the impact of promoter variants, further refining deep learning–based modeling of regulatory sequences.

In splicing prediction, DL brought a significant leap in accuracy. SpliceAI [68] utilized convolutional neural networks to capture long-range dependencies in pre-mRNA sequences, often outperforming previous models, though at the cost of interpretability. MMSplice [69] attempted to address this interpretability gap through more modular or explainable designs. While these approaches demonstrate substantial sensitivity, challenges persist regarding their "black box" nature and intensive computational requirements [70].

**Phase 5: Transformer Architectures**

The most recent technological leap has been catalyzed by adapting transformer architectures and language modeling principles to genomic and protein sequences. Originally developed for natural language processing, transformer models have become powerful tools for predicting functional impacts of genetic variants [71]. The core innovation lies in the self-attention mechanism, which allows the model to weigh the importance of different parts of the input sequence when processing each element [72]. This enables the capture of long-range dependencies and global context within biological sequences, a key aspect for understanding

how mutations in one part of a protein can affect distant functional regions [73]. The application of transformers has evolved into two powerful, complementary paradigms;

**Protein Language Models (pLMs):** Pre-trained on vast corpora of evolutionary sequences, pLMs learn fundamental principles of protein structure and function. Models like ESM-1b [74] excel at capturing subtle sequence constraints, distinguishing isoform-specific pathogenic variants with high accuracy (ROC-AUC: 0.905 on ClinVar, 0.897 on HGMD/gnomAD). This approach is exemplified by tools like Variant impact Predictor (VariPred) [75], which leverages these learned representations to predict variant effects, often outperforming traditional structure-dependent methods. The strength of pLMs is further demonstrated by their versatility in tasks such as masked residue prediction (82% accuracy) [76] and sequence conservation analysis (MCC = 0.596) [77].

**Regulatory Genome Transformers:** These models are designed to interpret the non-coding genome by learning the regulatory code directly from DNA sequence. Enformer [78] set a new standard by using a transformer architecture to achieve leading performance in predicting chromatin accessibility and gene expression profiles from sequence context, capturing effects of distal enhancers. This paradigm is extended by tools like AlphaGenome [79], which integrates over 100 regulatory features in a multitask framework. EpiGePT [80] focuses on precise prediction of context-specific epigenomic signals, achieving high performance (Pearson r = 0.710; auROC = 0.949).

The potential of this era is revealed in models that fuse these approaches or leverage their insights for specific clinical tasks. AlphaMissense [71] by DeepMind represents a seminal work, fusing structural insights from AlphaFold [81] with the pattern recognition of language models to generate a massive, highly accurate map of missense variant pathogenicity. Similarly, MutFormer [82] integrates self-attention with convolutional layers for missense analysis, and Genetic Transformer (GeneT) [83] achieves high recall rates (99% in synthetic data, 97.85%

in clinical cohorts) for identifying causative variants. This fusion also enables more specialized applications, such as SpTransformer [84], which incorporates tissue specificity into splicing prediction.

Together, these findings suggest that transformer architectures can achieve improved performance across diverse variant interpretation tasks, including coding, non-coding, and splicing variants. In benchmark comparisons, these models often outperform earlier DL approaches, though performance gains vary considerably with task definition, training data, and evaluation frameworks. These advances reflect a transition from feature-engineered methods to models that learn context-aware representations directly from biological sequences, potentially improving predictive capacity while introducing challenges in scalability and interpretability. Representative algorithms and VIPs across these technological phases are summarized in **Table 2**, with detailed tool characteristics in **Supplementary Table 1**.

**Table 2**. Computational Algorithms used for variant impact predictors

| No. | Algorithm | Description | VIPs | References |
|---|---|---|---|---|
| 1 | Naive Bayes | A probabilistic graphical model that represents a set of variables and their conditional dependencies. | PolyPhen-2, PolyPhen-HCM, CanPredict, SPANR | [41-44] |
| 2 | Support Vector Machines | A supervised learning model that analyzes data for classification and regression analysis. | CADD, PhD-SNP, MetaSVM | [33, 45, 47] |
| 3 | Random Forest | An ensemble learning method that operates by constructing multiple decision trees. | VEST, MutPred, SQUIRLS, GWAVA, VIPPID, PdmIRD, REVEL, parSMURF | [46, 48-52, 60, 61] |
| 4 | Gradient Boosting Machines | A ML technique for regression and classification problems that builds a model in a stage-wise fashion. | MAGPIE, CAPICE, INDELpred, M-CAP, PON-P3, CardioBoost | [5, 53-57] |
| 5 | Neural Networks | A set of algorithms modeled after the human brain, designed to recognize patterns. | DANN, PrimateAI, DeepSEA, Basenji, PromoterAI, SpliceAI, MMSplice, AIVAR | [63-69, 85] |
| 6 | Transformer | A DL model that uses self-attention mechanisms to process sequential data, capturing long-range dependencies and relationships in the data. | AlphaMissense, VariPred, Enformer, AlphaGenome, EpiGePT, MutFormer, GeneT, SpTransformer | [71, 75, 78-80, 82-84] |

To provide illustrative quantitative context, **Table 3** presents performance metrics for representative VIPs spanning major methodological phases, as reported in original publications. These values derive from heterogeneous evaluation settings and are not directly comparable.

**Table 3.** Representative variant impact predictors and their reported performance

| No. | Methodological paradigm | VIPs | Variant type(s) | Reported performance (as published) | Reference |
|---|---|---|---|---|---|
| 1 | Rule-based | SIFT | Missense (amino acid–altering) variants | ROC-AUC: 0.80 - 0.82 | [30] |
| | | PolyPhen | Missense (amino acid–altering) variants | ROC-AUC: ≈ 0.83 | [31] |
| 2 | Statistical | MutationAssessor | Missense (amino acid–altering) variants | ROC-AUC: ≈ 0.86 | [35] |
| | | FATHMM | Coding and non-coding variants | Accuracy: ≈ 0.86 | [36] |
| 3 | Machine Learning | CADD | Coding and non-coding variants | ROC-AUC: 0.90 - 0.93 | [33] |
| | | REVEL | Missense (amino acid–altering) variants | ROC-AUC: 0.90 - 0.91 | [60] |
| 4 | Deep Learning | SpliceAI | Splicing-altering variants | PR-AUC: ≈ 0.90 | [68] |
| | | DeepSEA | Non-coding regulatory variants | ROC-AUC: ≈ 0.96 | [65] |
| 5 | Transformer/ pLM | AlphaMissense | Missense (amino acid–altering) variants | ROC-AUC: ≈ 0.94 | [71] |
| | | MutFormer | Missense (amino acid–altering) variants | ROC-AUC: 0.92 - 0.97 | [82] |

***Note:*** *Variant types refer to the primary design scope of each method. Representative VIPs from each methodological paradigm are shown to illustrate the range of performance metrics reported in the literature. Metrics are taken from the original publications and are not directly comparable due to differences in datasets, variant classes, and evaluation protocols.*

## Integrative Tools for Variant Annotation

Comprehensive annotation pipelines consolidate outputs from diverse databases and predictive algorithms to streamline variant interpretation. Ensembl Variant Effect Predictor (VEP) [86] annotates variants with gene-level and regulatory features, supporting flexible plug-in integration of tools such as SIFT, CADD, and SpliceAI. ANNOVAR [87] enables gene-based,

region-based, and filter-based annotation in unified workflows, incorporating population frequency data (*e.g.,* gnomAD) and multiple pathogenicity scores.

Web-based platforms further enhance usability and evidence integration. VarSome [88] and MobiDetails [89] aggregate clinical annotations, *in silico* predictions, and allele frequencies via interactive interfaces, apply ACMG-based classification framework. InterVar [90], emphasizes rule-based implementation of ACMG guidelines, offering reproducible, guideline-concordant classification support. Exomiser [91] combines variant pathogenicity scores with phenotype data (HPO terms) to prioritize candidate variants, especially in rare disease diagnosis.

These integrative tools reduce the burden of manual curation by consolidating diverse resources into unified workflows, enabling efficient filtering and evidence synthesis for both research and clinical applications. However, while these pipelines effectively consolidate evidence from multiple sources, the complexity of variant interpretation requires a systematic approach to optimize their implementation. The selection of appropriate predictors and the integration of their outputs into coherent clinical decisions necessitates a structured workflow framework.

## Variant Interpretation Workflow and Evaluation Considerations

To address these challenges systematically, the variant interpretation process can be conceptualized as a structured pipeline that links raw genomic data to actionable clinical predictions (**Figure 2**). This systematic approach provides a comprehensive framework for improved variant classification accuracy and supports the development of more equitable genomic medicine applications.

The framework encompasses three interconnected phases that collectively transform raw genomic data into clinically actionable insights. The initial phase centers on comprehensive data acquisition, integrating population-level variant frequencies, disease-specific variants repositories, high-throughput molecular characterization profiles, and curated clinical annotations. This foundation transitions into an advanced preprocessing phase that systematically integrating heterogeneous data sources, including evolutionary conservation, protein structural, and regulatory element characterizations. Subsequent steps include normalization, feature extraction, and dimensionality reduction to optimize the computational feature space for downstream analytical applications [92].

The analytical phase leverages sophisticated predictive modeling approaches, employing rigorous hyperparameter optimization strategies to achieve optimal discriminative performance [93]. This computational framework generates probabilistic assessments that require systematic validation through robust benchmarking protocols. Evaluation encompasses multiple complementary metrics: sensitivity and specificity for detection of pathogenic versus benign variants, precision metrics that characterize predictive accuracy, and the area under the receiver operating characteristic (ROC) curve (AUC), which provides threshold-independent assessment of discriminative performance [94]. Comparative analyses against benchmark datasets, such as ClinVar or consortium-established validation cohorts, enable cross-method evaluations while ensuring generalizability through cross-validation [95].

The optimization of these computational models requires adherence to stringent development protocols that emphasize data integrity through integration of high-quality variant annotations from diverse repositories such as gnomAD and ClinVar. Advanced feature engineering requires integration of multi-omics data layers spanning genetic, proteomic, regulatory, and clinical domains [96]. Robust model development paradigms demand thorough cross-validation,

systematic hyperparameter tuning, and validation against independent external datasets to ensure reproducibility and clinical applicability [97].

## Challenges Limiting Clinical Translation

Despite these methodological advances across both general and specialized AI approaches, several interconnected challenges persist in limiting the clinical deployment of VIPs. These challenges span data limitations, dataset biases, polygenic and complex traits, and model interpretability, each of which would benefit from targeted solutions to bridge the gap between computational prediction and clinical application.

### Data Limitations and Functional Validation Bottlenecks

VUS continue to represent a large fraction of clinical findings, particularly for rare and novel or non-coding variants [98]. AI-based models address this challenge by leveraging evolutionary conservation, regulatory annotations, and protein structural features to infer potential pathogenicity and prioritize variants lacking database evidence or functional validation, although significant VUS challenges remain. While computational methods such as matrix factorization [99] and active learning [100] provide incremental gains, they often remain dependent on sparse or biased training data.

Recent years have seen growing reliance on high-throughput functional assays such as CRISPR-Cas9 screens and multiplexed assays of variant effects (MAVEs), which can directly measure variant function at scale [101] . These approaches are beginning to be incorporated into clinical frameworks, for example MAVE-derived scores have been incorporated into ClinGen rules for *BRCA1*, *TP53*, and *PTEN* [102]. Yet, widespread clinical use remains limited by assay cost, turnaround time, and availability outside specialized centers.

### Dataset Biases and Generalizability Issues

Beyond general data limitations, demographic imbalances in genomic databases primarily skewed toward individuals of European ancestry constrain model performance across diverse populations [103]. Such sampling biases may reduce the reliability of variant interpretation in underrepresented groups [104]. Training datasets for AI models are typically de-identified and accessed through controlled mechanisms governed by data use agreements and ethical guidelines. While targeted sequencing and federated learning are emerging, coverage remains incomplete for many populations, limiting equitable variant interpretation [105].

**Modeling Polygenic and Complex Traits**

Current single-variant VIPs like AlphaMissense are optimized for high-penetrance Mendelian variants, which differs substantially from diverges from the genetic architecture of complex, polygenic diseases. While these models excel at identifying variants disrupting protein stability or evolutionary conservation, they struggle to capture the cumulative contribution of numerous small-effect variants underlying conditions such as hypertension, hyperlipidemia, and type 2 diabetes [106].

Furthermore, current predictors operate largely in isolation, neglecting the "omnigenic" reality in which disease susceptibility is influenced by epistatic interactions and regulatory networks rather than single-locus disruptions. Consequently, binary classification of variants as "pathogenic" or "benign" lacks the granularity required for complex trait prediction, where risk is continuous and context-dependent [107].

**Model Interpretability versus Predictive Power**

Even when data issues are mitigated, the interpretability of advanced AI models remains an important barrier to clinical adoption. ML models like evolutionary model of variant effect (EVE) [108] have demonstrated high performance in variant prediction but are often too complex to explain and their opacity poses challenges for clinical trust, regulatory approval,

and usability [109, 110]. Recent efforts in XAI strategies [111] and interpretable classifiers like Artificial Intelligent Variant Classifier (AIVAR) [85] seek to bridge this gap by providing transparent and biologically meaningful rationales for prediction. XAI frameworks help distinguish robust predictions from algorithmic artifacts, but AI outputs are best interpreted as probabilistic prioritization requiring validation with clinical and experimental evidence.

### Clinical Role in AI-based Variant Interpretation

AI-based VIPs are designed to support clinical decision-making within established diagnostic workflows rather than operate as standalone classifiers. Empirical evaluations show VIPs function most effectively when augmenting expert review, with clinicians retaining final classification decisions, particularly for VUS [112, 113].

In practice, clinicians integrate AI predictions with phenotypic information, family history, segregation validation, and functional evidence to guide variant classification. This workflow underscores that successful clinical adoption is strongly influenced by how AI outputs are presented: interpretable scores, confidence estimates, and evidence aligned with ACMG/AMP guidelines are essential. Ultimately, clinical utility depends as much on usability, explainability, and seamless workflow integration as on algorithmic performance.

## Toward More Interpretable and Clinically Actionable Predictions

Emerging trends ranging from multi-omics fusion to foundation models have the potential to advance variant interpretation along five strategic axes. These axes collectively span molecular, computational, functional, and population-level dimensions of clinical genomics.

### Phenotype-Conditioned Variant Interpretation

Phenotype-conditioned approaches link genetic alterations directly to clinical manifestations, addressing allelic heterogeneity. Multi-task architectures such as the Variant-to-Phenotype framework [114] integrate protein interactomes, structural descriptors, and evolutionary constraints to predict pathogenicity conditioned on HPO categories. By capturing genotype-phenotype associations, these approaches accelerate rare disease diagnosis and identify therapeutic targets, enhancing variant scoring into a clinically useful tool that can inform patient-specific pathology.

**Multi-Omics Evidence Integration**

Integrating genomic, transcriptomic, proteomic, and epigenetic data [115] improves statistical power for associations with low-frequency variants [116]. Effective integration typically involves selectively, prioritizing omics layers most informative for specific variant types [117, 118]. Transcriptomic and splicing data are critical for interpreting non-coding and splice-site variants, particularly for resolving VUS [119, 120], whereas proteomic and structural information is more informative for missense variants [121]. Tissue-specific expression needs to be considered, as variant effects can be context-dependent.

AI-based approaches integrate multi-omics through feature-level aggregation, model-level fusion, or post hoc synthesis according to ACMG/AMP guidelines. Despite their potential, these approaches remain constrained by data heterogeneity, tissue specificity, and incomplete population coverage. Selective prioritization of high-value omics layers and shared databases offer a promising strategy for scalable clinical adoption.

**Foundation Models & Few-Shot Adaptation**

Transformer-based language models enable fine-tuning with minimal labeled data, addressing challenges posed by rare variants. Pre-trained on large, unlabeled datasets, these models can be efficiently adapted to novel variant types using parameter-efficient fine-tuning [122] and

prompt engineering [123], potentially making high-performance variant prediction accessible for understudied conditions. Recent benchmarking of DNA foundation models demonstrates their capacity for zero-shot variant effect prediction, with large, multi-species architectures demonstrating strong discriminative power in capturing both local and extended contextual effects [124].

These transformer-based approaches can be conceptually unified through a shared pipeline that transforms biological sequences into clinical predictions (**Figure 3**). Biological sequences undergo tokenization and embedding generation, converting raw genomic data into computational representations. Contextual encoding via multi-head self-attention processes these embeddings, where unsupervised pre-training objectives, including masked language modeling, permutation language modeling, and contrastive learning [125], enable the models to capture meaningful sequence patterns without labeled data. Task-specific adaptation occurs through specialized prediction heads (task-specific output layers) coupled with either frozen or fine-tuned backbones. Prompt-engineering strategies enable efficient knowledge transfer when limited labeled datasets are limited, and predictions can be further interpreted or calibrated using functional validation and systems-level analyses to support clinical interpretation.

**Scalable Functional Validation & Systems-Level Context**

Scaling functional validation beyond specialized centers is increasingly feasible through collaborative resources. Resources such as the Atlas of Variant Effects [126] and MaveDB [127] provide standardized repositories of multiplexed assay data directly incorporated into ACMG/AMP classification frameworks. At the same time, integration of functional readouts with systems biology analyses including pathway and interaction networks, may help contextualize variant effects at multiple biological levels [128]. Functional evidence is being more systematically integrated into precision medicine workflows, rather than an ad hoc

supplement, and pairing these insights with diverse population data supports robust cumulative-risk interpretation while accounting for population-specific variation.

## Equity-Aware Modeling Across Variant and Polygenic Risk

Systematically diversifying genomic databases addresses biases from population underrepresentation [103]. Collaborative initiatives like the Human Heredity and Health in Africa consortium [129] provide models for ethical, scientifically robust data collection among underserved populations. Ethical AI practices, such as fairness assessments, transparent model documentation, and inclusive stakeholder engagement help mitigate algorithmic biases.

## Algorithmic Strategies for Polygenic Risk Score Transferability

Equity challenges become particularly pronounced when extending variant-level prediction frameworks to polygenic risk modeling. While generating diverse population-scale data is essential for addressing these challenges, several computational strategies address ancestry bias using existing resources. Transfer learning adjusts models for population-specific linkage disequilibrium and variants shift using existing summary statistics [130, 131], while domain adaptation constructs ancestry-invariant representations through adversarial learning [132, 133]. Data augmentation via generative models supplements scarce data with synthetic genomes [134, 135].

For polygenic risk prediction, ensemble methods integrate multiple polygenic risk score construction strategies: supervised approaches like CT-SLEB [136] and multi-ancestry polygenic risk scores based on ensemble of penalized regression models [137] combine clumping-thresholding, empirical Bayes, and penalized regression using target population genome-wide association study data, while unsupervised frameworks such as Unsupervised Ensembles [138] aggregate pre-trained models based on prediction concordance without

requiring phenotype data from target populations, which enables robust performance even in underrepresented populations by circumventing sparse phenotype availability.

### Transitioning to Polygenic Modeling

For complex diseases, single-variant predictions may have limited utility. Emerging frameworks are moving toward systems-level polygenic modeling that integrates cumulative genetic architecture. Ensemble methods like polygenic risk predictions integrating common and rare variants [139] combine polygenic background with rare variant burden, enhancing predictive accuracy across ancestries. Graph neural networks like PRS-Net [140] model genes and pathways as networks, capturing epistatic dependencies [141] and potentially enhancing both interpretability and clinical actionability. Benchmark studies have shown that combining rare and common variant modeling improves cross-ancestry predictive performance, providing stronger clinical evidence for polygenic risk application. Realizing these advances will require continued efforts in both algorithmic innovation and data diversification to promote equitable benefits across populations.

## Implications and Limitations

Recent advances in AI-based variant prediction have shown substantial progress. DL approaches, particularly transformer architectures and pLMs, have improved sequence context capture and biological signals integration across diverse variant interpretation tasks.

However, important limitations constrain clinical translation of these findings. Reported performance gains are context-dependent, derived from specific benchmarks and curated datasets, and do not establish universal superiority of any single method. Differences in data composition, labeling practices, and evaluation protocols limit direct cross-method comparisons. Despite improved predictive accuracy, many models require further validation or

interpretability improvements for standalone clinical use, and challenges including ancestry bias and limited experimental validation persist.

These limitations underscore that AI-based VIPs function primarily as decision-support tools that complement expert judgment within established clinical workflows rather than as definitive classifiers. Continued progress requires standardized benchmarking across diverse populations, transparent reporting of model scope and limitations, development of interpretable architectures, and systematic experimental validation of computational predictions.

## Conclusions

In summary, the integration of diverse datasets, transparent predictive algorithms, and consistent validation practices will be critical for advancing AI-driven variant interpretation into routine clinical use. The rapid advancement of computational tools has significantly enhanced our ability to predict genetic variant pathogenicity, offering scalability and accuracy. However, several challenges remain, particularly around accurately classifying VUS, and ensuring fairness and transparency of predictive models. Continued improvement will require effectively integrating multi-omics information, expanding international cooperation to diversify genomic datasets, and systematically linking computational predictions to robust experimental validations. With these concerted efforts, next-generation computational tools can help realize the promise of personalized medicine, guiding clinicians and researchers toward deeper mechanistic insights and improved patient care.

## Data Availability

Not applicable.

## List of abbreviations

1KGP: 1000 Genomes Project; ACMG: American College of Medical Genetics and Genomics; AI: Artificial Intelligence; AIVAR: Artificial Intelligent Variant Classifier; AMP: Association for Molecular Pathology; AUC: Area Under the Curve; CADD: Combined Annotation Dependent Depletion; CAPICE: Consequence-Agnostic Pathogenicity Interpretation of Clinical Exome Variations; COSMIC: Catalogue of Somatic Mutations in Cancer; DANN: Deleterious Annotation of Genetic Variants using Neural Networks; dbSNP: Single Nucleotide Polymorphism Database; DL: Deep Learning; DoCM: Database of Curated Mutations; EVE: Evolutionary Model of Variant Effect; FATHMM: Functional Analysis Through Hidden Markov Models; GAVIN: Gene-Aware Variant INterpretation; GBM: Gradient Boosting Machine; GeneT: Genetic Transformer; GERP++: Genomic Evolutionary Rate Profiling ++; gnomAD: Genome Aggregation Database; GO: Gene Ontology; GWAVA: Genome-Wide Annotation of Variants; HGMD: Human Gene Mutation Database; HPO: Human Phenotype Ontology; HuVarBase: Human Variants Database; IRDs: Inherited Retinal Diseases; MAGPIE: Multimodal Annotation Generated Pathogenic Impact Evaluator; MAVEs: Multiplexed Assays of Variant Effect; M-CAP: Mendelian Clinically Applicable Pathogenicity; MetaSVM: Meta-analytic Support Vector Machine; ML: Machine Learning; OMIM: Online Mendelian Inheritance in Man; parSMURF: Parallel SMote Undersampled Random Forest; PDB: Protein Data Bank; PdmIRD: Prediction of Deleterious Missense Mutation for IRDs; PhD-SNP: Predictor of human Deleterious Single Nucleotide Polymorphisms; pLMs: Protein Language Models; PolyPhen: Polymorphism Phenotyping; PolyPhen-HCM: Polymorphism Phenotyping for Hypertrophic Cardiomyopathy; REVEL: Rare Exome Variant Ensemble Learner; RF: Random Forest; ROC: Receiver Operating Characteristic; SIFT: Sorting Intolerant From

Tolerant; SPANR: Splicing-based Analysis of Variants; SVM: Support Vector Machine; VariBench: Benchmark Database for Variations; VariPred: Variant impact Predictor; VEP: Variant Effect Predictor; VEST: Variant Effect Scoring Tool; VIPPID: Variant Impact Predictor for Primary Immunodeficiency Diseases; VIPs: Variant Impact Predictors; VUS: Variants of Uncertain Significance; XAI: Explainable Artificial Intelligence.

**Ethics approval and consent to participate**

Not applicable.

**Consent for publication**

Not applicable.

**Competing interests**

The authors declare that they have no competing interests.

**Funding**

This work was supported by the China–Serbia Science and Technology Cooperation Committee Exchange Program, Sixth Session (Project 6-3).

**Authors' contributions**

IP, MS: Conceptualization, Investigation, Methodology, Writing – original draft

HL, MW: Visualization

ZS, MF: Conceptualization, Supervision, Project administration, Writing – review & editing

All authors read and approved the final manuscript.

**Acknowledgments**

We would like to thank Dr. Merry Meryam Martgrita from the Department of Bioprocess Engineering at Institut Teknologi Del, and Riyanthi Angrainy Sianturi from the Department of Software Engineering at Institut Teknologi Del, for their valuable advice.

## References


[1] O. S. Aworunse, O. Adeniji, O. L. Oyesola, I. Isewon, J. Oyelade, and O. O. Obembe, "Genomic Interventions in Medicine," *Bioinformatics and Biology Insights,* vol. 12, 2018, doi: 10.1177/1177932218816100.

[2] S. M. Rego and M. P. Snyder, "High Throughput Sequencing and Assessing Disease Risk," *Cold Spring Harbor Perspectives in Medicine,* vol. 9, no. 1, 2019, doi: 10.1101/cshperspect.a026849.

[3] M. Spielmann and M. Kircher, "Computational and experimental methods for classifying variants of unknown clinical significance," (in eng), *Cold Spring Harb Mol Case Stud,* vol. 8, no. 3, Apr 2022, doi: 10.1101/mcs.a006196.

[4] Y. Boulaimen, G. Fossi, L. Outemzabet, N. Jeanray, O. Levenets, S. Gerart, *et al.*, "Integrating Large Language Models for Genetic Variant Classification," *arXiv preprint arXiv:2411.05055,* 2024.

[5] Y. Liu, T. Zhang, N. You, S. Wu, and N. Shen, "MAGPIE: accurate pathogenic prediction for multiple variant types using machine learning approach," *Genome Medicine,* vol. 16, no. 1, p. 3, 2024/01/08 2024, doi: 10.1186/s13073-023-01274-4.

[6] K. J. Karczewski, B. Weisburd, B. Thomas, M. Solomonson, D. M. Ruderfer, D. Kavanagh, *et al.*, "The ExAC browser: displaying reference data information from over 60 000 exomes," *Nucleic Acids Research,* vol. 45, no. D1, pp. D840-D845, 2017, doi: 10.1093/nar/gkw971.

[7] M. J. Landrum, J. M. Lee, G. R. Riley, W. Jang, W. S. Rubinstein, D. M. Church , *et al.*, "ClinVar: public archive of relationships among sequence variation and human phenotype," *Nucleic acids research,* vol. 42, no. Database issue, pp. D980-5, Jan 2014, doi: 10.1093/nar/gkt1113.

[8] S. T. Sherry, M.-H. Ward, M. Kholodov, J. Baker, L. Phan, E. M. Smigielski, *et al.*, "dbSNP: the NCBI database of genetic variation," (in eng), *Nucleic Acids Res,* vol. 29, no. 1, pp. 308-11, Jan 1 2001, doi: 10.1093/nar/29.1.308.

[9] 1000 Genomes Project Consortium, A. Auton, L.D. Brooks, R.M. Durbin, E.P. Garrison, H.M. Kang, *et al.*, "A global reference for human genetic variation," *Nature,* vol. 526, no. 7571, pp. 68-74, 2015, doi: 10.1038/nature15393.

[10] N. Allen, C. Sudlow, P. Downey, T. Peakman, J. Danesh., P. Elliott, *et al.*, "UK Biobank: Current status and what it means for epidemiology," *Health Policy and*

*Technology,* vol. 1, no. 3, pp. 123-126, 2012/09/01/ 2012, doi: https://doi.org/10.1016/j.hlpt.2012.07.003.

[11] K. J. Karczewski, L. C. Francioli, G. Tiao, B. B. Cummings, J. Alföldi, Q. Wang, *et al.*, "The mutational constraint spectrum quantified from variation in 141,456 humans," *Nature,* vol. 581, no. 7809, pp. 434-443, May 2020, doi: 10.1038/s41586-020-2308-7.

[12] H. Zhou, T. Arapoglou, X. Li, Z. Li, X. Zheng, J. Moore, *et al.*, "FAVOR: functional annotation of variants online resource and annotator for variation across the human genome," *Nucleic acids research,* vol. 51, no. D1, pp. D1300-D1311, Jan 6 2023, doi: 10.1093/nar/gkac966.

[13] D. N. Cooper, E. V. Ball, and M. Krawczak, "The human gene mutation database," *Nucleic acids research,* vol. 26, no. 1, pp. 285-7, Jan 1 1998, doi: 10.1093/nar/26.1.285.

[14] K. Ganesan, A. Kulandaisamy, S. Binny Priya, and M. M. Gromiha, "HuVarBase: A human variant database with comprehensive information at gene and protein levels," (in eng), *PLoS One,* vol. 14, no. 1, p. e0210475, 2019, doi: 10.1371/journal.pone.0210475.

[15] H. L. Rehm, J. S Berg, L. D. Brooks, C. D. Bustamante, J. P. Evans, M. J. Landrum, *et al.*, "ClinGen — The Clinical Genome Resource," *New England Journal of Medicine,* vol. 372, no. 23, pp. 2235-2242, 2015, doi: doi:10.1056/NEJMsr1406261.

[16] A. Hamosh, A. F. Scott, J. Amberger, D. Valle, and V. A. McKusick, "Online Mendelian Inheritance in Man (OMIM)," (in eng), *Hum Mutat,* vol. 15, no. 1, pp. 57-61, 2000, doi: 10.1002/(sici)1098-1004(200001)15:1<57::Aid-humu12>3.0.Co;2-g.

[17] S. S. Weinreich, R. Mangon, J. J. Sikkens, M. E. Teeuw, and M. C. Cornel, "[Orphanet: a European database for rare diseases]," (in dut), *Ned Tijdschr Geneeskd,* vol. 152, no. 9, pp. 518-9, Mar 1 2008. Orphanet: een Europese database over zeldzame ziekten.

[18] P. N. Robinson, S. Köhler, S. Bauer, D. Seelow, D. Horn, and S. Mundlos, "The Human Phenotype Ontology: a tool for annotating and analyzing human hereditary disease," (in eng), *Am J Hum Genet,* vol. 83, no. 5, pp. 610-5, Nov 2008, doi: 10.1016/j.ajhg.2008.09.017.

[19] M. Ashburner, C. A. Ball, J. A. Blake, D. Botstein, H. Butler, J. M. Cherry, *et al.*, "Gene ontology: tool for the unification of biology. The Gene Ontology Consortium," (in eng), *Nat Genet,* vol. 25, no. 1, pp. 25-9, May 2000, doi: 10.1038/75556.

[20] S. Bamford, E. Dawson, S. Forbes, J. Clements, R. Pettett, A. Dogan, *et al.*, "The COSMIC (Catalogue of Somatic Mutations in Cancer) database and website," (in eng), *Br J Cancer,* vol. 91, no. 2, pp. 355-8, Jul 19 2004, doi: 10.1038/sj.bjc.6601894.

[21] B. J. Ainscough, M. Griffith, A. C. Coffman, A. H. Wagner, J. Kunisaki, M. N. Choudhary *et al.*, "DoCM: a database of curated mutations in cancer," (in eng), *Nat Methods,* vol. 13, no. 10, pp. 806-7, Sep 29 2016, doi: 10.1038/nmeth.4000.

[22] Y. Liu, J. Sun, and M. Zhao, "ONGene: A literature-based database for human oncogenes," *Journal of Genetics and Genomics,* vol. 44, no. 2, pp. 119-121, 2017, doi: 10.1016/j.jgg.2016.12.004.

[23] G. M. Findlay, R. M. Daza, B. Martin, M. D. Zhang, A. P. Leith, M. Gasperini., *et al.*, "Accurate classification of BRCA1 variants with saturation genome editing," *Nature,* vol. 562, no. 7726, pp. 217-222, 2018, doi: 10.1038/s41586-018-0461-z.

[24] R. Leinonen, F. G. Diez, D. Binns, W. Fleischmann, R. Lopez, and R. Apweiler, "UniProt archive," (in eng), *Bioinformatics,* vol. 20, no. 17, pp. 3236-7, Nov 22 2004, doi: 10.1093/bioinformatics/bth191.

[25] H. M. Berman, J. Westbrook, Z. Feng, G. Gilliland, T. N. Bhat , H. Weissig , *et al.*, "The Protein Data Bank," (in eng), *Nucleic Acids Res,* vol. 28, no. 1, pp. 235-42, Jan 1 2000, doi: 10.1093/nar/28.1.235.

[26] P. Sasidharan Nair and M. Vihinen, "VariBench: a benchmark database for variations," (in eng), *Hum Mutat,* vol. 34, no. 1, pp. 42-9, Jan 2013, doi: 10.1002/humu.22204.

[27] G. C. Schaafsma and M. Vihinen, "VariSNP, a benchmark database for variations from dbSNP," (in eng), *Hum Mutat,* vol. 36, no. 2, pp. 161-6, Feb 2015, doi: 10.1002/humu.22727.

[28] Z. Wang, G. Zhao, Z. Zhu, Y. Wang, X. Xiang, S. Zhang, *et al.*, "VarCards2: an integrated genetic and clinical database for ACMG-AMP variant-interpretation guidelines in the human whole genome," *Nucleic Acids Research,* vol. 52, no. D1, pp. D1478-D1489, 2024, doi: 10.1093/nar/gkad1061.

[29] M. Yazar and P. Ozbek, "In Silico Tools and Approaches for the Prediction of Functional and Structural Effects of Single-Nucleotide Polymorphisms on Proteins: An Expert Review," *OMICS,* vol. 25, no. 1, pp. 23-37, Jan 2021, doi: 10.1089/omi.2020.0141.

[30] P. C. Ng and S. Henikoff, "Predicting deleterious amino acid substitutions," (in eng), *Genome Res,* vol. 11, no. 5, pp. 863-74, May 2001, doi: 10.1101/gr.176601.

[31] V. Ramensky, P. Bork, and S. Sunyaev, "Human non-synonymous SNPs: server and survey," (in eng), *Nucleic Acids Res,* vol. 30, no. 17, pp. 3894-900, Sep 1 2002, doi: 10.1093/nar/gkf493.

[32] K. J. van der Velde, E. N. de Boer, C. C. van Diemen, B. Sikkema-Raddatz, K. M. Abbott, A. Knopperts *et al.*, "GAVIN: Gene-Aware Variant INterpretation for medical sequencing," *Genome Biology,* vol. 18, no. 1, p. 6, 2017/01/16 2017, doi: 10.1186/s13059-016-1141-7.

[33] M. Kircher, D. M. Witten, P. Jain, B. J. O'Roak, G. M. Cooper, and J. Shendure, "A general framework for estimating the relative pathogenicity of human genetic variants," *Nat Genet,* vol. 46, no. 3, pp. 310-5, Mar 2014, doi: 10.1038/ng.2892.

[34] R. A. Ertürk and M. Baysan, "Utilizing Tree-Based Algorithms for Genetic Variant Interpretation," in *2024 9th International Conference on Computer Science and Engineering (UBMK)*, 26-28 Oct. 2024 2024, pp. 689-694, doi: 10.1109/UBMK63289.2024.10773498.

[35] B. Reva, Y. Antipin, and C. Sander, "Predicting the functional impact of protein mutations: application to cancer genomics," *Nucleic acids research,* vol. 39, no. 17, p. e118, Sep 1 2011, doi: 10.1093/nar/gkr407.

[36] H. A. Shihab, J. Gough, D. N. Cooper, P. D. Stenson, G. L. A. Barker, K. J. Edwards, *et al.*, "Predicting the functional, molecular, and phenotypic consequences of amino acid substitutions using hidden Markov models," *Hum Mutat,* vol. 34, no. 1, pp. 57-65, Jan 2013, doi: 10.1002/humu.22225.

[37] I. Ionita-Laza, K. McCallum, B. Xu, and J. D. Buxbaum, "A spectral approach integrating functional genomic annotations for coding and noncoding variants," *Nature Genetics,* vol. 48, no. 2, pp. 214-220, 2016, doi: 10.1038/ng.3477.

[38] E. V. Davydov, D. L. Goode, M. Sirota, G. M. Cooper, A. Sidow, and S. Batzoglou, "Identifying a high fraction of the human genome to be under selective constraint using GERP++," (in eng), *PLoS Comput Biol,* vol. 6, no. 12, p. e1001025, Dec 2 2010, doi: 10.1371/journal.pcbi.1001025.

[39] W. Burke, E. Parens, W. K. Chung, S. M. Berger, and P. S. Appelbaum, "The Challenge of Genetic Variants of Uncertain Clinical Significance : A Narrative Review," *Ann Intern Med,* vol. 175, no. 7, pp. 994-1000, Jul 2022, doi: 10.7326/M21-4109.

[40] S. J. MacEachern and N. D. Forkert, "Machine learning for precision medicine," *Genome,* vol. 64, no. 4, pp. 416-425, Apr 2021, doi: 10.1139/gen-2020-0131.

[41] I. A. Adzhubei, S. Schmidt, L. Peshkin, V. E. Ramensky, A. Gerasimova, P. Bork, *et al.*, "A method and server for predicting damaging missense mutations," *Nature Methods,* vol. 7, no. 4, pp. 248-249, 2010/04/01 2010, doi: 10.1038/nmeth0410-248.

[42] D. M. Jordan, A. Kiezun, S. M. Baxter, V. Agarwala, R. C. Green, M. F. Murray, *et al.*, "Development and validation of a computational method for assessment of missense variants in hypertrophic cardiomyopathy," *Am J Hum Genet,* vol. 88, no. 2, pp. 183-92, Feb 11 2011, doi: 10.1016/j.ajhg.2011.01.011.

[43] J. S. Kaminker, Y. Zhang, C. Watanabe, and Z. Zhang, "CanPredict: a computational tool for predicting cancer-associated missense mutations," *Nucleic acids research,* vol. 35, no. Web Server issue, pp. W595-8, Jul 2007, doi: 10.1093/nar/gkm405.

[44] H. Y. Xiong, B. Alipanahi, L. J. Lee, H. Bretschneider, D. Merico, R. K.C. Yuen *et al.*, "The human splicing code reveals new insights into the genetic determinants of disease," *Science,* vol. 347, no. 6218, p. 1254806, 2015, doi: doi:10.1126/science.1254806.

[45] E. Capriotti, R. Calabrese, and R. Casadio, "Predicting the insurgence of human genetic diseases associated to single point protein mutations with support vector machines and evolutionary information," *Bioinformatics,* vol. 22, no. 22, pp. 2729-2734, 2006, doi: 10.1093/bioinformatics/btl423.

[46] H. Carter, C. Douville, P. D. Stenson, D. N. Cooper, and R. Karchin, "Identifying Mendelian disease genes with the Variant Effect Scoring Tool," *BMC Genomics,* vol. 14, no. S3, 2013, doi: 10.1186/1471-2164-14-s3-s3.

[47] C. Dong *et al.*, "Comparison and integration of deleteriousness prediction methods for nonsynonymous SNVs in whole exome sequencing studies," *Human Molecular Genetics,* vol. 24, no. 8, pp. 2125-2137, 2014, doi: 10.1093/hmg/ddu733.

[48] B. Li, P. Wei, X. Jian, R. Gibbs, E. Boerwinkle, K. Wang, *et al.*, "Automated inference of molecular mechanisms of disease from amino acid substitutions," *Bioinformatics,* vol. 25, no. 21, pp. 2744-50, Nov 1 2009, doi: 10.1093/bioinformatics/btp528.

[49] D. Danis, J. O.B. Jacobsen, L. C. Carmody, M. A. Gargano, J. A. McMurry, A. Hegde, *et al.*, "Interpretable prioritization of splice variants in diagnostic next-generation sequencing," *The American Journal of Human Genetics,* vol. 108, no. 9, pp. 1564-1577, 2021, doi: 10.1016/j.ajhg.2021.06.014.

[50] G. R. Ritchie, I. Dunham, E. Zeggini, and P. Flicek, "Functional annotation of noncoding sequence variants," (in eng), *Nat Methods,* vol. 11, no. 3, pp. 294-6, Mar 2014, doi: 10.1038/nmeth.2832.

[51] M. Fang, Z. Su, H. Abolhassani, Y. Itan, X. Jin, and L. Hammarstrom, "VIPPID: a gene-specific single nucleotide variant pathogenicity prediction tool for primary

immunodeficiency diseases," *Brief Bioinform,* vol. 23, no. 5, Sep 20 2022, doi: 10.1093/bib/bbac176.

[52] B. Zeng, D. C. Liu, J. G. Huang, X. B. Xia, and B. Qin, "PdmIRD: missense variants pathogenicity prediction for inherited retinal diseases in a disease-specific manner," *Human genetics,* vol. 143, no. 3, pp. 331-342, Mar 2024, doi: 10.1007/s00439-024-02645-6.

[53] S. Li, K. J. van der Velde, D. de Ridder, A. D. J. van Dijk, D. Soudis, L. R. Zwerwer, *et al.*, "CAPICE: a computational method for Consequence-Agnostic Pathogenicity Interpretation of Clinical Exome variations," *Genome Medicine,* vol. 12, no. 1, 2020, doi: 10.1186/s13073-020-00775-w.

[54] Y. Wei, T. Zhang, B. Wang, X. Jiang, F. Ling, M. Fang, *et al.*, "INDELpred: Improving the prediction and interpretation of indel pathogenicity within the clinical genome," (in eng), *HGG Adv,* vol. 5, no. 4, p. 100325, Oct 10 2024, doi: 10.1016/j.xhgg.2024.100325.

[55] K. A. Jagadeesh, A. M. Wenger, M. J. Berger, H. Guturu, P. D. Stenson, D. N. Cooper, *et al.*, "M-CAP eliminates a majority of variants of uncertain significance in clinical exomes at high sensitivity," *Nature Genetics,* vol. 48, no. 12, pp. 1581-1586, 2016, doi: 10.1038/ng.3703.

[56] M. Kabir, S. Ahmed, H. Zhang, I. Rodríguez-Rodríguez, S. M. Najibi, and M. Vihinen, "PON-P3: Accurate Prediction of Pathogenicity of Amino Acid Substitutions," (in eng), *Int J Mol Sci,* vol. 26, no. 5, Feb 25 2025, doi: 10.3390/ijms26052004.

[57] X. Zhang, R. Walsh, N. Whiffin, R. Buchan, W. Midwinter, A. Wilk, *et al.*, "Disease-specific variant pathogenicity prediction significantly improves variant interpretation in inherited cardiac conditions," *Genet Med,* vol. 23, no. 1, pp. 69-79, Jan 2021, doi: 10.1038/s41436-020-00972-3.

[58] A. C. Gunning, V. Fryer, J. Fasham, A. H. Crosby, S. Ellard, E. L. Baple, *et al.*, "Assessing performance of pathogenicity predictors using clinically relevant variant datasets," *J Med Genet,* vol. 58, no. 8, pp. 547-555, Aug 2021, doi: 10.1136/jmedgenet-2020-107003.

[59] S. Whalen and G. Pandey, "A Comparative Analysis of Ensemble Classifiers: Case Studies in Genomics," in *2013 IEEE 13th International Conference on Data Mining*, 7-10 Dec. 2013 2013, pp. 807-816, doi: 10.1109/ICDM.2013.21.

[60] N. M. Ioannidis, J. H. Rothstein, V. Pejaver, S. Middha, S. K. McDonnell, S. Baheti, *et al.*, "REVEL: An Ensemble Method for Predicting the Pathogenicity of Rare

Missense Variants," *Am J Hum Genet,* vol. 99, no. 4, pp. 877-885, Oct 6 2016, doi: 10.1016/j.ajhg.2016.08.016.

[61] A. Petrini, M. Mesiti, M. Schubach, M. Frasca, D. Danis, M. Re, G. Grossi, *et al.*, "parSMURF, a high-performance computing tool for the genome-wide detection of pathogenic variants," *Gigascience,* vol. 9, no. 5, May 1 2020, doi: 10.1093/gigascience/giaa052.

[62] J. Zou, M. Huss, A. Abid, P. Mohammadi, A. Torkamani, and A. Telenti, "A primer on deep learning in genomics," *Nat Genet,* vol. 51, no. 1, pp. 12-18, Jan 2019, doi: 10.1038/s41588-018-0295-5.

[63] D. Quang, Y. Chen, and X. Xie, "DANN: a deep learning approach for annotating the pathogenicity of genetic variants," *Bioinformatics,* vol. 31, no. 5, pp. 761-3, Mar 1 2015, doi: 10.1093/bioinformatics/btu703.

[64] L. Sundaram, H. Gao, S.R. Padigepati, J.F. McRae,Y. Li, J.A. Kosmicki, *et al.*, "Predicting the clinical impact of human mutation with deep neural networks," *Nature Genetics,* vol. 50, no. 8, pp. 1161-1170, 2018, doi: 10.1038/s41588-018-0167-z.

[65] J. Zhou and O. G. Troyanskaya, "Predicting effects of noncoding variants with deep learning-based sequence model," *Nat Methods,* vol. 12, no. 10, pp. 931-4, Oct 2015, doi: 10.1038/nmeth.3547.

[66] D. R. Kelley, Y. A. Reshef, M. Bileschi, D. Belanger, C. Y. McLean, and J. Snoek, "Sequential regulatory activity prediction across chromosomes with convolutional neural networks," (in eng), *Genome Res,* vol. 28, no. 5, pp. 739-750, May 2018, doi: 10.1101/gr.227819.117.

[67] K. Jaganathan, N. Ersaro, G. Novakovsky, Y. Wang, T. James, J. Schwartzentruber, *et al.*, "Predicting expression-altering promoter mutations with deep learning," *Science,* vol. 0, no. 0, p. eads7373, doi: doi:10.1126/science.ads7373.

[68] K. Jaganathan, *et al.*, "Predicting Splicing from Primary Sequence with Deep Learning," *Cell,* vol. 176, no. 3, pp. 535-548.e24, 2019, doi: 10.1016/j.cell.2018.12.015.

[69] J. Cheng, S. K. Panagiotopoulou, J. F. McRae, S. F. Darbandi, D. Knowles, Y. I. Li, *et al.*, "MMSplice: modular modeling improves the predictions of genetic variant effects on splicing," *Genome Biology,* vol. 20, no. 1, p. 48, 2019/03/01 2019, doi: 10.1186/s13059-019-1653-z.

[70] Y. LeCun, Y. Bengio, and G. Hinton, "Deep learning," *Nature,* vol. 521, no. 7553, pp. 436-444, 2015/05/01 2015, doi: 10.1038/nature14539.

[71] J. Cheng, G. Novati, J. Pan, C. Bycroft , A. emgulyt , T. Applebaum, *et al.*, "Accurate proteome-wide missense variant effect prediction with AlphaMissense," *Science,* vol. 381, no. 6664, p. eadg7492, Sep 22 2023, doi: 10.1126/science.adg7492.

[72] A. Vaswani, N. Shazeer, N. Parmar, J. Uszkoreit, L. Jones, A. N. Gomez, *et al.*, "Attention is all you need," presented at the Proceedings of the 31st International Conference on Neural Information Processing Systems, Long Beach, California, USA, 2017.

[73] A. Rives, J. Meier, T. Sercu, S. Goyal, Z. Lin, J. Liu, *et al.*, "Biological structure and function emerge from scaling unsupervised learning to 250 million protein sequences," (in eng), *Proc Natl Acad Sci U S A,* vol. 118, no. 15, Apr 13 2021, doi: 10.1073/pnas.2016239118.

[74] N. Brandes, G. Goldman, C. H. Wang, C. J. Ye, and V. Ntranos, "Genome-wide prediction of disease variant effects with a deep protein language model," *Nature Genetics,* vol. 55, no. 9, pp. 1512-1522, 2023/09/01 2023, doi: 10.1038/s41588-023-01465-0.

[75] W. Lin, J. Wells, Z. Wang, C. Orengo, and A. C. R. Martin, "Enhancing missense variant pathogenicity prediction with protein language models using VariPred," *Sci Rep,* vol. 14, no. 1, p. 8136, Apr 7 2024, doi: 10.1038/s41598-024-51489-7.

[76] A. V. Kulikova, D. J. Diaz, T. Chen, T. J. Cole, A. D. Ellington, and C. O. Wilke, "Two sequence- and two structure-based ML models have learned different aspects of protein biochemistry," *Scientific Reports,* vol. 13, no. 1, p. 13280, 2023/08/16 2023, doi: 10.1038/s41598-023-40247-w.

[77] C. Marquet, M. Heinzinger, T. Olenyi, C. Dallago, K. Erckert, M. Bernhofer, *et al.*, "Embeddings from protein language models predict conservation and variant effects," *Human Genetics,* vol. 141, no. 10, pp. 1629-1647, 2022/10/01 2022, doi: 10.1007/s00439-021-02411-y.

[78] Ž. Avsec, V. Agarwal, D. Visentin, J. R. Ledsam, A. Grabska-Barwinska, K. R. Taylor, *et al.*, "Effective gene expression prediction from sequence by integrating long-range interactions," *Nature Methods,* vol. 18, no. 10, pp. 1196-1203, 2021/10/01 2021, doi: 10.1038/s41592-021-01252-x.

[79] Ž. Avsec, N. Latysheva, J. Cheng, G. Novati, K. R. Taylor, T. Ward, *et al.*, "AlphaGenome: advancing regulatory variant effect prediction with a unified DNA

sequence model," *bioRxiv,* p. 2025.06.25.661532, 2025, doi: 10.1101/2025.06.25.661532.

[80] Z. Gao, Q. Liu, W. Zeng, R. Jiang, and W. H. Wong, "EpiGePT: a pretrained transformer-based language model for context-specific human epigenomics," *Genome Biology,* vol. 25, no. 1, p. 310, 2024/12/18 2024, doi: 10.1186/s13059-024-03449-7.

[81] J. Jumper, R. Evans, A. Pritzel, T. Green, M. Figurnov, O. Ronneberger, *et al.*, "Highly accurate protein structure prediction with AlphaFold," *Nature,* vol. 596, no. 7873, pp. 583-589, 2021/08/01 2021, doi: 10.1038/s41586-021-03819-2.

[82] T. T. Jiang, L. Fang, and K. Wang, "Deciphering "the language of nature": A transformer-based language model for deleterious mutations in proteins," *The Innovation,* vol. 4, no. 5, p. 100487, 2023/09/11/ 2023, doi: https://doi.org/10.1016/j.xinn.2023.100487.

[83] L. Liang, Y. Chen, T. Wang, D. Jiang, J. Jin, Y. Pang, *et al.*, "Genetic Transformer: An Innovative Large Language Model Driven Approach for Rapid and Accurate Identification of Causative Variants in Rare Genetic Diseases," *medRxiv,* p. 2024.07.18.24310666, 2024, doi: 10.1101/2024.07.18.24310666.

[84] N. You, C. van Dijk, I. Timpanaro, P. Hop, B. Kenna, M. Kooyman, *et al.*, "SpliceTransformer predicts tissue-specific splicing linked to human diseases," (in eng), *Nat Commun,* vol. 15, no. 1, p. 9129, Oct 23 2024, doi: 10.1038/s41467-024-53088-6.

[85] J. Luo, *et al.*, "Assessing concordance among human, in silico predictions and functional assays on genetic variant classification," *Bioinformatics,* vol. 35, no. 24, pp. 5163-5170, 2019, doi: 10.1093/bioinformatics/btz442.

[86] W. McLaren, T. Zhou, X. You, Y. Zi, X. Li, Y. Wu, *et al.*, "The Ensembl Variant Effect Predictor," *Genome Biology,* vol. 17, no. 1, p. 122, 2016/06/06 2016, doi: 10.1186/s13059-016-0974-4.

[87] K. Wang, M. Li, and H. Hakonarson, "ANNOVAR: functional annotation of genetic variants from high-throughput sequencing data," (in eng), *Nucleic acids research,* vol. 38, no. 16, p. e164, Sep 2010, doi: 10.1093/nar/gkq603.

[88] C. Kopanos, V. Tsiolkas, A. Kouris, C. E. Chapple, M. A. Aguilera, R. Meyer, A. Massouras, *et al.*, "VarSome: the human genomic variant search engine," (in eng), *Bioinformatics,* vol. 35, no. 11, pp. 1978-1980, Jun 1 2019, doi: 10.1093/bioinformatics/bty897.

[89] D. Baux, C. V. Goethem, O. Ardouin, T. Guignard, A. Bergougnoux, M. Koenig, *et al.*, "MobiDetails: online DNA variants interpretation," *European Journal of Human*

*Genetics,* vol. 29, no. 2, pp. 356-360, 2021/02/01 2021, doi: 10.1038/s41431-020-00755-z.

[90] Q. Li and K. Wang, "InterVar: Clinical Interpretation of Genetic Variants by the 2015 ACMG-AMP Guidelines," *The American Journal of Human Genetics,* vol. 100, no. 2, pp. 267-280, 2017, doi: 10.1016/j.ajhg.2017.01.004.

[91] P. N. Robinson, S. Köhler, A. Oellrich; Sanger Mouse Genetics Project; K. Wang, C. J. Mungall, *et al.*, "Improved exome prioritization of disease genes through cross-species phenotype comparison," (in eng), *Genome research,* vol. 24, no. 2, pp. 340-348, 2014/02// 2014, doi: 10.1101/gr.160325.113.

[92] K. Maharana, S. Mondal, and B. Nemade, "A review: Data pre-processing and data augmentation techniques," *Global Transitions Proceedings,* vol. 3, no. 1, pp. 91-99, 2022/06/01/ 2022, doi: https://doi.org/10.1016/j.gltp.2022.04.020.

[93] A. Pfob, S.-C. Lu, and C. Sidey-Gibbons, "Machine learning in medicine: a practical introduction to techniques for data pre-processing, hyperparameter tuning, and model comparison," *BMC Medical Research Methodology,* vol. 22, no. 1, p. 282, 2022/11/01 2022, doi: 10.1186/s12874-022-01758-8.

[94] C. Marzban, "The ROC Curve and the Area under It as Performance Measures," (in English), *Weather and Forecasting,* vol. 19, no. 6, pp. 1106-1114, 01 Dec. 2004 2004, doi: https://doi.org/10.1175/825.1.

[95] P. Krusche, L. Trigg, P. C. Boutros, C. E. Mason, F. M. De La Vega, B. L. Moore, *et al.*, "Best practices for benchmarking germline small-variant calls in human genomes," (in eng), *Nat Biotechnol,* vol. 37, no. 5, pp. 555-560, May 2019, doi: 10.1038/s41587-019-0054-x.

[96] M. Zitnik, F. Nguyen, B. Wang, J. Leskovec, A. Goldenberg, and M. M. Hoffman, "Machine Learning for Integrating Data in Biology and Medicine: Principles, Practice, and Opportunities," (in eng), *Inf Fusion,* vol. 50, pp. 71-91, Oct 2019, doi: 10.1016/j.inffus.2018.09.012.

[97] P. Charilaou and R. Battat, "Machine learning models and over-fitting considerations," (in eng), *World J Gastroenterol,* vol. 28, no. 5, pp. 605-607, Feb 7 2022, doi: 10.3748/wjg.v28.i5.605.

[98] L. Hoffman-Andrews, "The known unknown: the challenges of genetic variants of uncertain significance in clinical practice," (in eng), *J Law Biosci,* vol. 4, no. 3, pp. 648-657, Dec 2017, doi: 10.1093/jlb/lsx038.

[99] T. Schnabel, A. Swaminathan, A. Singh, N. Chandak, and T. Joachims, "Recommendations as Treatments: Debiasing Learning and Evaluation," in *International Conference on Machine Learning*, 2016.

[100] R. Karimi, C. Freudenthaler, A. Nanopoulos, and L. Schmidt-Thieme, "Towards Optimal Active Learning for Matrix Factorization in Recommender Systems," in *2011 IEEE 23rd International Conference on Tools with Artificial Intelligence*, 7-9 Nov. 2011 2011, pp. 1069-1076, doi: 10.1109/ICTAI.2011.182.

[101] M. Gasperini, L. Starita, and J. Shendure, "The power of multiplexed functional analysis of genetic variants," (in eng), *Nat Protoc,* vol. 11, no. 10, pp. 1782-7, Oct 2016, doi: 10.1038/nprot.2016.135.

[102] A. E. McEwen, M. Tejura, S. Fayer, L. M. Starita, and D. M. Fowler, "Multiplexed assays of variant effect for clinical variant interpretation," (in eng), *Nat Rev Genet,* Jul 21 2025, doi: 10.1038/s41576-025-00870-x.

[103] L. G. Landry, N. Ali, D. R. Williams, H. L. Rehm, and V. L. Bonham, "Lack Of Diversity In Genomic Databases Is A Barrier To Translating Precision Medicine Research Into Practice," *Health Aff (Millwood),* vol. 37, no. 5, pp. 780-785, May 2018, doi: 10.1377/hlthaff.2017.1595.

[104] A. R. Martin, M. Kanai, Y. Kamatani, Y. Okada, B. M. Neale, and M. J. Daly, "Clinical use of current polygenic risk scores may exacerbate health disparities," *Nature genetics,* vol. 51, no. 4, pp. 584-591, Apr 2019, doi: 10.1038/s41588-019-0379-x.

[105] L. A. Hindorff, V. L. Bonham, and L. Ohno-Machado, "Enhancing diversity to reduce health information disparities and build an evidence base for genomic medicine," *Per Med,* vol. 15, no. 5, pp. 403-412, Sep 2018, doi: 10.2217/pme-2018-0037.

[106] D. Curtis, "Assessment of ability of AlphaMissense to identify variants affecting susceptibility to common disease," (in eng), *Eur J Hum Genet,* vol. 32, no. 11, pp. 1419-1427, Nov 2024, doi: 10.1038/s41431-024-01675-y.

[107] E. A. Boyle, Y. I. Li, and J. K. Pritchard, "An Expanded View of Complex Traits: From Polygenic to Omnigenic," (in eng), *Cell,* vol. 169, no. 7, pp. 1177-1186, Jun 15 2017, doi: 10.1016/j.cell.2017.05.038.

[108] J. Frazer, P. Notin, M. Dias, A. Gomez, J. K. Min, K. Brock, *et al.*, "Disease variant prediction with deep generative models of evolutionary data," *Nature,* vol. 599, no. 7883, pp. 91-95, Nov 2021, doi: 10.1038/s41586-021-04043-8.

[109] A. Raz, B. Heinrichs, N. Avnoon, G. Eyal, and Y. Inbar, "Prediction and explainability in AI: Striking a new balance?," *Big Data & Society,* vol. 11, no. 1, p. 20539517241235871, 2024, doi: 10.1177/20539517241235871.

[110] Z. Zheng, Y. Wang, Y. Huang , S. Song, M. Yang, B. Tang, *et al.*, "Attention heads of large language models," *Patterns (N Y),* vol. 6, no. 2, p. 101176, Feb 14 2025, doi: 10.1016/j.patter.2025.101176.

[111] I. R. Mallela, S. Aravind, O. Tharan, D. P. Goel, D. S. Pal, and Singh, "Explainable AI for Compliance and Regulatory Models," *International Journal for Research Publication and Seminar,* 2020.

[112] K. D. Doig, R. Perera, Y. Kankanige, A. Fellowes, J. Li, R. Lupat, *et al.*, "Using artificial intelligence (AI) to model clinical variant reporting for next generation sequencing (NGS) oncology assays," (in eng), *BioData Min,* vol. 18, no. 1, p. 74, Oct 29 2025, doi: 10.1186/s13040-025-00489-y.

[113] M. Costa, A. García S, A. León, and O. Pastor, "The promises and pitfalls of automated variant interpretation: a comprehensive review," *Briefings in Bioinformatics,* vol. 26, no. 5, 2025, doi: 10.1093/bib/bbaf545.

[114] D. Stein, M. E. Kars, B. Milisavljevic, M. Mort, P. D. Stenson, J. Casanova, *et al.*, "Expanding the utility of variant effect predictions with phenotype-specific models," *Nature Communications,* vol. 16, no. 1, p. 11113, 2025/11/28 2025, doi: 10.1038/s41467-025-66607-w.

[115] I. Subramanian, S. Verma, S. Kumar, A. Jere, and K. Anamika, "Multi-omics Data Integration, Interpretation, and Its Application," (in eng), *Bioinform Biol Insights,* vol. 14, p. 1177932219899051, 2020, doi: 10.1177/1177932219899051.

[116] T. Yang, P. Wei, and W. Pan, "Integrative analysis of multi-omics data for discovering low-frequency variants associated with low-density lipoprotein cholesterol levels," *Bioinformatics,* vol. 36, no. 21, pp. 5223-5228, 2020, doi: 10.1093/bioinformatics/btaa898.

[117] C. Y. Hsu, S. Askar, S. S. Alshkarchy, P. P. Nayak, K. A. L. Attabi, M. A. Khan, *et al.*, "AI-driven multi-omics integration in precision oncology: bridging the data deluge to clinical decisions," (in eng), *Clin Exp Med,* vol. 26, no. 1, p. 29, Nov 21 2025, doi: 10.1007/s10238-025-01965-9.

[118] E. Hernandez-Lemus and S. Ochoa, "Methods for multi-omic data integration in cancer research," *Front Genet,* vol. 15, p. 1425456, 2024, doi: 10.3389/fgene.2024.1425456.

[119] M. M.-C. Chui, A. K.-Y. Kwong, H. Y. C. Leung, C. Pang, I. F. Scheller, S. S.-N. Wong, *et al.*, "An outlier approach: advancing diagnosis of neurological diseases through integrating proteomics into multi-omics guided exome reanalysis," *npj Genomic Medicine,* vol. 10, no. 1, p. 36, 2025/05/03 2025, doi: 10.1038/s41525-025-00493-5.

[120] D. Smirnov, N. Konstantinovskiy, and H. Prokisch, "Integrative omics approaches to advance rare disease diagnostics," (in eng), *J Inherit Metab Dis,* vol. 46, no. 5, pp. 824-838, Sep 2023, doi: 10.1002/jimd.12663.

[121] L. Gerasimavicius, S. A. Teichmann, and J. A. Marsh, "Leveraging protein structural information to improve variant effect prediction," (in eng), *Curr Opin Struct Biol,* vol. 92, p. 103023, Jun 2025, doi: 10.1016/j.sbi.2025.103023.

[122] N. Ding, Y. Qin, G. Yang, F. Wei, Z. Yang, Y. Su, *et al.*, "Parameter-efficient fine-tuning of large-scale pre-trained language models," *Nature Machine Intelligence,* vol. 5, no. 3, pp. 220-235, 2023/03/01 2023, doi: 10.1038/s42256-023-00626-4.

[123] L. Wang, X. Chen, X.W. Deng, H. Wen, M.K. You, W.Z. Liu, *et al.*, "Prompt engineering in consistency and reliability with the evidence-based guideline for LLMs," *npj Digital Medicine,* vol. 7, no. 1, p. 41, 2024/02/20 2024, doi: 10.1038/s41746-024-01029-4.

[124] H. Feng, L. Wu, B. Zhao, C. Huff, J. Zhang, J. Wu, *et al.*, "Benchmarking DNA foundation models for genomic and genetic tasks," *Nature Communications,* vol. 16, no. 1, p. 10780, 2025/11/28 2025, doi: 10.1038/s41467-025-65823-8.

[125] A. R. Sajun, I. Zualkernan, and D. Sankalpa, "A historical survey of advances in transformer architectures," *Applied Sciences,* vol. 14, no. 10, p. 4316, 2024.

[126] D. M. Fowler, D. J. Adams, A. L. Gloyn, W. C. Hahn, D. S. Marks, L. A. Muffley, *et al.*, "An Atlas of Variant Effects to understand the genome at nucleotide resolution," *Genome Biology,* vol. 24, no. 1, p. 147, 2023/07/03 2023, doi: 10.1186/s13059-023-02986-x.

[127] A. F. Rubin, J. Stone, A. H. Bianchi, B. J. Capodanno, E. Y. Da, M. Dias, *et al.*, "MaveDB 2024: a curated community database with over seven million variant effects from multiplexed functional assays," *Genome Biology,* vol. 26, no. 1, p. 13, 2025/01/21 2025, doi: 10.1186/s13059-025-03476-y.

[128] P. Buphamalai, T. Kokotovic, V. Nagy, and J. Menche, "Network analysis reveals rare disease signatures across multiple levels of biological organization," *Nature*

*Communications,* vol. 12, no. 1, p. 6306, 2021/11/09 2021, doi: 10.1038/s41467-021-26674-1.

[129] C. Dandara, F. Huzair, A. Borda-Rodriguez, S. Chirikure, I. Okpechi, L. Warnich, *et al.*, "H3Africa and the African life sciences ecosystem: building sustainable innovation," (in eng), *Omics,* vol. 18, no. 12, pp. 733-9, Dec 2014, doi: 10.1089/omi.2014.0145.

[130] Z. Zhao, L. G. Fritsche, J. A. Smith, B. Mukherjee, and S. Lee, "The construction of cross-population polygenic risk scores using transfer learning," (in eng), *Am J Hum Genet,* vol. 109, no. 11, pp. 1998-2008, Nov 3 2022, doi: 10.1016/j.ajhg.2022.09.010.

[131] P. Tian, T. H. Chan, Y. F. Wang, W. Yang, G. Yin, and Y. D. Zhang, "Multiethnic polygenic risk prediction in diverse populations through transfer learning," (in eng), *Front Genet,* vol. 13, p. 906965, 2022, doi: 10.3389/fgene.2022.906965.

[132] E. Tzeng, J. Hoffman, K. Saenko, and T. Darrell, "Adversarial discriminative domain adaptation," in *Proceedings of the IEEE conference on computer vision and pattern recognition*, 2017, pp. 7167-7176.

[133] M. C. Cara, D. M. Montserrat, and A. G. Ioannidis, "PopGenAdapt: Semi-Supervised Domain Adaptation for Genotype-to-Phenotype Prediction in Underrepresented Populations," in *Biocomputing 2024*, pp. 327-340.

[134] B. Yelmen, A. Decelle, L. Ongaro, D. Marnetto, C. Tallec, F. Montinaro, *et al.*, "Creating artificial human genomes using generative neural networks," (in eng), *PLoS Genet,* vol. 17, no. 2, p. e1009303, Feb 2021, doi: 10.1371/journal.pgen.1009303.

[135] S. Das and X. Shi, "Offspring GAN augments biased human genomic data," presented at the Proceedings of the 13th ACM International Conference on Bioinformatics, Computational Biology and Health Informatics, Northbrook, Illinois, 2022. [Online]. Available: https://doi.org/10.1145/3535508.3545537.

[136] H. Zhang, J. Zhan, J. Jin, J. Zhang, W. Lu, R. Zhao, *et al.*, "A new method for multiancestry polygenic prediction improves performance across diverse populations," (in eng), *Nat Genet,* vol. 55, no. 10, pp. 1757-1768, Oct 2023, doi: 10.1038/s41588-023-01501-z.

[137] J. Zhang, J. Zhan, J. Jin, C. Ma, R. Zhao, J. O'Connell, *et al.*, "An ensemble penalized regression method for multi-ancestry polygenic risk prediction," *Nature Communications,* vol. 15, no. 1, p. 3238, 2024/04/15 2024, doi: 10.1038/s41467-024-47357-7.

[138] C. Gao, J. D. Tubbs, Y. Han, M. Guo, S. Li, E. Ma, *et al.*, "Unsupervised Ensemble Learning for Efficient Integration of Pre-trained Polygenic Risk Scores," (in eng), *medRxiv,* Mar 20 2025, doi: 10.1101/2025.01.06.25320058.

[139] J. Williams, T. Chen, X. Hua, W. Wong, K. Yu, P. Kraft, *et al.*, "Integrating Common and Rare Variants Improves Polygenic Risk Prediction Across Diverse Populations," *medRxiv,* p. 2024.11.05.24316779, 2024, doi: 10.1101/2024.11.05.24316779.

[140] H. Li, J. Zeng, M. P. Snyder, and S. Zhang, "PRS-Net: Interpretable Polygenic Risk Scores via Geometric Learning," Cham, 2024: Springer Nature Switzerland, in Research in Computational Molecular Biology, pp. 377-380.

[141] H. Li, J. Zeng, M. P. Snyder, and S. Zhang, "Modeling gene interactions in polygenic prediction via geometric deep learning," (in eng), *Genome Res,* vol. 35, no. 1, pp. 178-187, Jan 22 2025, doi: 10.1101/gr.279694.124.

## Figure Legends

**Figure 1**. Evolution of *in silico* Tools for Predicting Genetic Variant Pathogenicity

This figure illustrates the temporal progression of computational approaches for genetic variant pathogenicity prediction. The evolutionary trajectory is categorized into four phases: (1) Early Stages, characterized by rudimentary rule-based algorithms; (2) The Emergence of Predictive Tools, marking the transition to more sophisticated statistical frameworks; (3) Machine Learning Era, defined by the integration of supervised and unsupervised learning methodologies; and (4) Advanced AI, representing contemporary approaches that leverage deep learning architectures and multi-modal data integration.

**Figure 2.** Integrated Pipeline for Variant Pathogenicity Prediction

This figure delineates a systematic framework for developing variant pathogenicity prediction models. The pipeline harnesses multi-modal data inputs (genomic, proteomic, molecular, and clinical) acquired from public repositories, experimental assays, clinical documentation, and literature curation. Critical preprocessing steps include variant annotation, normalization, cross-platform integration, and feature engineering to enhance signal integrity. The analytical workflow culminates in a machine learning implementation with rigorous hyperparameter optimization and comprehensive performance assessment to ensure robust predictive capacity across diverse genetic contexts.

**Figure 3.** Transformer-Based Framework for Variant Pathogenicity Prediction

This figure illustrates a transformer-based pipeline for predicting genetic variant pathogenicity. Input sequences ($X \in R^{B \times L \times D}$, where X represents the input tensor, R denotes real numbers, B = batch size, L = sequence length, D = embedding dimension) are processed through three key stages: (1) tokenization and embedding, (2) contextual encoding via multi-head self-attention, and (3) classification. The model undergoes pre-training on multi-omics datasets followed by pathogenicity-specific fine-tuning. Prompt engineering facilitates performance with limited

labeled data. Other key components include LayerNorm to mitigate vanishing or exploding gradients, Fully Connected Layers for effective feature extraction, and Softmax activation for generating robust probability distributions. Together, the overall architecture and training strategies enable the model to leverage latent biological mechanisms for accurate variant classification.

**Table 1.** Genomic Database Overview

| No. | Name | Description | Data Types | Cross-link | Entries (as of September 2025) | Last Updates | Website | Reference |
|---|---|---|---|---|---|---|---|---|
| 1 | 1KGP | Large-scale project to create a comprehensive resource on human genetic variation. | Population genomic data | Uses refID from dbSNP; variants included in gnomAD | >88 million variants (84.7M SNPs, 3.6M indels, 60k SVs) | 2024-11-18 | https://www.internationalgenome.org/ | [9] |
| 2 | *BRCA1* Dataset | Focused dataset on SNVs in the *BRCA1* gene. | Gene-specific genomic data | Variants cross-validated with ClinVar and HGMD | 3,893 SNVs | 2018-08-20 | https://sge.gs.washington.edu/BRCA1/ | [23] |
| 3 | ClinGen | Clinical genomics resource defining the clinical relevance of genes and variants. | Clinical genomics resource | Provides gene-disease evidence to ClinVar; uses HPO and OMIM | 3,256 genes, 11,062 variants | 2025-09-18 | https://clinicalgenome.org/ | [15] |
| 4 | ClinVar | Public archive of reports of the relationships among human variations and phenotypes. | Clinical genomic data | Integrates rsID from dbSNP; links to ClinGen, OMIM, HPO | 5,640,148 records (3,759,476 unique variants) | 2025-08-24 | https://www.ncbi.nlm.nih.gov/clinvar/ | [7] |
| 5 | COSMIC | Catalogue of somatic mutations in cancer. | Somatic genomic data | Overlaps with DoCM and ClinVar | 25,014,261 variants | 2025-05-21 | https://cancer.sanger.ac.uk/cosmic | [20] |
| 6 | dbSNP | Database of Single Nucleotide Polymorphisms and other variants. | Genomic variant registry | Referenced by ClinVar and gnomAD via rsID | 1,206,053,617 unique rs | 2025-01-15 | https://www.ncbi.nlm.nih.gov/snp/ | [8] |
| 7 | DoCM | Manually curated database of clinically relevant mutations. | Clinically curated genomic data | Overlaps with COSMIC and ClinVar | 3,818 variants | 2024-10-15 | http://www.docm.info/ | [21] |
| 8 | FAVOR | Aggregated variant and indel functional annotations from multiple databases. | Functional annotation data | Indexed by genomic coordinates and rsID; integrates multi-source functional annotations | 8,892,915,237 variants (8,812,917,339 SNVs and 79,997,898 indels) | 2025-02-05 | https://favor.genohub.org/ | [12] |
| 9 | gnomAD | Aggregated and harmonized human exome and genome sequencing data. | Population genomic data | Uses rsID from dbSNP, referenced in ClinVar | 730,947 exomes and 76,215 whole genomes | 2024-04-19 | https://gnomad.broadinstitute.org/ | [6] |

| No. | Name | Description | Data Types | Cross-link | Entries (as of September 2025) | Last Updates | Website | Reference |
|---|---|---|---|---|---|---|---|---|
| 10 | GO | Structured vocabulary for gene product functions and processes. | Ontology | Used in UniProt | 39,906 terms, 9.41 M annotations, 1.60 M gene products, 5,497 species | 2025-07-22 | http://www.geneontology.org/ | [19] |
| 11 | HGMD | Comprehensive collection of germline mutations in human genes. | Clinically curated genomic data | Often cross-validated with ClinVar and OMIM | 549,178 mutations | 2025-07-07 | http://www.hgmd.cf.ac.uk/ac/ index.php | [13] |
| 12 | HPO | Ontology for describing human phenotypic abnormalities. | Phenotype ontology | Used by OMIM and ClinGen; mapped to GO | 18,000 terms and over 156,000 annotations | 2024-04-19 | https://hpo.jax.org/ | [18] |
| 13 | HuVarBase | Annotated human variation database. | Curated genomic variant data | Merged from COSMIC, ClinVar, 1000 Genomes | 774,863 variants from 18,318 proteins (702,048 disease causing and 72,815 neutral variants) | 2018-06-01 | https://www.iitm.ac.in/bioinfo/huvarbase | [14] |
| 14 | OMIM | Comprehensive catalog of human genes and genetic phenotypes. | Gene–phenotype data | Includes HPO annotations; links to ClinVar and UniProt | 27,938 entries (26,394 Autosomal, 1,407 X Linked, 64 Y Linked, 73 Mitochondrial) | 2025-09-17 | https://www.omim.org/ | [16] |
| 15 | ONGene | Curated database of human oncogenes. | Cancer gene annotation data | - | 803 oncogenes (698 protein-coding genes + 105 non-coding) | 2016-12-26 | https://ongene.bioinfo-minzhao.org/ | [22] |
| 16 | Orphanet | Database for rare diseases and orphan drugs | Rare disease ontology | Shares HPO and OMIM terms | 9,785 Clinical entities 6,528 Rare disorders 8,296 Disease gene relationships 115,611 Phenotypic annotations 16,418 Epidemiological data 689 Orphan drugs 8,648 Expert centers | 2025-06-24 | https://www.orpha.net/ | [17] |
| 17 | PDB | Repository for 3D structural data of biological macromolecules. | Protein structural data | Linked from UniProt entries | 242,296 structures, 1,068,577 computed structure models | 2025-09-17 | https://www.rcsb.org/ | [25] |
| 18 | UK Biobank | Large-scale biomedical database with genetic and health information. | Population-scale genotype–phenotype data | Overlaps with dbSNP | 90 million variants | 2024-08-13 | https://www.ukbiobank.ac.uk/ | [10] |

| No. | Name | Description | Data Types | Cross-link | Entries (as of September 2025) | Last Updates | Website | Reference |
|---|---|---|---|---|---|---|---|---|
| 19 | UniProt / UniProtKB | Comprehensive protein sequence and annotation resource. | Protein sequence and functional annotation data | Cross-links to PDB, GO, dbSNP, ClinGen, Orphanet, | 253,206,171 entries (UniProtKB/Swiss-Prot: 572,970 entries and UniProtKB/TrEMBL: 252,633,201 entries) | 2025-06-17 | https://www.uniprot.org/ | [24] |
| 20 | VarCards2 | Updated version of VarCards, human variant annotation and interpretation. | Variant annotation and interpretation data | Some extracted from gnomAD, ClinVar, COSMIC, dbSNP | 368,820,266 indels, 2,773,555 CNVs | 2023-10-21 | http://www.genemed.tech/varcards2/ | [28] |
| 21 | VariBench | Benchmark database for variation datasets in bioinformatics. | Benchmark variant datasets | Some extracted from dbSNP, OMIM | >90 million variants | 2023-05-12 | https://structure.bmc.lu.se/VariBench/ | [26] |
| 22 | VariSNP | A benchmark database suite comprising variation datasets that can be used for developing and testing the performance of variant effect prediction | Benchmark variant data | Selected from dbSNP | 30,571,777 variants | 2017-02-16 | https://structure.bmc.lu.se/VariSNP/ | [27] |

Click here to access/download;Figure;Figures_5 Jan 2026.pdf

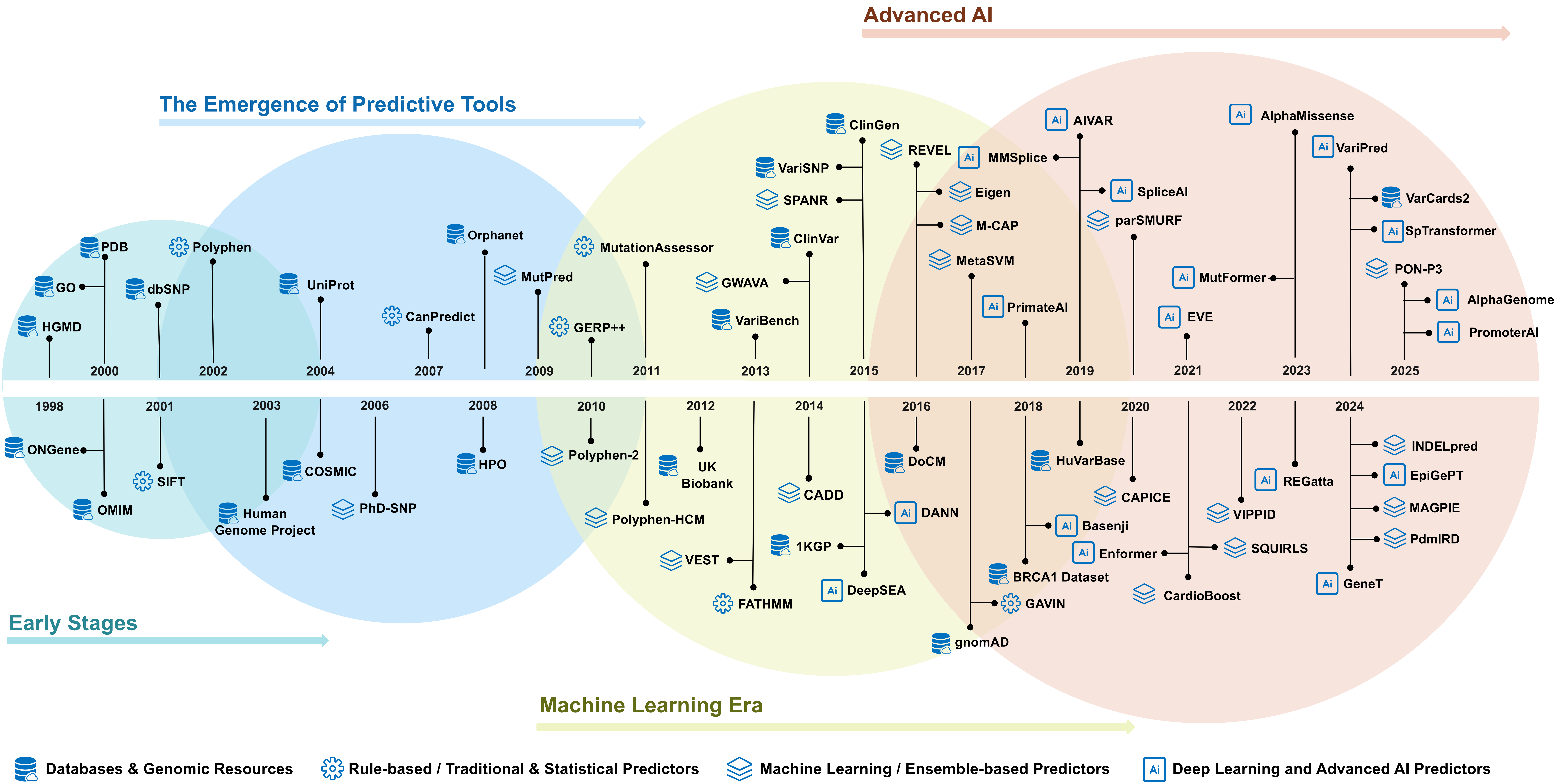

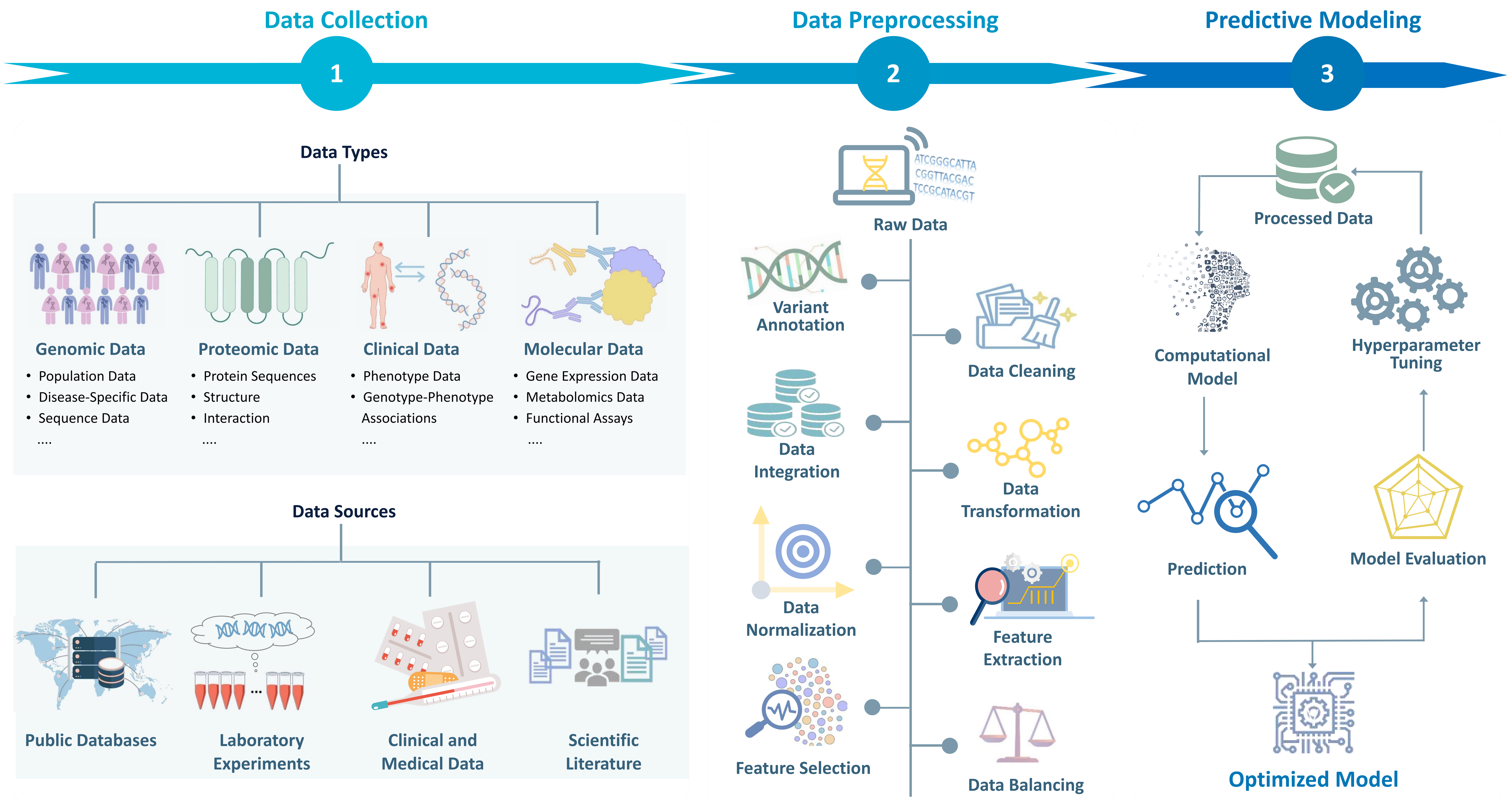

Data Collection
1
Data Preprocessing
2
Predictive Modeling
3
Data Types
Genomic Data
• Population Data
• Disease-Specific Data
• Sequence Data
....
Proteomic Data
• Protein Sequences
• Structure
• Interaction
....
Clinical Data
• Phenotype Data
• Genotype-Phenotype Associations
....
Molecular Data
• Gene Expression Data
• Metabolomics Data
• Functional Assays
....
Data Sources
Public Databases
Laboratory Experiments
Clinical and Medical Data
Scientific Literature
ATCGGGCATTA
CGGTTACGAC
TCCGCATACGT
Raw Data
Variant Annotation
Data Cleaning
Data Integration
Data Transformation
Data Normalization
Feature Extraction
Feature Selection
Data Balancing
Processed Data
Computational Model
Hyperparameter Tuning
Prediction
Model Evaluation
Optimized Model

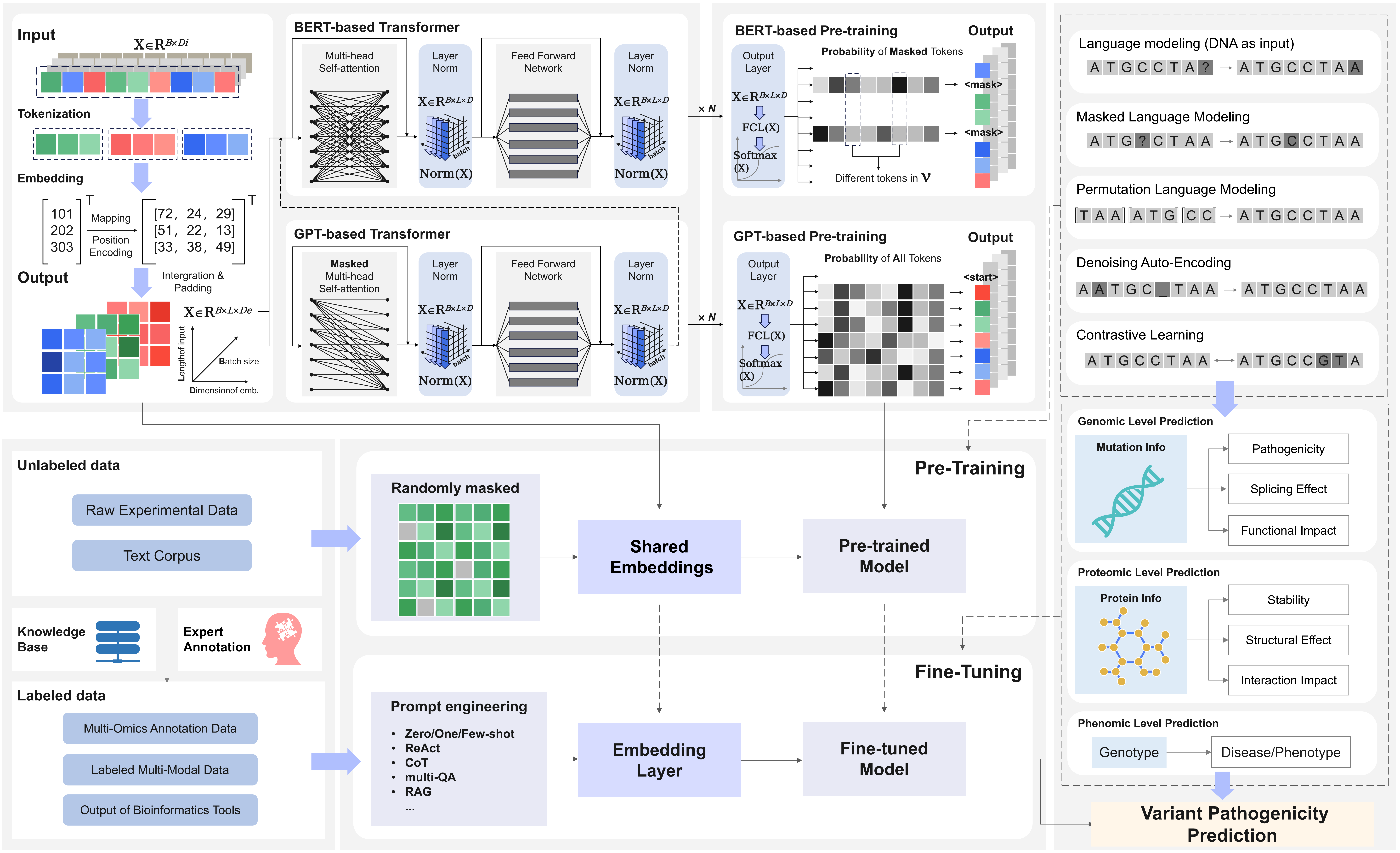

Input
Tokenization
Embedding
Mapping
Position Encoding
Output
Intergration & Padding
Batch size
Lengthof input
Dimensionof emb.
BERT-based Transformer
Multi-head Self-attention
Layer Norm
Feed Forward Network
Norm(X)
× N
GPT-based Transformer
Masked Multi-head Self-attention
BERT-based Pre-training
Output
Output Layer
FCL(X)
Softmax (X)
Probability of Masked Tokens
<mask>
Different tokens in V
GPT-based Pre-training
Probability of All Tokens
<start>
Language modeling (DNA as input)
Masked Language Modeling
Permutation Language Modeling
Denoising Auto-Encoding
Contrastive Learning
Genomic Level Prediction
Mutation Info
Pathogenicity
Splicing Effect
Functional Impact
Proteomic Level Prediction
Protein Info
Stability
Structural Effect
Interaction Impact
Phenomic Level Prediction
Genotype
Disease/Phenotype
Variant Pathogenicity Prediction
Unlabeled data
Raw Experimental Data
Text Corpus
Knowledge Base
Expert Annotation
Labeled data
Multi-Omics Annotation Data
Labeled Multi-Modal Data
Output of Bioinformatics Tools
Pre-Training
Randomly masked
Shared Embeddings
Pre-trained Model
Fine-Tuning
Prompt engineering
Zero/One/Few-shot
ReAct
CoT
multi-QA
RAG
...
Embedding Layer
Fine-tuned Model

Click here to access/download
**Supplementary Material**
point-by-point-response 20260105.docx

Supplementary table

Click here to access/download
**Supplementary Material**
Supplementary Table 1_20260105.docx
