# Supplementary material for: Harnessing artificial intelligence for genomic variant prediction: advances, challenges, and future directions
Source: Gigascience. 2026 Jan 10;15:giag004. doi: 10.1093/gigascience/giag004 (PMC12888390; doi:10.1093/gigascience/giag004)
Supplement: giag004_Authors_Response_To_Reviewer_Comments_original_submission [file giag004_authors_response_to_reviewer_comments_original_submission.pdf]

Author's Response To Reviewer Comments

Dear Dr. Ma,
Thank you for the opportunity to revise our manuscript based on the thoughtful feedback provided by the expert reviewer. We have carefully considered all comments and suggestions and have made substantial revisions to address each point raised.
A summary of the key revisions is provided below:
1.Moderated claims regarding transformer performance to emphasize context-dependent gains, and added a new “Implications and Limitations” section separating evidence-based conclusions from future directions.
2.Enhanced quantitative context through a new comparative table (Table 3) summarizing representative models and their reported performance metrics, with explicit caution regarding cross-study comparability.
3.Clarified performance reporting across methodological phases, justifying our emphasis on protein language models while avoiding potentially misleading comparisons.
4.Expanded discussion of ancestry bias, incorporating algorithmic strategies such as transfer learning, domain adaptation, parameter-efficient fine-tuning, fairness-aware optimization, and ancestry-aware embeddings.
5.Addressed limitations for complex traits, clarifying constraints of single-variant predictors and expanding future directions toward polygenic modeling approaches.
6.Refined the Background section to clearly distinguish established knowledge, unresolved challenges, and the novel contribution of this review.
7.Improved search strategy transparency, including verification searches in additional databases.
8.Expanded discussion of the clinician’s role, emphasizing AI as decision-support within established diagnostic workflows.
9.Strengthened the multi-omics integration discussion, highlighting selective, context-dependent integration strategies alongside practical implementation.
10.Clarified the interpretation of novel variants, distinguishing variant discovery from AI-based interpretation of rare and unannotated variants.
11.Explicitly addressed erroneous predictions, emphasizing probabilistic outputs, explainable AI approaches, and the need for orthogonal validation.
We believe these revisions have further strengthened the manuscript and addressed all reviewer concerns comprehensively. Thank you for considering this revised version for publication. We look forward to your response.

Yours sincerely
Mingyan Fang on behalf of all authors

Reviewer #1:
This manuscript provides a comprehensive and well-structured review of artificial intelligence methods for genomic variant pathogenicity prediction. It effectively traces the evolution from rule-based systems to transformer architectures and integrates discussion of databases, model interpretability, and clinical translation. The topic is timely and relevant to the GigaScience community. I have few comments:
Author Response: We sincerely thank you for the thoughtful evaluation and positive assessment of our manuscript. We are pleased that you find our work comprehensive, timely, and relevant to the GigaScience community. We have carefully addressed all comments raised and believe the revisions have strengthened the manuscript substantially, with detailed point-by-point responses provided below.
1) The discussion is insightful but should moderate claims such as "transformer architectures consistently outperform prior methods. Consider adding a brief "Implications and Limitations" section to clearly separate evidence-based conclusions from forward-looking speculation.
Author Response: We appreciate this constructive feedback, which has prompted us to examine the nuance of our performance claims more carefully. While the comparative advantages of transformer architectures are well-documented across multiple studies, the magnitude and consistency of these improvements vary substantially depending on variant type, dataset composition, and evaluation framework. We agree that more explicit acknowledgment of context-dependency and benchmarking variability would enhance the manuscript's precision.
We have revised the manuscript to moderate claims regarding transformer performance through two main

approaches:
1) Refined performance characterizations throughout the manuscript to explicitly acknowledge context-dependency. We replaced categorical statements about consistent superiority with more nuanced phrasing that recognizes variability across variant types, dataset compositions, and evaluation metrics. These revisions appear primarily in Section 3 “The Technological Trajectory of Variant Pathogenicity Assessment” where transformer architectures are discussed (Page 12, Lines 268-273).
2) Added an “Implications and Limitations” subsection (Pages 22-23, Lines 489-504) that highlights key considerations for interpreting reported benchmark results, including limitations in cross-study comparability, validation, interpretability, and population representation. This section also clarifies that AI-based variant interpretation tools are best viewed as decision-support systems within established clinical workflows, rather than standalone clinical classifiers.
We believe this addition improves conceptual clarity and appropriately moderates claims while preserving the manuscript’s forward-looking scope. These revisions strengthen the manuscript's scientific rigor while preserving our assessment of genuine advances in transformer-based variant interpretation. We thank you for helping us achieve this improved balance.

2) While no new analyses are conducted, the description of performance metrics is accurate. Including a short comparative table of representative models with reported AUC/sensitivity values (from cited literature) would provide useful quantitative context.
Author Response:
We thank the reviewer for this helpful suggestion and agree that providing concise quantitative context enhances the manuscript’s value for readers seeking to understand performance trends across different methodological paradigms. In response, we have added a new comparative table (Page 13, Table 3; Lines 276-279) to the main text.
Table 3 summarizes representative variant pathogenicity predictors spanning major methodological phases, including rule-based, statistical, machine learning, deep learning, and transformer/protein language model–based approaches. For each representative tool, we report the primary variant class addressed and the performance metrics as originally published (e.g., ROC-AUC, PR-AUC, or accuracy, where available). These examples are intended to illustrate the range of performance values reported across methodological generations, with particular emphasis on the increasing use of standardized, large-scale benchmarking practices in later methodological phases, rather than implying strict performance superiority across tools. To ensure appropriate interpretation, we explicitly state in the table caption and accompanying text that the reported metrics are derived from heterogeneous evaluation settings and are therefore not directly comparable across tools. Differences in datasets, variant class distributions, labeling standards, and benchmarking protocols substantially limit cross-study comparisons. Accordingly, the table is presented to provide historical and illustrative quantitative context, complementing the more detailed methodological descriptions and tool characteristics provided in Supplementary Table 1.
We believe this addition addresses your concern while maintaining appropriate scientific caution about cross-study comparisons.

Reviewer #2:
This review systematically examines the application of artificial intelligence (AI) in genetic variant interpretation, tracing the evolution from traditional methods to modern machine learning, deep learning, and protein language models. It successfully identifies major challenges in the field, such as data heterogeneity, interpretability, and the interpretation of Variants of Uncertain Significance (VUS). The topic is well-positioned and demonstrates significant scientific and clinical relevance, aligning closely with current trends in precision medicine. The article is well-structured, logically coherent, and features accurately chosen keywords, reflecting a comprehensive grasp of the subject area.
Author Response: We sincerely thank you for the thorough evaluation and positive feedback. We are pleased that you find our systematic examination comprehensive and well-aligned with current trends in precision medicine. We appreciate your recognition of the manuscript's structure, logical coherence, and identification of key challenges in the field. We have carefully addressed all your comments, with detailed point-by-point responses provided below.

To further strengthen the manuscript, the following enhancements are recommended:
1) In the section on Protein Language Models (Phase 5), the authors explicitly cite high performance metrics (e.g., ROC-AUC of 0.905 for ESM-1b). However, similar quantitative data is largely absent for the

tools discussed in earlier phases (Phases 1-4). Was this disparity in reporting intended to emphasize the specific performance leap of Transformer-based models, or does it reflect a lack of comparable benchmarks for earlier tools? Clarifying this would help justify why pLMs are presented as a significant advancement.
Author Response: We thank you for this insightful observation. The emphasis on specific metrics for pLMs was indeed intentional, designed to highlight the substantial performance gains enabled by Transformer architectures. However, we recognize that the relative lack of metrics for earlier phases could suggest an imbalanced presentation.
The disparity reflects both our initial editorial choice and practical constraints in the literature. Earlier phases (1-4) lack the standardized, large-scale benchmarking protocols that have become common in the deep learning era, making direct cross-phase comparisons scientifically problematic. Our original approach was to emphasize methodological evolution while avoiding potentially misleading numeric comparisons across heterogeneous evaluation frameworks.
In response to your feedback and Reviewer 1's suggestion for quantitative context, we have now addressed this in two complementary ways:
1) Added Table 3 in the main text (Page 13, Line 279) providing representative performance metrics across all methodological phases, with explicit caveats about comparability limitations.
2) Enhanced Supplementary Table 1 by adding a "reported performance" column that summarizes the performance values of all discussed tools. The table legend explicitly notes that metrics are study-specific and cross-tool comparisons should be interpreted cautiously.
Together, these additions provide readers with quantitative context for understanding the field's progression while maintaining appropriate scientific caution about heterogeneous benchmarking practices. We believe this approach better justifies the advancement represented by pLMs within a comprehensive historical framework.

2) The manuscript correctly identifies ancestry bias in reference databases as a critical barrier. To address this, the authors primarily suggest data-centric solutions such as targeted sequencing and federated learning. However, generating new population-scale data is time-consuming. Could the authors discuss if there are algorithmic or computational strategies available in recent literature that can improve the generalizability of current models to underrepresented populations without immediately requiring massive new datasets? Discussing how existing models might be adapted or fine-tuned for diverse ancestries would strengthen the practical utility of this review.
Author Response: We thank the reviewer for this excellent suggestion. We agree that while generating diverse population-scale datasets is essential in the long term, near-term algorithmic and computational strategies are needed to mitigate ancestry bias using existing resources.
In response, we have substantially expanded the discussion in the " Algorithmic Strategies for Polygenic Risk Score Transferability" section (Pages 21-22, Lines 459-475) to systematically highlight computational strategies that enhance model generalizability using existing resources. The revised discussion now organizes these approaches into four complementary categories:
1) Transfer Learning and Domain Adaptation: We discuss transfer learning methods that adjust existing models for population-specific linkage disequilibrium and allele frequency differences using available summary statistics [130, 131] [PMID: 36240765; 36061179]. Domain adaptation techniques based on adversarial learning are also noted as a means to construct ancestry-invariant representations, improving cross-population generalizability [132,133] [Corpus ID: 4357800; PMID: 38160290].
2) Synthetic Data Generation: The revised section briefly describes the use of generative models, such as GAN-based approaches, to augment limited real-world data with synthetic genomes, thereby partially alleviating data scarcity in underrepresented populations [134,135] [PMID: 33539374; Corpus ID: 251136196].
3) Ensemble-based PRS methods: We highlight supervised ensemble frameworks, including CT-SLEB and related multi-ancestry penalized regression approaches, which integrate information across multiple PRS construction strategies to improve predictive performance in diverse populations [136,137] [PMID: 37749244; 36993331].
4) Unsupervised and Zero-shot Approaches: we note emerging unsupervised ensemble methods that aggregate pre-trained PRS models based on prediction concordance, enabling adaptation to target populations without requiring labeled phenotype data [138] [PMID: 39830281].
Although several of these strategies were originally developed in the context of polygenic risk scores, the underlying principles of transfer learning, domain adaptation, and ancestry-invariant representation learning are increasingly being adapted to variant-level pathogenicity prediction models as well.

We believe these additions further strengthen the practical utility of our review by providing readers with actionable computational strategies that can be implemented in the near term while large-scale data generation efforts progress. We appreciate you highlighting this important gap in our original manuscript.

3) The manuscript focuses heavily on predictors for Mendelian diseases. However, the applicability of these tools to complex, polygenic diseases is not explicitly addressed, given that such conditions are driven by cumulative small-effect variants. Could the authors clarify the limitations of current single-variant predictors for complex traits? Additionally, expanding the 'Future Directions' section to discuss how future AI frameworks might model complex polygenic interactions would significantly strengthen the manuscript's forward-looking perspective.
Author Response: We thank the reviewer for this important observation regarding the applicability of variant predictors to complex, polygenic diseases. We agree that distinguishing between Mendelian and polygenic architectures is crucial for a forward-looking review.
In response to your feedback, we have made two substantial additions to the manuscript:
1) Added a new subsection " Modeling Polygenic and Complex Traits" (Page 17, Lines 364-375) which clarifies the limitations of current single-variant predictors, such as AlphaMissense in capturing the "omnigenic" architecture and non-linear epistatic interactions of polygenic diseases [PMID: 28622505].
2) Expanded the "Future Directions" section with a new subsection " Transitioning to Polygenic Modeling" (Page 22, Lines 476-487) that discusses the shift from single-variant scoring to systems-level polygenic modeling. This addition discusses emerging directions that move beyond isolated variant scoring, including ensemble-based approaches that integrate polygenic background with rare variant burden (e.g., polygenic risk predictions integrating common and rare variants [139]) and graph-based neural network models such as PRS-Net [140], which offer a framework for capturing non-linear gene–gene and pathway-level interactions [141].
[DOI: doi.org/10.1101/2024.11.05.24316779; doi.org/10.1007/978-1-0716-3989-4_35; PMID; 39562137].
These additions clarify the current limitations of single-variant predictors for complex traits while providing a comprehensive forward-looking perspective on how AI frameworks are evolving to address polygenic architecture. We believe this further strengthens the manuscript's scope and practical relevance across different genetic architectures. We appreciate your guidance in addressing this important dimension of variant interpretation.

Reviewer #3:
In this review the authors discussed the evolution from traditional rule-based systems and statistical models to contemporary machine learning, deep learning, and protein language models, while addressing critical challenges in variant classification. The topic is interesting. Some concerns and suggestions are listed as below:
Author Response: We sincerely thank you for the positive feedback and for recognizing the significance of tracing the evolution of variant classification methodologies. We appreciate your interest in the topic and have carefully considered all your concerns and suggestions. We believe the revisions have substantially improved the manuscript, and detailed point-by-point responses are provided below.

1) The authors performed a narrative, method-focused search of PubMed and Google Scholar combining terms such as variant pathogenicity, in silico prediction, deep learning, transformer/foundation model, splicing prediction, non-coding variants, functional assays, and database/tool names. This exclusion of other databases may introduce selection bias, as significant research may have been published in other languages or databases.
Author Response: We thank the reviewer for this important methodological consideration regarding potential selection bias from our search strategy.
Our search strategy, which focused primarily on PubMed, Google Scholar, and Scopus, was designed to capture the dominant methodological frameworks and tools in computational genomics. The field is characterized by a strong convention of publishing major tools and methodological advances in English-language, internationally indexed journals and repositories to ensure broad adoption, reproducibility, and citation. This convention makes a search of these core databases particularly effective for a review tracing influential approach.
Regarding database coverage, we acknowledge the theoretical concern about missing important work indexed in other databases. To address this, we performed a validation check of our included tools against

Scopus indexing. We found that all tools discussed in our review are indexed in Scopus, with the exception of three very recent preprints (AlphaGenome, Genetic Transformer, MPC) that have not yet undergone peer review. This suggests that our PubMed and Google Scholar search strategy, combined with extensive citation tracking and manual examination of tool repositories, effectively captured the major tools and frameworks in the field without systematic gaps.
We note that attempting a comprehensive keyword-based search across all tools proved impractical due to the heterogeneity of tool names, methodological descriptors, and evolving terminology in this rapidly developing field. Instead, our narrative review approach prioritized identifying influential methodological frameworks and widely adopted tools through iterative literature exploration, forward and backward citation tracking, and consultation of major tool repositories and benchmarking studies.
We have updated the Methods section (Page 4, Lines 75-83) to more explicitly describe our search strategy, including the validation check against Scopus indexing, and to clarify that as a narrative review focused on methodological evolution, our goal is to trace influential approaches that have shaped the field rather than to achieve exhaustive cataloging of all related publications.

2) The authors should introduce what is known and unknown in this field (AI for genomic variant prediction). What about new information in current review? The authors mentioned too many general concepts.
Author Response: We thank the reviewer for this important comment. In response, we have substantially revised the Background to clearly distinguish what is currently well established in the field from what remains unresolved, and to articulate the specific contribution of this review.
The revised introductory paragraphs (Pages 3-4, Lines 54-74) now explicitly contrast empirically supported advances with persistent challenges. We acknowledge that ML and DL approaches have demonstrably improved pathogenicity prediction for many coding variants and that sequence-based models have advanced the interpretation of splicing and regulatory variants. However, we also identify critical gaps that limit clinical translation: a substantial proportion of variants remain classified as VUS; reference datasets exhibit ancestry imbalances that compromise generalizability; deep learning models often operate as "black boxes," complicating alignment with ACMG/AMP guidelines; and experimental validation lags behind computational predictions.
Regarding the contribution of this review, we have clarified that rather than introducing new predictive algorithms or repeating general concepts, we provide a structured, method-focused synthesis that: (1) maps the technological evolution of VIPs across computational paradigms, (2) characterizes the supporting database ecosystem and practical workflows for model development and evaluation, (3) critically examines translational barriers to clinical implementation, and (4) provides actionable recommendations for model selection, ancestry-aware algorithm design, multi-omics integration strategies, and validation frameworks. The analytical purpose is to clarify where current AI approaches are robust, where caution is warranted, and where further validation is most urgently needed. We believe this revised structure directly addresses the reviewer's concern by moving beyond general concepts to provide a clear framework that distinguishes empirical evidence from unresolved questions while emphasizing the practical, translational focus of our synthesis.

3) The V2P (Variant to Phenotype), is designed to accelerate genetic diagnostics and aid in the discovery of new treatments for complex and rare diseases. This point should be discussed.
Author Response: We thank the reviewer for this valuable suggestion to discuss the Variant-to-Phenotype (V2P) framework and its contributions to accelerating genetic diagnostics and therapeutic discovery.
We fully agree that linking molecular variants to specific clinical manifestations represents a critical evolution in the field. This phenotype-conditioned approach addresses the fundamental challenge of allelic heterogeneity and transforms variant interpretation from binary classification into a clinically actionable framework aligned with patient-specific pathology.
In response, we have added a new subsection entitled “Phenotype-Conditioned Variant Interpretation” (Pages 18-19, Lines 402-409). This section introduces phenotype-conditioned approaches that address allelic heterogeneity by conditioning pathogenicity predictions on clinical phenotype information. We specifically describe the Variant-to-Phenotype (V2P) framework as an example of a multi-task architecture that integrates protein interactomes, structural descriptors, and evolutionary constraints to generate predictions conditioned on Human Phenotype Ontology (HPO) categories.
The revised text highlights how modeling genotype phenotype associations can support rare disease diagnosis and facilitate the identification of phenotype-relevant therapeutic targets, thereby enhancing the

clinical interpretability of variant scores. We believe this addition strengthens the review by illustrating how emerging AI frameworks move beyond phenotype-agnostic variant scoring toward more clinically contextualized interpretation.

4) The manuscript should be edited. For example, 'Variants of uncertain significance (VUS)' should be 'VUS' in line 316.
Author Response: We thank you for this observation. We have replaced “Variants of uncertain significance (VUS)” with the abbreviated form 'VUS' in the revised manuscript (Page 16, Line 343) and ensured consistent use of abbreviations throughout the document.

5) How AI gets access to big training genetic data? This point should be discussed in details.
Author Response: We thank the reviewer for highlighting this important aspect of AI model development in genomic variant interpretation. In current practice, AI-based variant pathogenicity predictors are trained on large-scale datasets assembled by researchers from multiple complementary data sources.
As described throughout the manuscript, these training resources typically include population-scale variant databases, curated clinical annotation repositories, protein sequence and structure databases, and large collections of genomic sequences. In particular, recent transformer-based and foundation models leverage extensive unlabeled sequence corpora through self-supervised learning, which enables model pretraining at scale without requiring phenotype-linked or patient-identifiable information.
We address these data sources throughout the manuscript:
1) Section 2 comprehensively reviews the database ecosystem (Pages 4-6, Lines 85-124), including data provenance, curation processes, and access mechanisms.
2) Section 5.2 and 5.3 discusses dataset limitations and biases arising from data collection methods (Pages 16-17, Lines 342-363).
We further clarify that most large-scale training resources used by current AI models are publicly accessible or controlled-access, de-identified datasets governed by database-specific usage and ethical guidelines (e.g., data access committees and data use agreements), which limits direct exposure to individual-level clinical or identifiable genetic data (Page 6, Lines 122-124; Page 17, Lines 360-362).
Together, these discussions provide context for how large-scale genetic data are assembled and utilized for training AI-based variant interpretation models, as well as the practical and conceptual limitations associated with these approaches.

6) The role of clinician in AI-driven variant interpretation should be discussed in details. I wonder if clinicians need to know all computational tools (explainable and user-friendly tools?) in this case.
Author Response: We thank the reviewer for raising this important point regarding the role of clinicians in AI-driven variant interpretation. We agree that AI-based tools should support, rather than replace, clinical expertise and that clinicians are not expected to understand the underlying computational or machine learning architectures.
In response, we have added a new section entitled “Clinical Role in AI-based Variant Interpretation” (Page 18, Lines 386-396). This section clarifies that AI-based variant impact predictors function as decision-support tools within established clinical workflows, with clinicians retaining responsibility for final variant classification. The revised text emphasizes that effective clinical use depends on interpretable outputs, confidence estimates, and traceable evidence aligned with ACMG/AMP guidelines, rather than detailed knowledge of individual computational models.
We also describe how clinicians integrate AI-derived predictions with patient phenotype, family history, segregation analysis, and functional evidence to reach informed classification decisions. We believe this addition directly addresses the reviewer’s question and clarifies the clinician’s role in AI-assisted variant interpretation.

7) Regarding effectively integrating multi-omics information, more texts should be added.
Author Response: We thank the reviewer for their valuable suggestion to expand the discussion on effective multi-omics integration. In response, we have added a focused paragraph to the manuscript that strengthens the conceptual discussion of how multi-omics information is incorporated into current variant interpretation approaches (Page 19, Lines 410-422).
The revised section emphasizes that effective multi-omics integration is typically selective rather than uniform, with omics layers prioritized according to variant class and disease context. We provide illustrative examples highlighting the relevance of transcriptomic and splicing data for non-coding and splice-site

variants, and proteomic or structural information for missense variants affecting protein function.
In addition, the paragraph outlines common integration strategies used in AI-based frameworks, including feature-level aggregation, model-level fusion, and post hoc evidence synthesis aligned with ACMG/AMP guidelines. We also briefly acknowledge practical challenges such as data heterogeneity, tissue specificity, and incomplete population representation, and note that prioritization of high-value omics layers and shared databases may facilitate scalable clinical application.
We believe this addition enhances the clarity of the discussion on multi-omics integration while remaining consistent with the scope and focus of the review.

8) How AI founds new genetic variants?
Author Response: Thank you for raising this question. We note that the technical detection of variants from sequencing data (variant calling) is typically performed by bioinformatics pipelines rather than AI-based predictors. Our review focuses specifically on AI's role in interpreting and prioritizing variants once they have been identified.
To clarify AI's role in interpreting novel variants, we have revised the Data Limitations and Functional Validation Bottlenecks section to explicitly describe how AI-based models support the interpretation and prioritization of rare and novel variants that lack prior annotation or functional evidence (Page 16, Lines 343-347). As now discussed, AI approaches leverage patterns learned from evolutionary conservation, regulatory annotations, and protein structural features to infer potential functional impact and contextualize variants absent from existing databases. This is particularly valuable given that VUS continue to represent a large fraction of clinical findings, especially for rare and non-coding variants.
We believe the revised text now clarifies how AI contributes to the interpretation of novel variants within the scope of this review.

9) Some predictions from AI may turn out to be quite wrong. How did you address this issue?
Author Response: Thank you for this important concern regarding erroneous predictions from AI models. We agree that addressing the risk of false positives and false negatives is essential for clinical safety and have discussed this issue from multiple perspectives throughout the manuscript:
1) Model positioning: We have revised the Model Interpretability versus Predictive Power section (Pages 17-18, Lines 376-385) to explicitly clarify that AI-based predictors are probabilistic prioritization tools rather than definitive classifiers, requiring cross-validation with orthogonal clinical and experimental evidence. As discussed in the revised text, explainable AI (XAI) frameworks help mitigate the risk of 'false confidence' in erroneous outputs by providing biological rationales, allowing clinicians to distinguish between robust predictions and potential algorithmic artifacts.
2) Clinical integration: The Clinical Role in AI-based Variant Interpretation section (Page 18, Lines 386-396) emphasizes that clinicians retain responsibility for final classification decisions and must integrate AI predictions with patient phenotype, family history, segregation data, and functional evidence.
3) Validation requirements: Throughout the manuscript, we discuss the need for rigorous benchmarking, external validation, and functional validation to identify model limitations and reduce the risk of incorrect predictions affecting clinical decisions.
We believe these multi-layered safeguards positioning AI as decision support rather than replacement, requiring explainability, maintaining clinical oversight, and enforcing validation standards, collectively address the concern about erroneous predictions.

Cookie Preference Center

We use cookies which are necessary to make our site work. We may also use additional cookies to analyze, improve and personalize our content and your digital experience. For more information, see our Cookie Policy.

You may choose not to allow some types of cookies. However, blocking some types may impact your experience of our site and the services we are able to offer. See the different category headings below to find out more or change your settings.

Allow all

Manage Consent Preferences

Strictly Necessary Cookies

Always active

These cookies are necessary for the website to function and cannot be switched off in our systems. They are usually only set in response to actions made by you which amount to a request for services, such as setting your privacy preferences, logging in or filling in forms. You can set your browser to block or alert you about these cookies, but some parts of the site will not then work.

Functional Cookies

These cookies enable the website to provide enhanced functionality and personalisation. They may be set by us or by third party providers whose services we have added to our pages. If you do not allow these cookies then some or all of these services may not function properly.

Performance Cookies

These cookies allow us to count visits and traffic sources so we can measure and improve the performance of our site. They help us to know which pages are the most and least popular and see how visitors move around the site.

Targeting Cookies

These cookies may be set through our site by our advertising partners. They may be used by those companies to build a profile of your interests and show you relevant adverts on other sites. If you do not allow these cookies, you will experience less targeted advertising.

Cookie List

Clear

Apply Cancel
Consent Leg.Interest

Confirm my choices

Powered by onetrust
