# Supplementary material for: Harnessing artificial intelligence for genomic variant prediction: advances, challenges, and future directions
Source: Gigascience. 2026 Jan 10;15:giag004. doi: 10.1093/gigascience/giag004 (PMC12888390; doi:10.1093/gigascience/giag004)
Supplement: giag004_Reviewer_2_Report_original_submission — Weiyang Li -- 12/8/2025 [file giag004_reviewer_2_report_original_submission.pdf]

Reviewer Report

Title: Harnessing Artificial Intelligence for Genomic Variant Prediction: Advances, Challenges, and Future Directions

Version: Original Submission **Date:** 12/8/2025

Reviewer name: Weiyang Li

Reviewer Comments to Author:

This review systematically examines the application of artificial intelligence (AI) in genetic variant interpretation, tracing the evolution from traditional methods to modern machine learning, deep learning, and protein language models. It successfully identifies major challenges in the field, such as data heterogeneity, interpretability, and the interpretation of Variants of Uncertain Significance (VUS). The topic is well-positioned and demonstrates significant scientific and clinical relevance, aligning closely with current trends in precision medicine. The article is well-structured, logically coherent, and features accurately chosen keywords, reflecting a comprehensive grasp of the subject area.

To further strengthen the manuscript, the following enhancements are recommended:

1.In the section on Protein Language Models (Phase 5), the authors explicitly cite high performance metrics (e.g., ROC-AUC of 0.905 for ESM-1b). However, similar quantitative data is largely absent for the tools discussed in earlier phases (Phases 1-4). Was this disparity in reporting intended to emphasize the specific performance leap of Transformer-based models, or does it reflect a lack of comparable benchmarks for earlier tools? Clarifying this would help justify why pLMs are presented as a significant advancement.

2.The manuscript correctly identifies ancestry bias in reference databases as a critical barrier. To address this, the authors primarily suggest data-centric solutions such as targeted sequencing and federated learning. However, generating new population-scale data is time-consuming. Could the authors discuss if there are algorithmic or computational strategies available in recent literature that can improve the generalizability of current models to underrepresented populations without immediately requiring massive new datasets? Discussing how existing models might be adapted or fine-tuned for diverse ancestries would strengthen the practical utility of this review.

3.The manuscript focuses heavily on predictors for Mendelian diseases. However, the applicability of these tools to complex, polygenic diseases is not explicitly addressed, given that such conditions are driven by cumulative small-effect variants. Could the authors clarify the limitations of current single-variant predictors for complex traits? Additionally, expanding the 'Future Directions' section to discuss how future AI frameworks might model complex polygenic interactions would significantly strengthen the manuscript's forward-looking perspective.

Methods

Are the methods appropriate to the aims of the study, are they well described, and are necessary controls included? Choose an item.

Conclusions

Are the conclusions adequately supported by the data shown? Choose an item.

Reporting Standards

Does the manuscript adhere to the journal's guidelines on minimum standards of reporting? Choose an item.

Choose an item.

Statistics

Are you able to assess all statistics in the manuscript, including the appropriateness of statistical tests used? Choose an item.

Quality of Written English

Please indicate the quality of language in the manuscript: Choose an item.

Declaration of Competing Interests

Please complete a declaration of competing interests, considering the following questions:

- Have you in the past five years received reimbursements, fees, funding, or salary from an organisation that may in any way gain or lose financially from the publication of this manuscript, either now or in the future?
- Do you hold any stocks or shares in an organisation that may in any way gain or lose financially from the publication of this manuscript, either now or in the future?
- Do you hold or are you currently applying for any patents relating to the content of the manuscript?
- Have you received reimbursements, fees, funding, or salary from an organization that holds or has applied for patents relating to the content of the manuscript?
- Do you have any other financial competing interests?
- Do you have any non-financial competing interests in relation to this paper?

If you can answer no to all of the above, write 'I declare that I have no competing interests' below. If your reply is yes to any, please give details below.

I declare that I have no competing interests.

I agree to the open peer review policy of the journal. I understand that my name will be included on my report to the authors and, if the manuscript is accepted for publication, my named report including any attachments I upload will be posted on the website along with the authors' responses. I agree for my

report to be made available under an Open Access Creative Commons CC-BY license (http://creativecommons.org/licenses/by/4.0/). I understand that any comments which I do not wish to be included in my named report can be included as confidential comments to the editors, which will not be published.

Choose an item.

To further support our reviewers, we have joined with Publons, where you can gain additional credit to further highlight your hard work (see: https://publons.com/journal/530/gigascience). On publication of this paper, your review will be automatically added to Publons, you can then choose whether or not to claim your Publons credit. I understand this statement.

Yes Choose an item.
