# Supplementary material for: Harnessing artificial intelligence for genomic variant prediction: advances, challenges, and future directions
Source: Gigascience. 2026 Jan 10;15:giag004. doi: 10.1093/gigascience/giag004 (PMC12888390; doi:10.1093/gigascience/giag004)
Supplement: giag004_Reviewer_3_Report_original_submission — Jinming Han -- 12/17/2025 [file giag004_reviewer_3_report_original_submission.pdf]

Reviewer Report

Title: Harnessing Artificial Intelligence for Genomic Variant Prediction: Advances, Challenges, and Future Directions

Version: Original Submission **Date:** 12/17/2025

Reviewer name: Jinming Han

Reviewer Comments to Author:

In this review the authors discussed the evolution from traditional rule-based systems and statistical models to contemporary machine learning, deep learning, and protein language models, while addressing critical challenges in variant classification. The topic is interesting. Some concerns and suggestions are listed as below:
The authors performed a narrative, method-focused search of PubMed and Google Scholar combining terms such as variant pathogenicity, in silico prediction, deep learning, transformer/foundation model, splicing prediction, non-coding variants, functional assays, and database/tool names. This exclusion of other databases may introduce selection bias, as significant research may have been published in other languages or databases.
The authors should introduce what is known and unknown in this field (AI for genomic variant prediction). What about new information in current review? The authors mentioned too many general concepts.
The V2P (Variant to Phenotype), is designed to accelerate genetic diagnostics and aid in the discovery of new treatments for complex and rare diseases. This point should be discussed.
The manuscript should be edited. For example, 'Variants of uncertain significance (VUS)' should be 'VUS' in line 316.
How AI gets access to big training genetic data? This point should be discussed in details.
The role of clinician in AI-driven variant interpretation should be discussed in details. I wonder if clinicans need to know all computational tools (explainable and user-friendly tools?) in this case.
Regarding effectively integrating multi-omics information, more texts should be added.
How AI founds new genetic variants?
Some predictions from AI may turn out to be quite wrong. How did you address this issue?

Methods

Are the methods appropriate to the aims of the study, are they well described, and are necessary controls included? Choose an item.

Conclusions

Are the conclusions adequately supported by the data shown? Choose an item.

Reporting Standards

Does the manuscript adhere to the journal's guidelines on minimum standards of reporting? Choose an item.

Choose an item.

Statistics

Are you able to assess all statistics in the manuscript, including the appropriateness of statistical tests used? Choose an item.

Quality of Written English

Please indicate the quality of language in the manuscript: Choose an item.

Declaration of Competing Interests

Please complete a declaration of competing interests, considering the following questions:

- Have you in the past five years received reimbursements, fees, funding, or salary from an organisation that may in any way gain or lose financially from the publication of this manuscript, either now or in the future?
- Do you hold any stocks or shares in an organisation that may in any way gain or lose financially from the publication of this manuscript, either now or in the future?
- Do you hold or are you currently applying for any patents relating to the content of the manuscript?
- Have you received reimbursements, fees, funding, or salary from an organization that holds or has applied for patents relating to the content of the manuscript?
- Do you have any other financial competing interests?
- Do you have any non-financial competing interests in relation to this paper?

If you can answer no to all of the above, write 'I declare that I have no competing interests' below. If your reply is yes to any, please give details below.

none

I agree to the open peer review policy of the journal. I understand that my name will be included on my report to the authors and, if the manuscript is accepted for publication, my named report including any attachments I upload will be posted on the website along with the authors' responses. I agree for my report to be made available under an Open Access Creative Commons CC-BY license (http://creativecommons.org/licenses/by/4.0/). I understand that any comments which I do not wish to be included in my named report can be included as confidential comments to the editors, which will not be published.

Choose an item.

To further support our reviewers, we have joined with Publons, where you can gain additional credit to further highlight your hard work (see: https://publons.com/journal/530/gigascience). On publication of this paper, your review will be automatically added to Publons, you can then choose whether or not to claim your Publons credit. I understand this statement.

Yes Choose an item.
